# Nickela‐electrocatalyzed C−H Alkoxylation with Secondary Alcohols: Oxidation‐Induced Reductive Elimination at Nickel(III)

**DOI:** 10.1002/anie.201913930

**Published:** 2020-01-16

**Authors:** Shou‐Kun Zhang, Julia Struwe, Lianrui Hu, Lutz Ackermann

**Affiliations:** ^1^ Institut für Organische und Biomolekulare Chemie Georg-August-Universität Göttingen Tammannstrasse 2 37077 Göttingen Germany

**Keywords:** C−H alkoxylation, electrocatalysis, electrochemistry, nickel, oxygenation

## Abstract

Nickela‐electrooxidative C−H alkoxylations with challenging secondary alcohols were accomplished in a fully dehydrogenative fashion, thereby avoiding stoichiometric chemical oxidants, with H_2_ as the only stoichiometric byproduct. The nickela‐electrocatalyzed oxygenation proved viable with various (hetero)arenes, including naturally occurring secondary alcohols, without racemization. Detailed mechanistic investigation, including DFT calculations and cyclovoltammetric studies of a well‐defined C−H activated nickel(III) intermediate, suggest an oxidation‐induced reductive elimination at nickel(III).

## Graphical abstract

**II, III, or IV**: Challenging secondary C−H alkoxygenations were accomplished with a versatile nickel electrocatalyst. Mechanistic investigation through isolation experiments, cyclovoltammetry, and density functional theory (DFT) calculations provided support for a process involving oxidation‐induced reductive elimination. MQ=6‐methylquinoline.

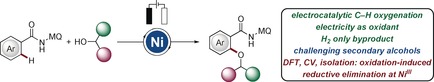

Transformations that form C−O bonds^1^ are of utmost importance in the synthesis of bioactive pharmaceuticals,^2^ natural products,^3^ and functional materials.^4^ Classical approaches for the synthesis of aryl ethers, such as the palladium‐catalyzed Buchwald–Hartwig cross‐couplings^5^ and copper‐catalyzed Ullmann–Goldberg^6^ or Chan–Evans–Lam reactions,^7^ rely on prefunctionalized substrates, the preparation and use of which result in undesired byproducts and solvent waste. In contrast, dehydrogenative functionalizations of otherwise inert C−H bonds constitute more sustainable strategies, which significantly reduce the footprint of organic syntheses.^8^ Despite major advances in C−H activation, C−H alkoxylations are less developed than typical hydroxylations,^9^ acetoxylations,^10^ and phenoxylations^11^ because competing β‐hydride elimination or overoxidation represent undesired side reactions. Specifically, C−H alkoxylations with sterically encumbered secondary alcohols continue to be difficult, which contrasts the wealth of viable methods for the use of primary alcohols.^12^

In recent years, electrosynthesis^13^ has gained significant attention through the use of waste‐free and inexpensive electric current as redox equivalent, thereby avoiding stoichiometric amounts of toxic and costly chemical redox reagents. Electrochemical C−H activations^14^ have until recently largely required expensive 5d and 4d metals, such as palladium,^15^ ruthenium,^16^ rhodium,^17^ and iridium.^18^ In sharp contrast, major recent momentum was gained by the use of earth‐abundant, less toxic 3d metals,^19^ such as cobalt^20^ and copper,^21^ as reported by the groups of Ackermann, Lei, and Mei, among others. In spite of the indisputable progress, such cost‐effective nickel electrocatalysis has proven elusive until very recently, when we established nickela‐electrocatalyzed C−H aminations, which were however restricted to morpholine‐type amines.^22^ In contrast, we have now found that versatile nickel catalysts are uniquely effective for challenging C−H electro‐alkoxylations with sterically encumbered secondary alcohols, which we report herein. It is noteworthy that complexes of cobalt, copper, and even precious palladium, iridium, ruthenium, and rhodium did not catalyze the difficult secondary C−H alkoxylations. In addition, we disclose mechanistic support for an oxidation‐induced reductive elimination nickel(III/IV) regime (Figure 1).

**Figure 1:**
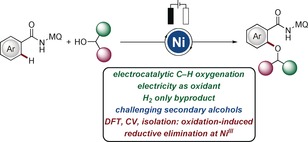
Nickela‐electrocatalyzed C−H alkoxylation with secondary alcohols: Mechanistic insights from isolation, CV, and DFT studies. MQ=6‐methylquinoline.

We began our studies by optimizing the reaction conditions for the envisioned nickela‐electrocatalyzed C−H oxygenation of amide **1 a** with the challenging secondary alcohol **2 a** in an undivided cell set‐up (Table 1 and Table S1–S7 in the Supporting Information). After considerable experimentation, the desired product **3 aa** was obtained with Ni(DME)Cl_2_ as the catalyst and bulky carboxylate NaO_2_CAd as an additive, whilst reticulated vitreous carbon (RVC) and nickel‐foam electrodes were found to be beneficial (entries 1–4). C−H acetoxylations were not observed. The performance of the catalysts was improved by adjusting the alcohol concentration (Table S4). Control experiments confirmed that the eletrooxidative C−H transformation could not be realized in the absence of electricity, the nickel complex, or the additive (entries 7–9). Other nickel compounds, such as Ni(COD)_2_, Ni(acac)_2_, or Ni(OAc)_2_ also furnished the desired product **3 aa** (entry 10, and Table S3). It is particularly noteworthy that the nickel catalysts featured proved uniquely effective for the challenging C−H activation with secondary alcohols, while other transition metals, including cobalt, copper, and even precious palladium, iridium, ruthenium, or rhodium, fell short under otherwise identical reaction conditions (entries 11–17 and Table S7). Indeed, while palladium, copper, and cobalt catalysts were highly effective for primary alcohols, no or very minor catalytic turnover was accomplished with the secondary alcohol **2 a** (Table S9).

**Table 1:** Optimization of the nickela‐electrocatalyzed secondary alkoxylation.^[a]^ 
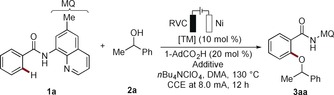

Entry	[TM]	Additive	**3 aa** [%]
1	Ni(DME)Cl_2_	NaOPiv	45^[b]^
2	Ni(DME)Cl_2_	NaO_2_CAd	55^[b]^
3	Ni(DME)Cl_2_	KOAc	24^[b]^
4	Ni(DME)Cl_2_	K_2_HPO_4_	–^[b]^
5	Ni(DME)Cl_2_	NaO_2_CAd	74
6	Ni(DME)Cl_2_	NaO_2_CAd	69^[c]^
7	–	NaO_2_CAd	–
8	Ni(DME)Cl_2_	–	–
9	Ni(DME)Cl_2_	NaO_2_CAd	–^[d]^
10	Ni(COD)_2_	NaO_2_CAd	67
11	Co(OAc)_2_⋅4 H_2_O	NaO_2_CAd	–
12	Mn(OAc)_2_	NaO_2_CAd	–
13	Cu(OAc)_2_⋅H_2_O	NaO_2_CAd	–
14	Ru(OAc)_2_(PPh_3_)_2_	NaO_2_CAd	–
15	[Cp*RhCl_2_]_2_	NaO_2_CAd	–^[e]^
16	Pd(OAc)_2_	NaO_2_CAd	–
17	[Cp*IrCl_2_]_2_	NaO_2_CAd	–^[e]^

[a] Reaction conditions: **1 a** (0.25 mmol), **2 a** (2.5 mmol), 1‐AdCO_2_H (20 mol %), [TM] (10 mol %), additive (1.0 equiv), *n*Bu_4_NClO_4_ (0.5 mmol), DMA (3.0 mL), constant current electrolysis (CCE) at 8.0 mA, 12 h, N_2_, RVC anode and Ni foam cathode, yield of isolated product. [b] **2 a** (1.25 mmol). [c] DMPU as solvent. [d] No current. [e] [TM] (5.0 mol %). DMA=*N*,*N*‐dimethylacetamide, DME=1,2‐dimethoxyethane, COD=cycloocta‐1,5‐diene, Cp*=1,2,3,4,5‐pentamethylcyclopenta‐1,3‐diene, Ad=1‐Adamantane, Piv=pivalic, DMPU=1,3‐dimethyltetrahydropyrimidin‐2(1H)‐one.

The efficacy of the nickela‐electrooxidation was considerably affected by the substitution pattern of the quinoline moiety (Scheme 1). Analysis by computation at the PEB0/Def2TZVP level of theory^23^ unraveled the key importance of increased electron‐density at the quinolinyl nitrogen, while decreased electron density at the amide nitrogen was beneficial (Figure S19 in the Supporting Information). These findings indicate the importance of increased σ‐donation at the sp^2^‐hybridized quinolinyl nitrogen in concert with an anionic amide nitrogen.

**Scheme 1:**
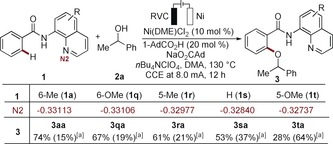
The power of directing‐groups for nickela‐electrooxidative alkoxylation. [a] Yields of recovered starting materials in parenthesis. N2=net atomic charges of the N2 atom

With the optimized reaction conditions in hand, we probed the versatility of the nickela‐electrocatalyzed C−H alkoxylation with various secondary alcohols **2** (Scheme 2). Not only benzylic alcohols **2 b** and **2 c** were well accepted, but also alicyclic, cyclic, and heterocyclic alcohols were successfully converted with moderate to excellent yields (**3 ab**–**3 ap**). Remarkably, the naturally occurring alcohols menthol, cholesterol, and β‐estradiol **2 q**–**2 s** were identified as viable substrates, notably without racemization at the stereogenic centers (Figures S1 and S2).

**Scheme 2:**
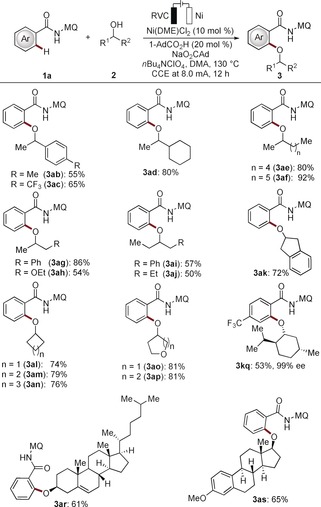
Electrooxidative C−H alkoxylation of arenes with secondary alcohols.

Moreover, we evaluated the robustness of the nickela‐electrocatalyzed C−H alkoxylation with a variety of functionalized benzamides **1** (Scheme 3). Thus, the reactions proceeded efficiently with arenes **1** bearing valuable functional groups, such as halo, sulfido, and cyano substituents. For the *meta*‐substituted substrates **1 b** and **1 c**, the reaction occurred with high position selectivity owing to repulsive steric interactions. The nickela‐electrocatalysis was not limited to arenes, but the heteroarene **1 o** was also selectively transformed. It is noteworthy that strongly coordinating pyridine was fully tolerated to give bidentate amide‐guided C−H functionalization (**3 pa**). Likewise, the gram‐scale synthesis was realized without compromising the efficacy on scale (**3 ba**).

**Scheme 3:**
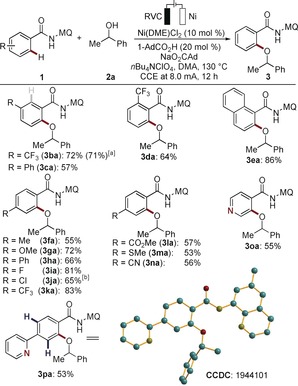
Electrooxidative C−H alkoxylation of arenes. [a] Gram‐scale testing with **1 b** (4.0 mmol,1.32 g). [b] 3.0 mA, 32 h.^28^

In addition, we carried out electricity on/off experiments to probe a radical‐chain scenario (Scheme 4 a). The reaction was halted without electrochemistry, however, the C−H alkoxylation continued when switching the electric current back on, thereby ruling out a radical‐chain process.

**Scheme 4:**
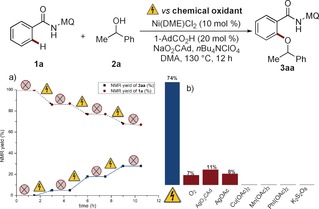
a) On/off experiment. b) Electrochemical versus chemical oxidants.

The clear benefits of electricity in this case were not restricted to it being a green and inexpensive oxidant. Indeed, the electrocatalytic reaction was characterized by significantly improved levels of performance as compared to the chemical oxidants AgOAc, Cu(OAc)_2_, molecular oxygen, PhI(OAc)_2_, or K_2_S_2_O_8_ (Scheme 4 b).

Given the unique performance of the nickela‐electrocatalyzed C−H activation, a series of experiments were conducted to gain insight into the reaction mechanism. Intermolecular competition experiments between secondary alcohol **2 p** and amine **2 t**, or with primary alcohol **2 u**, highlighted the particular challenge of nickela‐eletrooxidative secondary C−H alkoxylations (Scheme 5 a,b). In contrast to cobalta‐electrocatalysis by a BIES mechanism, an intermolecular competition experiment showed electron‐deficient arenes **1** to be inherently more reactive (Scheme 5 c). This finding is indicative of a concerted metalation–deprotonation (CMD) mechanism for the C−H activation.^24^ Head‐space gas‐chromatographic analysis identified H_2_ as the only stoichiometric byproduct (Figure S14). The electrocatalysis was inhibited by the typical radical scavengers TEMPO, BHT, and BQ, which is indicative of single‐electron transfer (SET) steps (Scheme 5 d). A minor kinetic isotope effect of *k*_H_/*k*_D_≈1.4 as measured by independent experiments gave support for a facile C−H scission (Figure S13). H/D exchange was not found when using isotopically labeled *t*BuOD as the additive (Figure S11). An irreversible nickelation^25^ was further found by DFT calculations to generate the substrate‐coordinated nickel(II) intermediate **Ni^II^‐II** (Figure S20). Thus, combined analysis by DFT and CV studies provided strong support for a viable nickel(II/III) oxidation (purple, Figure S22).

**Scheme 5:**
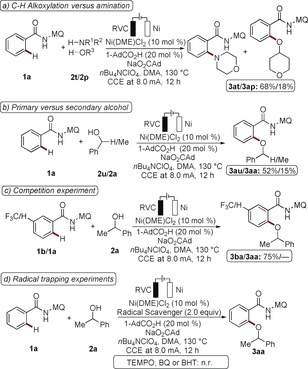
Summary of selected mechanistic findings. Conversions determined by ^1^H‐NMR analysis with 1,3,5‐(MeO)_3_C_6_H_3_ as the internal standard. TEMPO=(2,2,6,6‐tetramethylpiperidin‐1‐yl)oxyl, BQ=benzoquinone, BHT=2,6‐di‐tert‐butyl‐4‐methylphenol.

To rationalize the elementary process of C−O formation, the well‐defined nickel(III) complex **Ni^III^‐I** was independently synthesized, and fully characterized, including by X‐ray diffraction analysis (Scheme 6 a).^26^ **Ni^III^‐I** was competent in a catalytic and stoichiometric setting, provided that electricity was applied (Scheme 6 b,c). Cyclic voltammetric studies of **Ni^III^‐I** showed facile oxidation at a potential of 0.50 V vs. Fc^0/+^ (red, Scheme 6 e), thus suggesting the formation of a formal nickel(IV) complex.

**Scheme 6:**
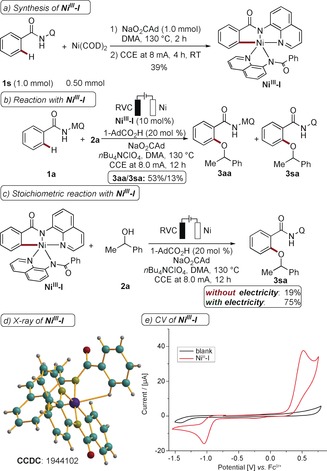
a) Synthesis of **Ni^III^‐I**. b, c) Catalytic and stoichiometric reactions with **Ni^III^‐I**, conversions determined by ^1^H‐NMR analysis with 1,3,5‐(MeO)_3_C_6_H_3_ as the internal standard. d) X‐ray diffraction analysis of **Ni^III^‐I**.^28^ e) CV data of **Ni^III^‐I** (DMA, 0.1 m [*n*Bu_4_NPF_6_], 100 mV s^−1^).

In good agreement with these results, DFT calculations indicate a non‐innocent ligand phenomenon in the oxidation process to generate a formal nickel(IV) species (Scheme 7). The oxidation is thus best described as a ligand‐centered process. Finally, high‐valent intermediate **Ni^IV^‐I** will be coordinated by the alcohol **2**, along with subsequent deprotonation and reductive elimination to furnish the alkoxylated products **3** (Figure S21).^26^

**Scheme 7:**
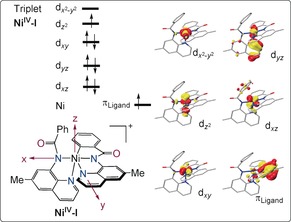
Calculated electronic configuration of **Ni^IV^‐I** ground triplet state.

As to the synthetic utility of this method, it is noteworthy that the 6‐methylquinuoline was easily removed in a traceless fashion to provide efficient access to benzamide **4**, benzoic acid **5**, or aromatic aldehyde **6** (Scheme S17–S19).

In summary, we have developed a carboxylate‐enabled nickela‐electrocatalyzed alkoxylations with challenging secondary alcohols. The robust electrochemical C−H activation was accomplished with broad substrate scope through the use of traceless removable quinoline amides. The most user‐friendly nickel electrocatalyst ensured high levels of both chemoselectivity and position selectivity. The C−H oxygenation was more effective with electricity than with any other chemical oxidant. Detailed mechanistic studies through isolation experiments, cyclovoltammetry, and computation provided strong support for an oxidation‐induced reductive elimination nickel(III/IV) manifold.

## Conflict of interest

The authors declare no conflict of interest.

## Supporting information

As a service to our authors and readers, this journal provides supporting information supplied by the authors. Such materials are peer reviewed and may be re‐organized for online delivery, but are not copy‐edited or typeset. Technical support issues arising from supporting information (other than missing files) should be addressed to the authors.

Supplementary
