# Supplementary material for: Nickela‐electrocatalyzed C−H Alkoxylation with Secondary Alcohols: Oxidation‐Induced Reductive Elimination at Nickel(III)
Source: Angew Chem Int Ed Engl. 2020 Jan 16;59(8):3178–83. doi: 10.1002/anie.201913930 (PMC7028089; doi:10.1002/anie.201913930)
Supplement: Supplementary file 1 — Supplementary [file ANIE-59-3178-s001.pdf]

Angewandte Chemie
Eine Zeitschrift der Gesellschaft Deutscher Chemiker

Supporting Information

Nickela-electrocatalyzed C−H Alkoxylation with Secondary Alcohols: Oxidation-Induced Reductive Elimination at Nickel(III)

*Shou-Kun Zhang, Julia Struwe, Lianrui Hu, and Lutz Ackermann**

anie_201913930_sm_miscellaneous_information.pdf

Table of Contents

General remarks S-2

Optimization of the reaction condition S-3

Testing of secondary alcohols following reported reaction methods S-11

General procedure for nickellaelectro-catalyzed C–H alkoxylation S-13

Characterization data for products S-15

Studies on the potential racemization of **3kq** S-47

X-Ray crystallographic analysis S-48

Mechanistic studies S-53

- Competition experiment S-53
- Deuteration experiment S-58
- KIE studies S-61
- Headspace analysis S-63
- Radical trapping experiment S-64
- The synthesis and catalysis analysis of **Ni(III)-I** S-65
- Switch off-on experiment S-70
- DFT calculation details S-71

Gram-scale experiment S-89

Traceless removal of the directing group S-90

Cyclic voltammetry S-93

References S-95

NMR spectra S-97

General remarks

Catalytic reactions were carried out in undivided electrochemical cells using pre-dried glassware under N_2, if not noted otherwise. As mentioned in the manuscript, hydrogen gas is generated during the reaction and can lead to an explosive gaseous mixture with atmospheric oxygen. Therefore, the reactions should be performed in a well-ventilated fume-hood and specialized reaction equipment should be used, when the reaction is performed in larger scale, e.g. electro-flow cells.[1] Amides **1** were synthesized according to previously described methods[2] and alcohols **2** were used as obtained by commercial sources. Nickel-foam electrodes (10 mm × 15 mm × 1.4 mm, RCM-Ni5763; obtained from Recemat BV, Germany) and RVC electrodes (10 mm × 15 mm × 6 mm, SIGRACELL®GFA 6 EA, obtained from SGL Carbon, Wiesbaden, Germany) were connected using stainless steel adapters. Electrocatalysis was conducted using an AXIOMET AX-3003P potentiostat in constant current mode. Cyclic Voltammetry studies were performed using a Metrohm Autolab PGSTAT204 workstation and Nova 2.0 software. Yields refer to isolated compounds, estimated to be >98% pure as determined by ^{1}H NMR. Chromatography: Merck silica gel 60 (40–63 µm). NMR: Spectra were recorded on a Varian Unity 300, Mercury 300, Inova 50.00 or Bruker Avance III 300, Bruker Avance III HD 400 and Bruker Avance III HD 50.00 in the solvent indicated; chemical shifts (δ) are given in ppm relative to the residual solvent peak. All IR spectra were recorded on a Bruker FT-IR Alpha device. MS: EI-MS- spectra were recorded on Jeol AccuTOF at 70 eV and ESI-MS-spectra on Bruker microTOF and maXis; High resolution mass spectrometry (HR-MS) with APEX IV 7T FTICR. M.p.: Stuart melting point apparatus SMP3, Barloworld Scientific, values are uncorrected. *In situ* IR measurements were carried out using ReactIR15 from Mettler Toledo equipped with a Diamond probe. Headspace analysis of the reaction mixture was performed on an Agilent 7890B GC System using a Thermal Conductivity Detector and a 5Å MS column. LC-MS measurements were performed on an Agilent 6100s Series Single Quad using RP column ZORBAX SB-C18, 5 µm.

Optimization of the reaction condition

Table S–1: Optimization of the electrolyte and redox mediator[a]

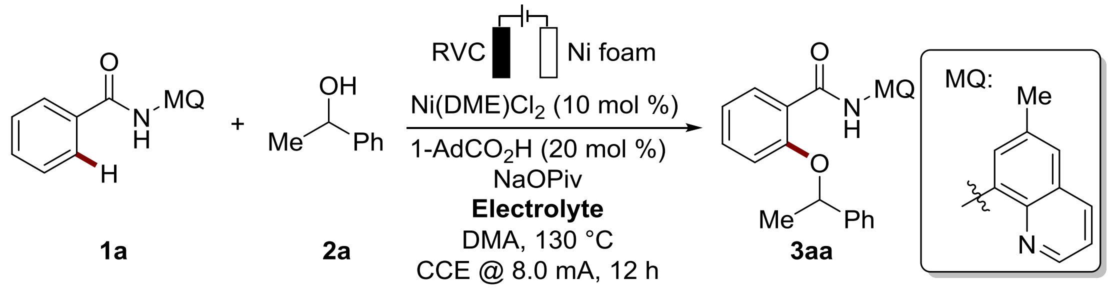

Entry	Electrolyte or redox mediator	Yield[b]
1	nBu$_4$NBF$_4$	42%
**2**	**nBu$_4$NClO$_4$**	**45%**
3	nBu$_4$NOAc	17%
4	nBu$_4$NPF$_6$	---
5	Ferrocene	---[c]
6	BQ	---[c]
7	nBu$_4$NI	23%[c]
8	LiClO$_4$	< 5%
9	nBu$_4$NClO$_4$	39%[c]
10	nBu$_4$NClO$_4$	46%[d]
11	nBu$_4$NClO$_4$	39%[e]

[a] Reaction conditions: **1a** (0.25 mmol), **2a** (1.25 mmol), Ni(DME)Cl$_2$ (10 mol %), 1-AdCO$_2$H (20 mol %), NaOPiv (0.25 mmol), electrolyte or redox mediator (0.50 mmol), DMA (3.0 mL), CCE = 8.0 mA, 12 h, 130 ^{o}C, N$_2$, RVC anode, nickel-foam cathode, DMA = *N,N*-Dimethylacetamide. BQ = 1,4-benzoquinone. [b] Isolated yield. [c] (0.125 mmol). [d] (1.0 mmol). [e] (0.25 mmol).

Table S–2: Optimization of the additive[a]

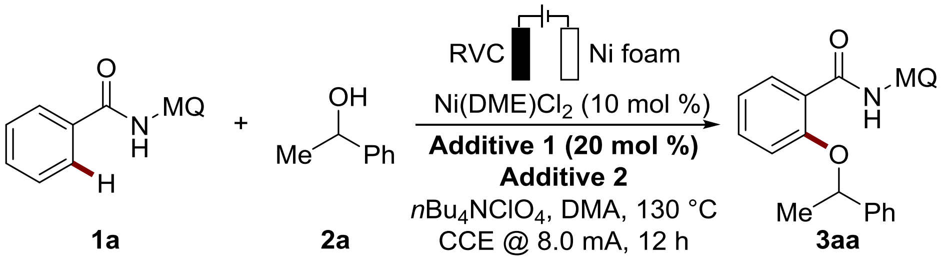

Entry	Additive 1	Additive 2	Yield[b]
1	1-AdCO$_2$H	NaOPiv	45%
2	1-AdCO$_2$H	NaO$_2$CAd	55%
3	---	NaO$_2$CAd	18%[c]
4	1-AdCO$_2$H	KOAc	24%
5	1-AdCO$_2$H	Cs$_2$CO$_3$	---
6	1-AdCO$_2$H	K$_2$HPO$_4$	---
7	1-AdCO$_2$H	KO$_2$CMes	---
8	1-AdCO$_2$H	KOPiv	32%
9	1-AdCO$_2$H	NaOtBu	---

[a] Reaction conditions: **1a** (0.25 mmol), **2a** (1.25 mmol), Ni(DME)Cl$_2$ (10 mol %), Additive 1 (20 mol %), Additive 2 (0.25 mmol), *n*Bu$_4$NClO$_4$ (0.50 mmol), DMA (3.0 mL), CCE = 8.0 mA, 12 h, N$_2$, 130 ^{o}C, RVC anode, nickel-foam cathode. [b] Isolated yield. [c] NaO$_2$CAd (0.5 mmol).

Table S–3: Optimization of nickel catalysts[a]

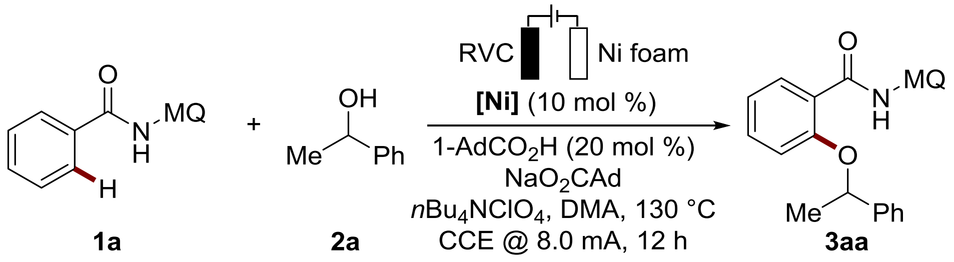

Entry	[Ni] (10 mol %)	Yield[b]
**1**	**$Ni(DME)Cl_2$**	**55%**
2	$Ni(OAc)_2{\cdot}4H_2O$	49%
3	$Ni(OTf)_2$	46%
4	$Ni(TFA)_2$	---
5	NiF_2	36%
6	NiI_2	15%
7	$NiSO_4{\cdot}6H_2O$	16%
8	$NiClO_4{\cdot}6H_2O$	---
9	$Ni(PPh_3)_2Cl_2$	45%
10	$NiCl_2$	41%
11	$Ni(NO_3)_2{\cdot}6H_2O$	39%
12	$Ni(BF_4)_2{\cdot}6H_2O$	---
13	$NiCO_3$	---
14	$Ni(acac)_2$	50%
15	$[Ni(bpy)_3][BF_4]_2$	---
16	$Ni(bpy)Cl_2$	---

[a] Reaction conditions: **1a** (0.25 mmol), **2a** (1.25 mmol), [Ni] (10 mol %), 1-AdCO_2H (20 mol %), NaO_2CAd (0.25 mmol), *n*Bu_4NClO_4 (0.50 mmol), DMA (3.0 mL), CCE = 8.0 mA, 12 h, N_2, 130 °C, RVC anode, nickel-foam cathode. [b] Isolated yield.

Table S–4: Optimization of further reaction parameters[a]

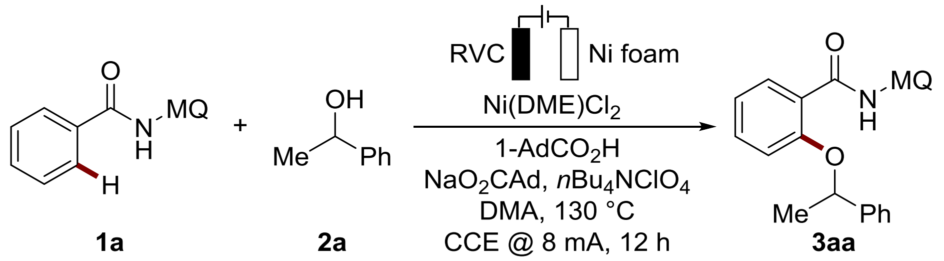

Entry	**2a** (equiv)	[Ni] (mol %)	T (^{o}C)	NaO_2CAd (equiv)	1-$AdCO_2H$ (mol %)	DMA (mL)	Yield[b]
1	5.0	5.0	130	1.0	20	3.0	21%
2	5.0	10	130	1.0	20	3.0	55%
3	5.0	20	130	1.0	20	3.0	60%
4	5.0	20	140	1.0	20	3.0	55%
5	5.0	20	120	1.0	20	3.0	54%
6	5.0	20	100	1.0	20	3.0	26%
7	5.0	20	130	2.0	20	3.0	61%
8	5.0	20	130	0.5	20	3.0	14%
9	5.0	20	130	---	20	3.0	---
10	5.0	20	130	1.0	20	4.0	61%
11	5.0	20	130	1.0	20	5.0	54%
12	10	20	130	1.0	20	3.0	72%
**13**	**10**	**10**	**130**	**1.0**	**20**	**3.0**	**74%**
14	10	10	130	1.0	20	3.0	69%[c]
15	3.0	10	130	1.0	20	3.0	34%
16	1.5	10	130	1.0	20	3.0	20%
17	10	10	130	1.0	50	3.0	57%
18	10	10	130	1.0	---	3.0	66%

[a] Reaction conditions: **1a** (0.25 mmol), **2a** (x mmol), $Ni(DME)Cl_2$ (x mol %), 1-$AdCO_2H$ (x mol %), NaO_2CAd (x mmol), $n$$Bu_4NClO_4$ (0. 50 mmol), DMA (x mL), CCE = 8.0 mA, 12 h, N_2, RVC anode, nickel-foam cathode. [b] Isolated yield. [c] DMPU as the solvent, DMPU = 1,3-dimethyltetrahydropyrimidin-2(*1H*)-one.

Table S–5: Control experiments[a]

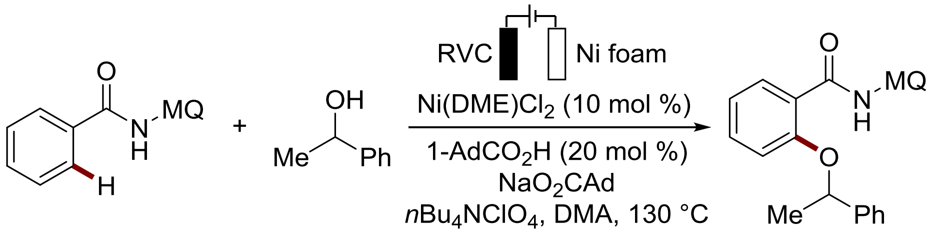

Entry	Variations from the Standard condition	Yield[b]
1	---	74%
2	Without current	---
3	Without $Ni(DME)Cl_2$	---
4	Without 1-$AdCO_2H$	66%
5	Without NaO_2CAd	---
6	Without $n$$Bu_4NClO_4$	50%
7	Under air	30%
8	Under O_2	47%
9	Under air without current	6%
10	Under O_2 without current	7%

[a] Standard conditions: **1a** (0.25 mmol), **2a** (2.5 mmol), $Ni(DME)Cl_2$ (10 mol %), 1-$AdCO_2H$ (20 mol %), NaO_2CAd (0.25 mmol), $n$$Bu_4NClO_4$ (0.50 mmol), DMA (3.0 mL), CCE = 8.0 mA, 12 h, N_2, 130 ^{o}C, RVC anode, nickel-foam cathode. [b] Isolated yield.

Table S–6: Electrochemical *vs.* chemical C–H oxygenation[a]

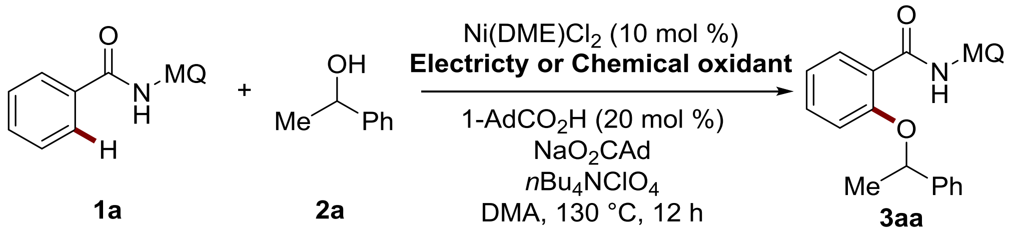

Entry	Oxidant	Yield[b]
1	(CCE = 8.0 mA, 12 h)	74%
2	$Cu(OAc)_2 \cdot H_2O$	---
3	$Mn(OAc)_3 \cdot 2H_2O$	---
4	$PhI(OAc)_2$	---
5	$K_2S_2O_8$	---
6	AgOAc	8%
7	$1\text{-}AdCO_2Ag$	11%

[a] Reaction conditions: **1a** (0.25 mmol), **2a** (2.5 mmol), $Ni(DME)Cl_2$ (10 mol %), Chemical oxidant (1.0 equiv), $1\text{-}AdCO_2H$ (20 mol %), NaO_2CAd (0.25 mmol), $n Bu_4NClO_4$ (0.50 mmol), DMA (3.0 mL). [b] Isolated yield.

Table S–7: The advantage of nickel over other transition metals[a]

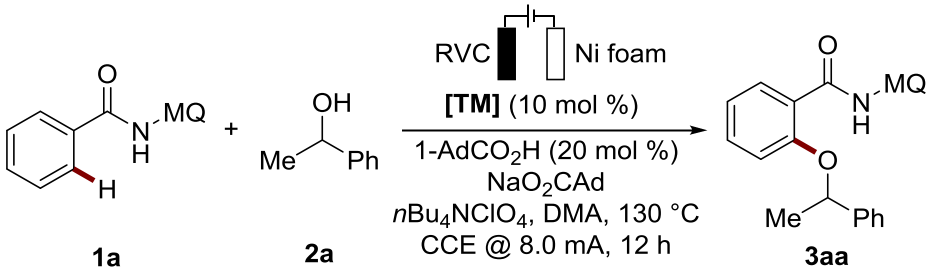

Entry	Catalyst	Yield[b]
**1**	**Ni(DME)Cl_2**	**74%**
2	Ni(COD)$_2$	67%
3	Co(OAc)$_2$ ·4H_2O	---
4	Fe(acac)$_3$	---
5	Mn(CO)$_5$Br	---
6	Mn(OAc)$_2$	---
7	Mn(OAc)$_3$ ·H_2O	---
8	Cu(OAc)$_2$ ·H_2O	---
9	Ru(OAc)$_2$(PPh$_3$)$_2$	---
10	[Cp*RhCl$_2$]$_2$ (5.0 mol %)	---
11	Pd(OAc)$_2$	---
12	[Cp*IrCl$_2$]$_2$ (5.0 mol %)	---

[a] Reaction conditions: **1a** (0.25 mmol), **2a** (2.5 mmol), [TM] (10 mol %), 1-AdCO$_2$H (20 mol %), NaO$_2$CAd (0.25 mmol), *n*Bu$_4$NClO$_4$ (0.50 mmol), DMA (3.0 mL), CCE = 8.0 mA, 12 h, N_2, 130 °C, RVC anode, nickel-foam cathode. [b] Isolated yield.

Table S–8: Directing group power for the nickellaelectrooxidative C–H oxygenation[a]

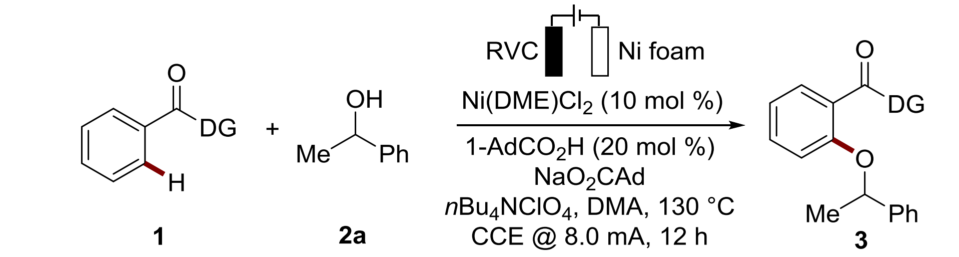

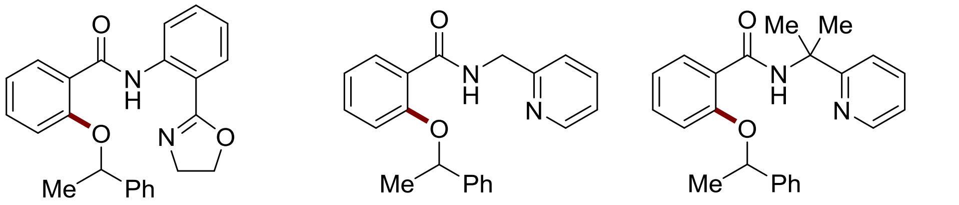

3ua: ---   **3va**: ---   **3wa**: ---

[a] Reaction conditions: **1** (0.25 mmol), **2a** (2.5 mmol), Ni(DME)Cl_2 (10 mol %), 1-AdCO_2H (20 mol %), NaO_2CAd (0.25 mmol), $n$$Bu_4NClO_4$ (0.50 mmol), DMA (3.0 mL), CCE = 8.0 mA, 12 h, N_2, 130 ^{o}C, RVC anode, nickel-foam cathode, isolated yield.

Testing of secondary alcohols following reported literature methods

Table S–9: Known methods with secondary alcohol

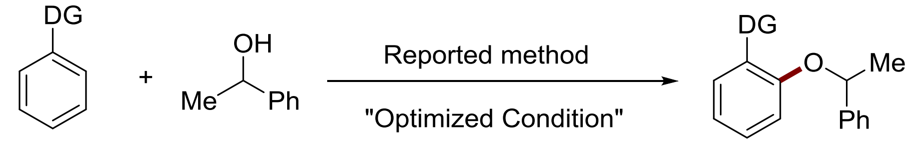

1 + **2a** → **3**

Entry	Substrate	Usage of alcohol **2a**	[TM]	Isolated Yield
1 ref. 2b	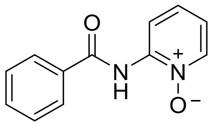 (0.2 mmol)	1.5 mL **(62 equiv)**	$Co(OAc)_2$	10%
2 ref. 3a	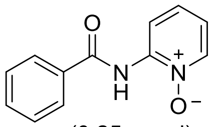 (0.25 mmol)	2.3 mL **(76 equiv)**	$Co(OAc)_2$	n.r.
3 ref. 3b	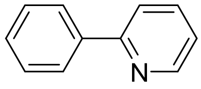 (0.5 mmol)	2.0 mL **(33 equiv)**	$Ni(acac)_2$	n.r.
4 ref. 3c	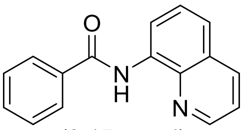 (0.15 mmol)	0.9 mL **(50 equiv)**	$Ni(OAc)_2$	18%
5 ref. 3d	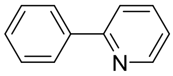 (1.0 mmol)	3.0 mL **(30 equiv)**	$Cu(OAc)_2$	n.r.
6 ref. 3e	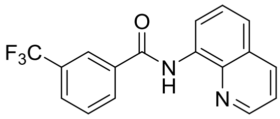 (0.5 mmol)	0.3 mL **(5.0 equiv)**	$Cu_2(OH)_2CO_3$	19%
7 ref. 3f	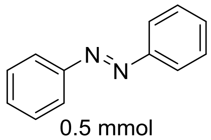 0.5 mmol	2.0 mL (**33 equiv**)	$Pd(OAc)_2$	n.r.[a]

8 *ref. 3g*	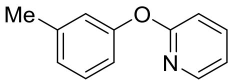 0.5 mmol	2.2 mL (**36 equiv**)	Pd(OAc)$_2$	n.r. [a]
9 *ref. 3h*	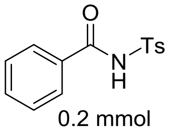 0.2 mmol	1.7 mL (**70 equiv**)	Pd(OAc)$_2$	n.r. [a]

The reactions were carried out according to the optimized reaction conditions of the literature methods, only by replacing the alcohol with the secondary alcohol **2a**. [a] **2a** was oxidized to acetophenone.

General procedure for nickellaelectro-catalyzed C–H alkoxylation

The electrolysis was carried out in an undivided cell, with a RVC anode (10 mm × 15 mm × 6 mm) and a nickel-foam cathode (10 mm × 15 mm × 1.4 mm). $Ni(DME)Cl_2$ (5.5 mg, 0.050 mmol, 10 mol %), 1-$AdCO_2H$ (9.0 mg, 0.05 mmol), NaO_2CAd (50.5 mg, 0.25 mmol), *n*Bu_4NClO_4 (0.50 mmol) and benzamide **1** (0.25 mmol, 1.0 equiv) were dissolved in DMA (3.0 mL) and then the alcohol **2** (2.5 mmol) was added. At 130 ℃, electrolysis was started with a constant current of 8.0 mA which was then maintained for 12 h under N_2. At ambient temperature, the mixture was transferred to a separating funnel and the electrodes were rinsed with EtOAc (10.0 mL). Then, the mixture was washed with H_2O (10.0 mL) and the organic layer was separated. The aqueous layer was extracted with EtOAc (2 × 5.0 mL). Evaporation of the combined organic layer and subsequent column chromatography on silica gel (eluent: *n*-hexane/EtOAc, *n*-hexane/aceton *or* CH_2Cl_2/aceton) yielded the desired product.

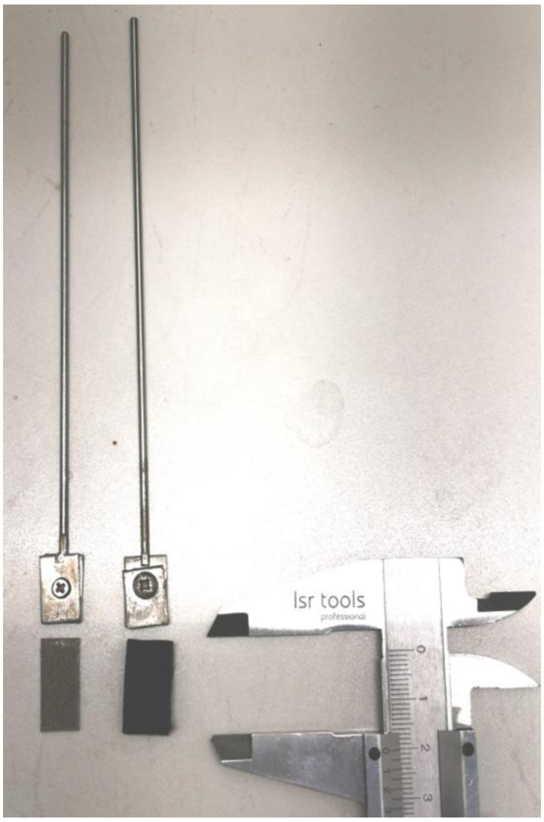

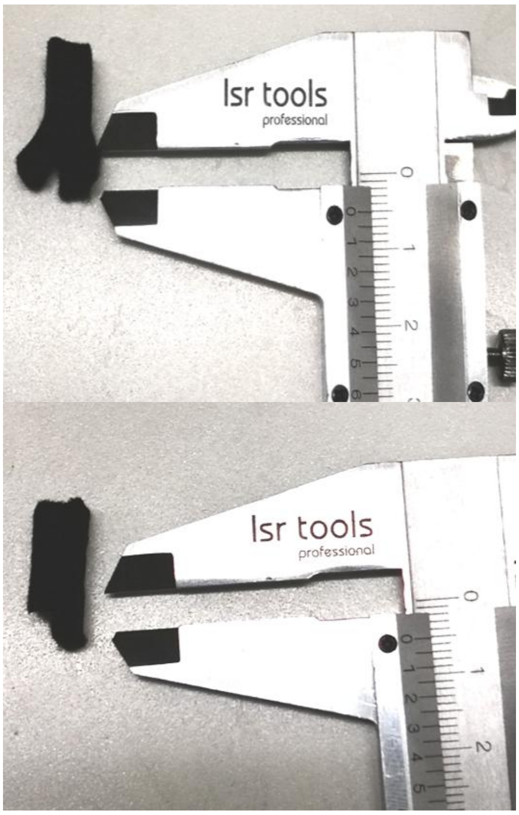

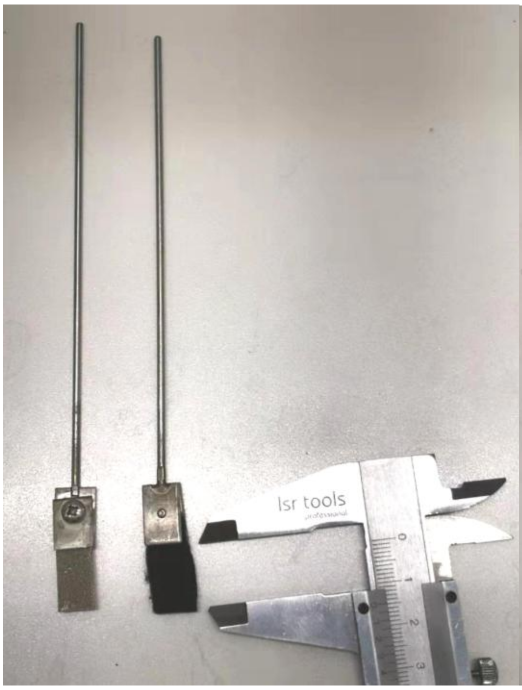
lsr tools
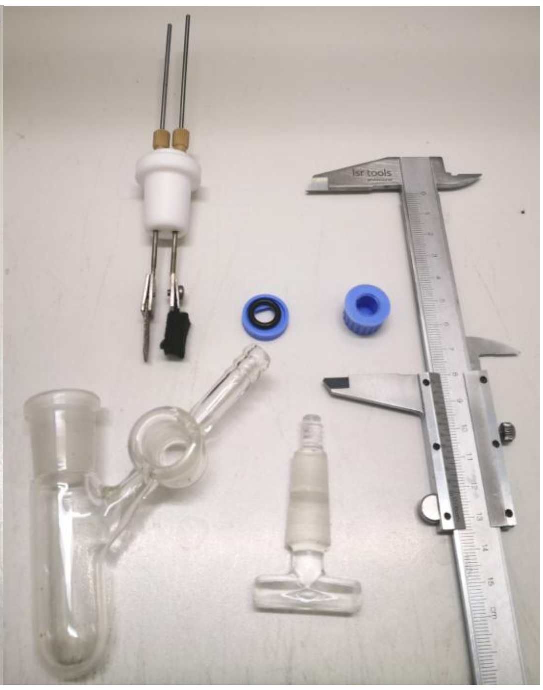
lsr tools
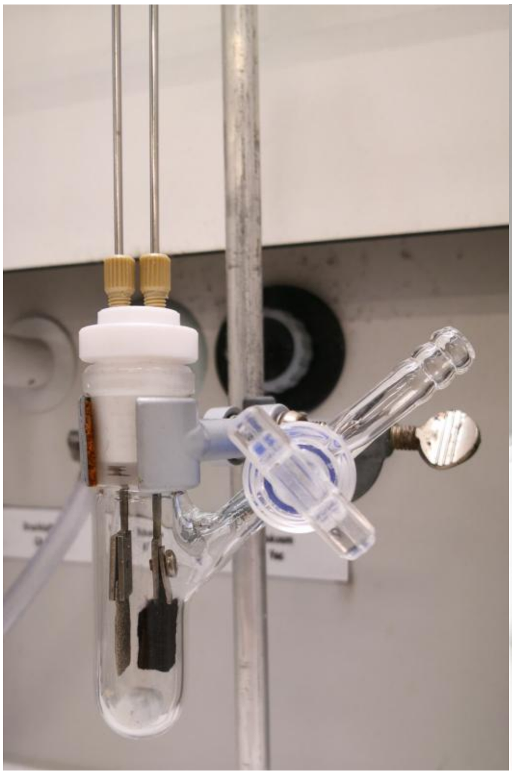
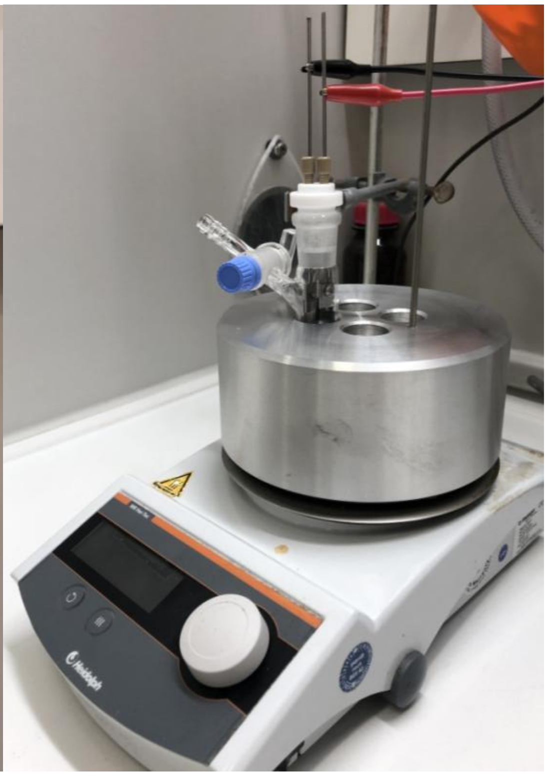

Characterization data for products

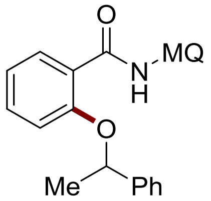

N-(6-Methylquinolin-8-yl)-2-(1-phenylethoxy)benzamide (3aa)

The general procedure was followed using benzamide **1a** (0.25 mmol, 65.5 mg) and 1-phenylethan-1-ol (**2a**) (305 mg, 2.5 mmol). Purification by column chromatography on silica gel (*n*-hexane/EtOAc: 16/1→9/1) yielded **3aa** (70.7 mg, 74%) as a white solid. **M.p.**: 126–127 ℃. **^{1}H NMR** (400 MHz, $CDCl_3$) δ = 12.12 (s, 1H), 9.01 (d, *J* = 1.8 Hz, 1H), 8.69 (dd, *J* = 4.2, 1.7 Hz, 1H), 8.29 (dd, *J* = 7.8, 1.9 Hz, 1H), 8.07 (dd, *J* = 8.2, 1.7 Hz, 1H), 7.62–7.56 (m, 2H), 7.38 (dd, *J* = 8.2, 4.2 Hz, 1H), 7.35–7.29 (m, 3H), 7.29–7.22 (m, 2H), 7.02 (ddd, *J* = 8.3, 7.3, 1.0 Hz, 1H), 6.91–6.88 (m, 1H), 5.61 (q, *J* = 6.5 Hz, 1H), 2.58 (d, *J* = 1.0 Hz, 3H), 1.94 (d, *J* = 6.5 Hz, 3H). **^{13}C NMR** (100 MHz, $CDCl_3$) δ = 164.1 (C_q), 156.0 (C_q), 146.8 (CH), 142.5 (C_q), 137.9 (C_q), 137.7 (C_q), 135.5 (CH), 135.4 (C_q), 132.7 (CH), 132.4 (CH), 128.8 (CH), 128.1 (C_q), 127.7 (CH), 125.6 (CH), 123.1 (C_q), 121.5 (CH), 121.0 (CH), 120.5 (CH), 119.9 (CH), 114.0 (CH), 77.9 (CH), 25.0 (CH_3), 22.4 (CH_3). **IR** (ATR): 3298, 2924, 1657, 1531, 1476, 1222, 855, 754, 696 cm^{-1}. **MS** (ESI) *m/z* (relative intensity): 405 (40) $[M+Na]^+$, 383 (100) $[M+H]^+$. **HR-MS** (ESI) *m/z* calcd for $C_{25}H_{23}N_2O_2$ $[M+H]^+$: 383.1754, found: 383.1754.

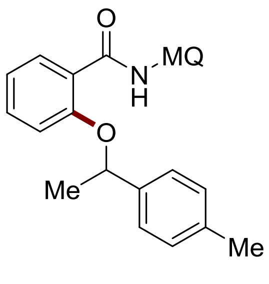

N-(6-Methylquinolin-8-yl)-2-[1-(*p*-tolyl)ethoxy]benzamide (3ab)

The general procedure was followed using benzamide **1a** (0.25 mmol, 65.5 mg) and 1-(*p*-tolyl)ethan-1-ol (**2b**) (341 mg, 2.5 mmol). Purification by column chromatography on silica gel (*n*-hexane/EtOAc: 16/1→9/1) yielded **3ab** (54.4 mg, 55%) as a white solid. **M.p.**: 128–130 ℃. **^{1}H NMR** (300 MHz, $CDCl_3$) δ = 12.18 (s, 1H), 9.06 (d, J = 1.8 Hz, 1H), 8.79–8.74 (m, 1H), 8.34 (dd, J = 7.9, 1.9 Hz, 1H), 8.11 (dd, J = 8.2, 1.7 Hz, 1H), 7.56–7.49 (m, 2H), 7.42 (dd, J = 8.2, 4.2 Hz, 1H), 7.39–7.29 (m, 2H), 7.17 (d, J = 7.9 Hz, 2H), 7.09–7.03 (m, 1H), 7.00–6.94 (m, 1H), 5.63 (q, J = 6.4 Hz, 1H), 2.63 (d, J = 0.9 Hz, 3H), 2.34 (s, 3H), 1.97 (d, J = 6.4 Hz, 3H). **^{13}C NMR** (75 MHz, $CDCl_3$) δ = 164.2 (C_q), 156.1 (C_q), 146.8 (CH), 139.5 (C_q), 138.0 (C_q), 137.7 (C_q), 137.5 (C_q), 135.5 (C_q),135.5 (CH), 132.8 (CH), 132.5 (CH), 129.5 (CH), 128.2 (C_q), 125.7 (CH), 123.1 (C_q), 121.5 (CH), 120.9 (CH), 120.5 (CH), 119.8 (CH), 114.1 (CH), 77.8 (CH), 25.1 (CH_3), 22.4 (CH_3), 21.2 (CH_3). **IR** (ATR): 3302, 1657, 1527, 1476, 1422, 1291, 1221, 1067, 753 cm^{-1}. **MS** (ESI) *m/z* (relative intensity): 419 (15) $[M+Na]^+$, 397 (100) $[M+H]^+$, 279 (90), 159 (50), 119 (100). **HR-MS** (ESI) *m/z* calcd for $C_{26}H_{25}N_2O_2$ $[M+H]^+$: 397.1911, found: 397.1913.

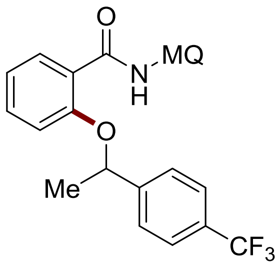

***N*-(6-Methylquinolin-8-yl)-2-{1-[4-(trifluoromethyl)phenyl]ethoxy}benzamide (3ac)**

The general procedure was followed using benzamide **1a** (0.25 mmol, 65.5 mg) and 1-[4-(trifluoromethyl)phenyl]ethan-1-ol (**2c**) (475 mg, 2.5 mmol). Purification by column chromatography on silica gel (*n*-hexane/EtOAc: 16/1→9/1) yielded **3ac** (73.6 mg, 65%) as a white solid. **M.p.**: 138–139 ℃. **^{1}H NMR** (400 MHz, $CDCl_3$) δ = 12.03 (s, 1H), 9.01 (d, J = 1.8 Hz, 1H), 8.63 (dd, J = 4.2, 1.7 Hz, 1H), 8.30 (dd, J = 7.8, 1.8 Hz, 1H), 8.07 (dd, J = 8.2, 1.7 Hz, 1H), 7.75–7.66 (m, 2H), 7.64–7.53 (m, 2H),

7.38 (dd, J = 8.2, 4.2 Hz, 1H), 7.33–7.27 (m, 2H), 7.06 (ddd, J = 8.2, 7.3, 1.0 Hz, 1H), 6.83 (dd, J = 8.5, 1.0 Hz, 1H), 5.66 (q, J = 6.5 Hz, 1H), 2.58 (d, J = 0.9 Hz, 3H), 1.93 (d, J = 6.5 Hz, 3H). **^{13}C NMR** (100 MHz, $CDCl_3$) δ = 163.9 (C_q), 155.5 (C_q), 146.7 (CH), 146.5 (C_q), 137.8 (C_q), 137.7 (C_q), 135.6 (CH), 135.2 (C_q), 132.8 (CH), 132.6 (CH), 130.0 (q, $^2J_{C\text{-}F}$ = 32.4 Hz, C_q), 128.1 (C_q), 126.0 (CH), 125.9 (q, $^3J_{C\text{-}F}$ = 3.8 Hz, CH), 123.9 (q, $^1J_{C\text{-}F}$ = 272.3 Hz, C_q), 123.3 (C_q), 121.5 (CH), 121.4 (CH), 120.6 (CH), 119.8 (CH), 113.7 (CH), 77.1 (CH), 24.7 (CH_3), 22.4 (CH_3). **^{19}F NMR** (282 MHz, $CDCl_3$) δ = -62.5 (s). **IR** (ATR): 3312, 2926, 1658, 1531, 1478, 1325, 1125, 1069, 753 cm^{-1}. **MS** (ESI) *m/z* (relative intensity): 473 (15) [M+Na]$^+$, 451 (100) [M+H]$^+$. **HR-MS** (ESI) *m/z* calcd for $C_{26}H_{22}F_3N_2O_2$ [M+H]$^+$: 451.1628, found: 451.1636.

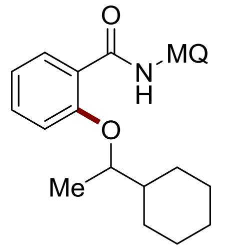

2-(1-Cyclohexylethoxy)-*N*-(6-methylquinolin-8-yl)benzamide (3ad)

The general procedure was followed using benzamide **1a** (0.25 mmol, 65.5 mg) and 1-cyclohexylethan-1-ol (**2d**) (320 mg, 2.5 mmol). Purification by column chromatography on silica gel (*n*-hexane/aceton: 16/1→9/1) yielded **3ad** (77.9 mg, 80%) as a colourless oil. **^{1}H NMR** (400 MHz, $CDCl_3$) δ = 11.95 (s, 1H), 8.98 (d, J = 1.9 Hz, 1H), 8.76 (dd, J = 4.2, 1.7 Hz, 1H), 8.32 (dd, J = 7.9, 1.9 Hz, 1H), 8.05 (dd, J = 8.2, 1.7 Hz, 1H), 7.45 (ddd, J = 8.4, 7.3, 1.9 Hz, 1H), 7.40 (dd, J = 8.2, 4.2 Hz, 1H), 7.28 (dq, J = 1.9, 1.0 Hz, 1H), 7.12–7.03 (m, 2H), 4.46 (p, J = 6.2 Hz, 1H), 2.56 (d, J = 1.0 Hz, 3H), 2.16–2.05 (m, 1H), 2.02 (ddt, J = 11.7, 3.7, 1.9 Hz, 1H), 1.92–1.84 (m, 1H), 1.77–1.68 (m, 1H), 1.69–1.56 (m, 2H), 1.47 (d, J = 6.2 Hz, 3H), 1.21–1.00 (m, 5H). **^{13}C NMR** (100 MHz, $CDCl_3$) δ = 164.3 (C_q), 156.7 (C_q), 146.7 (CH), 138.0 (C_q), 137.6 (C_q), 135.5 (C_q), 135.4 (CH), 132.9 (CH), 132.6 (CH), 128.1 (C_q), 123.5 (C_q), 121.4 (CH), 120.8 (CH), 120.5 (CH), 119.8 (CH), 113.8 (CH), 80.6

(CH), 42.3 (CH), 29.8 (CH_2), 28.1 (CH_2), 26.4 (CH_2), 26.1 (CH_2), 25.9 (CH_2), 22.3 (CH_3), 16.9 (CH_3). **IR** (ATR): 3295, 2923, 2851, 1656, 1521, 1475, 1420, 1286, 854, 752 cm^{-1}. **MS** (ESI) *m/z* (relative intensity): 411 (90) $[M+Na]^+$, 389 (100) $[M+H]^+$. **HR-MS** (ESI) *m/z* calcd for $C_{25}H_{29}N_2O_2$ $[M+H]^+$: 389.2224, found: 389.2217

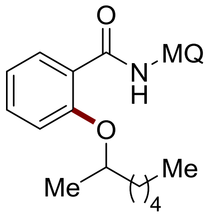

2-[(Heptan-2-yl)oxy]-*N*-(6-methylquinolin-8-yl)benzamide (3ae)

The general procedure was followed using benzamide **1a** (0.25 mmol, 65.5 mg) and heptan-2-ol (**2e**) (290 mg, 2.5 mmol). Purification by column chromatography on silica gel (*n*-hexane/EtOAc: 16/1→9/1) yielded **3ae** (75.0 mg, 80%) as a colourless oil. **^{1}H NMR** (400 MHz, $CDCl_3$) δ = 12.00 (s, 1H), 8.98 (d, J = 1.9 Hz, 1H), 8.74 (dd, J = 4.2, 1.7 Hz, 1H), 8.34 (dd, J = 7.8, 1.9 Hz, 1H), 8.05 (dd, J = 8.2, 1.7 Hz, 1H), 7.45 (ddd, J = 8.3, 7.2, 1.9 Hz, 1H), 7.39 (dd, J = 8.2, 4.2 Hz, 1H), 7.28 (dq, J = 1.9, 0.9 Hz, 1H), 7.12–7.04 (m, 2H), 4.71 (h, J = 6.2 Hz, 1H), 2.55 (d, J = 0.9 Hz, 3H), 2.17 (dddd, J = 13.6, 10.1, 6.5, 5.6 Hz, 1H), 1.79 (ddt, J = 13.6, 9.8, 6.0 Hz, 1H), 1.53 (d, J = 6.2 Hz, 3H), 1.51–1.44 (m, 1H), 1.44–1.35 (m, 1H), 1.31–1.15 (m, 4H), 0.78 (t, J = 7.0 Hz, 3H). **^{13}C NMR** (100 MHz, $CDCl_3$) δ = 164.2 (C_q), 156.5 (C_q), 146.7 (CH), 138.0 (C_q), 137.6 (C_q), 135.5 (C_q), 135.4 (CH), 132.9 (CH), 132.6 (CH), 128.1 (C_q), 123.4 (C_q), 121.4 (CH), 120.9 (CH), 120.4 (CH), 119.8 (CH), 113.8 (CH), 76.2 (CH), 36.0 (CH_2), 31.7 (CH_2), 25.4 (CH_2), 22.5 (CH_2), 22.3 (CH_3), 19.8 (CH_3), 13.9 (CH_3). **IR** (ATR): 3295, 2929, 1656, 1525, 1476, 1421, 1288, 752 cm^{-1}. **MS** (ESI) *m/z* (relative intensity): 399 (98) $[M+Na]^+$, 377 (100) $[M+H]^+$. **HR-MS** (ESI) *m/z* calcd for $C_{24}H_{29}N_2O_2$ $[M+H]^+$: 377.2224, found: 377.2225.

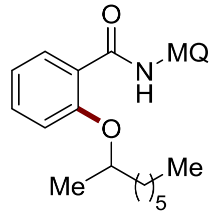

N-(6-Methylquinolin-8-yl)-2-[(octan-2-yl)oxy]benzamide (3af)

The general procedure was followed using benzamide **1a** (0.25 mmol, 65.5 mg) and octan-2-ol (**2f**) (325 mg, 2.5 mmol). Purification by column chromatography on silica gel (*n*-hexane/aceton: 16/1→9/1) yielded **3af** (89.6 mg, 92%) as a white solid. **M.p.**: 133–134 °C. **^{1}H NMR** (400 MHz, $CDCl_3$) δ = 12.00 (s, 1H), 8.97 (d, J = 1.9 Hz, 1H), 8.75 (dd, J = 4.2, 1.7 Hz, 1H), 8.33 (dd, J = 7.8, 1.9 Hz, 1H), 8.05 (dd, J = 8.2, 1.7 Hz, 1H), 7.45 (ddd, J = 8.2, 7.2, 1.9 Hz, 1H), 7.40 (dd, J = 8.2, 4.2 Hz, 1H), 7.28 (dq, J = 1.9, 1.0 Hz, 1H), 7.15–7.01 (m, 2H), 4.71 (h, J = 6.2 Hz, 1H), 2.56 (d, J = 1.0 Hz, 3H), 2.17 (dddd, J = 13.6, 10.1, 6.6, 5.5 Hz, 1H), 1.79 (ddt, J = 13.6, 9.9, 5.9 Hz, 1H), 1.53 (d, J = 6.2 Hz, 3H), 1.51–1.33 (m, 2H) 1.33–1.09 (m, 6H), 0.77 (t, J = 6.9 Hz, 3H). **^{13}C NMR** (100 MHz, $CDCl_3$) δ = 164.2 (C_q), 156.5 (C_q), 146.7 (CH), 138.0 (C_q), 137.7 (C_q), 135.5 (C_q), 135.4 (CH), 132.9 (CH), 132.6 (CH), 128.1 (C_q), 123.5 (C_q), 121.4 (CH), 120.9 (CH), 120.4 (CH), 119.8 (CH), 113.8 (CH), 76.3 (CH), 36.1 (CH_2), 31.7 (CH_2), 29.2 (CH_2), 25.7 (CH_2), 22.5 (CH_2), 22.4 (CH_3), 19.9 (CH_3), 14.0 (CH_3). **IR** (ATR): 3299, 2927, 1659, 1530, 1477, 753 cm^{-1}. **MS** (EI) *m/z* (relative intensity): 391 (10) $[M+H]^+$, 390 (30) $[M]^+$, 158 (100), 121 (50). **HR-MS** (EI) *m/z* calcd for $C_{25}H_{30}N_2O_2$ $[M]^+$: 390.2302, found: 390.2311.

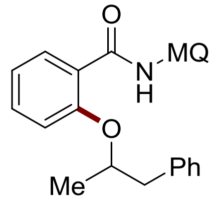

N-(6-Methylquinolin-8-yl)-2-[(1-phenylpropan-2-yl)oxy]benzamide (3ag)

The general procedure was followed using benzamide **1a** (0.25 mmol, 65.5 mg) and 1-phenylpropan-2-ol (**2g**) (340 mg, 2.5 mmol). Purification by column

chromatography on silica gel (*n*-hexane/EtOAc: 16/1→9/1) yielded **3ag** (84.7 mg, 86%) as a colourless oil. **^{1}H NMR** (400 MHz, $CDCl_3$) δ = 12.07 (s, 1H), 9.01 (d, *J* = 1.9 Hz, 1H), 8.76 (dd, *J* = 4.2, 1.7 Hz, 1H), 8.35 (dd, *J* = 7.9, 1.9 Hz, 1H), 8.06 (dd, *J* = 8.2, 1.7 Hz, 1H), 7.43 (ddd, *J* = 8.3, 7.2, 1.9 Hz, 1H), 7.40 (dd, *J* = 8.2, 4.2 Hz, 1H), 7.30 (dq, *J* = 1.9, 1.0 Hz, 1H), 7.28–7.25 (m, 2H), 7.25–7.20 (m, 2H), 7.18–7.13 (m, 1H), 7.09 (ddd, *J* = 8.1, 7.2, 1.0 Hz, 1H), 7.02 (dd, *J* = 8.3, 0.8 Hz, 1H), 4.99–4.85 (m, 1H), 3.59 (dd, *J* = 13.6, 6.3 Hz, 1H), 3.08 (dd, *J* = 13.6, 6.7 Hz, 1H), 2.58 (d, *J* = 1.0 Hz, 3H), 1.55 (d, *J* = 6.1 Hz, 3H). **^{13}C NMR** (100 MHz, $CDCl_3$) δ = 164.0 (C_q), 156.0 (C_q), 146.8 (CH), 138.1 (C_q), 137.9 (C_q), 137.7 (C_q), 135.5 (CH), 135.4 (C_q), 132.9 (CH), 132.6 (CH), 129.3 (CH), 128.4 (CH), 128.1 (C_q), 126.4 (CH), 123.6 (C_q), 121.4 (CH), 121.2 (CH), 120.5 (CH), 119.8 (CH), 113.9 (CH), 77.3 (CH), 42.4 (CH_2), 22.3 (CH_3), 19.5 (CH_3). **IR** (ATR): 3230, 2925, 1655, 1524, 1476, 1422, 1288, 750, 698 cm^{-1}. **MS** (EI) *m/z* (relative intensity): 397 (5) $[M+H]^+$, 396 (15) $[M]^+$, 158 (100), 120 (50), 91 (50). **HR-MS** (EI) *m/z* calcd for $C_{26}H_{24}N_2O_2$ $[M]^+$: 396.1832, found: 396.1848.

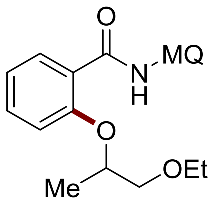

2-[(1-Ethoxypropan-2-yl)oxy]-*N*-(6-methylquinolin-8-yl)benzamide (3ah)

The general procedure was followed using benzamide **1a** (0.25 mmol, 65.5 mg) and 1-ethoxypropan-2-ol (**2h**) (260 mg, 2.5 mmol). Purification by column chromatography on silica gel (*n*-hexane/EtOAc: 16/1→9/1) yielded **3ah** (48.5 mg, 54%) as a colourless oil. **^{1}H NMR** (400 MHz, $CDCl_3$) δ = 11.95 (s, 1H), 8.96 (d, *J* = 1.9 Hz, 1H), 8.77 (dd, *J* = 4.2, 1.7 Hz, 1H), 8.32 (dd, *J* = 7.9, 1.9 Hz, 1H), 8.06 (dd, *J* = 8.2, 1.7 Hz, 1H), 7.46 (ddd, *J* = 8.4, 7.2, 1.9 Hz, 1H), 7.41 (dd, *J* = 8.2, 4.2 Hz, 1H), 7.28 (dq, *J* = 1.9, 1.0 Hz, 1H), 7.16 (ddd, *J* = 8.4, 1.0, 0.5 Hz, 1H), 7.11 (ddd, *J* = 7.9, 7.2, 1.0 Hz, 1H), 4.89 (tdd, *J* = 6.1, 5.4, 0.7 Hz, 1H), 4.03 (dd, *J* = 10.1, 6.1 Hz, 1H), 3.71 (dd, *J* = 10.1, 5.4 Hz, 1H), 3.49 (q, *J* = 7.0 Hz, 2H), 2.55 (d, *J* = 1.0 Hz, 3H),

1.56 (d, J = 6.3 Hz, 3H), 1.09 (t, J = 7.0 Hz, 3H). **^{13}C NMR** (100 MHz, $CDCl_3$) δ = 164.0 (C_q), 156.3 (C_q), 146.7 (CH), 138.0 (C_q), 137.7 (C_q), 135.6 (CH), 135.4 (C_q), 132.9 (CH), 132.5 (CH), 128.1 (C_q), 123.7 (C_q), 121.4 (CH), 121.4 (CH), 120.5 (CH), 119.8 (CH), 114.4 (CH), 75.5 (CH), 73.6 (CH_2), 66.9 (CH_2), 22.3 (CH_3), 17.4 (CH_3), 15.1 (CH_3). **IR** (ATR): 3300, 2976, 1655, 1523, 1475, 1219, 1109, 854, 751, 692 cm^{-1}. **MS** (ESI) *m/z* (relative intensity): 387 (30) $[M+Na]^+$, 365 (100) $[M+H]^+$. **HR-MS** (ESI) *m/z* calcd for $C_{22}H_{25}N_2O_3$ $[M+H]^+$: 365.1860, found: 365.1861.

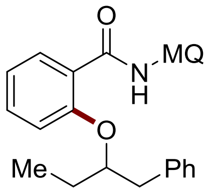

***N*-(6-Methylquinolin-8-yl)-2-[(1-phenylbutan-2-yl)oxy]benzamide (3ai)**

The general procedure was followed using benzamide **1a** (0.25 mmol, 65.5 mg) and 1-phenylbutan-2-ol (**2i**) (375 mg, 2.5 mmol). Purification by column chromatography on silica gel (*n*-hexane/aceton: 30/1→15/1) yielded **3ai** (58.5 mg, 57%) as a colourless oil. **^{1}H NMR** (400 MHz, $CDCl_3$) δ = 12.12 (s, 1H), 9.03 (d, J = 1.9 Hz, 1H), 8.74 (dd, J = 4.2, 1.7 Hz, 1H), 8.36 (dd, J = 7.9, 1.9 Hz, 1H), 8.06 (dd, J = 8.3, 1.7 Hz, 1H), 7.44–7.38 (m, 2H), 7.30 (dq, J = 1.9, 1.0 Hz, 1H), 7.28–7.23 (m, 2H), 7.22–7.17 (m, 2H), 7.15–7.10 (m, 1H), 7.08 (ddd, J = 7.9, 7.2, 1.0 Hz, 1H), 6.99 (d, J = 8.4, 1H), 4.85–4.72 (m, 1H), 3.50 (dd, J = 13.8, 6.8 Hz, 1H), 3.11 (dd, J = 13.8, 5.8 Hz, 1H), 2.58 (d, J = 1.0 Hz, 3H), 2.10 (ddd, J = 14.2, 7.5, 6.8 Hz, 1H), 1.98–1.86 (m, 1H), 1.04 (t, J = 7.5 Hz, 3H). **^{13}C NMR** (100 MHz, $CDCl_3$) δ = 164.0 (C_q), 156.7 (C_q), 146.6 (CH), 138.1 (C_q), 138.0 (C_q), 137.6 (C_q), 135.5 (C_q), 135.5 (CH), 132.9 (CH), 132.7 (CH), 129.3 (CH), 128.4 (CH), 128.1 (C_q), 126.3 (CH), 123.3 (C_q), 121.4 (CH), 121.0 (CH), 120.5 (CH), 119.8 (CH), 113.7 (CH), 82.5 (CH), 40.0 (CH_2), 26.4 (CH_2), 22.3 (CH_3), 9.9 (CH_3). **IR** (ATR): 3295, 2968, 1654, 1521, 1475, 1419, 1219, 854, 747, 692 cm^{-1}. **MS** (ESI) *m/z* (relative intensity): 433 (50) $[M+Na]^+$, 411 (100) $[M+H]^+$. **HR-MS** (ESI) *m/z* calcd for $C_{27}H_{27}N_2O_2$ $[M+H]^+$: 411.2067, found: 411.2067.

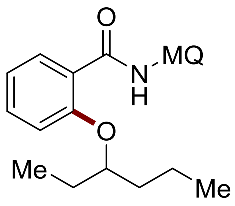

2-[(Hexan-3-yl)oxy]-*N*-(6-methylquinolin-8-yl)benzamide (3aj)

The general procedure was followed using benzamide **1a** (0.25 mmol, 65.5 mg) and hexan-3-ol (**2j**) (255 mg, 2.5 mmol). Purification by column chromatography on silica gel (*n*-hexane/EtOAc: 20/1→9/1) yielded **3aj** (45.5 mg, 50%) as a colourless oil. **^{1}H NMR** (400 MHz, $CDCl_3$) δ = 12.05 (s, 1H), 8.99 (d, J = 1.9 Hz, 1H), 8.74 (dd, J = 4.2, 1.7 Hz, 1H), 8.47–8.26 (m, 1H), 8.05 (dd, J = 8.2, 1.7 Hz, 1H), 7.49–7.42 (m, 1H), 7.40 (dd, J = 8.2, 4.2 Hz, 1H), 7.28 (dd, J = 1.9, 1.0 Hz, 1H), 7.12–7.05 (m, 2H), 4.65–4.48 (m, 1H), 2.56 (d, J = 1.0 Hz, 3H), 2.19–2.00 (m, 2H), 1.96–1.73 (m, 2H), 1.63–1.38 (m, 2H), 1.01 (t, J = 7.4 Hz, 3H), 0.87 (t, J = 7.4 Hz, 3H). **^{13}C NMR** (100 MHz, $CDCl_3$) δ = 164.2 (C_q), 157.1 (C_q), 146.6 (CH), 138.1 (C_q), 137.6 (C_q), 135.6 (C_q), 135.4 (CH), 132.9 (CH), 132.7 (CH), 128.1 (C_q), 123.1 (C_q), 121.4 (CH), 120.7 (CH), 120.5 (CH), 119.9 (CH), 113.6 (CH), 81.2 (CH), 35.6 (CH_2), 26.7 (CH_2), 22.4 (CH_3), 18.8 (CH_2), 14.1 (CH_3), 10.0 (CH_3). **IR** (ATR): 3293, 2960, 1654, 1522, 1475, 1287, 949, 854, 752, 692 cm^{-1}. **MS** (ESI) *m/z* (relative intensity): 385 (30) $[M+Na]^+$, 363 (100) $[M+H]^+$. **HR-MS** (ESI) *m/z* calcd for $C_{23}H_{27}N_2O_2$ $[M+H]^+$: 363.2067, found: 363.2068.

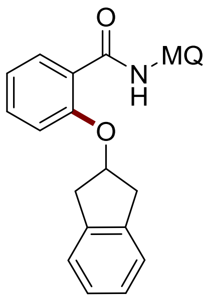

2-[(2,3-Dihydro-*1H*-inden-2-yl)oxy)-*N*-(6-methylquinolin-8-yl)benzamide (3ak)

The general procedure was followed using benzamide **1a** (0.25 mmol, 65.5 mg) and 2,3-dihydro-*1H*-inden-2-ol (**2k**) (335 mg, 2.5 mmol). Purification by column

chromatography on silica gel (*n*-hexane/aceton: 16/1→9/1) yielded **3ak** (70.7 mg, 72%) as a white solid. **M.p.**: 157–158 ℃. **^{1}H NMR** (300 MHz, $CDCl_3$) δ = 11.77 (s, 1H), 8.91 (d, *J* = 1.9 Hz, 1H), 8.41 (dd, *J* = 4.2, 1.7 Hz, 1H), 8.34 (dd, *J* = 8.0, 1.9 Hz, 1H), 8.00 (dd, *J* = 8.2, 1.7 Hz, 1H), 7.54–7.46 (m, 1H), 7.31 (dd, *J* = 8.2, 4.2 Hz, 1H), 7.26–7.23 (m, 1H), 7.18–7.09 (m, 6H), 5.59–5.39 (m, 1H), 3.73 (dd, *J* = 16.7, 4.6 Hz, 2H), 3.54 (dd, *J* = 16.7, 7.2 Hz, 2H), 2.53 (d, *J* = 0.9 Hz, 3H). **^{13}C NMR** (126 MHz, $CDCl_3$) δ = 163.7 (C_q), 155.8 (C_q), 146.6 (CH), 140.1 (C_q), 137.7 (C_q), 137.4 (C_q), 135.3 (CH), 135.1 (C_q), 132.8 (CH), 132.7 (CH), 127.9 (C_q), 126.6 (CH), 124.5 (CH), 123.4 (C_q), 121.3 (CH), 121.2 (CH), 120.4 (CH), 119.6 (CH), 113.5 (CH), 79.4 (CH), 39.6 (CH_2), 22.4 (CH_3). **IR** (ATR): 3300, 1651, 1531, 1475, 1290, 1028, 860, 744, 684, 591 cm^{-1}. **MS** (ESI) *m/z* (relative intensity): 417 (30) $[M+Na]^+$, 395 (100) $[M+H]^+$. **HR-MS** (ESI) *m/z* calcd for $C_{26}H_{23}N_2O_2$ $[M+H]^+$: 395.1754, found: 395.1755.

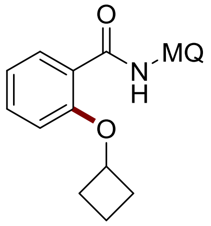

2-Cyclobutoxy-*N*-(6-methylquinolin-8-yl)benzamide (3al)

The general procedure was followed using benzamide **1a** (0.25 mmol, 65.5 mg) and cyclobutanol (**2l**) (188 mg, 2.5 mmol). Purification by column chromatography on silica gel (*n*-hexane/aceton: 16/1→9/1) yielded **3al** (61.8 mg, 74%) as a white solid. **M.p.**: 86–87 ℃. **^{1}H NMR** (300 MHz, $CDCl_3$) δ = 12.01 (s, 1H), 8.98 (d, *J* = 1.9 Hz, 1H), 8.74 (dd, *J* = 4.2, 1.7 Hz, 1H), 8.35 (dd, *J* = 7.9, 1.8 Hz, 1H), 8.04 (dd, *J* = 8.3, 1.7 Hz, 1H), 7.43 (ddd, *J* = 8.4, 7.2, 1.8 Hz, 1H), 7.38 (dd, *J* = 8.3, 4.2 Hz, 1H), 7.28 (dq, *J* = 1.9, 1.0 Hz, 1H), 7.09 (ddd, *J* = 7.9, 7.2, 1.0 Hz, 1H), 6.88 (dd, *J* = 8.4, 1.0 Hz, 1H), 4.94 (pd, *J* = 7.2, 1.0 Hz, 1H), 2.76–2.57 (m, 4H), 2.55 (d, *J* = 1.0 Hz, 3H), 2.11–1.91 (m, 1H), 1.85–1.72 (m, 1H). **^{13}C NMR** (126 MHz, $CDCl_3$) δ = 163.8 (C_q), 155.7 (C_q), 146.7 (CH), 137.9 (C_q), 137.5 (C_q), 135.5 (C_q), 135.4 (CH), 132.9

(CH), 132.5 (CH), 128.0 (C_q), 122.3 (C_q), 121.3 (CH), 120.9 (CH), 120.4 (CH), 119.8 (CH), 112.9 (CH), 72.9 (CH), 30.7 (CH_2), 22.4 (CH_3), 13.6 (CH_2). **IR** (ATR): 3298, 2937, 1654, 1524, 1476, 1290, 1219, 854, 750, 689 cm^{-1}. **MS** (ESI) *m/z* (relative intensity): 355 (80) $[M+Na]^+$, 333 (100) $[M+H]^+$. **HR-MS** (ESI) *m/z* calcd for $C_{21}H_{21}N_2O_2$ $[M+H]^+$: 333.1598, found: 333.1601.

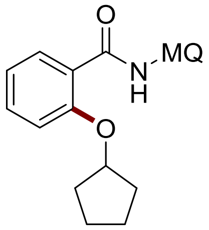

2-(Cyclopentyloxy)-*N*-(6-methylquinolin-8-yl)benzamide (3am)

The general procedure was followed using benzamide **1a** (0.25 mmol, 65.5 mg) and cyclopentanol (**2m**) (215 mg, 2.5 mmol). Purification by column chromatography on silica gel (*n*-hexane/aceton: 16/1→9/1) yielded **3am** (68.4 mg, 79%) as a white solid. **M.p.**: 96–98 ℃. **^{1}H NMR** (300 MHz, $CDCl_3$) δ = 11.80 (s, 1H), 9.03 (d, J = 1.9 Hz, 1H), 8.77 (dd, J = 4.2, 1.7 Hz, 1H), 8.36 (dd, J = 7.8, 1.9 Hz, 1H), 8.09 (dd, J = 8.2, 1.7 Hz, 1H), 7.49 (ddd, J = 8.2, 7.3, 1.9 Hz, 1H), 7.43 (dd, J = 8.2, 4.2 Hz, 1H), 7.33 (dq, J = 1.9, 1.0 Hz, 1H), 7.16–7.06 (m, 2H), 5.13 (tt, J = 6.1, 2.9 Hz, 1H), 2.60 (d, J = 1.0 Hz, 3H), 2.45–2.33 (m, 2H), 2.22–2.02 (m, 2H), 1.98–1.80 (m, 2H), 1.67 (tdd, J = 10.9, 8.2, 5.2 Hz, 2H). **^{13}C NMR** (100 MHz, $CDCl_3$) δ = 164.3 (C_q), 156.3 (C_q), 146.6 (CH), 138.0 (C_q), 137.6 (C_q), 135.5 (CH), 135.4 (C_q), 132.8 (CH), 132.7 (CH), 128.1 (C_q), 123.1 (C_q), 121.4 (CH), 120.6 (CH), 120.5 (CH), 120.0 (CH), 113.6 (CH), 81.2 (CH), 32.9 (CH_2), 24.4 (CH_2), 22.3 (CH_3). **IR** (ATR): 3305, 2960, 1656, 1528, 1478, 1421, 1289, 753 cm^{-1}. **MS** (ESI) *m/z* (relative intensity): 369 (100) $[M+Na]^+$, 347 (90) $[M+H]^+$. **HR-MS** (ESI) *m/z* calcd for $C_{22}H_{23}N_2O_2$ $[M+H]^+$: 347.1754, found: 347.1756.

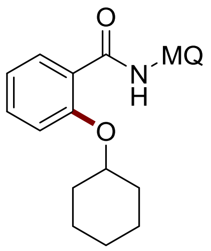

2-(Cyclohexyloxy)-*N*-(6-methylquinolin-8-yl)benzamide (3an)

The general procedure was followed using benzamide **1a** (0.25 mmol, 65.5 mg) and cyclohexanol (**2n**) (251 mg, 2.5 mmol). Purification by column chromatography on silica gel (*n*-hexane/aceton: 20/1) yielded **3an** (68.4 mg, 76%) as a white solid. **M.p.**: 121–123 °C. **^{1}H NMR** (300 MHz, $CDCl_3$) δ = 11.97 (s, 1H), 9.01 (d, *J* = 1.9 Hz, 1H), 8.78 (dd, *J* = 4.2, 1.7 Hz, 1H), 8.34 (dd, *J* = 8.1, 1.9 Hz, 1H), 8.09 (dd, *J* = 8.2, 1.7 Hz, 1H), 7.48 (ddd, *J* = 8.4, 7.3, 1.9 Hz, 1H), 7.44 (dd, *J* = 8.2, 4.2 Hz, 1H), 7.32 (dq, *J* = 1.9, 1.0 Hz, 1H), 7.16–7.06 (m, 2H), 4.59 (tt, *J* = 9.8, 4.0 Hz, 1H), 2.60 (d, *J* = 1.0 Hz, 3H), 2.29–2.20 (m, 2H), 2.03–1.92 (m, 2H), 1.88 (dt, *J* = 7.3, 3.3 Hz, 2H), 1.74–1.65 (m, 1H), 1.51–1.26 (m, 3H). **^{13}C NMR** (100 MHz, $CDCl_3$) δ = 164.3 (C_q), 156.0 (C_q), 146.7 (CH), 137.9 (C_q), 137.6 (C_q), 135.4 (CH), 135.4 (C_q), 132.7 (CH), 132.5 (CH), 128.1 (C_q), 123.6 (C_q), 121.4 (CH), 120.9 (CH), 120.4 (CH), 119.8 (CH), 113.9 (CH), 77.9 (CH), 31.9 (CH_2), 25.6 (CH_2), 24.4 (CH_2), 22.3 (CH_3). **IR** (ATR): 3293, 2936, 2857, 1656, 1527, 1476, 1289, 1221, 753 cm^{-1}. **MS** (ESI) *m/z* (relative intensity): 383 (25) $[M+Na]^+$, 361 (100) $[M+H]^+$. **HR-MS** (ESI) *m/z* calcd for $C_{23}H_{25}N_2O_2$ $[M+H]^+$: 361.1911, found: 361.1913.

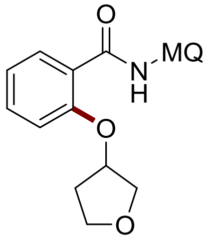

***N*-(6-Methylquinolin-8-yl)-2-[(tetrahydrofuran-3-yl)oxy]benzamide (3ao)**

The general procedure was followed using benzamide **1a** (0.25 mmol, 65.5 mg) and tetrahydrofuran-3-ol (**2o**) (220 mg, 2.5 mmol). Purification by column

chromatography on silica gel (*n*-hexane/EtOAc: 16/1→9/1) yielded **3ao** (70.2 mg, 81%) as a white solid. **M.p.**: 113–114 °C. **^{1}H NMR** (400 MHz, $CDCl_3$) δ = 11.76 (s, 1H), 8.96 (d, *J* = 1.9 Hz, 1H), 8.79 (dd, *J* = 4.2, 1.7 Hz, 1H), 8.36 (dd, *J* = 7.9, 1.9 Hz, 1H), 8.04 (dd, *J* = 8.2, 1.7 Hz, 1H), 7.47 (ddd, *J* = 8.4, 7.3, 1.9 Hz, 1H), 7.39 (dd, *J* = 8.2, 4.2 Hz, 1H), 7.28 (dq, *J* = 1.9, 1.0 Hz, 1H), 7.13 (ddd, *J* = 7.9, 7.3, 1.0 Hz, 1H), 6.98 (dd, *J* = 8.4, 1.0 Hz, 1H), 5.26–5.17 (m, 1H), 4.54 (dd, *J* = 10.3, 2.3 Hz, 1H), 4.21 (dd, *J* = 10.3, 5.4 Hz, 1H), 4.09 (td, *J* = 8.8, 6.3 Hz, 1H), 3.96 (td, *J* = 8.2, 3.6 Hz, 1H), 2.55 (d, *J* = 1.0 Hz, 3H), 2.59–3.53 (m, 1H), 2.32 (dddd, *J* = 13.4, 9.0, 8.0, 6.4 Hz, 1H). **^{13}C NMR** (100 MHz, $CDCl_3$) δ = 163.7 (C_q), 155.5 (C_q), 146.9 (CH), 137.9 (C_q), 137.6 (C_q), 135.5 (CH), 135.2 (C_q), 133.1 (CH), 132.9 (CH), 128.1 (C_q), 123.1 (C_q), 121.6 (CH), 121.4 (CH), 120.6 (CH), 119.9 (CH), 112.9 (CH), 79.1 (CH), 72.9 (CH_2), 67.3 (CH_2), 33.2 (CH_2), 22.3 (CH_3). **IR** (ATR): 3307, 1653, 1525, 1477, 1421, 1290, 1220, 855, 752, 688 cm^{-1}. **MS** (ESI) *m/z* (relative intensity): 371 (70) $[M+Na]^+$, 349 (100) $[M+H]^+$. **HR-MS** (ESI) *m/z* calcd for $C_{21}H_{21}N_2O_3$ $[M+H]^+$: 349.1547, found: 349.1550.

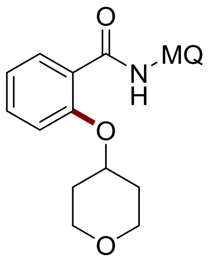

***N*-(6-Methylquinolin-8-yl)-2-[(tetrahydro-*2H*-pyran-4-yl)oxy]benzamide (3ap)**

The general procedure was followed using benzamide **1a** (0.25 mmol, 65.5 mg) and tetrahydro-*2H*-pyran-4-ol (**2p**) (255 mg, 2.5 mmol). Purification by column chromatography on silica gel (*n*-hexane/EtOAc: 4/1→2/1) yielded **3ap** (73.4 mg, 81%) as a white solid. **M.p.**: 145–147 °C. **^{1}H NMR** (400 MHz, $CDCl_3$) δ = 11.81 (s, 1H), 8.94 (d, *J* = 1.7 Hz, 1H), 8.76 (dd, *J* = 4.3, 1.6 Hz, 1H), 8.28 (dd, *J* = 7.8, 1.9 Hz, 1H), 8.05 (dd, *J* = 8.3, 1.6 Hz, 1H), 7.45 (ddd, *J* = 8.9, 7.5, 1.9 Hz, 1H), 7.40 (dd, *J* = 8.3, 4.3 Hz, 1H), 7.30–7.27 (m, 1H), 7.14–7.05 (m, 2H), 4.79 (tt, *J* = 8.9, 4.3 Hz, 1H),

4.03 (dt, *J* = 11.9, 4.3 Hz, 2H), 3.52 (ddd, *J* = 12.1, 9.9, 2.8 Hz, 2H), 2.55 (s, 3H), 2.27 (dtd, *J* = 13.5, 9.3, 4.3 Hz, 2H), 2.18–2.09 (m, 2H). **^{13}C NMR** (100 MHz, CDCl$_3$) δ = 164.0 (C$_q$), 155.2 (C$_q$), 146.9 (CH), 137.8 (C$_q$), 137.7 (C$_q$), 135.5 (CH), 135.1 (C$_q$), 132.8 (CH), 132.7 (CH), 128.1 (C$_q$), 124.0 (C$_q$), 121.6 (CH), 121.4 (CH), 120.6 (CH), 119.7 (CH), 113.8 (CH), 74.0 (CH), 65.7 (CH$_2$), 31.8 (CH$_2$), 22.4 (CH$_3$). **IR** (ATR): 3295, 2960, 1649, 1524, 1477, 980, 857, 753, 590 cm^{-1}. **MS** (EI) *m/z* (relative intensity): 363 (5) [M+H]$^+$, 362 (15) [M]$^+$, 158 (100), 121 (50). **HR-MS** (EI) *m/z* calcd for $C_{22}H_{22}N_2O_3$ [M]$^+$: 362.1625, found: 362.1632.

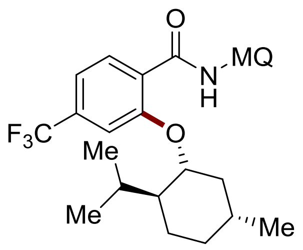

2-{[(*1R,2S,5R*)-2-Isopropyl-5-methylcyclohexyl]oxy}-*N*-(quinolin-8-yl)-4-(trifluoromethyl)benzamide (3kq)

The general procedure was followed using benzamide **1k** (0.25 mmol, 82.5 mg) and (*1S,2R,5S*)-menthol (**2q**) (391 mg, 2.5 mmol). Purification by column chromatography on silica gel (*n*-hexane/aceton: 30/1) yielded **3kq** (64.0 mg, 53%) as a white solid. **M.p.**: 87–89 °C. **^{1}H NMR** (300 MHz, CDCl$_3$) δ = 12.07 (s, 1H), 9.00 (d, *J* = 1.7 Hz, 1H), 8.81 (dd, *J* = 4.2, 1.6 Hz, 1H), 8.50 (d, *J* = 8.1 Hz, 1H), 8.12 (dd, *J* = 8.3, 1.6 Hz, 1H), 7.47 (dd, *J* = 8.3, 4.2 Hz, 1H), 7.40–7.35 (m, 3H), 4.50 (td, *J* = 10.2, 4.3 Hz, 1H), 2.61 (s, 3H), 2.36–2.11 (m, 3H), 1.86 (tt, *J* = 10.2, 3.0 Hz, 2H), 1.64 (dt, *J* = 9.0, 5.6 Hz, 2H), 1.34–1.05 (m, 2H), 1.01 (d, *J* = 5.8 Hz, 3H), 0.77 (dd, *J* = 8.6, 6.9 Hz, 6H). **^{13}C NMR** (100 MHz, CDCl$_3$) δ = 162.8 (C$_q$), 156.6 (C$_q$), 146.9 (CH), 138.0 (C$_q$), 137.7 (C$_q$), 135.6 (CH), 135.1 (C$_q$), 134.5 (q, $^2J_{C\text{-}F}$ = 32.4 Hz, C$_q$), 133.5 (CH), 128.1 (C$_q$), 126.3 (C$_q$), 123.6 (q, $^1J_{C\text{-}F}$ = 272.9 Hz, C$_q$), 121.6 (CH), 120.9 (CH), 120.0 (CH), 117.4 (q, $^3J_{C\text{-}F}$ = 3.8 Hz, CH), 110.1 (q, $^3J_{C\text{-}F}$ = 3.8 Hz, CH), 80.3 (CH), 47.0 (CH), 40.1 (CH$_2$), 34.6 (CH$_2$), 31.5 (CH), 26.1 (CH), 23.6 (CH$_2$), 22.3 (CH$_3$), 22.1 (CH$_3$), 20.6 (CH$_3$), 16.5 (CH$_3$). **^{19}F NMR** (376 MHz, CDCl$_3$) δ = -62.9 (s). **IR**

(ATR): 3300, 2956, 2927, 1665, 1532, 1325, 1127, 984, 855, 696 cm^{-1}. **MS** (ESI) *m/z* (relative intensity): 507 (8) $[M+Na]^+$, 485 (100) $[M+H]^+$, 347 (10). **HR-MS** (ESI) *m/z* calcd for $C_{28}H_{32}F_3N_2O_2$ $[M+H]^+$: 485.2410, found: 485.2401.

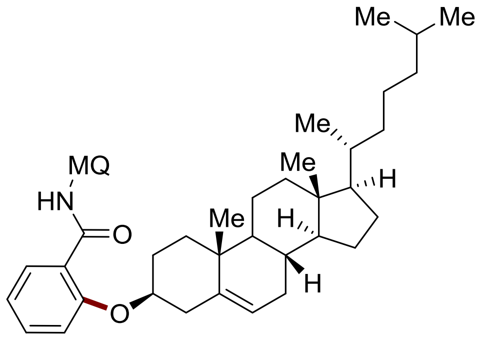

2-{[(*3S,8S,10R,13R,14S,17R*)-10,13-Dimethyl-17-[(*R*)-6-methylheptan-2-yl]-2,3,4,7,8,9,10,11,12,13,14,15,16,17-tetradecahydro-*1H*-cyclopenta[a]phenanthren-3-yl]-oxy}-*N*-(6-methylquinolin-8-yl)benzamide (3ar)

The general procedure was followed using benzamide **1a** (0.25 mmol, 65.5 mg) and cholesterol (**2r**) (966 mg, 2.5 mmol). Purification by column chromatography on silica gel (*n*-hexane/EtOAc: 20/1→9/1) yielded **3ar** (98.6 mg, 61%) as a white solid. **M.p.**: 227–229 ℃. **^{1}H NMR** (400 MHz, $CDCl_3$) δ = 11.93 (s, 1H), 8.99 (d, *J* = 1.8 Hz, 1H), 8.75 (dd, *J* = 4.2, 1.7 Hz, 1H), 8.33 (dd, *J* = 7.8, 1.9 Hz, 1H), 8.05 (dd, *J* = 8.2, 1.7 Hz, 1H), 7.44 (ddd, *J* = 8.3, 7.3, 1.9 Hz, 1H), 7.39 (dd, *J* = 8.2, 4.2 Hz, 1H), 7.28 (dq, *J* = 1.8, 1.0 Hz, 1H), 7.11–7.04 (m, 2H), 5.43 (dt, *J* = 5.5, 1.9 Hz, 1H), 4.44 (tt, *J* = 11.2, 4.6 Hz, 1H), 2.90 (tq, *J* = 11.4, 2.7 Hz, 1H), 2.70–2.64 (m, 1H), 2.55 (d, *J* = 1.0 Hz, 3H), 2.19 (dt, *J* = 11.0, 3.6 Hz, 1H), 2.11 (ddd, *J* = 14.4, 11.0, 3.8 Hz, 1H), 2.06–1.91 (m, 3H), 1.91–1.77 (m, 1H), 1.64–1.41 (m, 8H), 1.45–1.21 (m, 4H), 1.22–1.07 (m, 5H), 1.06 (s, 3H), 1.07–0.91 (m, 3H), 0.91 (d, *J* = 6.5 Hz, 3H), 0.85 (dd, *J* = 6.6, 1.8 Hz, 6H), 0.67 (s, 3H). **^{13}C NMR** (100 MHz, $CDCl_3$) δ = 164.2 (C_q), 156.1 (C_q), 146.8 (CH), 140.2 (C_q), 138.0 (C_q), 137.7 (C_q), 135.5 (C_q), 135.5 (CH), 132.9 (CH), 132.7 (CH), 128.2 (C_q), 123.4 (C_q), 122.6 (CH), 121.4 (CH), 121.0 (CH), 120.5 (CH), 119.9 (CH), 114.0 (CH), 79.3 (CH), 56.7 (CH), 56.1 (CH), 50.1 (CH), 42.3 (C_q), 39.7 (CH_2), 39.5 (CH_2), 38.7 (CH_2), 37.3 (CH_2), 36.9 (C_q), 36.2

(CH_2), 35.8 (CH), 32.0 (CH_2), 31.9 (CH), 28.2 (CH_2), 28.1 (CH_2), 28.0 (CH), 24.3 (CH_2), 23.8 (CH_2), 22.8 (CH_3), 22.6 (CH_3), 22.4 (CH_3), 21.1 (CH_2), 19.6 (CH_3), 18.7 (CH_3), 11.8 (CH_3). **IR** (ATR): 3331, 2946, 1655, 1535, 1477, 1221, 1019, 858, 746, 675 cm^{-1}. **MS** (ESI) *m/z* (relative intensity): 669 (15) $[M+Na]^+$, 647 (100) $[M+H]^+$. **HR-MS** (ESI) *m/z* calcd for $C_{44}H_{59}N_2O_2$ $[M+H]^+$: 647.4571, found: 647.4569.

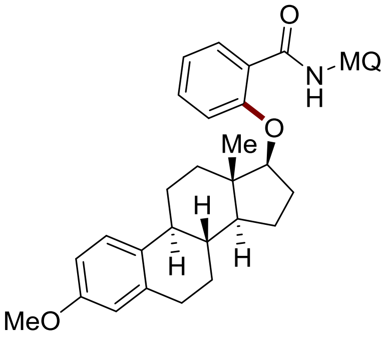

2-{[*(8R,9S,13S,14S,17S)*-3-Methoxy-13-methyl-7,8,9,11,12,13,14,15,16,17-decahydro-*6H*-cyclopenta[a]phenanthren-17-yl]oxy}-*N*-(6-methylquinolin-8-yl) benzamide (3as)

The general procedure was followed using benzamide **1a** (0.25 mmol, 65.5 mg) and *β*-estradiol 3-methyl ether (**2s**) (716 mg, 2.5 mmol). Purification by column chromatography on silica gel (*n*-hexane/EtOAc: 16/1→9/1) yielded **3as** (88.2 mg, 65%) as a white solid. **M.p.**: 193–195 ℃. **^{1}H NMR** (300 MHz, $CDCl_3$) δ = 11.52 (s, 1H), 9.02 (d, *J* = 1.7 Hz, 1H), 8.75 (dd, *J* = 4.2, 1.7 Hz, 1H), 8.27 (dd, *J* = 7.8, 1.9 Hz, 1H), 8.09 (dd, *J* = 8.3, 1.7 Hz, 1H), 7.52–7.46 (m, 1H), 7.43 (dd, *J* = 8.3, 4.2 Hz, 1H), 7.34 (dq, *J* = 1.9, 1.0 Hz, 1H), 7.21 (dd, *J* = 8.4, 6.9 Hz, 2H), 7.16–7.09 (m, 1H), 6.72 (dd, *J* = 8.6, 2.8 Hz, 1H), 6.64 (d, *J* = 2.8 Hz, 1H), 4.76 (t, *J* = 8.2 Hz, 1H), 3.79 (s, 3H), 2.91–2.83 (m, 2H), 2.61 (s, 3H), 2.52–2.21 (m, 4H), 2.17 (dt, *J* = 11.7, 2.7 Hz, 1H), 1.99–1.78 (m, 1H), 1.63–1.28 (m, 7H), 0.91 (s, 3H). **^{13}C NMR** (100 MHz, $CDCl_3$) δ = 164.6 (C_q), 157.4 (C_q), 157.0 (C_q), 146.7 (CH), 137.8 (C_q), 137.7 (C_q), 137.6 (C_q), 135.5 (CH), 135.1 (C_q), 132.6 (CH), 132.4 (CH), 132.3 (C_q), 128.2 (C_q), 126.3 (CH), 123.7 (C_q), 121.4 (CH), 120.9 (CH), 120.6 (CH), 120.1 (CH), 114.2 (CH), 113.8 (CH), 111.4 (CH), 87.8 (CH), 55.2 (CH_3), 50.3 (CH), 44.1 (C_q), 43.7 (CH), 38.6

(CH), 38.1 (CH_2), 29.7 (CH_2), 27.9 (CH_2), 27.2 (CH_2), 26.3 (CH_2), 23.4 (CH_2), 22.3 (CH_3), 12.3 (CH_3). **IR** (ATR): 3273, 2921, 1651, 1530, 1475, 1423, 1252, 760, 696 cm^{-1}. **MS** (EI) *m/z* (relative intensity): 547 (10) $[M+H]^+$, 546 (30) $[M]^+$, 262 (10), 158 (100). **HR-MS** (EI) *m/z* calcd for $C_{36}H_{38}N_2O_3$ $[M]^+$: 546.2877, found: 546.2879.

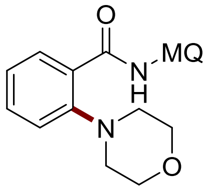

_N_-(6-Methylquinolin-8-yl)-2-morpholinobenzamide (3at)

3at: White solid. **M.p.**: 168–170 ℃. **^{1}H NMR** (400 MHz, $CDCl_3$) δ = 12.64 (s, 1H), 9.03 (d, J = 1.7 Hz, 1H), 8.82 (dd, J = 4.2, 1.7 Hz, 1H), 8.20 (dd, J = 8.0, 1.8 Hz, 1H), 8.11 (dd, J = 8.2, 1.7 Hz, 1H), 7.56–7.50 (m, 1H), 7.46 (dd, J = 8.2, 4.2 Hz, 1H), 7.35–7.34 (m, 1H), 7.32–7.23 (m, 2H), 4.03–3.92 (m, 4H), 3.26–3.12 (m, 4H), 2.61 (d, J = 0.9 Hz, 3H). **^{13}C NMR** (100 MHz, $CDCl_3$) δ = 165.7 (C_q), 151.1 (C_q), 147.2 (CH), 137.7 (C_q), 137.6 (C_q), 135.7 (CH), 135.1 (C_q), 132.3 (CH), 132.1 (CH), 128.9 (C_q), 128.4 (C_q), 124.3 (CH), 121.7 (CH), 120.8 (CH), 119.8 (CH), 119.2 (CH), 66.2 (CH_2), 53.9 (CH_2), 22.4 (CH_3). **IR** (ATR): 2958, 2830, 1659, 1521, 1478, 1114, 919, 763, 704 cm^{-1}. **MS** (ESI) *m/z* (relative intensity): 370 (45) $[M+Na]^+$, 348 (100) $[M+H]^+$. **HR-MS** (ESI) *m/z* calcd for $C_{21}H_{22}N_3O_2$ $[M+H]^+$: 348.1707, found: 348.1711.

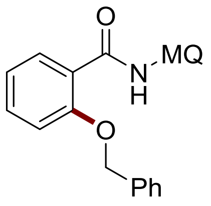

2-(Benzyloxy)-_N_-(6-methylquinolin-8-yl)benzamide (3au)

3au: White solid. **M.p.**: 104–106 ℃. **^{1}H NMR** (400 MHz, $CDCl_3$) δ = 12.22 (s, 1H), 8.92 (d, J = 1.8 Hz, 1H), 8.40 (dd, J = 4.2, 1.7 Hz, 1H), 8.32 (dd, J = 7.8, 1.8 Hz, 1H), 8.01 (dd, J = 8.2, 1.7 Hz, 1H), 7.57–7.52 (m, 2H), 7.40 (ddd, J = 8.3, 7.3, 1.9 Hz, 1H), 7.33–7.24 (m, 5H), 7.13–7.07 (m, 1H), 7.05 (dd, J = 8.4, 1.0 Hz, 1H), 5.49 (s, 2H),

2.56 (d, J = 1.0 Hz, 3H). **^{13}C NMR** (100 MHz, $CDCl_3$**)** δ = 163.8 (C_q), 156.6 (C_q), 147.0 (CH), 137.7 (C_q), 137.6 (C_q), 136.3 (C_q), 135.4 (CH), 135.1 (C_q), 132.8 (CH), 132.3 (CH), 128.6 (CH), 128.0 (CH), 128.0 (C_q), 127.5 (CH), 123.2 (C_q), 121.5 (CH), 121.3 (CH), 120.4 (CH), 119.5 (CH), 113.4 (CH), 71.1 (CH_2), 22.3 (CH_3). **IR** (ATR): 3277, 1655, 1526, 1475, 1424, 1209, 1001, 853, 732, 691 cm^{-1}. **MS** (ESI) *m/z* (relative intensity): 391 (10) $[M+Na]^+$, 369 (10) $[M+H]^+$. **HR-MS** (ESI) *m/z* calcd for $C_{24}H_{21}N_2O_2$ $[M+H]^+$: 369.1598, found: 369.1599.

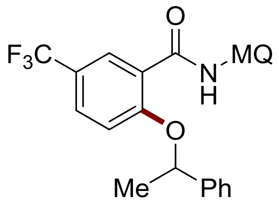

N-(6-Methylquinolin-8-yl)-2-(1-phenylethoxy)-5-(trifluoromethyl)benzamide (3ba)

The general procedure was followed using benzamide **1b** (0.25 mmol, 82.5 mg) and 1-phenylethan-1-ol (**2a**) (305 mg, 2.5 mmol). Purification by column chromatography on silica gel (*n*-hexane/EtOAc: 16/1) yielded **3ba** (81.5 mg, 72%) as a white solid. **M.p.**: 136–137 ℃. **^{1}H NMR** (400 MHz, $CDCl_3$) δ = 12.08 (s, 1H), 8.99 (d, J = 1.7 Hz, 1H), 8.70 (dd, J = 4.2, 1.7 Hz, 1H), 8.60 (dd, J = 2.5, 0.8 Hz, 1H), 8.08 (dd, J = 8.3, 1.7 Hz, 1H), 7.62–7.56 (m, 2H), 7.52 (ddd, J = 8.7, 2.5, 0.8 Hz, 1H), 7.40 (dd, J = 8.3, 4.2 Hz, 1H), 7.37–7.32 (m, 3H), 7.30–7.25 (m, 1H), 6.98 (d, J = 8.7 Hz, 1H), 5.66 (q, J = 6.4 Hz, 1H), 2.59 (d, J = 0.9 Hz, 3H), 1.99 (d, J = 6.4 Hz, 3H). **^{13}C NMR** (100 MHz, $CDCl_3$) δ = 162.6 (C_q), 158.1 (C_q), 146.9 (CH), 141.6 (C_q), 137.8 (C_q), 137.7 (C_q), 135.6 (CH), 135.0 (C_q), 130.1 (q, $^3J_{C\text{-}F}$ = 3.8 Hz, CH), 129.6 (q, $^3J_{C\text{-}F}$ = 3.5 Hz, CH), 129.0 (CH), 128.1 (CH), 128.1 (C_q), 125.5 (CH), 123.9 (q, $^1J_{C\text{-}F}$ = 271.7 Hz, C_q), 123.4 (C_q), 123.3 (q, $^2J_{C\text{-}F}$ = 33.3 Hz, C_q), 121.6 (CH), 120.9 (CH), 120.0 (CH), 114.3 (CH), 78.6 (CH), 25.0 (CH_3), 22.4 (CH_3). **^{19}F NMR** (282 MHz, $CDCl_3$) δ = -61.9 (s). **IR** (ATR): 3307, 1660, 1529, 1329, 1270, 1114, 1066, 738, 700 cm^{-1}. **MS** (ESI) *m/z* (relative intensity): 473 (10) $[M+Na]^+$, 451 (100) $[M+H]^+$, 347 (45), 105 (40). **HR-MS** (ESI) *m/z* calcd for $C_{26}H_{22}F_3N_2O_2$ $[M+H]^+$: 451.1628, found: 451.1640.

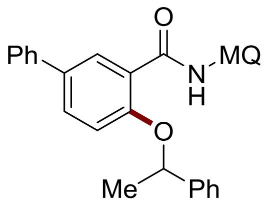

***N*-(6-methylquinolin-8-yl)-4-(1-phenylethoxy)-[1,1'-biphenyl]-3-carboxamide (3ca)**

The general procedure was followed using benzamide **1c** (0.25 mmol, 84.5 mg) and 1-phenylethan-1-ol (**2a**) (305 mg, 2.5 mmol). Purification by column chromatography on silica gel (*n*-hexane/aceton: 20/1) yielded **3ca** (65.5 mg, 57%) as a white solid. **M.p.**: 120–122 °C. **^{1}H NMR** (400 MHz, $CDCl_3$) δ = 12.22 (s, 1H), 9.09 (d, J = 1.8 Hz, 1H), 8.75 (dd, J = 4.2, 1.7 Hz, 1H), 8.63 (d, J = 2.6 Hz, 1H), 8.12 (dd, J = 8.2, 1.7 Hz, 1H), 7.67–7.61 (m, 4H), 7.57 (dd, J = 8.6, 2.6 Hz, 1H), 7.47–7.43 (m, 2H), 7.43–7.36 (m, 4H), 7.36–7.29 (m, 2H), 7.02 (d, J = 8.6 Hz, 1H), 5.70 (q, J = 6.5 Hz, 1H), 2.64 (d, J = 0.9 Hz, 3H), 2.02 (d, J = 6.5 Hz, 3H). **^{13}C NMR** (100 MHz, $CDCl_3$) δ = 164.1 (C_q), 155.5 (C_q), 146.9 (CH), 142.5 (C_q), 139.8 (C_q), 138.0 (C_q), 137.8 (C_q), 135.6 (CH), 135.5 (C_q), 133.9 (C_q), 131.1 (CH), 131.0 (CH), 128.9 (CH), 128.8 (CH), 128.2 (C_q), 127.9 (CH), 127.0 (CH), 126.7 (CH), 125.7 (CH), 123.2 (C_q), 121.6 (CH), 120.6 (CH), 119.9 (CH), 114.5 (CH), 78.1 (CH), 25.1 (CH_3), 22.4 (CH_3). **IR** (ATR): 3303, 2924, 1656, 1525, 1422, 1266, 1067, 760, 735, 698 cm^{-1}. **MS** (ESI) *m/z* (relative intensity): 481 (15) $[M+Na]^+$, 459 (100) $[M+H]^+$, 352 (30). **HR-MS** (ESI) *m/z* calcd for $C_{31}H_{27}N_2O_2$ $[M+H]^+$: 459.2067, found: 459.2069.

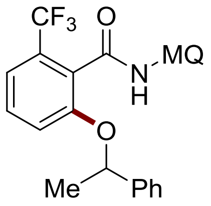

***N*-(6-Methylquinolin-8-yl)-2-(1-phenylethoxy)-6-(trifluoromethyl)benzamide (3da)**

The general procedure was followed using benzamide **1d** (0.25 mmol, 82.5 mg) and 1-phenylethan-1-ol (**2a**) (305 mg, 2.5 mmol). Purification by column chromatography

on silica gel (*n*-hexane/aceton: 16/1) yielded **3da** (72.2 mg, 64%) as a white solid. **M.p.**: 126–128 ℃. **^{1}H NMR** (400 MHz, $CDCl_3$) δ = 10.11 (s, 1H), 8.88 (d, J = 1.7 Hz, 1H), 8.72 (dd, J = 4.2, 1.6 Hz, 1H), 8.11 (dd, J = 8.3, 1.6 Hz, 1H), 7.46–7.39 (m, 3H), 7.37 (td, J = 2.0, 1.0 Hz, 1H), 7.34 (dd, J = 8.1, 1.0 Hz, 1H), 7.31–7.26 (m, 3H), 7.26–7.21 (m, 1H), 7.03 (d, J = 8.1 Hz, 1H), 5.40 (q, J = 6.5 Hz, 1H), 2.64 (d, J = 1.0 Hz, 3H), 1.53 (d, J = 6.5 Hz, 3H). **^{13}C NMR** (100 MHz, $CDCl_3$) δ = 163.6 (C_q), 155.2 (C_q), 147.2 (CH), 142.1 (C_q), 137.8 (C_q), 137.2 (C_q), 135.6 (CH), 134.1 (C_q), 130.4 (CH), 129.2 (q, $^2J_{C\text{-}F}$ = 31.8 Hz, C_q), 128.7 (CH), 128.1 (C_q), 127.7 (CH), 126.2 (q, $^3J_{C\text{-}F}$ = 2.2 Hz, C_q), 125.5 (CH), 123.5 (q, $^1J_{C\text{-}F}$ = 274.2 Hz, C_q), 121.7 (CH), 120.9 (CH), 119.0 (CH), 118.2 (q, $^3J_{C\text{-}F}$ = 5.0 Hz, CH), 117.6 (CH), 77.5 (CH), 24.5 (CH_3), 22.4 (CH_3). **^{19}F NMR** (376 MHz, $CDCl_3$) δ = -59.2 (s). **IR** (ATR): 3343, 1684, 1527, 1318, 1268, 1131, 701 cm^{-1}. **MS** (ESI) *m/z* (relative intensity): 473 (90) $[M+Na]^+$, 451 (100) $[M+H]^+$. **HR-MS** (ESI) *m/z* calcd for $C_{26}H_{22}F_3N_2O_2$ $[M+H]^+$: 451.1628, found: 451.1625.

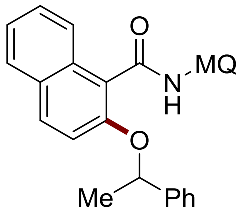

***N*-(6-Methylquinolin-8-yl)-2-(1-phenylethoxy)-1-naphthamide (3ea)**

The general procedure was followed using benzamide **1e** (0.25 mmol, 78.0 mg) and 1-phenylethan-1-ol (**2a**) (305 mg, 2.5 mmol). Purification by column chromatography on silica gel (*n*-hexane/EtOAc: 16/1→9/1) yielded **3ea** (92.3 mg, 86%) as a white solid. **M.p.**: 155–157 ℃. **^{1}H NMR** (400 MHz, $CDCl_3$) δ = 10.47 (s, 1H), 9.05 (d, J = 1.8 Hz, 1H), 8.65 (dd, J = 4.2, 1.7 Hz, 1H), 8.19 (dq, J = 8.6, 1.0 Hz, 1H), 8.07 (dd, J = 8.3, 1.7 Hz, 1H), 7.77–7.72 (m, 2H), 7.51–7.44 (m, 3H), 7.40–7.31 (m, 3H), 7.27–7.21 (m, 2H), 7.21–7.14 (m, 2H), 5.52 (q, J = 6.4 Hz, 1H), 2.64 (d, J = 1.0 Hz, 3H), 1.57 (d, J = 6.4 Hz, 3H). **^{13}C NMR** (100 MHz, $CDCl_3$) δ = 165.9 (C_q), 152.4 (C_q), 147.1 (CH), 142.8 (C_q), 137.7 (C_q), 137.3 (C_q), 135.5 (CH), 134.6 (C_q), 131.9

(C_q), 131.2 (CH), 128.9 (C_q), 128.6 (CH), 128.1 (C_q), 127.9 (CH), 127.5 (CH), 127.4 (CH), 125.7 (CH), 124.7 (CH), 124.2 (CH), 121.7 (C_q), 121.6 (CH), 120.6 (CH), 118.7 (CH), 115.6 (CH), 77.8 (CH), 24.5 (CH_3), 22.4 (CH_3). **IR** (ATR): 3351, 1669, 1525, 1424, 1246, 1070, 701 cm^{-1}. **MS** (ESI) *m/z* (relative intensity): 455 (10) $[M+Na]^+$, 433 (100) $[M+H]^+$, 329 (45), 159 (50). **HR-MS** (ESI) *m/z* calcd for $C_{29}H_{25}N_2O_2$ $[M+H]^+$: 433.1911, found: 433.1907.

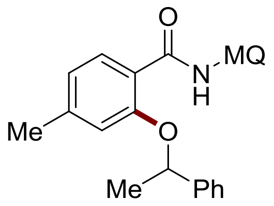

4-Methyl-*N*-(6-methylquinolin-8-yl)-2-(1-phenylethoxy)benzamide (3fa)

The general procedure was followed using benzamide **1f** (0.25 mmol, 69.0 mg) and 1-phenylethan-1-ol (**2a**) (305 mg, 2.5 mmol). Purification by column chromatography on silica gel (*n*-hexane/EtOAc: 16/1→9/1) yielded **3fa** (54.4 mg, 55%) as a white solid. **M.p.**: 160–162 ℃. **^{1}H NMR** (400 MHz, $CDCl_3$) δ = 12.12 (s, 1H), 9.01 (d, J = 1.8 Hz, 1H), 8.67 (dd, J = 4.2, 1.7 Hz, 1H), 8.19 (d, J = 8.0 Hz, 1H), 8.05 (dd, J = 8.2, 1.7 Hz, 1H), 7.62–7.57 (m, 2H), 7.37 (dd, J = 8.2, 4.2 Hz, 1H), 7.35–7.30 (m, 2H), 7.30–7.29 (m, 1H), 7.28–7.20 (m, 1H), 6.84 (ddd, J = 8.0, 1.5, 0.8 Hz, 1H), 6.77–6.68 (m, 1H), 5.62 (q, J = 6.5 Hz, 1H), 2.58 (d, J = 1.0 Hz, 3H), 2.25 (d, J = 0.8 Hz, 3H), 1.94 (d, J = 6.5 Hz, 3H). **^{13}C NMR** (100 MHz, $CDCl_3$) δ = 164.2 (C_q), 156.0 (C_q), 146.7 (CH), 143.5 (C_q), 142.5 (C_q), 138.0 (C_q), 137.7 (C_q), 135.6 (C_q), 135.4 (CH), 132.4 (CH), 128.8 (CH), 128.1 (C_q), 127.7 (CH), 125.7 (CH), 122.0 (CH), 121.4 (CH), 120.4 (C_q), 120.4 (CH), 119.7 (CH), 114.7 (CH), 77.7 (CH), 25.0 (CH_3), 22.4 (CH_3), 21.7 (CH_3). **IR** (ATR): 3305, 1655, 1528, 1423, 1286, 1258, 1067, 762, 701 cm^{-1}. **MS** (ESI) *m/z* (relative intensity): 419 (10) $[M+Na]^+$, 397 (100) $[M+H]^+$, 293 (70), 159 (40). **HR-MS** (ESI) *m/z* calcd for $C_{26}H_{25}N_2O_2$ $[M+H]^+$: 397.1911, found: 397.1909.

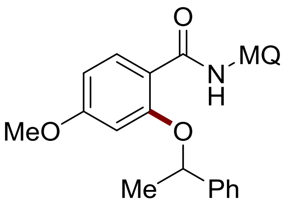

4-Methoxy-*N*-(6-methylquinolin-8-yl)-2-(1-phenylethoxy)benzamide (3ga)

The general procedure was followed using benzamide **1g** (0.25 mmol, 73.0 mg) and 1-phenylethan-1-ol (**2a**) (305 mg, 2.5 mmol). Purification by column chromatography on silica gel (*n*-hexane/EtOAc: 16/1→9/1) yielded **3ga** (74.4 mg, 72%) as a white solid. **M.p.**: 130–131 ℃. ^{1}**H NMR** (300 MHz, $CDCl_3$) δ = 12.10 (s, 1H), 9.05 (d, J = 1.8 Hz, 1H), 8.72 (dd, J = 4.2, 1.7 Hz, 1H), 8.31 (d, J = 8.8 Hz, 1H), 8.09 (dd, J = 8.3, 1.7 Hz, 1H), 7.68–7.59 (m, 2H), 7.41 (dd, J = 7.7, 3.7 Hz, 1H), 7.38 (d, J = 5.7 Hz, 1H), 7.37–7.32 (m, 2H), 7.32–7.26 (m, 1H), 6.59 (dd, J = 8.8, 2.3 Hz, 1H), 6.47 (d, J = 2.3 Hz, 1H), 5.62 (q, J = 6.4 Hz, 1H), 3.74 (s, 3H), 2.61 (d, J = 0.9 Hz, 3H), 2.01 (d, J = 6.4 Hz, 3H). ^{13}**C NMR** (100 MHz, $CDCl_3$) δ = 164.0 (C_q), 163.3 (C_q), 157.4 (C_q), 146.7 (CH), 142.4 (C_q), 138.1 (C_q), 137.7 (C_q), 135.7 (C_q), 135.5 (CH), 134.1 (CH), 128.9 (CH), 128.2 (C_q), 127.9 (CH), 125.7 (CH), 121.5 (CH), 120.3 (CH), 119.7 (CH), 116.0 (C_q), 105.7 (CH), 100.9 (CH), 78.0 (CH), 55.3 (CH_3), 25.1 (CH_3), 22.4 (CH_3). **IR** (ATR): 3308, 1651, 1523, 1476, 1261, 1247, 1168, 701 cm^{-1}. **MS** (ESI) *m/z* (relative intensity): 435 (10) $[M+Na]^+$, 413 (100) $[M+H]^+$, 309 (30). **HR-MS** (ESI) *m/z* calcd for $C_{26}H_{25}N_2O_3$ $[M+H]^+$: 413.1860, found: 413.1865.

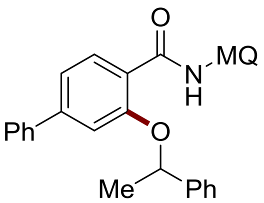

***N*-(6-Methylquinolin-8-yl)-3-(1-phenylethoxy)-(1,1'-biphenyl)-4-carboxamide (3ha)**

The general procedure was followed using benzamide **1h** (0.25 mmol, 84.5 mg) and 1-phenylethan-1-ol (**2a**) (305 mg, 2.5 mmol). Purification by column chromatography on silica gel (*n*-hexane/aceton: 16/1→9/1) yielded **3ha** (75 mg, 66%) as a white solid.

M.p.: 161–162 ℃. **^{1}H NMR** (400 MHz, $CDCl_3$) δ = 12.21 (s, 1H), 9.07 (d, J = 1.7 Hz, 1H), 8.75 (dd, J = 4.2, 1.7 Hz, 1H), 8.39 (d, J = 8.1 Hz, 1H), 8.12 (dd, J = 8.3, 1.7 Hz, 1H), 7.73–7.61 (m, 2H), 7.47–7.42 (m, 5H), 7.40–7.35 (m, 4H), 7.36–7.26 (m, 2H), 7.18 (d, J = 1.5 Hz, 1H), 5.75 (q, J = 6.4 Hz, 1H), 2.63 (d, J = 0.9 Hz, 3H), 2.04 (d, J = 6.4 Hz, 3H). **^{13}C NMR** (100 MHz, $CDCl_3$) δ = 164.0 (C_q), 156.3 (C_q), 146.8 (CH), 145.6 (C_q), 142.5 (C_q), 140.1 (C_q), 138.1 (C_q), 137.8 (C_q), 135.5 (CH), 135.5 (C_q), 133.0 (CH), 128.9 (CH), 128.8 (CH), 128.2 (C_q), 128.0 (CH), 127.9 (CH), 127.1 (CH), 125.8 (CH), 121.8 (C_q), 121.5 (CH), 120.6 (CH), 119.9 (CH), 119.8 (CH), 113.0 (CH), 78.1 (CH), 25.0 (CH_3), 22.4 (CH_3). **IR** (ATR): 3305, 1655, 1529, 1475, 1422, 1199, 854, 756, 697 cm^{-1}. **MS** (ESI) *m/z* (relative intensity): 481 (15) $[M+Na]^+$, 459 (100) $[M+H]^+$. **HR-MS** (ESI) *m/z* calcd for $C_{31}H_{27}N_2O_2$ $[M+H]^+$: 459.2067, found: 459.2067.

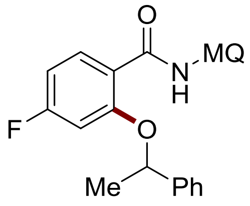

4-Fluoro-*N*-(6-methylquinolin-8-yl)-2-(1-phenylethoxy)benzamide (3ia)

The general procedure was followed using benzamide **1i** (0.25 mmol, 70.0 mg) and 1-phenylethan-1-ol (**2a**) (305 mg, 2.5 mmol). Purification by column chromatography on silica gel (*n*-hexane/EtOAc: 16/1→9/1) yielded **3ia** (81.1 mg, 81%) as a white solid. **M.p.**: 157–159 ℃. **^{1}H NMR** (400 MHz, $CDCl_3$) δ = 12.08 (s, 1H), 9.02 (d, J = 1.8 Hz, 1H), 8.73 (dd, J = 4.2, 1.7 Hz, 1H), 8.35 (dd, J = 8.8, 7.1 Hz, 1H), 8.11 (dd, J = 8.2, 1.7 Hz, 1H), 7.65–7.60 (m, 2H), 7.43 (dd, J = 8.2, 4.2 Hz, 1H), 7.41–7.34 (m, 3H), 7.34–7.29 (m, 1H), 6.76 (ddd, J = 8.8, 7.6, 2.4 Hz, 1H), 6.66 (dd, J = 10.9, 2.4 Hz, 1H), 5.59 (q, J = 6.4 Hz, 1H), 2.62 (d, J = 0.9 Hz, 3H), 2.01 (d, J = 6.4 Hz, 3H). **^{13}C NMR** (100 MHz, $CDCl_3$) δ =165.3 (d, $^{1}J_{C–F}$ = 252.0 Hz, C_q), 163.2 (C_q), 157.4 (d, $^{3}J_{C–F}$ = 10.7 Hz, C_q), 146.9 (CH), 141.7 (C_q), 138.0 (C_q), 137.7 (C_q), 135.6 (CH), 135.4 (C_q), 134.4 (d, $^{3}J_{C–F}$ = 10.7 Hz, CH), 129.0 (CH), 128.2 (C_q), 128.1 (CH), 125.7 (CH), 121.6 (CH), 120.6 (CH), 119.9 (CH), 119.4 (d, $^{4}J_{C–F}$ = 3.0 Hz, C_q), 108.1

(d, $^2J_{C-F}$ = 21.4 Hz, CH), 102.0 (d, $^2J_{C-F}$ = 26.4 Hz, CH), 78.6 (CH), 25.0 (CH_3), 22.4 (CH_3). **^{19}F NMR** (376 MHz, $CDCl_3$) δ = -105.3 (s). **IR** (ATR): 3309, 1657, 1528, 1477, 1428, 1261, 997, 836, 701 cm^{-1}. **MS** (ESI) *m/z* (relative intensity): 423 (15) $[M+Na]^+$, 401 (100) $[M+H]^+$, 297 (75), 159 (35), 105 (30). **HR-MS** (ESI) *m/z* calcd for $C_{25}H_{22}FN_2O_2$ $[M+H]^+$: 401.1660, found: 401.1660.

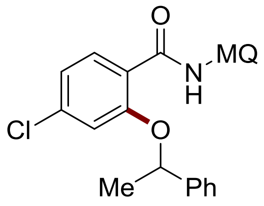

4-Chloro-*N*-(6-methylquinolin-8-yl)-2-(1-phenylethoxy)benzamide (3ja)

The general procedure was followed using benzamide **1j** (0.25 mmol, 74.0 mg) and 1-phenylethan-1-ol (**2a**) (305 mg, 2.5 mmol) at 3 mA for 32 h. Purification by column chromatography on silica gel (*n*-hexane/EtOAc: 16/1→9/1) yielded **3ja** (67.3 mg, 65%) as a white solid. **M.p.**: 115–117 ℃. **^{1}H NMR** (400 MHz, $CDCl_3$) δ = 12.08 (s, 1H), 9.01 (d, *J* = 1.8 Hz, 1H), 8.72 (dd, *J* = 4.2, 1.7 Hz, 1H), 8.27 (d, *J* = 8.5 Hz, 1H), 8.11 (dd, *J* = 8.3, 1.7 Hz, 1H), 7.68–7.60 (m, 2H), 7.43 (dd, *J* = 8.3, 4.2 Hz, 1H), 7.41–7.35 (m, 3H), 7.34–7.29 (m, 1H), 7.05 (dd, *J* = 8.5, 1.9 Hz, 1H), 6.95 (d, *J* = 1.9 Hz, 1H), 5.63 (q, *J* = 6.4 Hz, 1H), 2.62 (d, *J* = 0.9 Hz, 3H), 2.00 (d, *J* = 6.4 Hz, 3H). **^{13}C NMR** (100 MHz, $CDCl_3$) δ = 163.2 (C_q), 156.4 (C_q), 146.9 (CH), 141.7 (C_q), 138.4 (C_q), 138.0 (C_q), 137.7 (C_q), 135.6 (CH), 135.3 (C_q), 133.6 (CH), 129.0 (CH), 128.2 (C_q), 128.1 (CH), 125.7 (CH), 121.7 (C_q), 121.6 (CH), 121.5 (CH), 120.8 (CH), 119.9 (CH), 114.6 (CH), 78.5 (CH), 25.0 (CH_3), 22.4 (CH_3). **IR** (ATR): 3306, 1662, 1531, 1475, 1426, 1229, 948, 855, 701 cm^{-1}. **MS** (ESI) *m/z* (relative intensity): 439 (5) $[M+Na]^+$, 417 (100) $[M+H]^+$, 313 (30), 263 (30). **HR-MS** (ESI) *m/z* calcd for $C_{25}H_{22}ClN_2O_2$ $[M+H]^+$: 417.1364, found: 417.1372.

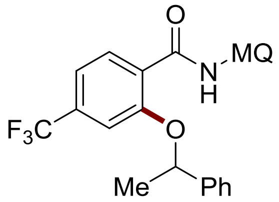

***N*-(6-Methylquinolin-8-yl)-2-(1-phenylethoxy)-4-(trifluoromethyl)benzamide (3ka)**

The general procedure was followed using benzamide **1k** (0.25 mmol, 82.5 mg) and 1-phenylethan-1-ol (**2a**) (305 mg, 2.5 mmol). Purification by column chromatography on silica gel (*n*-hexane/aceton: 16/1) yielded **3ka** (93.5 mg, 83%) as a white solid. **M.p.**: 153–154 ℃. **^{1}H NMR** (400 MHz, $CDCl_3$) δ = 12.13 (s, 1H), 9.02 (d, J = 1.7 Hz, 1H), 8.75 (dd, J = 4.2, 1.7 Hz, 1H), 8.43 (dd, J = 8.2, 1.0 Hz, 1H), 8.13 (dd, J = 8.2, 1.7 Hz, 1H), 7.68–7.61 (m, 2H), 7.45 (dd, J = 8.2, 4.2 Hz, 1H), 7.41–7.35 (m, 3H), 7.35–7.29 (m, 2H), 7.22 (d, J = 1.5 Hz, 1H), 5.70 (q, J = 6.4 Hz, 1H), 2.63 (d, J = 0.9 Hz, 3H), 2.02 (d, J = 6.4 Hz, 3H). **^{13}C NMR** (100 MHz, $CDCl_3$) δ = 162.8 (C_q), 155.9 (C_q), 147.0 (CH), 141.4 (C_q), 137.9 (C_q), 137.7 (C_q), 135.6 (CH), 135.0 (C_q), 134.0 (q, $^2J_{C\text{-}F}$ = 32.4 Hz, C_q), 133.2 (CH), 129.0 (CH), 128.2 (CH), 128.2 (C_q), 126.3 (C_q), 125.8 (CH), 123.4 (q, $^1J_{C\text{-}F}$ = 272.8 Hz, C_q), 121.7 (CH), 121.0 (CH), 120.0 (CH), 117.7 (q, $^3J_{C\text{-}F}$ = 3.8 Hz, CH), 111.2 (q, $^3J_{C\text{-}F}$ = 3.9 Hz, CH), 78.7 (CH), 24.8 (CH_3), 22.4 (CH_3). **^{19}F NMR** (376 MHz, $CDCl_3$) δ = -63.2 (s). **IR** (ATR): 3297, 1655, 1531, 1424, 1320, 1163, 1116, 952, 852, 702 cm^{-1}. **MS** (ESI) *m/z* (relative intensity): 473 (10) $[M+Na]^+$, 451 (100) $[M+H]^+$, 347 (50), 105 (20). **HR-MS** (ESI) *m/z* calcd for $C_{26}H_{22}F_3N_2O_2$ $[M+H]^+$: 451.1628, found: 451.1628.

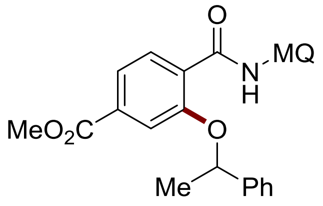

Methyl 4-[(6-methylquinolin-8-yl)carbamoyl]-3-(1-phenylethoxy)benzoate (3la)

The general procedure was followed using benzamide **1l** (0.25 mmol, 80.0 mg) and 1-phenylethan-1-ol (**2a**) (305 mg, 2.5 mmol). Purification by column chromatography on silica gel (*n*-hexane/aceton: 10/1) yielded **3la** (62.3 mg, 57%) as a white solid. **M.p.**: 178–180 ℃. **^{1}H NMR** (400 MHz, $CDCl_3$) δ = 12.18 (s, 1H), 9.03 (d, J = 1.8 Hz, 1H), 8.74 (dd, J = 4.2, 1.7 Hz, 1H), 8.37 (d, J = 8.5 Hz, 1H), 8.12 (dd, J = 8.2,

1.7 Hz, 1H), 7.77–7.60 (m, 4H), 7.44 (dd, J = 8.2, 4.2 Hz, 1H), 7.38–7.32 (m, 3H), 7.32–7.26 (m, 1H), 5.77 (q, J = 6.4 Hz, 1H), 3.91 (s, 3H), 2.63 (s, 3H), 2.01 (d, J = 6.4 Hz, 3H). **^{13}C NMR** (100 MHz, $CDCl_3$) δ = 166.2 (C_q), 163.2 (C_q), 155.7 (C_q), 147.0 (CH), 141.8 (C_q), 138.0 (C_q), 137.7 (C_q), 135.6 (CH), 135.2 (C_q), 133.8 (C_q), 132.6 (CH), 128.9 (CH), 128.2 (C_q), 128.0 (CH), 127.0 (C_q), 125.9 (CH), 121.8 (CH), 121.6 (CH), 120.9 (CH), 120.0 (CH), 115.3 (CH), 78.2 (CH), 52.4 (CH_3), 24.8 (CH_3), 22.4 (CH_3). **IR** (ATR): 3292, 1721, 1656, 1529, 1434, 1283, 1216, 1009, 738, 704 cm^{-1}. **MS** (ESI) *m/z* (relative intensity): 463 (10) $[M+Na]^+$, 441 (100) $[M+H]^+$, 337 (55), 159 (15), 105 (10). **HR-MS** (ESI) *m/z* calcd for $C_{27}H_{25}N_2O_4$ $[M+H]^+$: 441.1809, found: 441.1810.

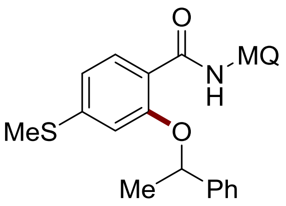

***N*-(6-Methylquinolin-8-yl)-4-(methylthio)-2-(1-phenylethoxy)benzamide (3ma)**

The general procedure was followed using benzamide **1m** (0.25 mmol, 77.0 mg) and 1-phenylethan-1-ol (**2a**) (305 mg, 2.5 mmol). Purification by column chromatography on silica gel (*n*-hexane/aceton: 10/1) yielded **3ma** (56.2 mg, 53%) as a white solid. **M.p.**: 120–122 °C. **^{1}H NMR** (400 MHz, $CDCl_3$) δ = 12.10 (s, 1H), 9.03 (d, J = 1.8 Hz, 1H), 8.73 (dd, J = 4.2, 1.7 Hz, 1H), 8.25 (d, J = 8.3 Hz, 1H), 8.10 (dd, J = 8.2, 1.7 Hz, 1H), 7.67–7.59 (m, 2H), 7.42 (dd, J = 8.2, 4.2 Hz, 1H), 7.38 (dd, J = 8.3, 6.7 Hz, 2H), 7.34 (d, J = 1.4 Hz, 1H), 7.33–7.29 (m, 1H), 6.90 (dd, J = 8.3, 1.7 Hz, 1H), 6.76 (d, J = 1.8 Hz, 1H), 5.63 (q, J = 6.5 Hz, 1H), 2.61 (d, J = 0.9 Hz, 3H), 2.37 (s, 3H), 2.00 (d, J = 6.5 Hz, 3H). **^{13}C NMR** (100 MHz, $CDCl_3$) δ = 163.8 (C_q), 156.1 (C_q), 146.8 (CH), 144.8 (C_q), 142.3 (C_q), 138.0 (C_q), 137.7 (C_q), 135.5 (CH), 135.5 (C_q), 132.7 (CH), 129.0 (CH), 128.2 (C_q), 128.0 (CH), 125.7 (CH), 121.5 (CH), 120.5 (CH), 119.8 (CH), 119.5 (C_q), 118.0 (CH), 111.0 (CH), 78.2 (CH), 25.0 (CH_3), 22.4 (CH_3), 14.9 (CH_3). **IR** (ATR): 3307, 1655, 1528, 1478, 1423, 1222, 950, 702 cm^{-1}.

MS (ESI) *m/z* (relative intensity): 451 (10) $[M+Na]^+$, 429 (100) $[M+H]^+$, 325 (20). **HR-MS** (ESI) *m/z* calcd for $C_{26}H_{25}N_2O_2S$ $[M+H]^+$: 429.1631, found: 429.1637.

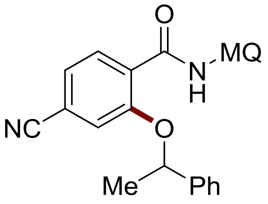

4-Cyano-*N*-(6-methylquinolin-8-yl)-2-(1-phenylethoxy)benzamide (3na)

The general procedure was followed using benzamide **1n** (0.25 mmol, 71.8 mg) and 1-phenylethan-1-ol (**2a**) (305 mg, 2.5 mmol). Purification by column chromatography on silica gel (*n*-hexane/EtOAc: 16/1→9/1) yielded **3na** (57.0 mg, 56%) as a white solid. **M.p.**: 214–216 ℃. **^{1}H NMR** (400 MHz, $CDCl_3$) δ = 12.10 (s, 1H), 8.99 (d, J = 1.7 Hz, 1H), 8.74 (dd, J = 4.2, 1.7 Hz, 1H), 8.40 (d, J = 8.0 Hz, 1H), 8.13 (dd, J = 8.2, 1.7 Hz, 1H), 7.65–7.60 (m, 2H), 7.45 (dd, J = 8.2, 4.2 Hz, 1H), 7.43–7.37 (m, 3H), 7.36–7.31 (m, 2H), 7.18 (d, J = 1.3 Hz, 1H), 5.64 (q, J = 6.4 Hz, 1H), 2.63 (d, J = 0.9 Hz, 3H), 2.01 (d, J = 6.4 Hz, 3H). **^{13}C NMR** (100 MHz, $CDCl_3$) δ = 162.2 (C_q), 155.8 (C_q), 147.0 (CH), 141.1 (C_q), 137.9 (C_q), 137.7 (C_q), 135.7 (CH), 134.8 (C_q), 133.4 (CH), 129.2 (CH), 128.4 (CH), 128.2 (C_q), 127.4 (C_q), 125.6 (CH), 124.6 (CH), 121.7 (CH), 121.2 (CH), 120.1 (CH), 118.1 (C_q), 117.5 (CH), 115.7 (C_q), 79.0 (CH), 25.0 (CH_3), 22.4 (CH_3). **IR** (ATR): 3306, 2230, 1664, 1533, 1426, 1282, 854, 702 cm^{-1}. **MS** (ESI) *m/z* (relative intensity): 430 (10) $[M+Na]^+$, 408 (100) $[M+H]^+$, 117 (50). **HR-MS** (ESI) *m/z* calcd for $C_{26}H_{22}N_3O_2$ $[M+H]^+$: 408.1707, found: 408.1710.

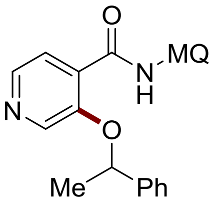

***N*-(6-Methylquinolin-8-yl)-3-(1-phenylethoxy)isonicotinamide (3oa)**

The general procedure was followed using benzamide **1o** (0.25 mmol, 65.8 mg) and 1-phenylethan-1-ol (**2a**) (305 mg, 2.5 mmol). Purification by column chromatography on silica gel (*n*-hexane/EtOAc: 9/1→4/1) yielded **3oa** (52.5 mg, 55%) as a white solid. **M.p.**: 156–158 ℃. **^{1}H NMR** (400 MHz, $CDCl_3$) δ = 12.13 (s, 1H), 9.00 (d, J = 1.9 Hz, 1H), 8.76 (dd, J = 4.2, 1.7 Hz, 1H), 8.43 (s, 1H), 8.36 (d, J = 4.9 Hz, 1H), 8.18–8.09 (m, 2H), 7.62 (dd, J = 7.4, 1.9 Hz, 2H), 7.45 (dd, J = 8.2, 4.2 Hz, 1H), 7.42–7.34 (m, 3H), 7.33–7.25 (m, 1H), 5.79 (q, J = 6.4 Hz, 1H), 2.62 (s, 3H), 2.04 (d, J = 6.4 Hz, 3H). **^{13}C NMR** (100 MHz, $CDCl_3$) δ = 162.0 (C_q), 150.9 (C_q), 147.1 (CH), 143.0 (CH), 141.4 (C_q), 137.9 (C_q), 137.7 (C_q), 137.6 (CH), 135.6 (CH), 134.8 (C_q), 129.3 (C_q), 129.1 (CH), 128.3 (CH), 128.2 (C_q), 125.7 (CH), 124.7 (CH), 121.7 (CH), 121.3 (CH), 120.1 (CH), 78.8 (CH), 25.1 (CH_3), 22.4 (CH_3). **IR** (ATR): 3298, 1663, 1528, 1424, 1224, 1064, 844, 734, 696 cm^{-1}. **MS** (ESI) *m/z* (relative intensity): 406 (40) [M+Na]$^+$, 384 (100) [M+H]$^+$. **HR-MS** (ESI) *m/z* calcd for $C_{24}H_{22}N_3O_2$ [M+H]$^+$: 384.1707, found: 384.1706.

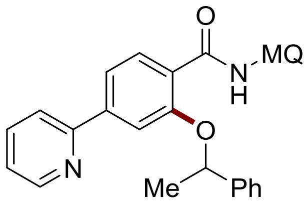

N-(6-Methylquinolin-8-yl)-2-(1-phenylethoxy)-4-(pyridin-2-yl)benzamide (3pa)

The general procedure was followed using benzamide **1p** (0.25 mmol, 84.8 mg) and 1-phenylethan-1-ol (**2a**) (305 mg, 2.5 mmol). Purification by column chromatography on silica gel (*n*-hexane/EtOAc: 9/1) yielded **3pa** (61.0 mg, 53%) as a white solid. **M.p.**: 165–167 ℃. **^{1}H NMR** (400 MHz, $CDCl_3$) δ = 12.25 (s, 1H), 9.07 (d, J = 1.8 Hz, 1H), 8.73 (dd, J = 4.2, 1.7 Hz, 1H), 8.71 (ddd, J = 4.8, 1.9, 0.9 Hz, 1H), 8.43 (d, J = 8.2 Hz, 1H), 8.11 (dd, J = 8.3, 1.7 Hz, 1H), 7.77 (d, J = 1.5 Hz, 1H), 7.74 (dd, J = 7.9, 6.1 Hz, 1H), 7.72 (d, J = 1.5 Hz, 1H), 7.70 (t, J = 1.2 Hz, 1H), 7.64 (dd, J = 8.2, 1.6 Hz, 1H), 7.61 (dt, J = 8.0, 1.1 Hz, 1H), 7.43 (dd, J = 8.2, 4.2 Hz, 1H), 7.38–7.32 (m, 3H), 7.31–7.21 (m, 2H), 5.90 (q, J = 6.4 Hz, 1H), 2.63 (d, J = 0.9 Hz, 3H), 2.03 (d, J = 6.4 Hz, 3H). **^{13}C NMR** (100 MHz, $CDCl_3$) δ = 163.9 (C_q), 156.4 (C_q), 156.1 (C_q),

149.7 (CH), 146.9 (CH), 143.5 (C_q), 142.4 (C_q), 138.1 (C_q), 137.7 (C_q), 136.8 (CH), 135.5 (CH), 135.5 (C_q), 133.0 (CH), 128.8 (CH), 128.2 (C_q), 127.8 (CH), 125.9 (CH), 123.3 (C_q), 122.7 (CH), 121.5 (CH), 120.8 (CH), 120.6 (CH), 119.9 (CH), 119.3 (CH), 112.8 (CH), 77.9 (CH), 24.8 (CH_3), 22.4 (CH_3). **IR** (ATR): 3230, 2909, 1655, 1525, 1438, 1422, 1199, 763, 732, 700 cm^{-1}. **MS** (ESI) *m/z* (relative intensity): 482 (35) $[M+Na]^+$, 460 (100) $[M+H]^+$, 356 (45). **HR-MS** (ESI) *m/z* calcd for $C_{30}H_{26}N_3O_2$ $[M+H]^+$: 460.2020, found: 460.2020.

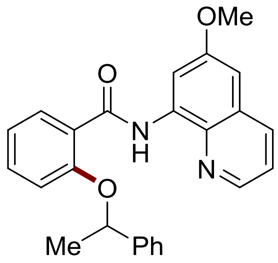

***N*-(6-Methoxyquinolin-8-yl)-2-(1-phenylethoxy)benzamide (3qa)**

The general procedure was followed using benzamide **1q** (0.25 mmol, 69.5 mg) and 1-phenylethan-1-ol (**2a**) (305 mg, 2.5 mmol). Purification by column chromatography on silica gel (*n*-hexane/EtOAc: 12/1→6/1) yielded **3qa** (66.5 mg, 67%) as a white solid and **1q** (13.0 mg, 19%). **M.p.**: 150–151 ℃. **^{1}H NMR** (400 MHz, $CDCl_3$) δ = 12.18 (s, 1H), 8.93 (d, J = 2.8 Hz, 1H), 8.63 (dd, J = 4.2, 1.7 Hz, 1H), 8.32 (dd, J = 7.9, 1.9 Hz, 1H), 8.08 (dd, J = 8.3, 1.7 Hz, 1H), 7.65–7.57 (m, 2H), 7.41 (dd, J = 8.3, 4.2 Hz, 1H), 7.39–7.33 (m, 2H), 7.33–7.27 (m, 2H), 7.05 (ddd, J = 8.1, 7.3, 1.0 Hz, 1H), 6.94 (dd, J = 8.5, 1.0 Hz, 1H), 6.88 (d, J = 2.8 Hz, 1H), 5.65 (q, J = 6.5 Hz, 1H), 4.00 (s, 3H), 1.99 (d, J = 6.5 Hz, 3H). **^{13}C NMR** (100 MHz, $CDCl_3$) δ = 164.2 (C_q), 158.6 (C_q), 156.0 (C_q), 145.1 (CH), 142.4 (C_q), 136.8 (C_q), 135.8 (C_q), 134.9 (CH), 132.8 (CH), 132.5 (CH), 129.0 (C_q), 128.8 (CH), 127.7 (CH), 125.6 (CH), 122.9 (C_q), 121.9 (CH), 121.0 (CH), 114.0 (CH), 109.9 (CH), 100.0 (CH), 77.9 (CH), 55.6 (CH_3), 25.0 (CH_3). **IR** (ATR): 3230, 1656, 1520, 1477, 1222, 1157, 1067, 752, 700 cm^{-1}. **MS** (ESI) *m/z* (relative intensity): 421 (35) $[M+Na]^+$, 399 (100) $[M+H]^+$, 295 (50). **HR-MS** (ESI) *m/z* calcd for $C_{25}H_{23}N_2O_3$ $[M+H]^+$: 399.1703, found: 399.1703.

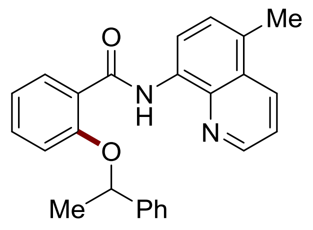

***N*-(5-Methylquinolin-8-yl)-2-(1-phenylethoxy)benzamide (3ra)**

The general procedure was followed using benzamide **1r** (0.25 mmol, 65.5 mg) and 1-phenylethan-1-ol (**2a**) (305 mg, 2.5 mmol). Purification by column chromatography on silica gel (*n*-hexane/EtOAc: 16/1→9/1) yielded **3ra** (57.8 mg, 61%) as a white solid and **1r** (13.9 mg, 21%). **M.p.**: 151–152 ℃. **^{1}H NMR** (300 MHz, $CDCl_3$) δ = 12.19 (s, 1H), 9.06 (d, J = 7.9 Hz, 1H), 8.81 (dd, J = 4.2, 1.6 Hz, 1H), 8.36 (ddd, J = 7.6, 5.9, 1.8 Hz, 2H), 7.67–7.60 (m, 2H), 7.53–7.45 (m, 2H), 7.43–7.25 (m, 4H), 7.10–7.03 (m, 1H), 6.94 (d, J = 8.2 Hz, 1H), 5.66 (q, J = 6.4 Hz, 1H), 2.69 (d, J = 0.8 Hz, 3H), 1.99 (d, J = 6.4 Hz, 3H). **^{13}C NMR** (75 MHz, $CDCl_3$) δ = 164.0 (C_q), 156.0 (C_q), 147.3 (CH), 142.5 (C_q), 139.5 (C_q), 134.3 (C_q), 132.9 (CH), 132.7 (CH), 132.5 (CH), 128.9 (CH), 128.2 (C_q), 127.8 (CH), 127.7 (CH), 127.4 (C_q), 125.7 (CH), 123.2 (C_q), 121.1 (CH), 121.0 (CH), 117.5 (CH), 114.0 (CH), 77.8 (CH), 25.1 (CH_3), 18.3 (CH_3). **IR** (ATR): 3306, 1654, 1520, 1495, 1455, 1225, 753, 701 cm^{-1}. **MS** (ESI) *m/z* (relative intensity): 405 (40) $[M+Na]^+$, 383 (100) $[M+H]^+$, 279 (85), 159 (50), 105 (40). **HR-MS** (ESI) *m/z* calcd for $C_{25}H_{23}N_2O_2$ $[M+H]^+$: 383.1754, found: 383.1755.

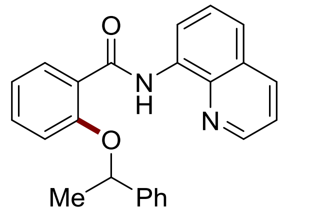

2-(1-Phenylethoxy)-*N*-(quinolin-8-yl)benzamide (3sa)

The general procedure was followed using benzamide **1s** (0.25 mmol, 62.0 mg) and 1-phenylethan-1-ol (**2a**) (305 mg, 2.5 mmol). Purification by column chromatography on silica gel (*n*-hexane/EtOAc: 16/1→9/1) yielded **3sa** (48.9 mg, 53%) as a white

solid and **1s** (23.0 mg, 37%). **M.p.**: 112–114 ℃. **^{1}H NMR** (400 MHz, $CDCl_3$) δ = 12.22 (s, 1H), 9.18 (dd, J = 7.7, 1.4 Hz, 1H), 8.81 (dd, J = 4.2, 1.7 Hz, 1H), 8.35 (dd, J = 7.8, 1.9 Hz, 1H), 8.21 (dd, J = 8.2, 1.7 Hz, 1H), 7.68–7.61 (m, 3H), 7.58 (dd, J = 8.3, 1.4 Hz, 1H), 7.47 (dd, J = 8.2, 4.2 Hz, 1H), 7.42–7.32 (m, 2H), 7.34–7.27 (m, 2H), 7.06 (ddd, J = 8.0, 7.3, 1.0 Hz, 1H), 6.94 (dd, J = 8.5, 1.0 Hz, 1H), 5.66 (q, J = 6.5 Hz, 1H), 1.99 (d, J = 6.5 Hz, 3H). **^{13}C NMR** (100 MHz, $CDCl_3$) δ = 164.2 (C_q), 156.0 (C_q), 147.8 (CH), 142.5 (C_q), 139.3 (C_q), 136.3 (CH), 135.9 (C_q), 132.8 (CH), 132.5 (CH), 128.9 (CH), 128.2 (C_q), 127.8 (CH), 127.6 (CH), 125.7 (CH), 123.1 (C_q), 121.6 (CH), 121.5 (CH), 121.0 (CH), 117.8 (CH), 114.1 (CH), 77.9 (CH), 25.1 (CH_3). **IR** (ATR): 3304, 1655, 1519, 1324, 1221, 1066, 790, 750, 699 cm^{-1}. **MS** (ESI) *m/z* (relative intensity): 391 (45) $[M+Na]^+$, 369 (90) $[M+H]^+$, 265 (100), 145 (70), 105 (30). **HR-MS** (ESI) *m/z* calcd for $C_{24}H_{21}N_2O_2$ $[M+H]^+$: 369.1598, found: 369.1597.

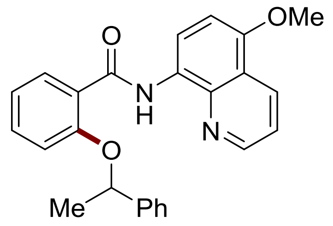

***N*-(5-Methoxyquinolin-8-yl)-2-(1-phenylethoxy)benzamide (3ta)**

The general procedure was followed using benzamide **1t** (0.25 mmol, 69.5 mg) and 1-phenylethan-1-ol (**2a**) (305 mg, 2.5 mmol). Purification by column chromatography on silica gel (*n*-hexane/EtOAc: 16/1→9/1) yielded **3ta** (27.4 mg, 28%) as a white solid and **1t** (44.6 mg, 64%). **M.p.**: 147–148 ℃. **^{1}H NMR** (400 MHz, $CDCl_3$) δ = 11.99 (s, 1H), 9.05 (d, J = 8.6 Hz, 1H), 8.77 (dd, J = 4.2, 1.7 Hz, 1H), 8.59 (dd, J = 8.4, 1.7 Hz, 1H), 8.30 (dd, J = 7.8, 1.9 Hz, 1H), 7.61–7.55 (m, 2H), 7.42 (dd, J = 8.4, 4.2 Hz, 1H), 7.35–7.29 (m, 2H), 7.31–7.20 (m, 2H), 7.01 (ddd, J = 8.1, 7.2, 1.0 Hz, 1H), 6.94–6.87 (m, 2H), 5.61 (q, J = 6.5 Hz, 1H), 4.01 (s, 3H), 1.95 (d, J = 6.5 Hz, 3H). **^{13}C NMR** (100 MHz, $CDCl_3$) δ = 163.6 (C_q), 155.9 (C_q), 150.3 (C_q), 148.2 (CH), 142.5 (C_q), 139.9 (C_q), 132.5 (CH), 132.4 (CH), 131.1 (CH), 129.3 (C_q), 128.8 (CH), 127.7 (CH), 125.6 (CH), 123.1 (C_q), 121.0 (CH), 120.6 (CH), 120.5 (C_q), 117.9 (CH),

114.0 (CH), 104.6 (CH), 77.8 (CH), 55.8 (CH_3), 25.1 (CH_3). **IR** (ATR): 3311, 1644, 1526, 1269, 1222, 1089, 751, 701 cm^{-1}. **MS** (ESI) *m/z* (relative intensity): 421 (30) $[M+Na]^+$, 399 (100) $[M+H]^+$, 295 (80), 175 (40), 105 (10). **HR-MS** (ESI) *m/z* calcd for $C_{25}H_{23}N_2O_3$ $[M+H]^+$: 399.1703, found: 399.1704.

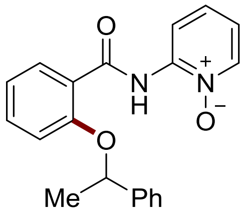

2-[2-(1-Phenylethoxy)benzamido]pyridine 1-oxide (3xa)

3xa was prepared according to the known method[2b] by using benzamide **1x** (0.2 mmol, 1.0 equiv, 42.8 mg) and 1-phenylethan-1-ol (**2a**) (1.5 mL, 12.4 mmol). Purification by column chromatography on silica gel (CH_2Cl_2/aceton: 10/1) yielded **3xa** (6.7 mg, 10%) as a white solid. **M.p.**: 179–180 ℃. **^{1}H NMR** (400 MHz, $CDCl_3$) δ = 12.39 (s, 1H), 8.77 (d, J = 8.6 Hz, 1H), 8.30 (s, 1H), 8.23 (dd, J = 7.9, 1.9 Hz, 1H), 7.53–7.41 (m, 2H), 7.43–7.26 (m, 4H), 7.23 (t, J = 7.3 Hz, 1H), 6.99 (t, J = 7.6 Hz, 2H), 6.89 (d, J = 8.4 Hz, 1H), 5.58 (q, J = 6.5 Hz, 1H), 1.97 (d, J = 6.5 Hz, 3H). **^{13}C NMR** (100 MHz, $CDCl_3$) δ = 164.1 (C_q), 156.4 (C_q), 145.4 (C_q), 141.8 (C_q), 137.4 (CH), 134.0 (CH), 132.6 (CH), 128.9 (CH), 127.9 (CH), 127.7 (CH), 125.7 (CH), 121.0 (CH), 120.9 (C_q), 118.5 (CH), 115.9 (CH), 114.3 (CH), 78.2 (CH), 24.8 (CH_3). **IR** (ATR): 3214, 1668, 1562, 1504, 1477, 1426, 1234, 752, 701 cm^{-1}. **MS** (ESI) *m/z* (relative intensity): 357 (75) $[M+Na]^+$, 335 (100) $[M+H]^+$, 231 (90). **HR-MS** (ESI) *m/z* calcd for $C_{20}H_{19}N_2O_3$ $[M+H]^+$: 335.1390, found: 335.1395.

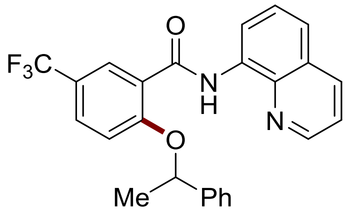

2-(1-Phenylethoxy)-*N*-(quinolin-8-yl)-5-(trifluoromethyl)benzamide (3ya)

3ya was prepared according to the the known method[3e] by using benzamide **1y** (0.5 mmol, 1.0 equiv, 158 mg) and 1-phenylethan-1-ol (**2a**) (305 mg, 2.5 mmol). Purification by column chromatography on silica gel (*n*-hexane/aceton: 16/1→9/1) yielded **3ya** (42.0 mg, 19%) as a white solid. **M.p.**: 118–120 ℃. **^{1}H NMR** (400 MHz, $CDCl_3$) δ = 12.13 (s, 1H), 9.10 (dd, J = 7.5, 1.5 Hz, 1H), 8.78 (dd, J = 4.2, 1.7 Hz, 1H), 8.60 (dd, J = 2.5, 0.8 Hz, 1H), 8.19 (dd, J = 8.3, 1.7 Hz, 1H), 7.66–7.55 (m, 4H), 7.52 (ddd, J = 8.7, 2.5, 0.8 Hz, 1H), 7.45 (dd, J = 8.3, 4.2 Hz, 1H), 7.39–7.32 (m, 2H), 7.31–7.25 (m, 1H), 6.98 (d, J = 8.7 Hz, 1H), 5.67 (q, J = 6.4 Hz, 1H), 2.00 (d, J = 6.4 Hz, 3H). **^{13}C NMR** (100 MHz, $CDCl_3$) δ = 162.6 (C_q), 158.1 (C_q), 147.8 (CH), 141.6 (C_q), 139.2 (C_q), 136.4 (CH), 135.5 (C_q), 130.2 (q, $^{3}J_{C\text{-}F}$ = 3.8 Hz, CH), 129.6 (q, $^{3}J_{C\text{-}F}$ = 3.6 Hz, CH), 129.1 (CH), 128.2 (CH), 128.1 (C_q), 127.6 (CH), 125.5 (CH), 123.9 (d, $^{1}J_{C\text{-}F}$ = 271.7 Hz, C_q), 123.4 (C_q), 123.4 (q, $^{2}J_{C\text{-}F}$ = 33.5 Hz, C_q), 122.0 (CH), 121.6 (CH), 117.9 (CH), 114.3 (CH), 78.6 (CH), 25.1 (CH_3). **^{19}F NMR** (282 MHz, $CDCl_3$) δ = -61.9 (s). **IR** (ATR): 3308, 1662, 1529, 1325, 1271, 1120, 824, 701 cm^{-1}. **MS** (ESI) *m/z* (relative intensity): 459 (10) $[M+Na]^+$, 437 (100) $[M+H]^+$, 333 (35). **HR-MS** (ESI) *m/z* calcd for $C_{25}H_{20}F_3N_2O_2$ $[M+H]^+$: 437.1471, found: 437.1477.

Studies on the potential racemization of 3kq

A racemic sample of ***rac*-3kq** was synthesized following the general procedure using L-Menthol/D-Menthol (1.25 mmol/1.25 mmol). Analysis by chiral HPLC showed that racemization did not take place. HPLC chromatograms were recorded on an Agilent 1290 Infinity instrument using CHIRALPAK ® IA-1 column and *n*-hexane/*i*-PrOH (98/2, 1.0 mL/min, detection at 273 nm, 99% ee)

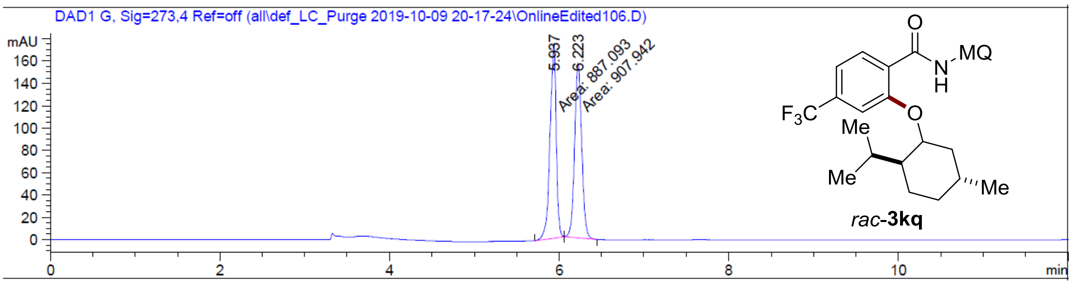

Peak #	RetTime [min]	Type	Width [min]	Area [mAU*s]	Height [mAU]	Area %
1	5.937	MM	0.0847	887.09283	174.49500	49.4193
2	6.223	MM	0.0970	907.94196	156.07780	50.5807

Figure S–1: HPLC-Chromatogram of *rac*-**3kq**.

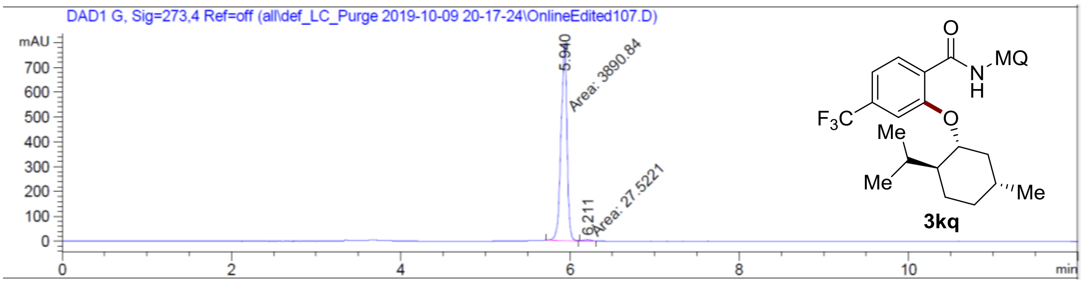

Signal 2: DAD1 G, Sig=273,4 Ref=off

Peak #	RetTime [min]	Type	Width [min]	Area [mAU*s]	Height [mAU]	Area %
1	5.940	MM	0.0814	3890.83911	796.27783	99.2976
2	6.211	MM	0.0993	27.52212	4.62122	0.7024

Figure S–2: HPLC-Chromatogram of **3kq**.

X-Ray crystallographic analysis

The crystal was kept at 99.99 K during data collection. Using Olex2,[4] the structure was solved with the XT[5] structure solution program using Intrinsic Phasing and refined with the XL[6] refinement package using Least Squares minimization.

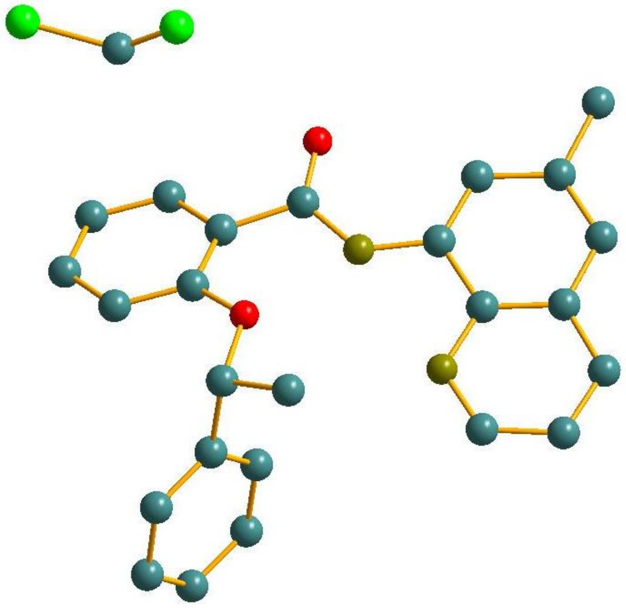

Figure S–3: Molecular structure of **3aa**. The hydrogen atoms are omitted for clarity.

Table S–10: Crystal data and structure refinement for product **3aa**

Compound	**3aa**
CCDC number	1901319
Empirical formula	$C_{25.5}H_{23}ClN_2O_2$
Formula weight	424.91
Temperature/K	100.0
Crystal system	monoclinic
Space group	C2/c
a/Å	22.5773(19)
b/Å	10.7400(8)
c/Å	19.2665(17)
α/°	90
β/°	115.203(3)

γ/°	90
Volume/Å^3	4227.0(6)
Z	8
ρ_{calc}g/cm^3	1.335
μ/mm^{-1}	0.206
F(000)	1784.0
Crystal size/mm^3	0.53 × 0.446 × 0.348
Radiation	MoKα (λ = 0.71073)
2Θ range for data collection/°	4.456 to 61.142
Index ranges	$-32 \leq h \leq 32$, $-15 \leq k \leq 15$, $-27 \leq l \leq 27$
Reflections collected	108498
Independent reflections	6467 [R_{int} = 0.0234, R_{sigma} = 0.0139]
Data/restraints/parameters	6467/22/294
Goodness-of-fit on F^2	1.035
Final R indexes [I>=2σ (I)]	R_1 = 0.0380, wR_2 = 0.1036
Final R indexes [all data]	R_1 = 0.0390, wR_2 = 0.1046
Largest diff. peak/hole / e Å^{-3}	0.44/-0.38

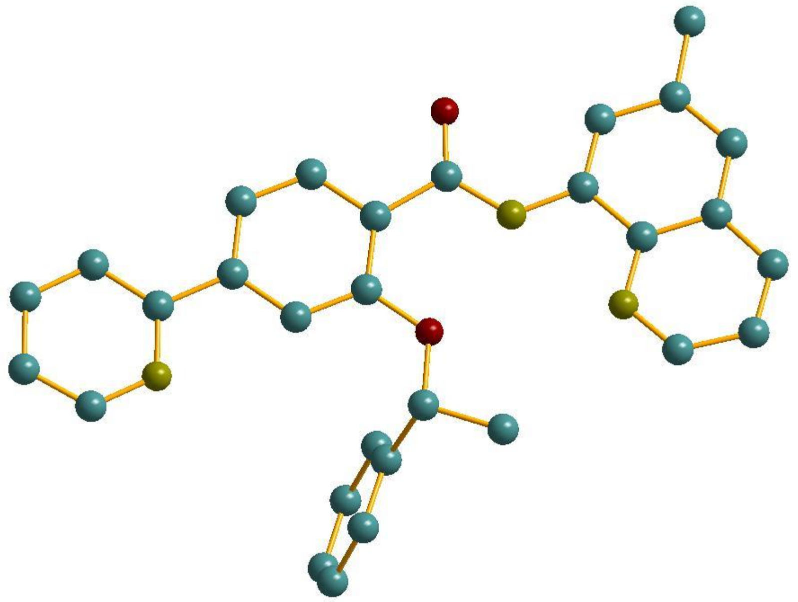

Figure S–4: Molecular structure of **3pa**. The hydrogen atoms are omitted for clarity.

Table S–11: Crystal data and structure refinement for product **3pa**

Compound	**3pa**
CCDC number	1944101
Empirical formula	$C_{30}H_{25}N_3O_2$
Formula weight	459.53
Temperature/K	99.91
Crystal system	triclinic
Space group	P-1
a/Å	5.2539(9)
b/Å	12.722(3)
c/Å	17.383(4)
α/°	102.879(9)
β/°	91.940(13)
γ/°	96.344(9)
Volume/Å^3	1123.7(4)
Z	2
ρ_{calc}g/cm^3	1.358
μ/mm^{-1}	0.086
F(000)	484.0
Crystal size/mm^3	0.227 × 0.219 × 0.059
Radiation	MoKα (λ = 0.71073)
2Θ range for data collection/°	4.816 to 55.886
Index ranges	$-6 \le h \le 6$, $-16 \le k \le 16$, $-22 \le l \le 22$
Reflections collected	51797
Independent reflections	5362 [R_{int} = 0.0268, R_{sigma} = 0.0132]
Data/restraints/parameters	5362/0/321
Goodness-of-fit on F^2	1.045
Final R indexes [I>=2σ (I)]	R_1 = 0.0385, wR_2 = 0.1054
Final R indexes [all data]	R_1 = 0.0430, wR_2 = 0.1102
Largest diff. peak/hole / e Å^{-3}	0.36/-0.22

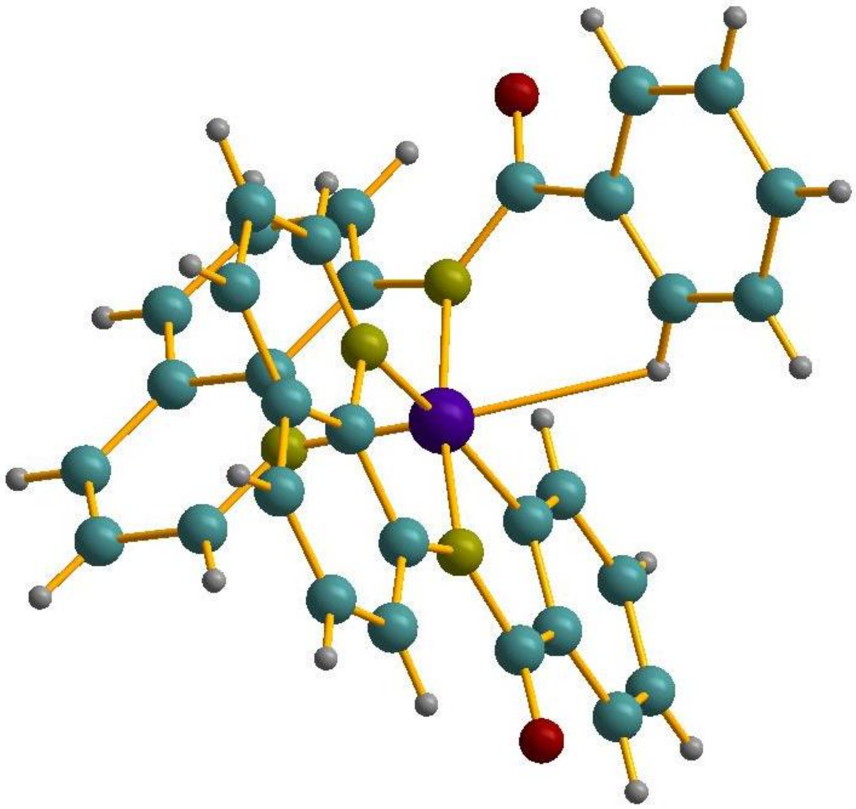

Figure S–5: Molecular structure of **Ni(III)-I**.

Table S-12: Crystal data and structure refinement for **Ni(III)-I**

Compound	**Ni(III)-I**
**CCDC** number	1944102
Empirical formula	$C_{32}H_{21}N_4NiO_2$
Formula weight	552.24
Temperature/K	100.0
Crystal system	monoclinic
Space group	$P2_1/c$
a/Å	8.8378(5)
b/Å	8.9552(5)
c/Å	30.7448(19)
α/°	90
β/°	97.987(2)
γ/°	90
Volume/Å^3	2409.7(2)
Z	4

ρ_{calc}g/cm^3	1.522
μ/mm^{-1}	0.846
F(000)	1140.0
Crystal size/mm^3	0.287 × 0.174 × 0.168
Radiation	MoKα (λ = 0.71073)
2Θ range for data collection/ °	5.278 to 61.058
Index ranges	-12 ≤ h ≤ 12, -12 ≤ k ≤ 12, -43 ≤ l ≤ 43
Reflections collected	46448
Independent reflections	7237 [R_{int} = 0.0259, R_{sigma} = 0.0187]
Data/restraints/parameters	7237/0/352
Goodness-of-fit on F^2	1.056
Final R indexes [I>=2σ (I)]	R_1 = 0.0337, wR_2 = 0.0805
Final R indexes [all data]	R_1 = 0.0392, wR_2 = 0.0838
Largest diff. peak/hole / e Å^{-3}	0.52/-0.50

Mechanistic studies

Competition experiment

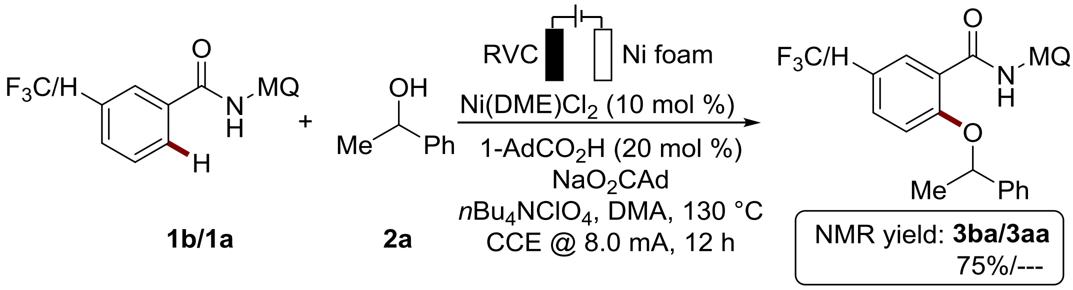

Scheme S–1: Competition experiment between arene **1a** and **1b**.

The general procedure was followed using benzamides **1a** (65.5 mg, 0.25 mmol) and **1b** (82.5 mg, 0.25 mmol) and 1-phenylethan-1-ol (**2a**) (305 mg, 2.5 mmol). Electrolysis was carried out at 130 ℃ and a constant current of 8.0 mA was maintained for 12 h. After cooling to ambient temperature, 1,3,5-trimethoxybenzene (0.25 mmol) was added as the internal standard to determine the ^{1}H NMR yield.

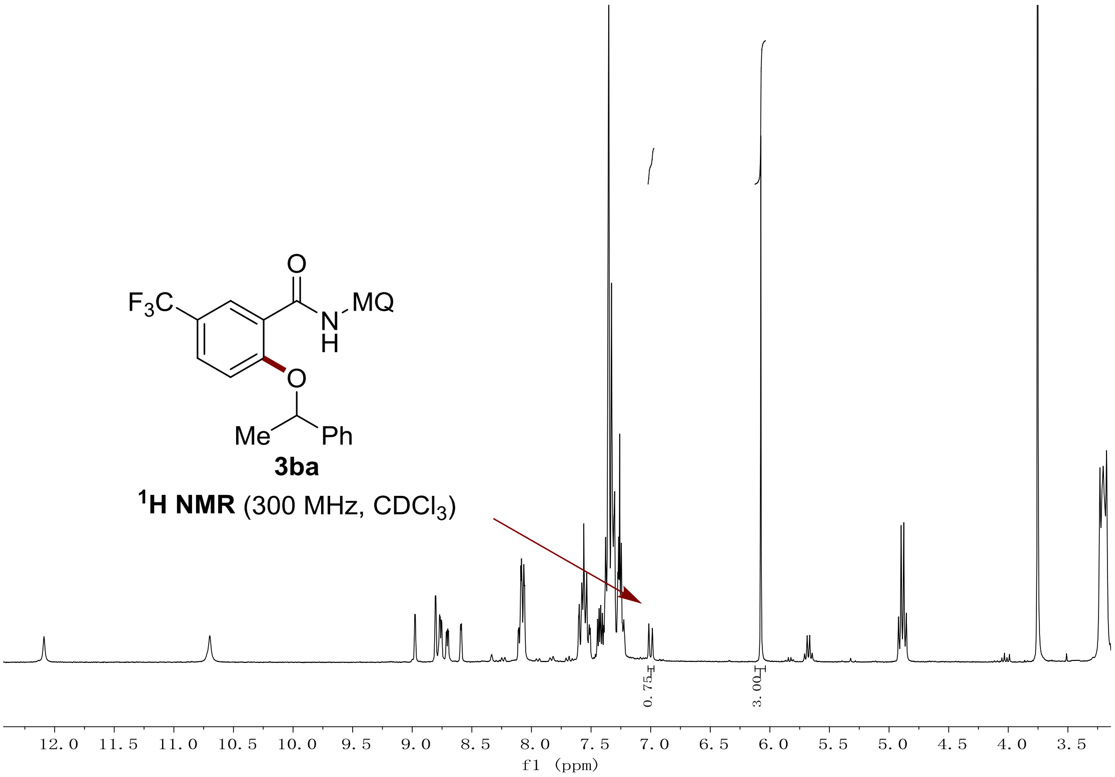

Figure S–6: Competition experiment between arene **1a** and **1b**.

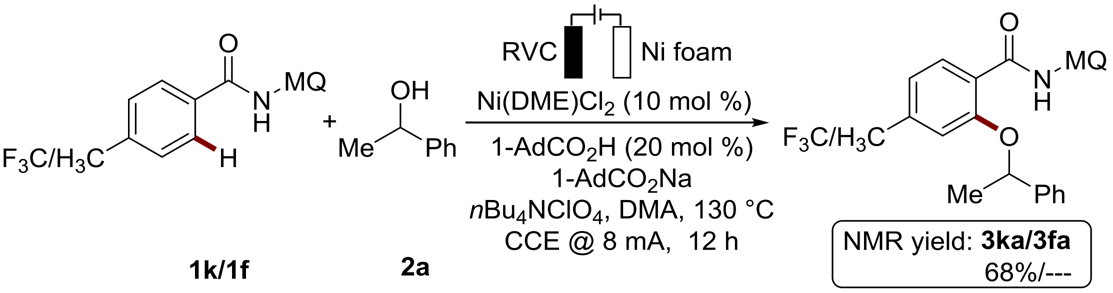

Scheme S–2: Competition experiment between arene **1f** and **1k**.

The general procedure was followed using benzamides **1f** (69.5 mg, 0.25 mmol) and **1k** (82.5 mg, 0.25 mmol) and 1-phenylethan-1-ol (**2a**) (305 mg, 2.5 mmol). Electrolysis was carried out at 130 ℃ and a constant current of 8.0 mA was maintained for 12 h. After cooling to ambient temperature, 1,3,5-trimethoxybenzene (0.25 mmol) was added as the internal standard to determine the ^{1}H NMR yield.

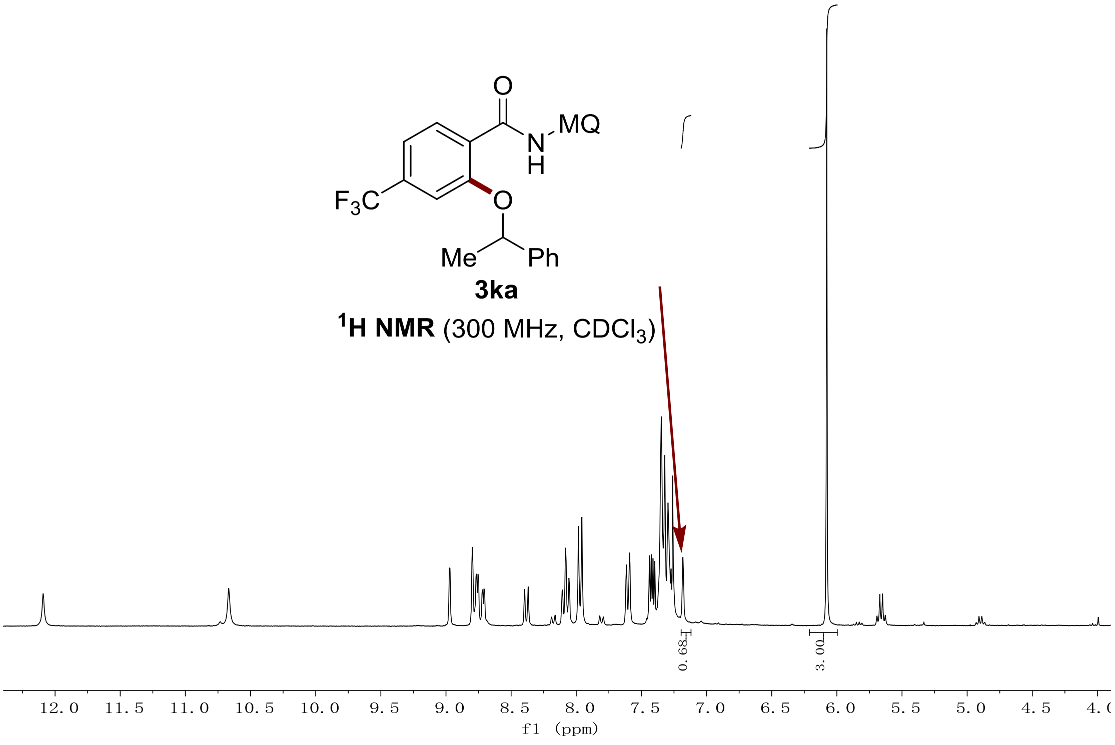

Figure S–7: Competition experiment between arene **1f** and **1k**.

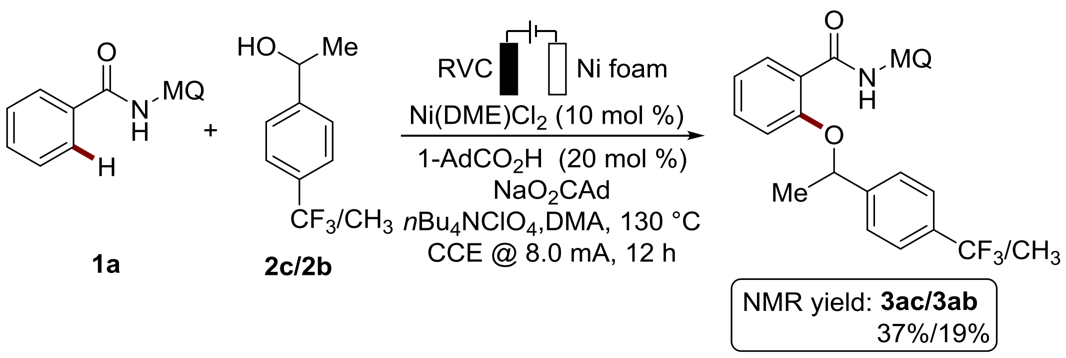

Scheme S–3: Competition experiment between alcohol **2b** and **2c**.

The general procedure was followed using benzamides **1a** (65.5 mg, 0.25 mmol), 1-(*p*-tolyl)ethan-1-ol (**2b**) (170 mg, 1.25 mmol) and 1-[4-(trifluoromethyl)phenyl]-ethan-1-ol (**2c)** (237 mg, 1.25 mmol). Electrolysis was carried out at 130 ℃ and a constant current of 8.0 mA was maintained for 12 h. After cooling to ambient temperature, 1,3,5-trimethoxybenzene (0.25 mmol) was added as the internal standard to determine the ^{1}H NMR yield.

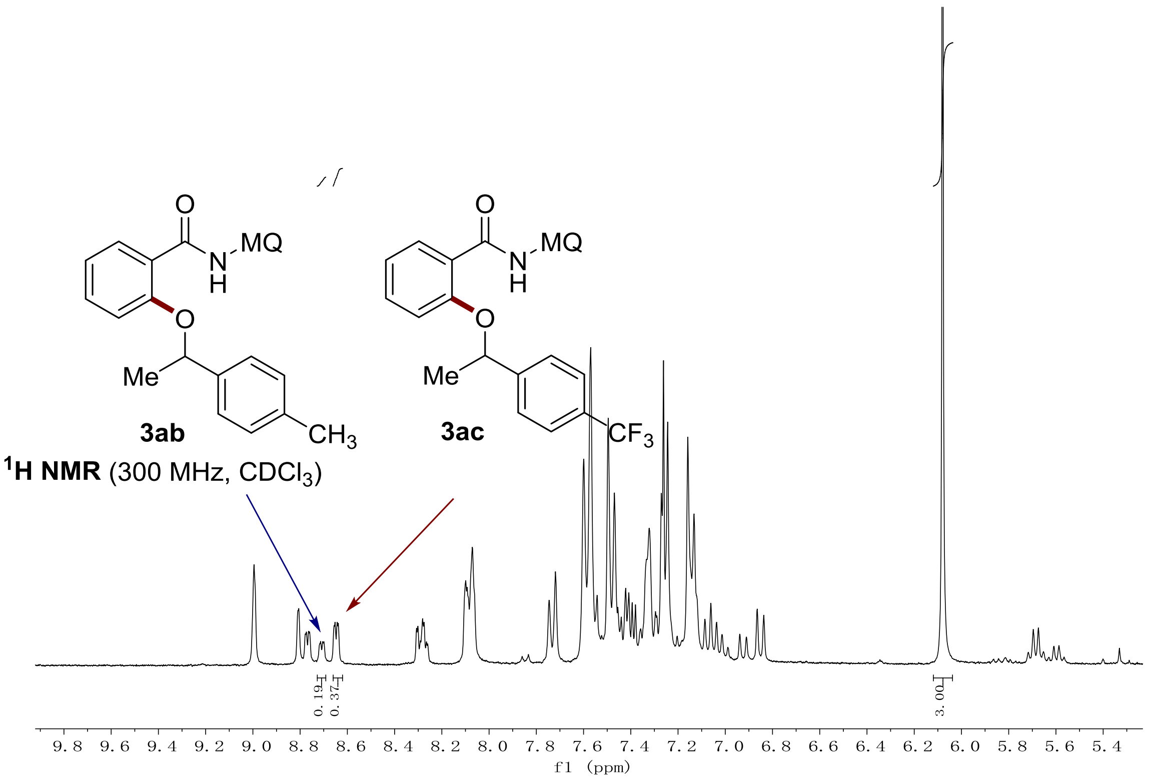

Figure S–8: Competition experiment between alcohol **2b** and **2c**.

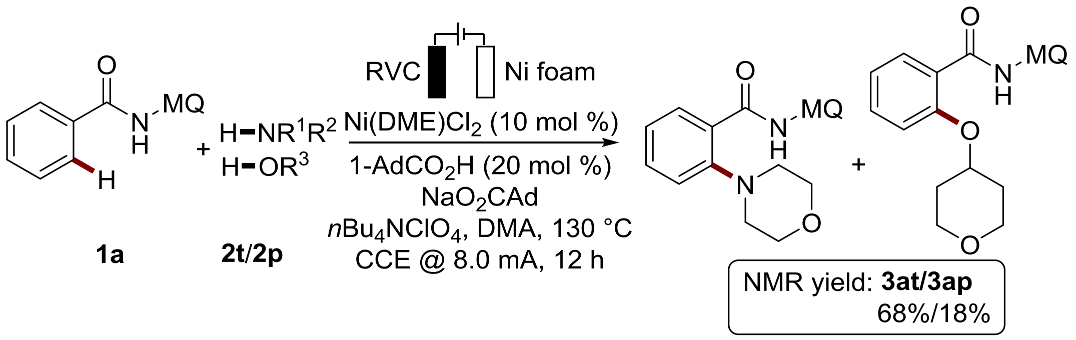

Scheme S–4: Competition experiment between alcohol **2p** and amine **2t**.

The general procedure was followed using benzamide **1a** (65.5 mg, 0.25 mmol), tetrahydro-2*H*-pyran-4-ol (**2p**) (128 mg, 1.25 mmol) and morpholine (**2t**) (109 mg, 1.25 mmol). Electrolysis was carried out at 130 ℃ and a constant current of 8.0 mA was maintained for 12 h. After cooling to ambient temperature, 1,3,5-trimethoxybenzene (0.25 mmol) was added as the internal standard to determine the ^{1}H NMR yield. **3at** and **3ap** were purified by column chromatography on silica gel (*n*-hexane/EtOAc: 10/1→4/1) yielded **3ap** (15.6 mg, 17%) as a white solid and **3at** (52.9 mg, 61%) as a white solid.

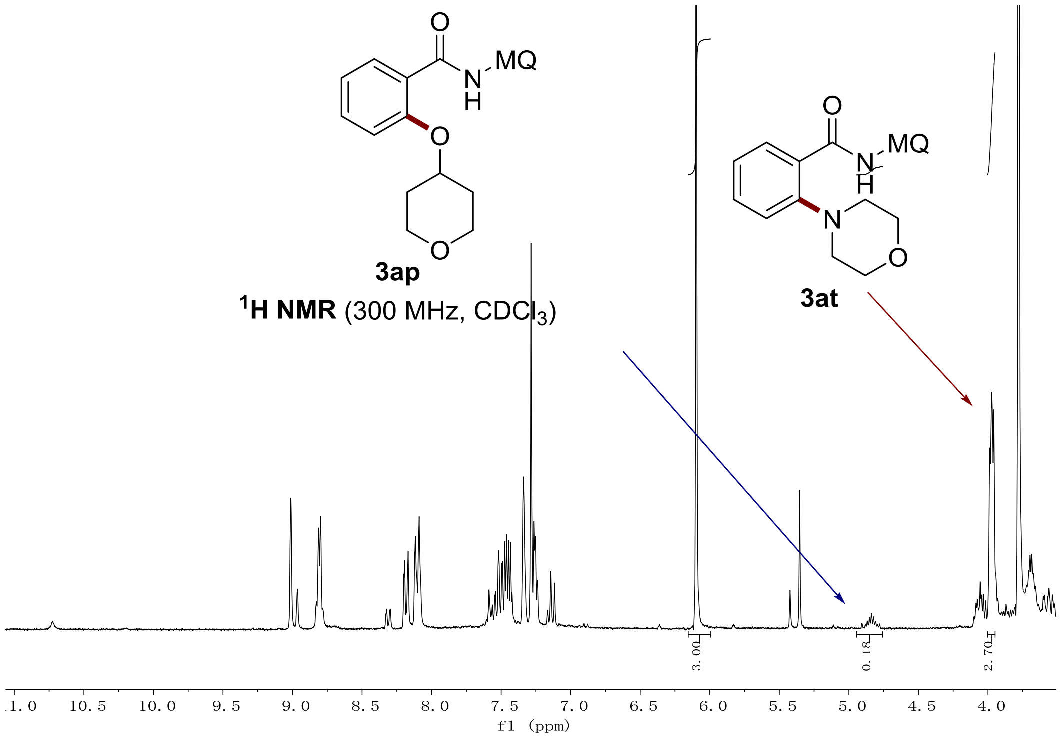

Figure S–9: Competition experiment between alcohol **2p** and amine **2t**.

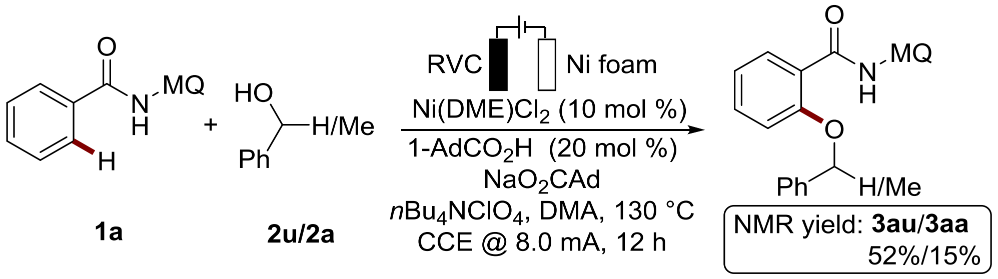

Scheme S–5: Competition experiment between primary alcohol **2u** and secondary alcohol **2a**.

The general procedure was followed using benzamides **1a** (65.5 mg, 0.25 mmol), 1-phenylethan-1-ol (**2a**) (153 mg, 1.25 mmol) and phenylmethanol (**2u)** (135 mg, 1.25 mmol). Electrolysis was carried out at 130 ℃ and a constant current of 8.0 mA was maintained for 12 h. After cooling to ambient temperature, CH_2Br_2 (0.25 mmol) was added as the internal standard to determine the ^{1}H NMR yield.

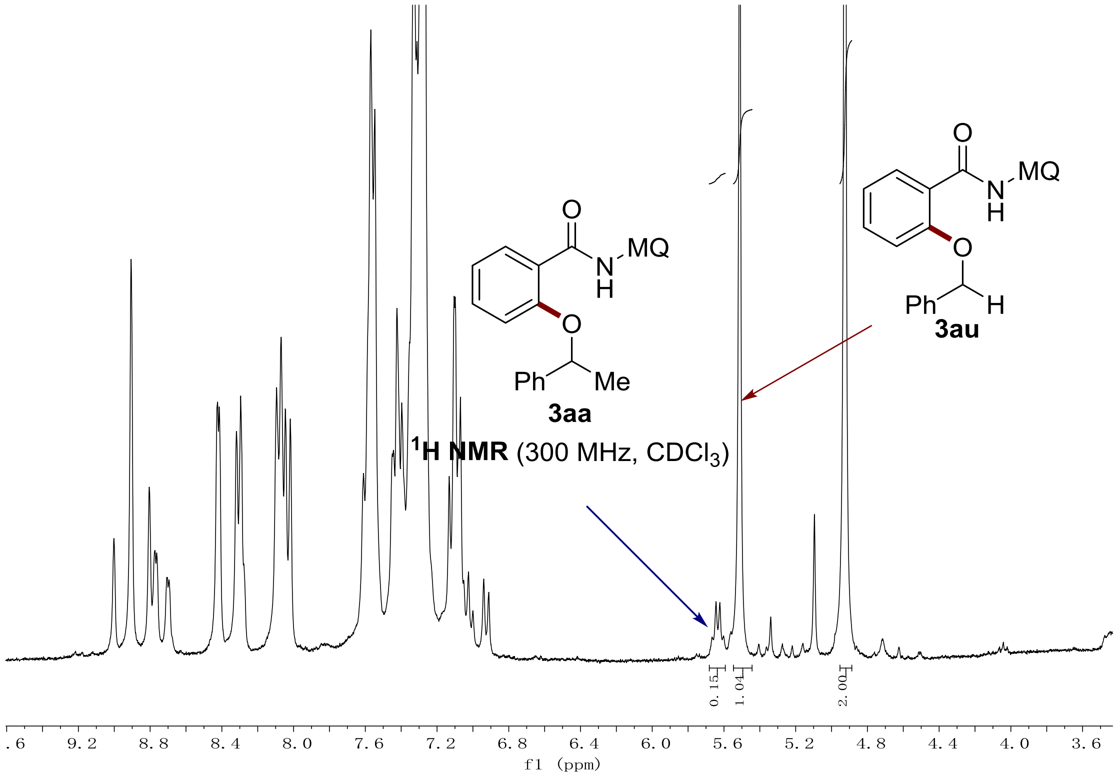

Figure S–10: Competition experiment between secondary alcohol **2a** and primary alcohol **2u**.

Deuteration experiment

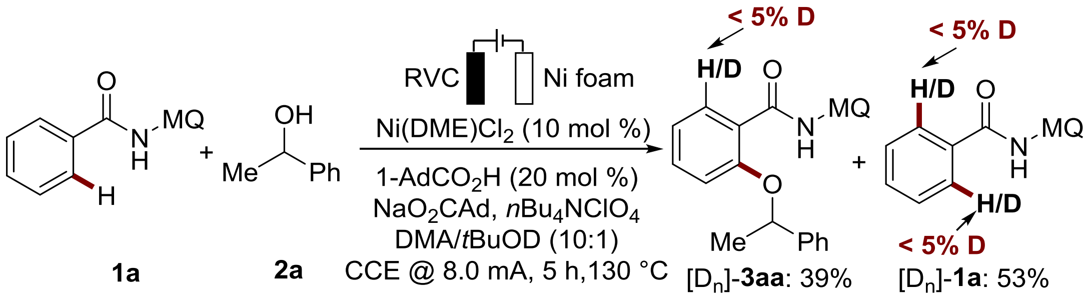

Scheme S–6: Deuteration experiment with *t*BuOD.

The general procedure was followed using benzamide **1a** (65.5 mg, 0.25 mmol) and 1-phenylethan-1-ol (**2a**) (305 mg, 2.5 mmol) using a mixture of DMA and *t*BuOD (3.0/0.3 mL) as solvent with a balloon. Electrolysis was carried out at 130 ℃ and a constant current of 8.0 mA was maintained for 5.0 h. Column chromatography (*n*-hexane/EtOAc: 16/1) yielded $[D]_n$–**3aa** (36.8 mg, 39%) as a white solid and reisolated starting material $[D]_n$–**1a** (34.5 mg, 53%) as a white solid. No deuteration was detected in either compound as determined by ^{1}H NMR spectroscopy.

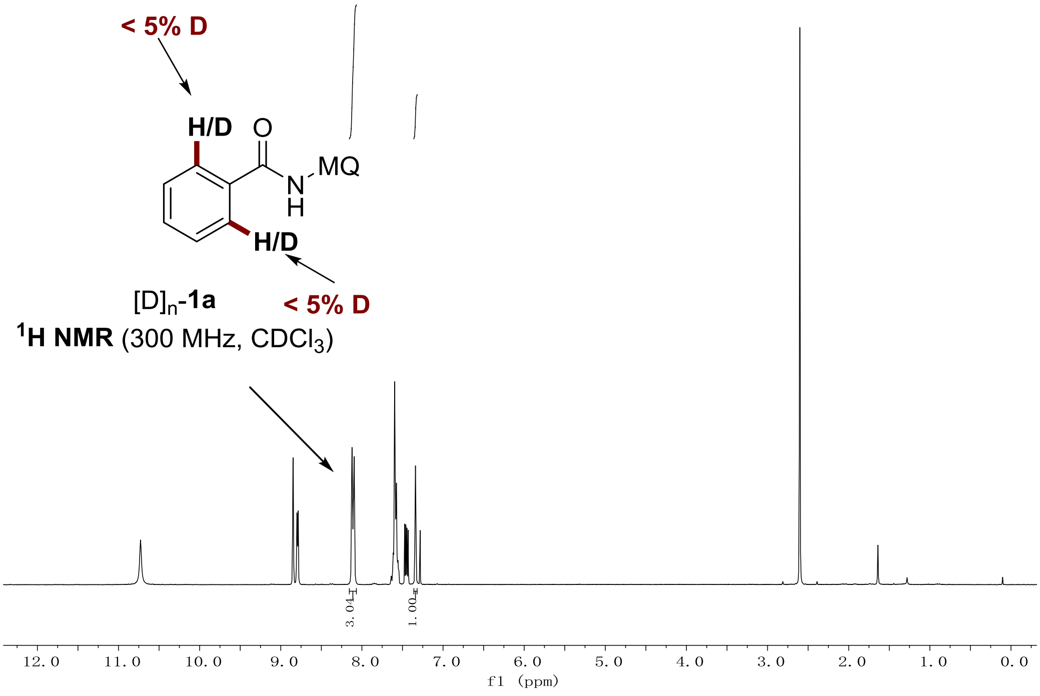

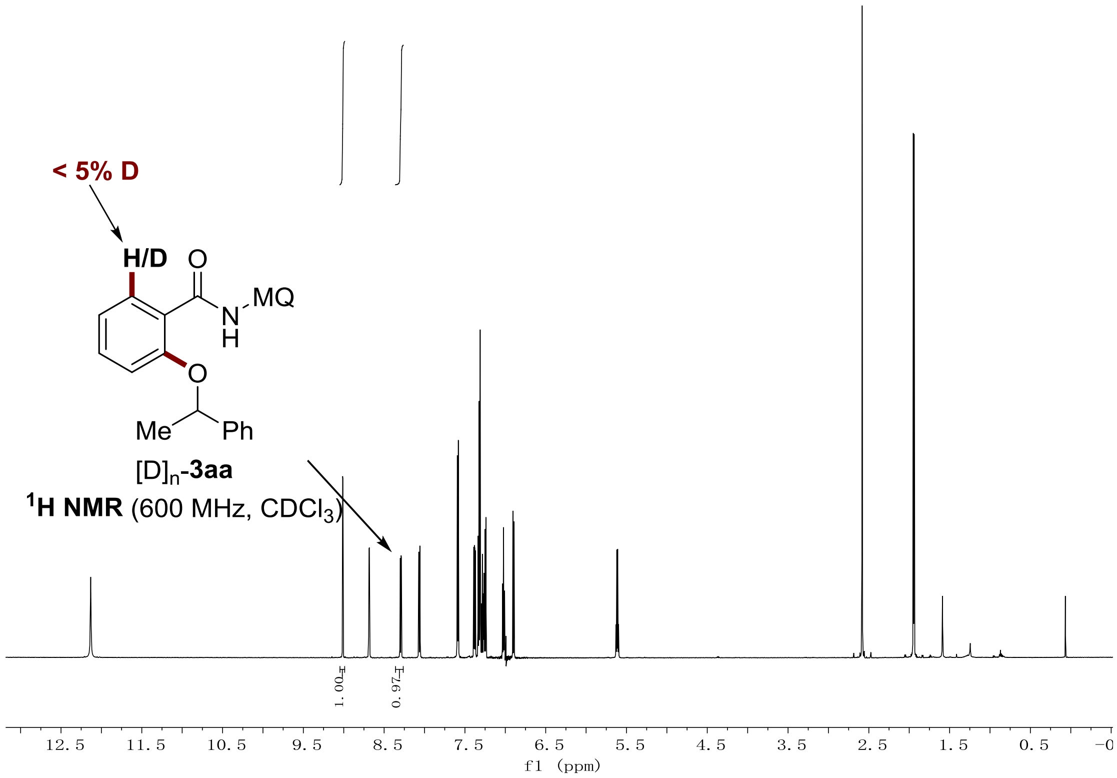

Figure S–11: Deuteration experiment with *t*BuOD.

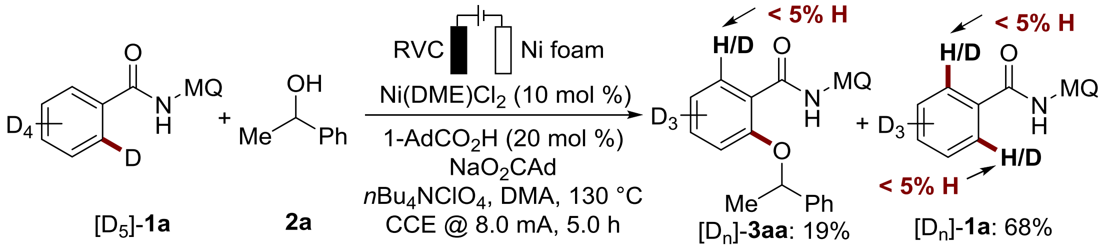

Scheme S–7: Deuteration experiment with [D_5]-**1a**.

The general procedure was followed using benzamide [D_5]-**1a** (66.8 mg, 0.25 mmol) and 1-phenylethan-1-ol (**2a**) (305 mg, 2.5 mmol) in DMA (3.0 mL). Electrolysis was carried out at 130 ℃ and a constant current of 8.0 mA was maintained for 5.0 h. Column chromatography (*n*-hexane/EtOAc 4/1) yielded [D]$_n$–**3aa** (18.5 mg, 19%) as a white solid and reisolated starting material [D]$_n$–**1a** (45.2 mg, 68%) as a white solid.

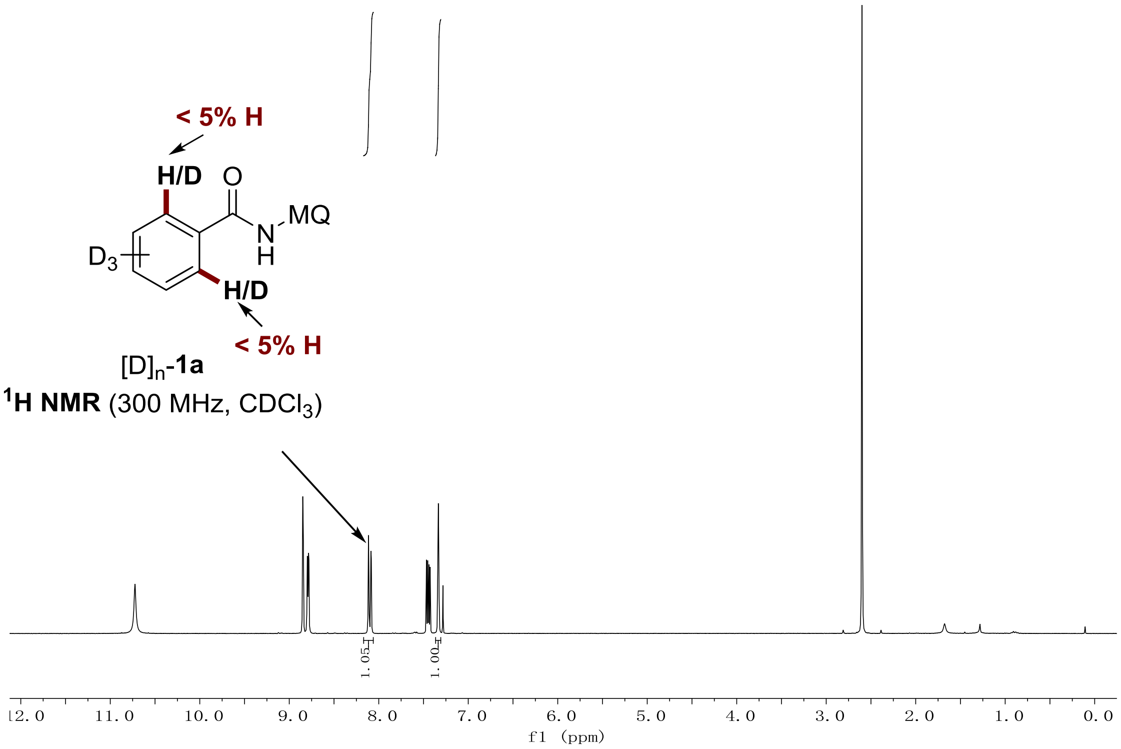

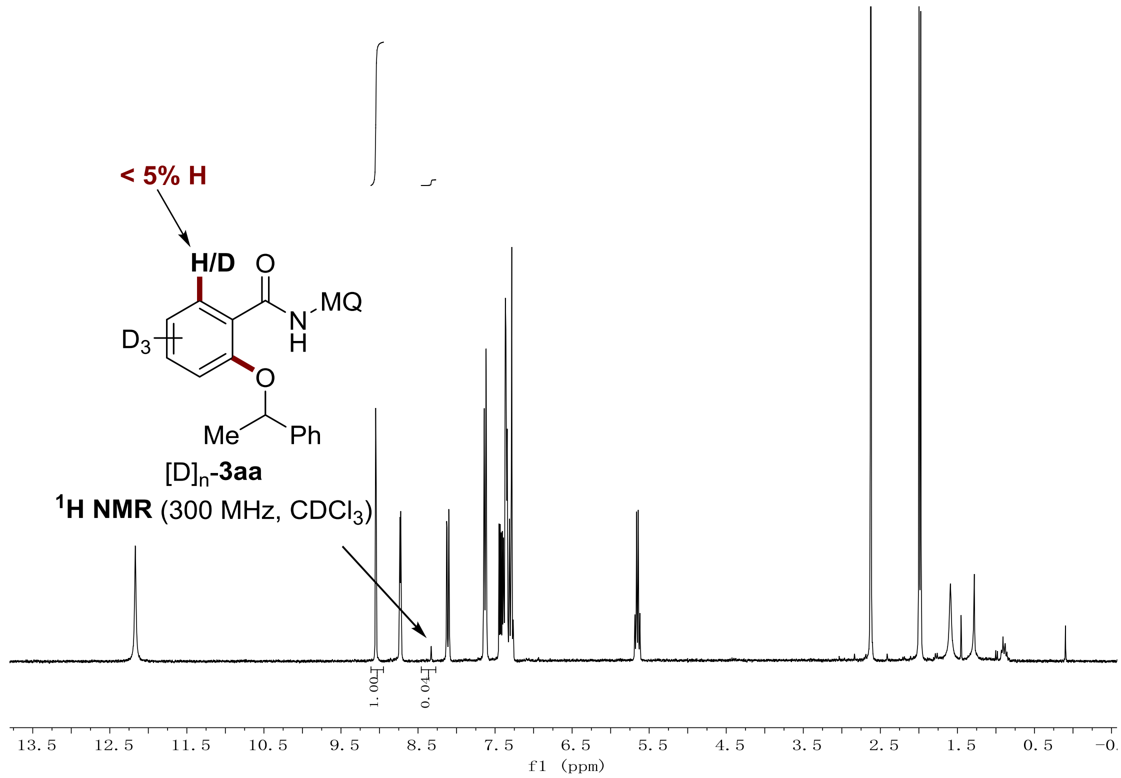

Figure S–12: Deuteration experiment with [D_5]-**1a**.

KIE studies

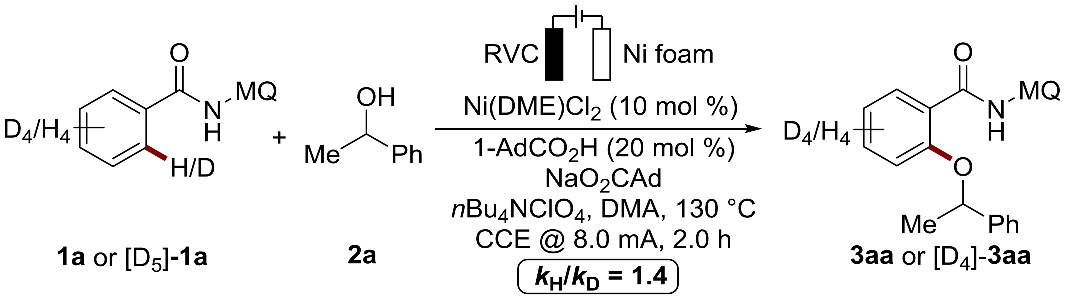

Scheme S–8: KIE studies.

Two parallel reactions were carried out with **1a** (196 mg, 0.75 mmol, 1.0 equiv) or $[D]_5$–**1a** (200 mg, 0.75 mmol, 1.0 equiv) following the general procedure using $Ni(DME)Cl_2$ (15.9 mg, 10 mol %), 1-$AdCO_2H$ (27.0 mg, 20 mol %), NaO_2CAd (151 mg, 0.75 mmol, 1.0 equiv), $n$$Bu_4NClO_4$ (511 mg, 1.50 mmol, 2.0 equiv) and **2a** (0.90 mL, 7.5 mmol, 10.0 equiv) in DMA (6.0 mL). 1,3,5-trimethoxybenzene (0.25 mmol) was added as an internal standard. After 10.0 minutes to reach a stable constant current of 8.0 mA, aliquots of 0.40 mL were removed from the cell every twenty minutes. The mixture was extracted with EtOAc (3.0 mL). After evaporation of the solvent, the crude mixture was analyzed by ^{1}H NMR spectroscopy. The KIE was determined by the analysis of the initial rates.

Table S–13: KIE studies

Time [min]	10	30	50	70	90	110	130
**3aa** [%]	0	2	6	10	13	17	19
$[D_4]$-**3aa** [%]	0	2	4	6	9	11	14

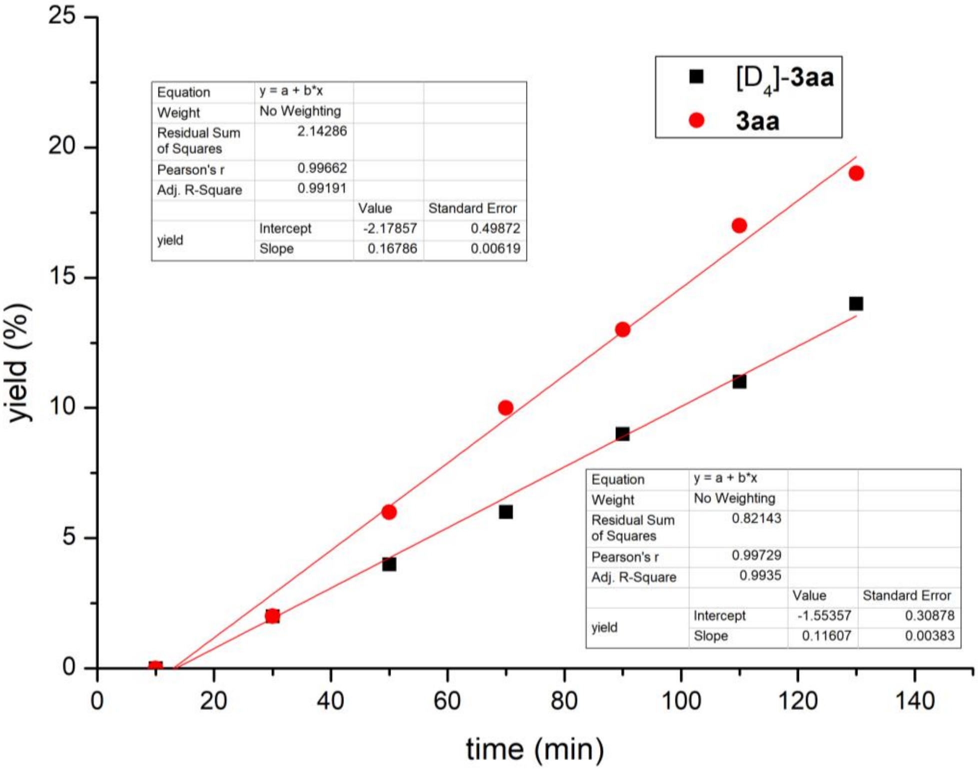

Figure S–13: KIE Studies.

Headspace analysis

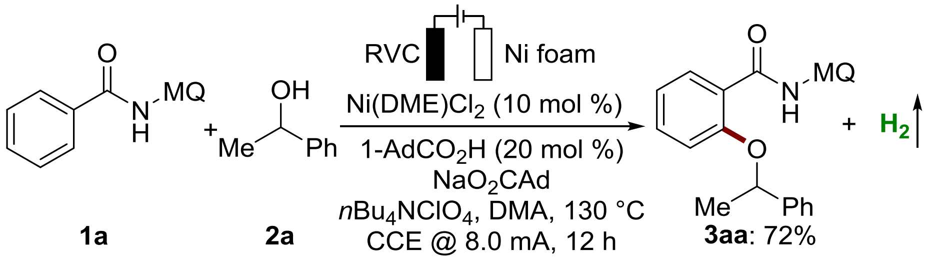

Scheme S–9: Headspace analysis.

Following the general procedure, After 12 h, the gas–phase over the reaction mixture was analyzed by headspace GC analysis using an Agilent 7890B Chromatograph equipped with an Agilent CP–Molsieve 5Å column (length: 25 m, diameter: 0.32 mm, temperature 10 ℃). Helium was used as the carrier gas (1.5 mL/min) and the sample was analyzed by a temperature conductivity detector at 110 ℃. For comparison, also a blank sample of the carrier gas and a pure sample of hydrogen were obtained.

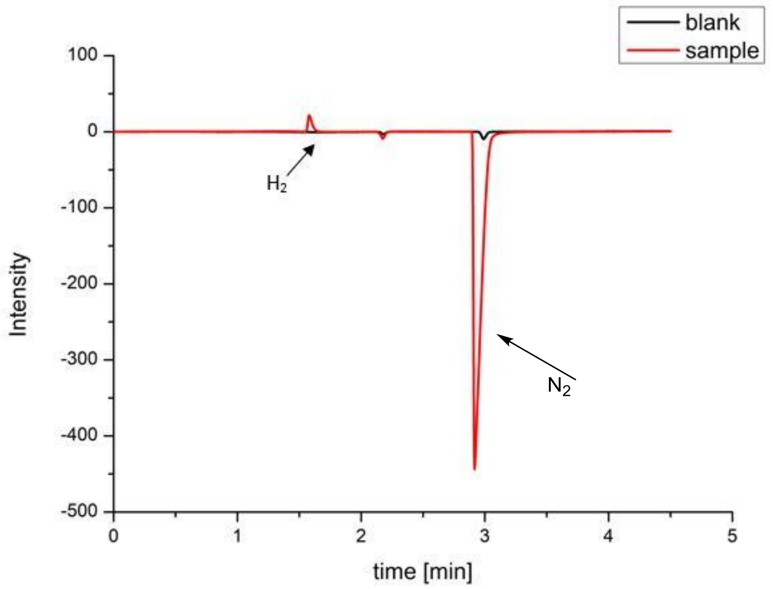

Figure S–14: Headspace analysis of the reaction mixture.

Radical trapping experiment

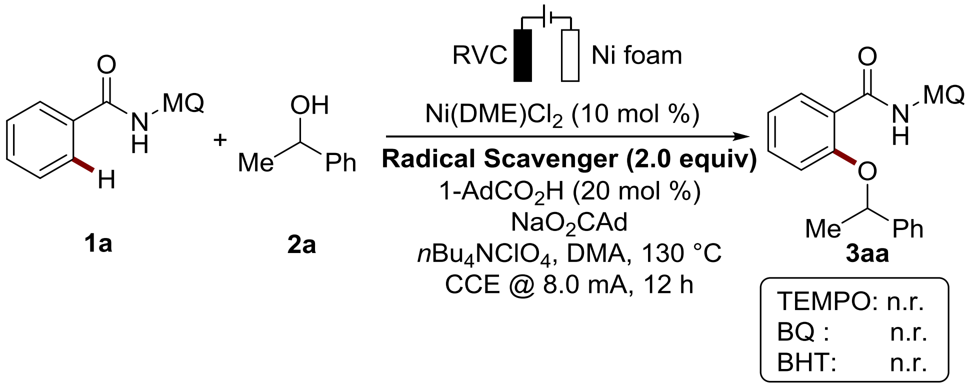

Scheme S–10: Radical trapping experiment.

The electrolysis was carried out in an undivided cell, with a RVC anode (10 mm × 15 mm × 6 mm) and a nickel-foam cathode (10 mm × 15 mm × 1.4 mm). Ni(DME)Cl_2 (5.5 mg, 0.025 mmol, 10 mol %), 1-AdCO_2H (9.0 mg, 0.050 mmol), NaO_2CAd (50.5 mg, 0.25 mmol), *n*Bu_4NClO_4 (0.50 mmol), benzamide **1a** (0.25 mmol, 1.0 equiv) and 2.0 equiv of radical scavenger (TEMPO, benzoquinone or 2,6-di-*tert*-butyl-4-methylphenol) was dissolved in DMA (3.0 mL) and then the 1-phenylethan-1-ol (**2a**) (2.5 mmol) was added. At 130 ℃, electrolysis was started with a constant current of 8.0 mA which was then maintained for 12 h. At ambient temperature, the mixture was transferred to a separating funnel and the electrodes were rinsed with EtOAc (10.0 mL). Then, H_2O (10.0 mL) was added to the reaction mixture and the organic layer was separated. The aqueous layer was extracted with EtOAc (2 × 5.0 mL). After evaporation of the collected organic layer, the mixture was checked by TLC and GC-MS, and no desired products were found.

The synthesis and catalysis analysis of Ni(III)-I

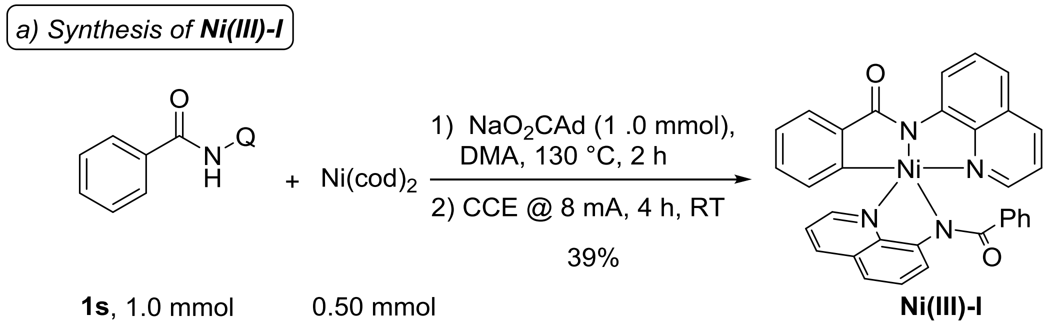

Scheme S–11: Synthesis and its X-ray analysis of **Ni(III)-I**.

According to a reported method,[7] an oven-dried 10 mL undivided electrochemical cell was charged with *N*-(quinolin-8-yl)benzamide (**1s**) (248 mg, 1.0 mmol), Na_2OCAd (202 mg, 1.0 mmol), $Ni(cod)_2$ (138 mg, 0.50 mmol) and dry DMA (3.0 mL) in a glovebox. The reaction vessel was capped and heated in an oil bath at 130 °C for 2.0 h. Upon cooling to room temperature, the cap of the undivided cell was exchanged by a RVC anode and a nickel-foam cathode. Electrolysis was continued with a constant current of 8.0 mA which was then maintained for 4 h at ambient temperature under N_2. Then the reaction mixture was diluted with EtOAc (10.0 mL) and washed with H_2O (10.0 mL). The aqueous layer was extracted with EtOAc (2 x 10.0 mL). The combined organic phase was dried over Na_2SO_4, filtered and evaporated in vacuum. The residue was purified by silica gel column chromatography at low temperature (eluent: *n*-hexane/EtOAc = 4/1 then EtOAc) to afford **Ni(III)-I** (108 mg, 39%) as a dark red solid. The product was crystallized using CH_2Cl_2 and *n*-hexane.

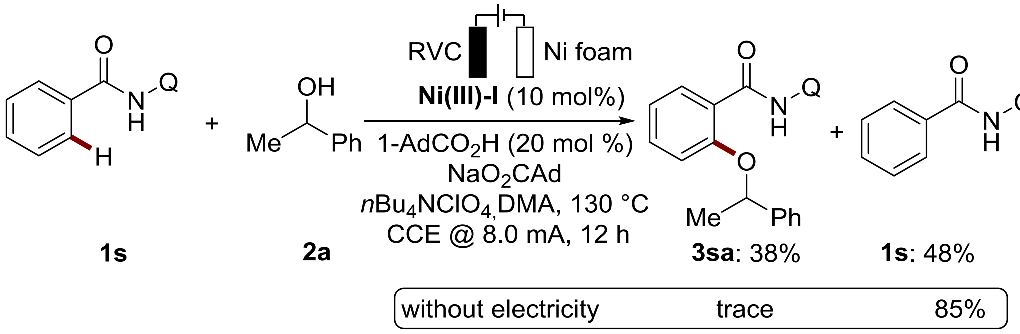

Scheme S–12: **Ni(III)-I** catalyzed reaction of substrate **1s** (w/o electricity).

The electrolysis was carried out in an undivided cell, with a RVC anode (10 mm × 15 mm × 6 mm) and a nickel-foam cathode (10 mm × 15 mm × 1.4 mm). **Ni(III)-I** (27.6 mg, 0.025 mmol, 10 mol %), 1-AdCO_2H (9.0 mg, 0.050 mmol), NaO_2CAd (50.5 mg, 0.25 mmol), *n*Bu_4NClO_4 (0.50 mmol) and benzamide **1s** (62.0 mg, 0.25 mmol, 1.0 equiv) were dissolved in DMA (3.0 mL), and then the 1-phenylethan-1-ol (**2a**) (2.5 mmol) was added. At 130 ℃, electrolysis was started with a constant current of 8.0 mA which was then maintained for 12 h. At ambient temperature, the mixture was transferred to a separating funnel and the electrodes were rinsed with EtOAc (10.0 mL). Then, H_2O (10.0 mL) was added to the reaction mixture and the organic layer was separated. The aqueous layer was extracted with EtOAc (2 × 5.0 mL). Evaporation of the collected organic layer and subsequent column chromatography on silica gel (*n*-hexane /EtOAc: 16/1) yielded the desired product **3sa** (41.6 mg, 0.113 mmol, 38%) and recovered **1s** (36.0 mg, 0.145 mmol, 48%).

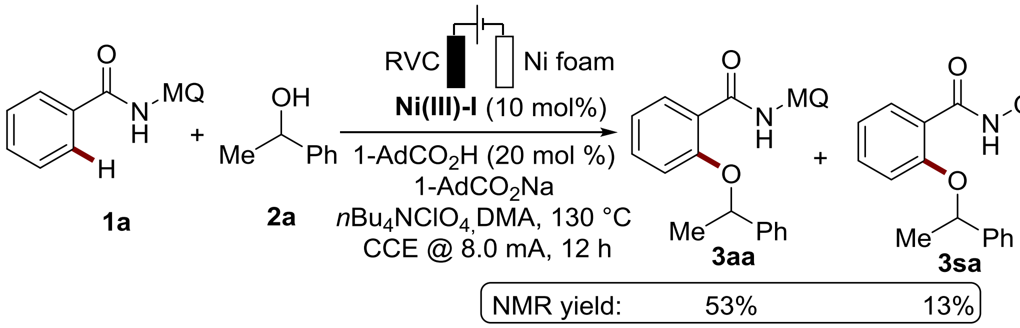

Scheme S–13: **Ni(III)-I** catalyzed reaction of substrate **1a**.

The electrolysis was carried out in an undivided cell, with a RVC anode (10 mm × 15 mm × 6 mm) and a nickel-foam cathode (10 mm × 15 mm × 1.4 mm). **Ni(III)-I** (27.6 mg, 0.025 mmol, 10 mol %), 1-AdCO_2H (9.0 mg, 0.050 mmol), NaO_2CAd (50.5 mg, 0.25 mmol), *n*Bu_4NClO_4 (0.50 mmol) and benzamide **1a** (65.5 mg, 0.25 mmol, 1.0 equiv) were dissolved in DMA (3.0 mL), and then the 1-phenylethan-1-ol (**2a**) (2.5 mmol) was added. At 130 ℃, electrolysis was started with a constant current of 8.0 mA which was then maintained for 12 h. After cooling

to ambient temperature, 1,3,5-trimethoxybenzene (0.25 mmol) was added as the internal standard to determine the ^{1}H NMR yield.

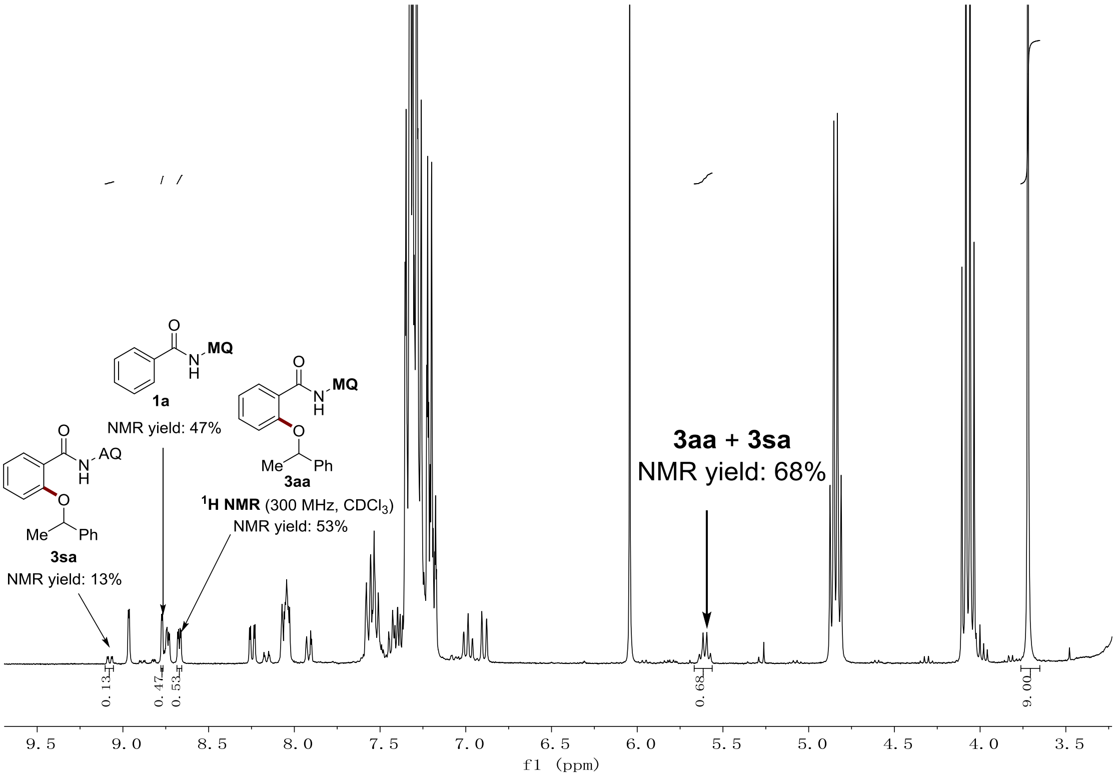

Figure S–15: **Ni(III)-I** catalyzed reaction of substrate **1a**.

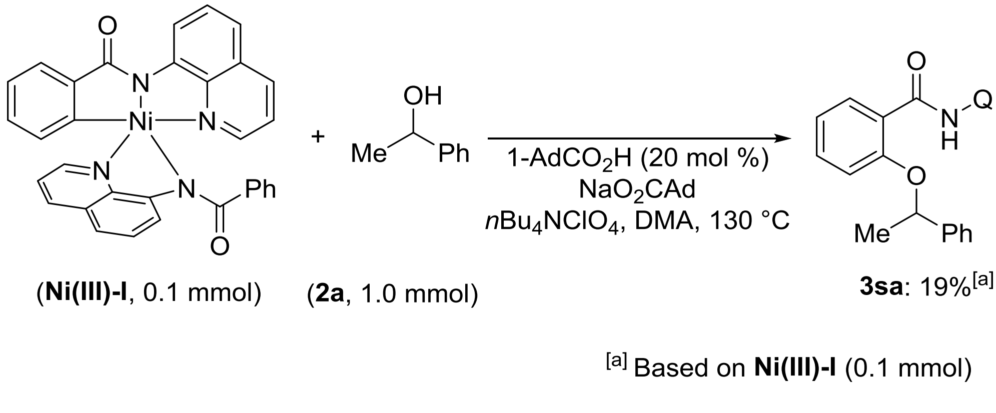

Scheme S–14: **Ni(III)-I** reacted with the alcohol **2a** without electricity.

The electrolysis was carried out in an undivided cell, with a RVC anode (10 mm × 15 mm × 6 mm) and a nickel-foam cathode (10 mm × 15 mm × 1.4 mm). **Ni(III)-I** (55.2 mg, 0.1 mmol), 1-AdCO$_2$H (3.6 mg, 0.02 mmol), NaO$_2$CAd (20.2 mg, 0.1 mmol) and nBu$_4$NClO$_4$ (0.20 mmol) were dissolved in DMA (1.5 mL), and then

the 1-phenylethan-1-ol (**2a**) (1.0 mmol) was added. Then the reaction was carried out without electricity at 130 ℃ for 12 h. After cooling to ambient temperature, 1,3,5-trimethoxybenzene (0.1 mmol) was added as the internal standard to determine the ^{1}H NMR yield.

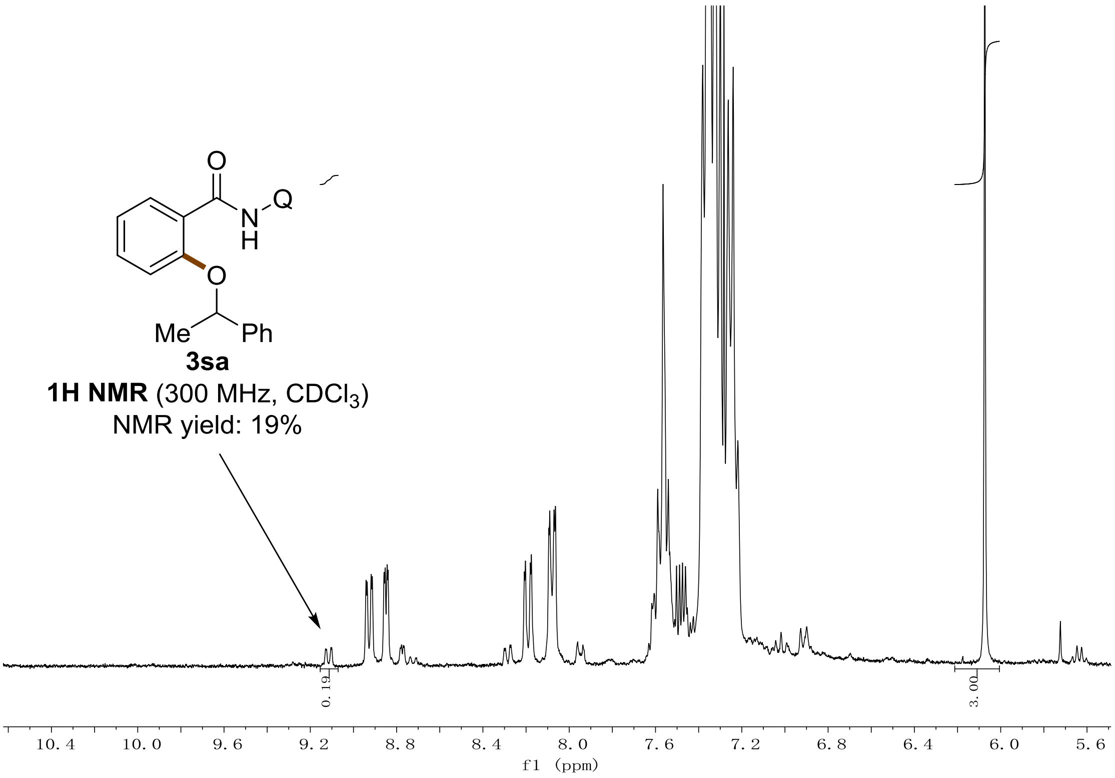

Figure S–16: **Ni(III)-I** reacted with the alcohol **2a** without electricity.

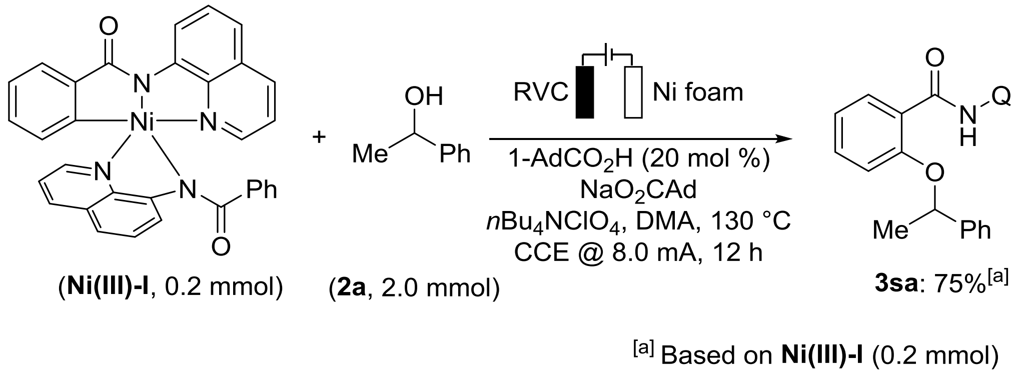

Scheme S–15: **Ni(III)-I** reacted with the alcohol **2a** with electricity.

The electrolysis was carried out in an undivided cell, with a RVC anode (10 mm × 15 mm × 6 mm) and a nickel-foam cathode (10 mm × 15 mm × 1.4 mm). **Ni(III)-I**

(110.4 mg, 0.2 mmol), 1-AdCO_2H (7.2 mg, 0.04 mmol), NaO_2CAd (40.4 mg, 0.2 mmol) and *n*Bu_4NClO_4 (0.5 mmol) were dissolved in DMA (3.0 mL), and then the 1-phenylethan-1-ol (**2a**) (2.0 mmol) was added. At 130 ℃, electrolysis was started with a constant current of 8.0 mA which was then maintained for 12 h. After cooling to ambient temperature, 1,3,5-trimethoxybenzene (0.2 mmol) was added as the internal standard to determine the ^{1}H NMR yield.

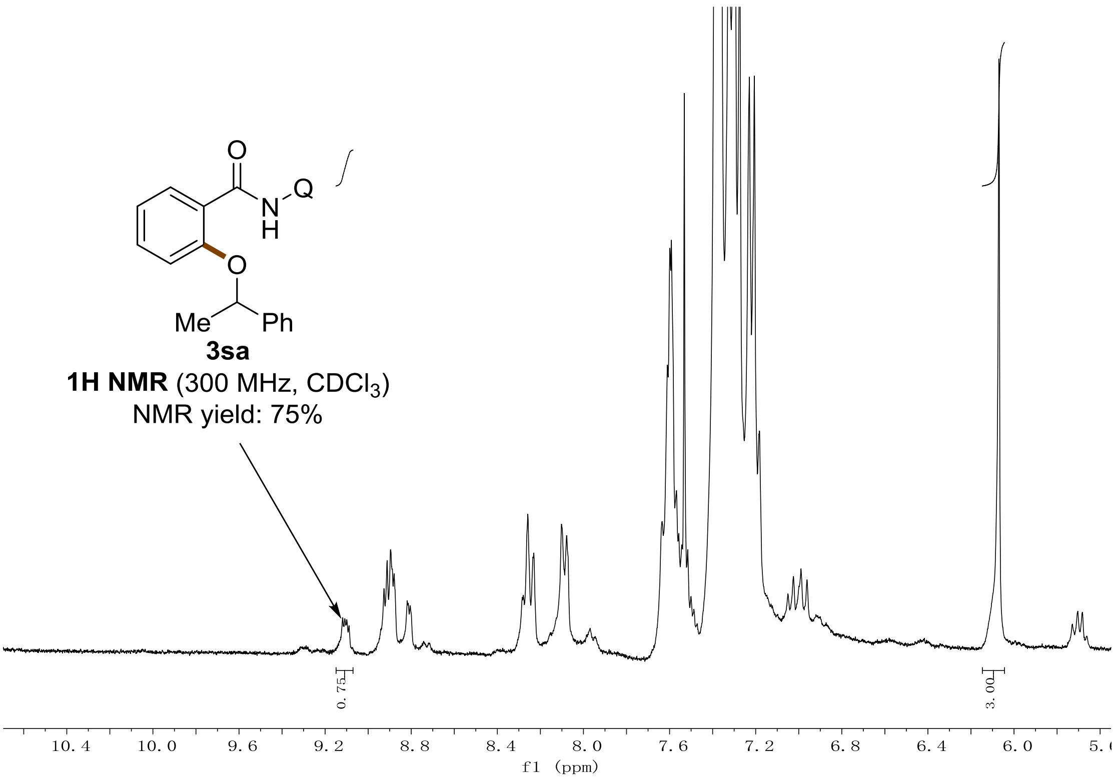

Figure S–17: **Ni(III)-I** reacted with the alcohol **2a** with electricity.

Switch off-on experiment

"Switch off-on" reactions were carried out with **1a** (196 mg, 0.75 mmol, 1.0 equiv) following the procedure **A** using Ni(DME)Cl_2 (15.9 mg, 10 mol %), 1-AdCO_2H (27.0 mg, 20 mol %), NaO_2CAd (152 mg, 0.75 mmol, 1.0 equiv), *n*Bu_4NClO_4 (512 mg, 1.5 mmol, 2.0 equiv) and 1-phenylethan-1-ol (**2a**) (0.90 mL, 7.5 mmol, 10.0 equiv) in DMA (6.0 mL). 1,3,5-trimethoxybenzene (0.25 mmol) was added as an internal standard. Aliquots of 0.40 mL were removed from the cell every 1.5 hours. The mixture was extracted with EtOAc (3.0 mL). After evaporation of solvent, the crude mixture was analyzed by ^{1}H NMR spectroscopy.

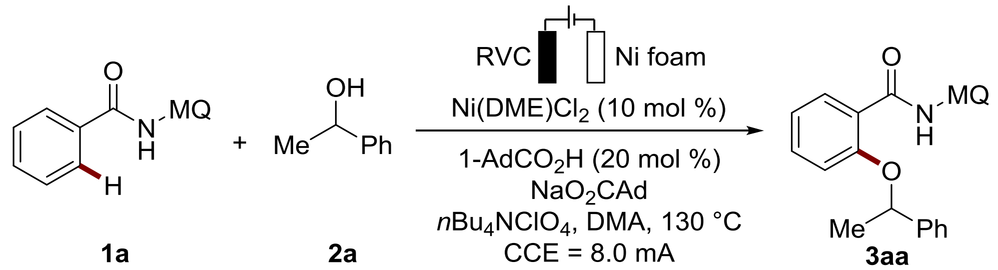

Time [h]	1.5	3.0	4.5	6.0	7.5	9.0	10. 5
**1a** [%]	100	86	87	77	77	68	67
**3aa** [%]	0	5	5	18	18	28	28

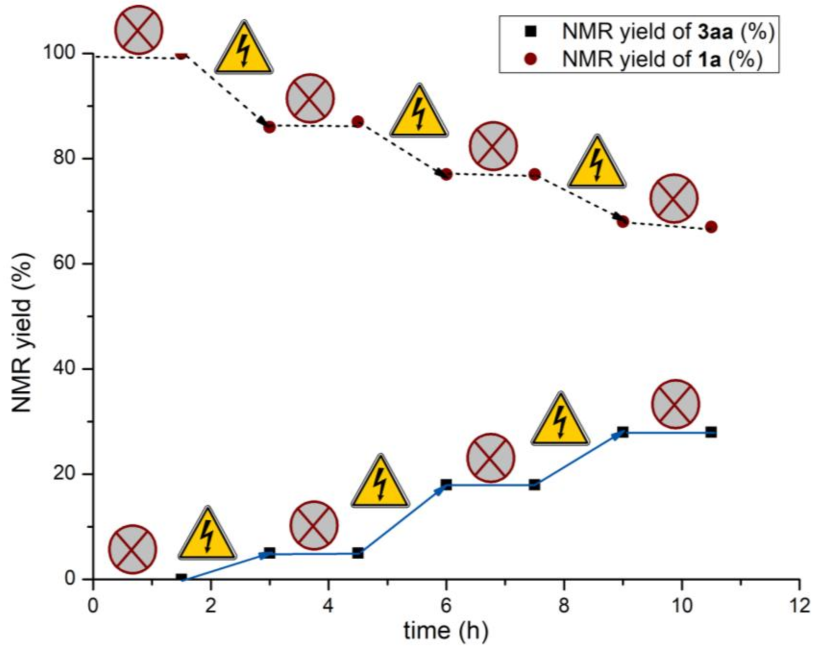

Figure S–18: Switch off-on experiment.

DFT calculation details

All molecular geometries were optimized at PBE0[8a] level in combined with def2-SVP basis set[8b] for all atoms, considering also Grimme's DFT empirical dispersion correction with the Becke-Jonson (D3BJ) damping.[8c, 8d] Optimized minima and transition states (TSs) were verified by harmonic vibrational analysis to have no and one proper imaginary frequency, respectively. To refine the calculated energy, single point calculation with larger basis set were then done based on these optimized structures, by using PBE0 functional with def2-TZVP basis set.[8b] The solvent effect was modeled in these single point calculations by employing SMD continuum solvation model,[8e] taking *N*,*N*-dimethylacetamide as the solvent for each reaction. The reported free energies in this work were based on the electronic energy calculated at the PBE0/def2-TZVP level, including the Gibbs free energy thermal correction obtained from vibrational analysis at the corresponding experimental reaction temperature (130 ℃). The net atomic charge on N atoms were obtained by Mulliken population analysis.[8f] All DFT geometry optimizations and single point calculations were performed with Gaussian 16 program.[8g]

1) Calculation of electron density of *N*,*N*-coordinate atoms from modified 8-Aminoquinoline **(PBE0 Method)**:

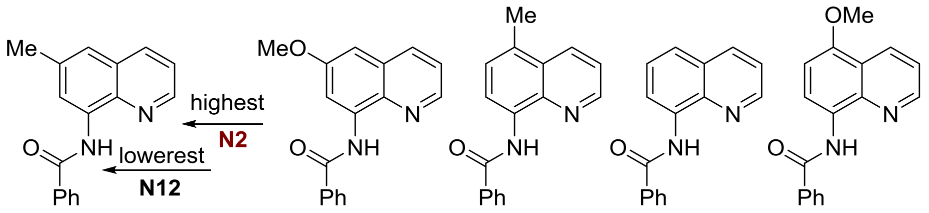

	1a	1q	1r	1s	1u
**N2**	**-0.331128**	-0.331060	-0.329771	-0.328400	-0.327368
**N12**	**-0.147543**	-0.148628	-0.153100	-0.152014	-0.155286

Figure S–19: Net atomic charges of N2 and N12 atom.

2) DFT calculated electron-shift diagram and relative energies reaction profiles

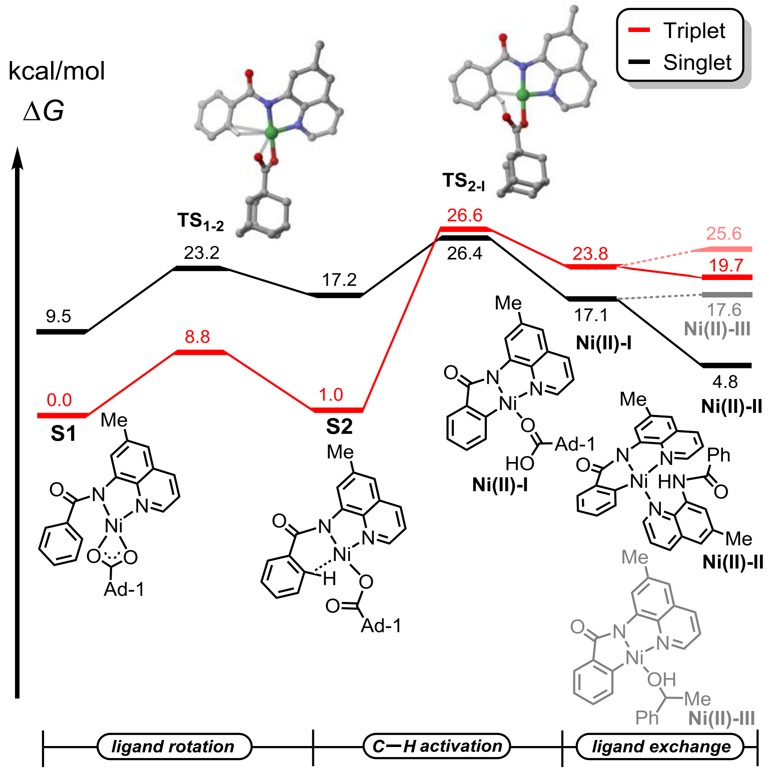

Figure S–20: Calculated energy profile of the nickellaelectro C−H alkoxylation before oxidation.

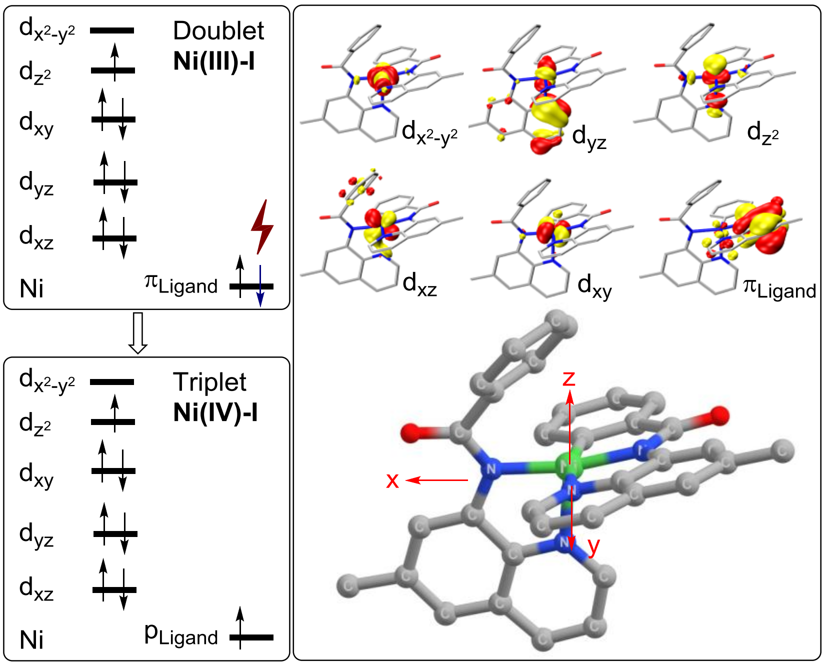

Figure S–21: Calculated electron-shift diagram of electrochemical oxidation process from **Ni(III)-I**.

3) Cartesian coordinates for the optimized structures

S1-Singlet

Ni	3.13236900	0.47767000	-0.10499300
N	1.52483200	1.26739000	0.48338900
O	4.65015000	-0.44701200	-0.80748600
C	3.85145700	-1.24077700	-1.40369300
O	2.62186000	-1.06050800	-1.16844800
C	0.31756200	0.70042500	-1.53113600
C	1.24283600	1.25800500	-2.42079200
C	1.21474000	0.91487600	-3.77090600
C	0.26781700	0.00361300	-4.24054900
C	-0.66793500	-0.54347900	-3.35967700
C	-0.65205600	-0.18354400	-2.01407100
H	1.98185400	1.97456600	-2.05236900
H	1.93574200	1.36070800	-4.46113300
H	0.25262300	-0.27462100	-5.29772000
H	-1.41503900	-1.25238200	-3.72631500
H	-1.38684200	-0.59649700	-1.31870600
C	4.33827900	-2.26357600	-2.38310500
C	5.63687100	-2.92152000	-1.89065700
C	3.27527100	-3.33985700	-2.63858400
C	4.62696800	-1.51686200	-3.70772200
C	5.13314300	-2.51047000	-4.75459200
H	3.70503900	-1.02318300	-4.05811600
H	5.37649400	-0.72916400	-3.52786500
C	6.14030100	-3.91262900	-2.94267900
H	6.39290000	-2.14459700	-1.69717300
H	5.44747200	-3.43939500	-0.93525700
C	3.78726300	-4.32974200	-3.68749800
H	3.04644000	-3.86369800	-1.69562600
H	2.34258400	-2.86315600	-2.97834600
H	5.33380500	-1.96709700	-5.69274000
C	6.42123500	-3.16789500	-4.25117400
H	7.06787900	-4.38332700	-2.57730800
C	5.07609500	-4.98709400	-3.18501000
H	3.01874000	-5.10142500	-3.85855400
C	4.06846600	-3.58428000	-4.99539800
H	5.44085700	-5.71930600	-3.92528700
H	4.87871900	-5.54170100	-2.25168800
H	4.41450900	-4.29174100	-5.76791400
H	3.14137400	-3.11952200	-5.37239600
H	6.80763000	-3.86961100	-5.00966900
H	7.19941100	-2.40209700	-4.09171200
O	-0.77581100	1.26827300	0.48549200
C	0.29758100	1.09091100	-0.08378200
C	1.70712200	2.00841200	1.64054800
C	3.08482300	2.23984300	1.92844600
C	0.77258300	2.58479600	2.49038900
C	3.50728400	3.04371600	3.01205900
C	1.17679400	3.38995900	3.59207700
H	-0.28599100	2.41460100	2.30354700
C	5.26873800	1.80173600	1.25262800
C	4.90477900	3.20832500	3.17233300
C	2.51684800	3.61801100	3.84736200
C	5.77647000	2.59528700	2.29670600
H	5.92427500	1.28187700	0.55004200
H	5.28137800	3.82239000	3.99462700
H	2.82702000	4.23625100	4.69319000
H	6.85679200	2.70808800	2.40152000
C	0.11200900	3.98118000	4.46591600
H	0.54086600	4.58527400	5.27748200
H	-0.56631900	4.62141200	3.87880400
H	-0.51029900	3.18998800	4.91490100
N	3.96740500	1.64302400	1.08574100

S1-Triplet

Ni	1.91173900	0.22892200	-0.02588600
N	0.63715700	1.41130400	0.86840600
O	3.01796300	-1.12353400	-1.11384900
C	2.91462300	-1.93967400	-0.14959400
O	2.30227000	-1.57002800	0.89296300
C	-0.91456000	-0.21732300	0.17474800
C	-0.38855100	-0.37403800	-1.11937200
C	-0.55092500	-1.57957900	-1.80646000

C	-1.23643900	-2.63466500	-1.20804400
C	-1.78861200	-2.47134600	0.06510600
C	-1.63794900	-1.26721200	0.74875700
H	0.08084800	0.47614700	-1.62917900
H	-0.14320800	-1.68764700	-2.81445600
H	-1.35300700	-3.58331200	-1.73785500
H	-2.33533800	-3.29509000	0.53139700
H	-2.06155600	-1.13631400	1.74735900
C	3.45437300	-3.34471600	-0.25858700
C	4.52167500	-3.46041100	-1.35371300
C	4.04498900	-3.80700900	1.08275600
C	2.26534300	-4.26520800	-0.61906600
C	2.74648400	-5.71305100	-0.73072000
H	1.48583300	-4.17420300	0.15481300
H	1.82054600	-3.93384600	-1.57246300
C	5.00040200	-4.91032100	-1.46309400
H	4.10297200	-3.11669600	-2.31217700
H	5.36821100	-2.79456900	-1.11547700
C	4.52528800	-5.25546200	0.96896000
H	4.88351400	-3.14630800	1.36219300
H	3.28073400	-3.71102700	1.86921500
H	1.88887800	-6.35628500	-0.98931200
C	3.81565300	-5.81200800	-1.82242700
H	5.76928300	-4.97844800	-2.25058900
C	5.59375800	-5.35623200	-0.12349700
H	4.95089200	-5.57169400	1.93583200
C	3.34032800	-6.15676000	0.60918700
H	5.96174100	-6.39376700	-0.19875300
H	6.46045900	-4.72401600	0.13545500
H	3.66955300	-7.20800200	0.54672300
H	2.57170000	-6.10415800	1.39913600
H	4.15308100	-6.85730100	-1.92698200
H	3.39176100	-5.50961700	-2.79537100
O	-1.53749900	1.54496100	1.66237400
C	-0.65823100	1.03169500	0.97841400
C	1.21809800	2.47315700	1.52818100
C	2.64916500	2.53294000	1.40158300
C	0.59347000	3.46118600	2.27687800
C	3.39076100	3.57520900	2.01426500
C	1.32888200	4.50991600	2.89236000
H	-0.48890800	3.41721700	2.39249900
C	4.57008100	1.54985200	0.53363200
C	4.79612000	3.55066300	1.83973300
C	2.70335300	4.56489300	2.76073800
C	5.38440700	2.54464000	1.10453700
H	4.99799100	0.73436000	-0.05648800
H	5.40341100	4.33686400	2.29635400
H	3.27776100	5.36655500	3.23154600
H	6.46500800	2.50510500	0.95723500
C	0.58095400	5.54176900	3.68098100
H	1.25584600	6.30105900	4.10005300
H	-0.16597900	6.05180300	3.05113700
H	0.02734400	5.07451600	4.51170000
N	3.25928800	1.55377500	0.68240600

$TS_{1\text{-}2}$-Singlet

Ni	2.39380400	0.08280800	-0.22900900
N	1.08667200	1.32245300	0.32103200
O	3.69624700	-1.14160500	-0.81112400
C	3.57818300	-0.92778700	-2.07061200
O	2.73107900	-0.09159900	-2.44963100
C	-0.68333700	-0.19950400	-0.27633900
C	0.00181000	-0.81975100	-1.32575400
C	-0.38266500	-2.08380500	-1.77391500
C	-1.46485600	-2.73305100	-1.18196600
C	-2.17066600	-2.10581300	-0.15199900
C	-1.78787200	-0.84327900	0.29362000
H	0.80520800	-0.29628000	-1.85394500
H	0.16040500	-2.55289600	-2.59884400
H	-1.76762300	-3.72431500	-1.52901300
H	-3.02558700	-2.60789100	0.30841700
H	-2.33221600	-0.34390400	1.09818000
C	4.47227500	-1.68185800	-3.01975400
C	4.42500200	-3.18685700	-2.69434800
C	4.04876400	-1.46279100	-4.47647200
C	5.92099500	-1.18145600	-2.83176300

C	6.85665200	-1.94531100	-3.77121600
H	5.96603900	-0.09913800	-3.04142700
H	6.22501800	-1.32621900	-1.78259300
C	5.36276300	-3.94876500	-3.63299100
H	4.71697000	-3.34267400	-1.64421300
H	3.38994200	-3.55384700	-2.80418200
C	4.98812500	-2.22913200	-5.41101700
H	3.00888900	-1.80260500	-4.61304700
H	4.06672200	-0.38613000	-4.70816900
H	7.88585900	-1.57563700	-3.63008600
C	6.79498700	-3.44015300	-3.44379600
H	5.31614600	-5.02387500	-3.39256800
C	4.92786700	-3.72407500	-5.08388400
H	4.67173700	-2.06463500	-6.45434500
C	6.42042000	-1.72122500	-5.22187000
H	5.58512500	-4.28828200	-5.76728900
H	3.90257700	-4.10325500	-5.23465700
H	7.10392300	-2.25064000	-5.90731300
H	6.47739100	-0.64814100	-5.47266100
H	7.48396500	-3.99896600	-4.09970900
H	7.12401500	-3.61393900	-2.40501200
O	-1.09778400	1.88650700	0.76310800
C	-0.26343100	1.12276900	0.29109100
C	1.63461500	2.28739100	1.16126800
C	2.91763700	1.91688200	1.65441500
C	1.09471000	3.48369100	1.60478800
C	3.62795600	2.71400600	2.58121600
C	1.80247500	4.31496900	2.51860700
H	0.10852500	3.78623900	1.25862900
C	4.57049100	0.27668500	1.62577800
C	4.87458500	2.21197900	3.02750800
C	3.04204500	3.93507300	2.99931900
C	5.33554500	0.99990400	2.55852400
H	4.91274700	-0.67067600	1.20404100
H	5.46099100	2.79167100	3.74516900
H	3.58198000	4.56711900	3.70857800
H	6.29118800	0.59055700	2.89005800
C	1.16538000	5.60142300	2.94876800
H	1.80452600	6.15789300	3.64818400
H	0.95926200	6.24661300	2.07936900
H	0.19660700	5.41348900	3.43968400
N	3.40386300	0.72968400	1.20212700

$TS_{1\text{-}2}$-Triplet

Ni	3.03430800	0.62705100	-0.31529000
N	1.41698100	1.19198800	0.69671800
O	4.69799800	-0.45677200	-0.87749800
C	4.04630900	-0.92455600	-1.85637900
O	2.85928500	-0.52593700	-2.03554600
C	-0.07943400	-0.29878000	-0.48333200
C	0.06573800	0.26972600	-1.75188200
C	-0.34730100	-0.42642800	-2.88625500
C	-0.88843100	-1.70551800	-2.76299100
C	-1.03655600	-2.27977800	-1.49748800
C	-0.65235600	-1.57074200	-0.36266300
H	0.50040900	1.26744200	-1.84749100
H	-0.23802900	0.02971700	-3.87360900
H	-1.20056500	-2.25695900	-3.65399600
H	-1.46249900	-3.28164300	-1.39722500
H	-0.78865500	-1.99823000	0.63368000
C	4.63759500	-1.95220900	-2.78831400
C	3.79184900	-3.23963100	-2.68861500
C	4.56231900	-1.42845100	-4.23593500
C	6.09416100	-2.27732500	-2.44165900
C	6.63537200	-3.33398900	-3.40857800
H	6.70092500	-1.35882400	-2.49621000
H	6.15432300	-2.63880500	-1.40261100
C	4.33766500	-4.29477600	-3.65227200
H	3.81853600	-3.61664200	-1.65176100
H	2.74220500	-3.00217900	-2.92373200
C	5.10794100	-2.48457900	-5.19912100
H	3.51616800	-1.18403500	-4.47831100
H	5.14577000	-0.49541900	-4.31964000
H	7.68329900	-3.55842800	-3.14902100
C	5.79348200	-4.60850300	-3.29580000
H	3.72761300	-5.20940100	-3.56673500

C	4.26593100	-3.75883600	-5.08515300
H	5.05322100	-2.09453300	-6.22911700
C	6.56338800	-2.79957600	-4.84203900
H	4.63538500	-4.52061400	-5.79265600
H	3.21799300	-3.54505900	-5.35660200
H	6.97237400	-3.54494900	-5.54533100
H	7.18181900	-1.89065800	-4.93827500
H	6.18934600	-5.38510100	-3.97230600
H	5.85331000	-5.01166700	-2.27028900
O	-0.49831200	0.40896100	1.72326500
C	0.26117900	0.46976100	0.76066000
C	1.71323300	2.16909600	1.63610500
C	3.06693300	2.64764600	1.62017000
C	0.84871900	2.76360800	2.54822300
C	3.49610700	3.68913100	2.48378600
C	1.26262400	3.81255200	3.41183200
H	-0.17696700	2.40438300	2.60783900
C	5.18913000	2.42405200	0.68911600
C	4.85427200	4.07968600	2.39210900
C	2.56636000	4.26852600	3.38057800
C	5.69833600	3.45248800	1.50133000
H	5.82296300	1.88930000	-0.02371900
H	5.22185600	4.88054200	3.03938200
H	2.89678200	5.07046500	4.04525500
H	6.74901900	3.73511700	1.41723000
C	0.25589100	4.40179700	4.35311300
H	0.69358100	5.20120400	4.96714000
H	-0.60104700	4.82016700	3.80043900
H	-0.14959000	3.63017600	5.02767800
N	3.92440600	2.05299100	0.75474800

S2-Singlet

Ni	2.09813700	0.24519300	-0.26262300
N	0.88661900	1.31659300	0.66204900
O	3.29619300	-0.87466000	-1.13449700
C	3.36744300	-0.71461800	-2.41764800
O	2.70873700	0.11774800	-3.03683400
C	-0.63693400	-0.27756200	-0.12827600
C	0.16128000	-0.53113900	-1.25888400
C	0.04080600	-1.73715800	-1.95770500
C	-0.88680400	-2.68606000	-1.54122600
C	-1.70088300	-2.42410400	-0.43387200
C	-1.58046500	-1.22805100	0.26876900
H	0.77005400	0.26599500	-1.74000200
H	0.66098700	-1.91039600	-2.84012100
H	-0.98923700	-3.62852600	-2.08436500
H	-2.43366300	-3.16861200	-0.11203800
H	-2.20221000	-1.02341200	1.14325700
C	4.33971700	-1.66043900	-3.11223000
C	3.89582700	-3.11689100	-2.87371600
C	4.38640000	-1.39315300	-4.61996900
C	5.75236700	-1.48050600	-2.52484000
C	6.73079500	-2.44140700	-3.20469000
H	6.08062600	-0.43597400	-2.66623300
H	5.72149600	-1.67034800	-1.44026300
C	4.87178700	-4.08103600	-3.55188500
H	3.85090800	-3.30852900	-1.79014000
H	2.87723200	-3.26295300	-3.27351300
C	5.36326000	-2.35771900	-5.29721800
H	3.37553500	-1.50598900	-5.04377900
H	4.68850600	-0.34855300	-4.79895300
H	7.73701000	-2.29615700	-2.77691400
C	6.27219800	-3.88295000	-2.96415900
H	4.53848600	-5.11743400	-3.37474700
C	4.90602800	-3.79948200	-5.05663900
H	5.38427600	-2.15219700	-6.38062900
C	6.76365500	-2.16093500	-4.70955500
H	5.59152100	-4.50405200	-5.55816300
H	3.90483000	-3.95631100	-5.49372900
H	7.48210000	-2.83649500	-5.20485300
H	7.10964400	-1.12938400	-4.89465200
H	6.98131700	-4.58851900	-3.43006100
H	6.26248200	-4.10101200	-1.88239700
O	-1.31263700	1.47894700	1.35265200
C	-0.41984700	0.95499400	0.69982400
C	1.46921100	2.29647400	1.44326000

C	2.89105600	2.23112400	1.40464600
C	0.87704100	3.27358900	2.22651900
C	3.69773000	3.13447100	2.13233200
C	1.67254400	4.19667600	2.96178400
H	-0.20972200	3.32783200	2.27777500
C	4.73130000	1.08594500	0.55867300
C	5.09941500	2.96116700	2.02718700
C	3.05349900	4.12769900	2.91313700
C	5.60790100	1.94323200	1.24878300
H	5.09177000	0.26760100	-0.06823700
H	5.76665200	3.63607000	2.56964400
H	3.66512000	4.83386000	3.47985800
H	6.68354700	1.78547900	1.15432800
C	0.97735500	5.23778800	3.78558300
H	1.69339000	5.88752800	4.30753500
H	0.33219800	5.87011200	3.15417800
H	0.32216700	4.76895800	4.53764700
N	3.42050500	1.23439100	0.63979400

S2-Triplet

Ni	2.49717200	-0.27516300	0.17703600
N	1.08035700	0.83980500	0.93105000
O	3.80327700	-1.68273200	-0.50619400
C	3.73142900	-1.17125600	-1.66360800
O	2.97971700	-0.16828000	-1.83328400
C	-0.39914400	-0.96357500	0.45373200
C	0.17274300	-1.29670700	-0.78331400
C	0.00175500	-2.57423100	-1.32054500
C	-0.74619100	-3.52590700	-0.62999900
C	-1.34294000	-3.18936500	0.58788100
C	-1.17917000	-1.91366500	1.12188500
H	0.70654000	-0.54156300	-1.36914000
H	0.44823600	-2.81768800	-2.28823800
H	-0.87609400	-4.52860700	-1.04498900
H	-1.93859700	-3.93111000	1.12646000
H	-1.64399200	-1.64199000	2.07252800
C	4.50413500	-1.74138200	-2.82679700
C	3.49395100	-2.28836000	-3.85802000
C	5.32695400	-0.62112300	-3.49161600
C	5.44353200	-2.87257900	-2.39513900
C	6.18831000	-3.42380000	-3.61411900
H	6.15880600	-2.49360800	-1.64694100
H	4.86138100	-3.66954400	-1.90580700
C	4.24129700	-2.84095700	-5.07304700
H	2.88511700	-3.08073600	-3.38955700
H	2.80781500	-1.48061300	-4.15838600
C	6.07343800	-1.17578200	-4.70642000
H	4.65209400	0.19640300	-3.78956600
H	6.04072800	-0.20566800	-2.75942000
H	6.86056500	-4.23535100	-3.28962400
C	5.17700700	-3.96797000	-4.62749300
H	3.50796000	-3.23041500	-5.79865100
C	5.06110400	-1.71995500	-5.71875000
H	6.66122100	-0.36526600	-5.16849300
C	7.00829300	-2.30406100	-4.26164300
H	5.58631400	-2.10204100	-6.61064000
H	4.39295400	-0.91009400	-6.05819400
H	7.56672400	-2.69612100	-5.12874000
H	7.75319400	-1.91778200	-3.54487600
H	5.70437700	-4.38876800	-5.50052800
H	4.59297100	-4.78812300	-4.17583000
O	-1.09721100	0.92518400	1.71202400
C	-0.18849100	0.38476300	1.08899500
C	1.54447100	2.03165000	1.45691100
C	2.97152900	2.19500500	1.39823400
C	0.80405500	3.06377000	2.01803300
C	3.59341100	3.37330300	1.88618400
C	1.41742800	4.24963600	2.50485400
H	-0.27632900	2.94843400	2.08793100
C	5.01353500	1.25774000	0.79631100
C	5.00481200	3.44124900	1.79075200
C	2.78887600	4.40083200	2.43812600
C	5.71318500	2.39048300	1.24974000
H	5.53850700	0.40136300	0.36328900
H	5.52043400	4.33465400	2.15321200
H	3.27053800	5.30808800	2.81121100

H	6.80102300	2.41964700	1.16793100
C	0.54367200	5.31907200	3.08714700
H	1.13075600	6.18385100	3.42651300
H	-0.19301300	5.67039800	2.34642600
H	-0.03061300	4.93249800	3.94483700
N	3.69935100	1.17634700	0.87212000

$TS_{2\text{-}I}$-Singlet

Ni	2.08504700	-0.04887000	-0.02563700
N	0.93730300	1.22873000	0.66859300
O	3.32956000	-1.17387700	-0.90640500
C	3.20350800	-1.10214200	-2.16446200
O	2.21943700	-0.51548700	-2.69666200
C	-0.67613900	-0.17064300	-0.25831900
C	0.42128200	-0.88079500	-0.79465500
C	0.18845900	-2.18886400	-1.24488100
C	-1.08892400	-2.75043200	-1.20889400
C	-2.16522400	-2.01027800	-0.71799100
C	-1.95606200	-0.71916600	-0.23368000
H	1.37067200	-0.42651200	-1.69003600
H	1.00958500	-2.77663500	-1.66614200
H	-1.24548500	-3.76902400	-1.57346500
H	-3.16705600	-2.44722100	-0.69868400
H	-2.77291600	-0.13379300	0.19633600
C	4.25012200	-1.76993200	-3.02409200
C	4.16508200	-3.29385400	-2.78806700
C	4.03476800	-1.47871000	-4.51362700
C	5.65064300	-1.28459000	-2.60443900
C	6.71636900	-1.99707600	-3.43986200
H	5.71862800	-0.19207600	-2.74306100
H	5.80396200	-1.49009500	-1.53356100
C	5.23314800	-4.00458200	-3.62190900
H	4.30647400	-3.50435000	-1.71623300
H	3.15990700	-3.65381100	-3.06662600
C	5.10505500	-2.19349300	-5.34227500
H	3.02903700	-1.81290800	-4.81318600
H	4.07723700	-0.39124000	-4.68777400
H	7.71181600	-1.63603600	-3.13270200
C	6.61964200	-3.50716100	-3.20316500
H	5.15849900	-5.09043000	-3.44584300
C	5.00845700	-3.70347200	-5.10610700
H	4.93834700	-1.97404600	-6.40974300
C	6.49176600	-1.69664200	-4.92419400
H	5.76081700	-4.23141700	-5.71625900
H	4.01787700	-4.07309500	-5.42180200
H	7.26997000	-2.18956200	-5.53128200
H	6.57625700	-0.61192400	-5.10808300
H	7.39892100	-4.03099100	-3.78224100
H	6.79818600	-3.73567600	-2.13861600
O	-1.26581300	1.91877200	0.77278300
C	-0.39718400	1.12639200	0.42855500
C	1.54725300	2.21754100	1.41053800
C	2.94949400	1.99853000	1.54939000
C	0.99607000	3.33578200	2.01889800
C	3.77043100	2.88214000	2.28708900
C	1.80850600	4.23693100	2.76133700
H	-0.07359100	3.51708600	1.92212100
C	4.72720100	0.60114200	1.01791300
C	5.14630800	2.55432300	2.36363500
C	3.16775500	4.01381200	2.89259300
C	5.61756600	1.42131600	1.73540100
H	5.06440900	-0.29727400	0.49620800
H	5.82340000	3.20529300	2.92300100
H	3.79220600	4.70406100	3.46497600
H	6.67279600	1.14676800	1.78029200
C	1.15505100	5.42823900	3.39425600
H	1.87887600	6.04361500	3.94636100
H	0.67278700	6.06151200	2.63191000
H	0.36211100	5.11551500	4.09284400
N	3.43979800	0.88722800	0.93418900

$TS_{2\text{-}I}$-Triplet

Ni	1.42888300	0.46360800	-1.25299700
N	1.09897000	0.62352700	0.66092800
O	2.98489200	-0.70380900	-1.66481500
C	2.85289500	-1.94495700	-1.53762100

O	1.74147900	-2.48779900	-1.25271200
C	-0.86663600	-0.52704500	0.05840400
C	-0.27809600	-0.81552800	-1.19805900
C	-1.13142900	-1.24790400	-2.22487300
C	-2.50666800	-1.39249700	-2.02527400
C	-3.05504500	-1.13006600	-0.76897800
C	-2.23379000	-0.70305200	0.27517300
H	0.89059900	-1.59761500	-1.22558800
H	-0.70834700	-1.50809800	-3.20152300
H	-3.15033000	-1.72413900	-2.84482000
H	-4.12861000	-1.25560900	-0.60308800
H	-2.64518100	-0.48197400	1.26349000
C	4.05255000	-2.84978100	-1.70549200
C	5.29683100	-2.06501800	-2.13548000
C	4.33181800	-3.53960800	-0.35293000
C	3.74381900	-3.93336100	-2.75637900
C	4.94017700	-4.87780100	-2.89258500
H	2.84391000	-4.49044600	-2.45263500
H	3.52211300	-3.45336600	-3.72499400
C	6.48944300	-3.01459300	-2.27210500
H	5.09949700	-1.55516300	-3.09236500
H	5.51164200	-1.27949400	-1.39388300
C	5.52730300	-4.48417200	-0.49156200
H	4.53389900	-2.77339800	0.41504900
H	3.43532300	-4.09503300	-0.03530200
H	4.70735700	-5.64445000	-3.65011100
C	6.17519300	-4.08204400	-3.32408000
H	7.37574900	-2.43713400	-2.58317600
C	6.76170900	-3.68839100	-0.92414700
H	5.71668700	-4.96658600	0.48167700
C	5.21175900	-5.55112500	-1.54401700
H	7.63421400	-4.35886400	-1.00460400
H	7.00698800	-2.92733000	-0.16372200
H	6.05815400	-6.25279000	-1.63576600
H	4.33336400	-6.14164300	-1.23239000
H	7.03739300	-4.75955500	-3.44616800
H	5.99610100	-3.60663600	-4.30371600
O	-0.40178400	0.07725000	2.33368400
C	-0.02616000	0.06237900	1.16416800
C	1.96147200	1.45810300	1.32443500
C	2.82830900	2.20935100	0.45360200
C	2.07002100	1.66985300	2.69272100
C	3.77223700	3.12367600	0.98856500
C	3.01536000	2.58168100	3.23339700
H	1.40337300	1.11848900	3.35543700
C	3.45804800	2.69192400	-1.72873900
C	4.58079400	3.82783400	0.06302100
C	3.85319300	3.29188300	2.39411600
C	4.42487500	3.61517800	-1.28877900
H	3.31325000	2.49667000	-2.79511600
H	5.32303800	4.53887000	0.43583000
H	4.58476800	3.99476000	2.80060200
H	5.03435300	4.14599100	-2.02225200
C	3.08053900	2.75036600	4.72136100
H	3.30197200	1.79072500	5.21617000
H	3.85192500	3.47583000	5.01524200
H	2.11228500	3.09479400	5.11980600
N	2.69237000	2.02395300	-0.88776700

Ni(II)-I-Singlet

Ni	1.92145900	0.09819100	-0.13694200
N	0.89959300	1.14752600	0.98974100
O	3.04557100	-1.02039700	-1.20256200
C	3.24324900	-2.20689100	-0.92449200
O	2.60242600	-2.80080700	0.04802400
C	-0.77907800	-0.27485400	0.23448300
C	0.29670900	-0.80092900	-0.51147700
C	-0.00614700	-1.80843600	-1.43675200
C	-1.31416000	-2.28744600	-1.58168000
C	-2.35369500	-1.76394800	-0.81072000
C	-2.08502100	-0.74223000	0.10092500
H	1.91210000	-2.17587400	0.37693700
H	0.77206100	-2.24088600	-2.07445500
H	-1.52225300	-3.07763800	-2.30925300
H	-3.37132000	-2.14575600	-0.92902500
H	-2.87811500	-0.29351100	0.70601500

C	4.18839400	-3.06606300	-1.70708800
C	5.09972900	-2.20461700	-2.59009500
C	5.04743800	-3.94104300	-0.77398200
C	3.32929300	-3.98718500	-2.60923100
C	4.25106200	-4.88220700	-3.44001200
H	2.65949700	-4.59834000	-1.98369700
H	2.69778700	-3.36997600	-3.26974000
C	6.01277400	-3.10701000	-3.42230100
H	4.48585200	-1.56833200	-3.24584200
H	5.70083100	-1.53376800	-1.95391500
C	5.96211800	-4.83457900	-1.61530500
H	5.64494400	-3.29285000	-0.11142800
H	4.39663400	-4.55702100	-0.13465300
H	3.62787000	-5.53075300	-4.07713700
C	5.15841900	-4.01257800	-4.31390100
H	6.66240700	-2.47484700	-4.04917800
C	6.87048900	-3.96585800	-2.48934700
H	6.57471900	-5.45061300	-0.93708900
C	5.10731800	-5.74109300	-2.50568800
H	7.54832000	-4.60488300	-3.07990700
H	7.50292200	-3.32126400	-1.85541700
H	5.75594300	-6.40830500	-3.09769800
H	4.46171600	-6.38377900	-1.88344800
H	5.80726100	-4.65208100	-4.93559500
H	4.54959300	-3.40150400	-5.00163800
O	-1.22068000	1.43223100	1.87403000
C	-0.42715300	0.85749200	1.13719300
C	1.57772800	2.14909700	1.64506000
C	2.96742600	2.18718500	1.30981900
C	1.09944100	3.08819300	2.55052300
C	3.83693800	3.15159700	1.87396300
C	1.96245900	4.06084900	3.12375400
H	0.04365400	3.06910100	2.81719500
C	4.66384700	1.22439800	0.05613400
C	5.19306900	3.09963800	1.46786900
C	3.30542400	4.09202500	2.79194700
C	5.60162400	2.14260800	0.56445100
H	4.95814600	0.45560900	-0.66284100
H	5.90368700	3.82328800	1.87613000
H	3.97067700	4.83974600	3.23076000
H	6.63991700	2.08210700	0.23355900
C	1.38120100	5.04822500	4.09072100
H	2.14284300	5.74430700	4.46890000
H	0.58010700	5.63747700	3.61530900
H	0.92507200	4.53307300	4.95177100
N	3.39401700	1.24848300	0.42047600

Ni(II)-I-Triplet

Ni	1.15574700	0.79052800	-1.55524600
N	1.10832800	0.62768000	0.39569500
O	2.64487500	-0.56254300	-2.01963100
C	2.59474100	-1.76390900	-1.76257300
O	1.46905000	-2.38322100	-1.52246900
C	-0.87153500	-0.53349800	-0.15661000
C	-0.54219800	-0.27631200	-1.51420000
C	-1.44623800	-0.75223500	-2.47734100
C	-2.61749500	-1.43160600	-2.12314800
C	-2.91335000	-1.66338600	-0.77879100
C	-2.03496200	-1.21567700	0.20726100
H	0.72711000	-1.72200000	-1.56513800
H	-1.23823200	-0.59953900	-3.54261900
H	-3.30152000	-1.78600000	-2.90019200
H	-3.82617400	-2.19803300	-0.50147100
H	-2.23593600	-1.38800600	1.26823300
C	3.82586300	-2.62152500	-1.67500300
C	5.07279400	-1.81749600	-2.06046200
C	3.96831300	-3.11015600	-0.21462000
C	3.69595300	-3.84801600	-2.59967200
C	4.94628200	-4.72074700	-2.46748100
H	2.79709100	-4.42349300	-2.32905100
H	3.57123700	-3.51005000	-3.64223000
C	6.31683600	-2.69850100	-1.93082000
H	4.96865300	-1.44752700	-3.09321800
H	5.15665300	-0.93350000	-1.40869100
C	5.21725800	-3.98527400	-0.09037900
H	4.03749800	-2.23884500	0.45867800

H	3.07068500	-3.67940400	0.07337700
H	4.84396400	-5.59108500	-3.13623800
C	6.18417800	-3.90912400	-2.85885400
H	7.20397400	-2.10867200	-2.21403300
C	6.45490400	-3.17388600	-0.48189100
H	5.30824800	-4.32488000	0.95443600
C	5.08343900	-5.19632100	-1.01817100
H	7.36164200	-3.79219000	-0.37069600
H	6.56819100	-2.30729500	0.19155600
H	5.96800400	-5.84752300	-0.91729500
H	4.20360500	-5.79720100	-0.73182200
H	7.08625000	-4.54025800	-2.78950000
H	6.10234700	-3.57502800	-3.90726200
O	-0.14023900	-0.27821300	2.11862100
C	0.06900500	-0.05785700	0.92644400
C	2.10550200	1.27984500	1.06434000
C	2.94130200	2.09133900	0.21384000
C	2.39060800	1.25542500	2.42523100
C	4.02576400	2.82664000	0.75989800
C	3.47538800	1.98834500	2.97370300
H	1.75267800	0.65371900	3.07263600
C	3.38073400	2.86080500	-1.93207700
C	4.79787400	3.59965900	-0.14183400
C	4.27947600	2.75799400	2.15325300
C	4.47790300	3.61836700	-1.48098600
H	3.10406000	2.85267400	-2.99020100
H	5.64434500	4.17726000	0.23957000
H	5.11796400	3.32454500	2.56612100
H	5.05621000	4.20619300	-2.19606900
C	3.72546400	1.90551200	4.44956800
H	3.91226400	0.86402900	4.75838100
H	4.58941000	2.51431400	4.75103000
H	2.84531400	2.24954200	5.01694200
N	2.64552300	2.13103900	-1.11516500

Ni(II)-II-Singlet

Ni	4.32606600	2.40734000	4.59707400
O	4.31041800	0.50096500	0.58555500
O	0.82414800	1.92606700	6.41373700
N	4.05162700	2.38536100	1.83736100
N	2.89438200	1.56733000	5.43651200
N	5.04016900	0.59087000	4.42242000
N	5.83970900	3.31415000	3.84600500
C	4.14504000	-0.32706000	4.88464000
C	1.83916500	2.32696000	5.85242300
C	2.94799000	0.19604900	5.46835800
C	3.65754400	1.15284200	1.38145700
C	2.37647500	0.64323100	1.95874900
C	7.34943600	4.11668700	2.12741800
C	6.13627100	3.47554800	2.52275600
C	3.31562800	3.98932700	4.82498300
C	5.26230500	2.99498400	1.49691000
C	1.47960300	6.07503200	5.39792900
H	0.77839800	6.88583000	5.61338100
C	2.00694800	-0.70650900	5.94913500
H	1.09171300	-0.31830200	6.39336000
C	1.18870200	4.76751400	5.78797800
H	0.26089700	4.52045700	6.31231300
C	5.63363300	3.12278800	0.17401300
H	4.95633500	2.74412800	-0.59260400
C	6.16311200	0.17562900	3.86245800
H	6.85589200	0.94121400	3.50509600
C	3.36877800	-2.60636300	5.27114200
H	3.52862200	-3.68403400	5.18528000
C	2.67449200	6.34035300	4.72460000
H	2.90467200	7.36394800	4.41324600
C	4.36358000	-1.72264700	4.78209900
C	2.09880800	3.75139500	5.49922700
C	3.58157100	5.31106800	4.44386000
H	4.50585400	5.56289700	3.91419400
C	2.17326700	-0.74233800	1.93925300
H	2.95025400	-1.37866500	1.51068600
C	6.46770200	-1.19069900	3.71912700
H	7.40764600	-1.47989100	3.24584700
C	7.68249900	4.23196300	0.75652900
H	8.62208300	4.72277300	0.49038100

C	1.38974600	1.47739700	2.49805200
H	1.51749100	2.56268700	2.50336500
C	6.66910600	3.77678100	4.76861600
H	6.37717300	3.62250100	5.81051100
C	0.02778200	-0.45314400	3.00433600
H	-0.88822900	-0.88134700	3.41971100
C	7.86805000	4.43878100	4.46062900
H	8.50470300	4.79949200	5.26978500
C	5.57391800	-2.13542500	4.17365300
H	5.78576300	-3.20292900	4.06908500
C	6.84903400	3.73287800	-0.22133900
C	8.20747800	4.60556200	3.14031200
H	9.13515200	5.10714100	2.85315300
C	0.21839600	0.92846500	3.01587300
H	-0.54628800	1.58491700	3.43671900
C	2.21264000	-2.10732400	5.84483600
C	1.00727200	-1.28946700	2.46418600
H	0.86181400	-2.37253500	2.45879100
H	3.56023500	2.77512200	2.64505100
C	7.18196900	3.83209100	-1.67707900
H	8.13525900	4.35387300	-1.83840500
H	7.25407300	2.83003800	-2.13025000
H	6.39278000	4.37240100	-2.22424300
C	1.13847000	-3.02442400	6.34680000
H	1.42139500	-4.08109200	6.24097000
H	0.91546500	-2.82921700	7.40816800
H	0.19957400	-2.86223300	5.79164400

Ni(II)-II-Triplet

Ni	3.71024500	2.04006700	4.00536100
O	4.65143700	0.69847700	0.39011900
O	2.20066500	2.64479600	7.74193800
N	4.02842500	2.48230500	1.65841800
N	3.25905000	1.68688800	5.91897500
N	4.44365800	0.03727600	4.22773300
N	5.60490400	2.81121900	3.93533500
C	4.30337900	-0.39055300	5.51230100
C	2.55790500	2.65424400	6.56252800
C	3.64921500	0.49024500	6.45027100
C	3.75908000	1.33913600	0.91069200
C	2.33019200	0.91721900	0.83532700
C	7.37705700	3.85120200	2.64636400
C	6.09346700	3.24369500	2.74419100
C	2.62442200	3.72649900	4.28706700
C	5.31506700	3.09550200	1.56367100
C	1.22316600	5.97940700	5.33344400
H	0.68523400	6.84505200	5.73037300
C	3.49170400	0.04069700	7.75952300
H	2.99408400	0.70225800	8.46774500
C	1.54298100	4.90550000	6.16285800
H	1.26574400	4.90304000	7.22093800
C	5.80517900	3.54118100	0.35873100
H	5.19205300	3.42134100	-0.53766000
C	5.06244200	-0.73046400	3.34983100
H	5.16918800	-0.34420900	2.33314100
C	4.59808000	-2.06536600	7.28219900
H	4.96985100	-3.04438900	7.59536500
C	1.59767100	5.93837700	3.98877100
H	1.35194600	6.77617900	3.32840600
C	4.78176000	-1.66073100	5.93612700
C	2.23170300	3.80363300	5.64477600
C	2.28743100	4.82836400	3.48654600
H	2.56018500	4.85234200	2.42180500
C	2.03058900	-0.15394400	-0.01678900
H	2.84220100	-0.61436000	-0.58352100
C	5.57592400	-1.99902400	3.68006400
H	6.07643600	-2.59181200	2.91216800
C	7.84806200	4.29191500	1.38585800
H	8.83736700	4.75380900	1.33168400
C	1.30272300	1.51184200	1.58062800
H	1.50820100	2.32829600	2.27740000
C	6.32584300	2.94800000	5.02939300
H	5.87575600	2.58501300	5.95813700
C	-0.29603200	-0.01709400	0.60809000
H	-1.32288000	-0.38113700	0.51962400
C	7.60733000	3.53205400	5.02767400

H	8.15986700	3.61875500	5.96464800
C	5.43077300	-2.46364100	4.96693200
H	5.81326700	-3.44613600	5.25703700
C	7.08362800	4.14808600	0.24602700
C	8.12992800	3.98340800	3.83886500
H	9.11994300	4.44458600	3.79593000
C	-0.00397400	1.04334500	1.46575300
H	-0.79709700	1.50907300	2.05544500
C	3.95998400	-1.23016400	8.17977200
C	0.72414300	-0.61602900	-0.13350800
H	0.49873100	-1.44913300	-0.80372200
H	3.25792600	3.14798500	1.71810100
C	7.56174800	4.61038400	-1.09480900
H	7.60107900	3.77054000	-1.80719000
H	6.87233800	5.35721500	-1.52055300
H	8.56268500	5.05889500	-1.03288300
C	3.75228900	-1.63732200	9.60791600
H	4.22728400	-0.91850600	10.29541400
H	2.67930100	-1.65221100	9.86013500
H	4.16723600	-2.63450200	9.81190700

Ni(II)-III-Singlet

Ni	2.10852900	-0.03219700	-0.49483800
N	0.86242800	0.96429300	0.44387300
C	-0.55533600	-0.70036200	-0.35040600
C	0.66019300	-1.18146700	-0.88078900
C	0.60671600	-2.39468400	-1.57917500
C	-0.60223700	-3.08106600	-1.74943200
C	-1.79192200	-2.57081500	-1.22446800
C	-1.76758700	-1.36923000	-0.51628600
H	1.50826500	-2.84152300	-2.01448900
H	-0.61419800	-4.02633800	-2.30060700
H	-2.73154700	-3.11160400	-1.36601100
H	-2.67776800	-0.94197600	-0.08515600
O	-1.39405400	1.16376500	0.92280100
C	-0.44708700	0.57509200	0.41167700
C	1.34158600	2.10665400	1.04058800
C	2.75623900	2.25854000	0.88900600
C	0.65272000	3.08863400	1.74269300
C	3.44514800	3.36197600	1.44809800
C	1.33115400	4.20841500	2.29511100
H	-0.42459200	2.98674900	1.86600000
C	4.70065200	1.32891100	0.03914800
C	4.84938400	3.39499100	1.26239300
C	2.70100800	4.34202200	2.15227000
C	5.47253500	2.37948900	0.57034400
H	5.16081700	0.51645900	-0.52754000
H	5.42604500	4.22647100	1.67646200
H	3.22513100	5.19911700	2.58238100
H	6.55346800	2.37682000	0.41915200
C	0.52739100	5.23396500	3.03716800
H	1.15948400	6.04720100	3.42052400
H	-0.24461800	5.67467000	2.38543700
H	-0.00210500	4.77706000	3.88908600
N	3.38992200	1.27930400	0.18843700
O	3.41006400	-0.92892600	-1.63800300
C	3.28173100	-0.59492800	-3.05239000
C	4.47434500	-1.13981400	-3.79928500
H	4.51297600	-2.23653600	-3.70761100
H	4.38956700	-0.89446100	-4.86806900
H	5.41982200	-0.72892400	-3.41676700
C	3.08121600	0.89922100	-3.11260200
C	1.77563600	1.40668100	-3.09863200
C	4.15621100	1.79240200	-3.08083100
C	1.54784000	2.77953600	-3.02243500
H	0.93058000	0.71477100	-3.14447700
C	3.93047100	3.16628000	-3.00313100
H	5.18228000	1.41890400	-3.09729300
C	2.62683100	3.66183700	-2.96338000
H	0.52381100	3.16077800	-3.00322900
H	4.77928100	3.85388800	-2.96622000
H	2.45153700	4.73852400	-2.89338300
H	2.36170500	-1.07670800	-3.41983700
H	3.42781000	-1.89557500	-1.54908500

Ni(II)-III-Triplet

Ni	2.60696100	-0.39519200	-0.04499800
N	1.42729500	1.09631100	0.41926300
C	-0.05176600	-0.25451600	-0.83097200
C	1.01315900	-1.18609900	-0.92491500
C	0.76449200	-2.34414800	-1.67552700
C	-0.46365000	-2.56765200	-2.31019900
C	-1.49065700	-1.62733600	-2.20473900
C	-1.28236500	-0.46828100	-1.45788300
H	1.54768700	-3.10355300	-1.78768500
H	-0.62031600	-3.48075600	-2.89332000
H	-2.44849700	-1.79858100	-2.70417500
H	-2.06414700	0.28981000	-1.35621400
O	-0.73764900	1.84695400	0.11515400
C	0.15771800	1.01926600	-0.05120100
C	1.97129400	2.08256700	1.18760600
C	3.32640800	1.81316000	1.60283200
C	1.39026000	3.27674800	1.60398200
C	4.03153200	2.74089500	2.41353700
C	2.09574400	4.20939100	2.40782500
H	0.36470500	3.48143100	1.29684300
C	5.12572300	0.34527000	1.55791400
C	5.35392600	2.39481500	2.78505900
C	3.39363300	3.94474900	2.80600200
C	5.89897900	1.20283800	2.36202500
H	5.53279300	-0.60530900	1.20123500
H	5.93044600	3.08348100	3.40878600
H	3.94367700	4.65656100	3.42668400
H	6.91503500	0.91294700	2.63563800
C	1.40469900	5.47769600	2.80981600
H	2.04948200	6.11347400	3.43265900
H	1.10168200	6.05742300	1.92259300
H	0.48325000	5.26112800	3.37453200
N	3.89022900	0.64133700	1.19608400
O	3.86862600	0.03320600	-1.62286700
C	3.47478200	-0.04522600	-2.99923300
C	4.66735900	0.25726900	-3.88342000
H	5.50110500	-0.42342400	-3.65369900
H	4.39526000	0.13284200	-4.94139300

H	5.00434600	1.29771900	-3.74155800
C	2.28807400	0.84281700	-3.29076500
C	1.29348400	0.40177600	-4.16895600
C	2.16892400	2.10937300	-2.71071900
C	0.19245500	1.20715300	-4.45539500
H	1.37426600	-0.59340000	-4.61575300
C	1.06618000	2.91445900	-2.99211500
H	2.93075100	2.47393600	-2.01627700
C	0.07368900	2.46520900	-3.86344800
H	-0.58436500	0.84397400	-5.13333700
H	0.97731400	3.89530600	-2.51799800
H	-0.79531600	3.09302100	-4.07672700
H	3.16627200	-1.09088100	-3.14206700
H	4.41692800	0.82022800	-1.47902900

Ni(III)-I-Doublet

Ni	3.76700700	2.09047900	3.83167900
O	3.63189700	1.79670400	-0.20635000
O	1.87266400	2.53265100	7.32582800
N	4.13608700	2.40260000	1.96401600
N	3.07872100	1.67641400	5.54699600
N	4.42129900	0.20664500	3.89390100
N	5.59623900	2.93159000	4.08754300
C	4.03568900	-0.40734900	5.04423200
C	2.38556700	2.64181400	6.22026200
C	3.29018900	0.38216500	5.97422000
C	3.40905600	1.78189700	1.00034100
C	2.30194200	0.93820400	1.57151500
C	7.30947700	4.08009700	2.82120200
C	6.08139300	3.37460500	2.90324200
C	2.94764400	3.79197000	4.12668400
C	5.30483400	3.09965100	1.72795300
C	1.70396900	6.15675400	4.96024200
H	1.22018900	7.08484800	5.27512300
C	2.87437300	-0.21803600	7.15437400
H	2.30620200	0.37287300	7.87134300
C	1.71418900	5.04891400	5.80526200
H	1.24456200	5.07527100	6.79266800

C	5.80004600	3.54522900	0.50902900
H	5.22995000	3.33139500	-0.39323500
C	5.13495900	-0.45020500	3.00088200
H	5.42644500	0.09408200	2.09909600
C	3.90457600	-2.33365600	6.53398900
H	4.14352500	-3.37847300	6.74687600
C	2.31297100	6.07722900	3.70563700
H	2.30334700	6.94548500	3.04048000
C	4.35331600	-1.76045800	5.31762900
C	2.33825300	3.87350200	5.38568700
C	2.93698700	4.89744000	3.28190800
H	3.40103200	4.86104900	2.29406700
C	2.24064700	-0.41278200	1.21023700
H	2.94941000	-0.80196300	0.47541800
C	5.50456500	-1.79444600	3.19082000
H	6.09356200	-2.29935600	2.42341700
C	7.76832100	4.51636400	1.55404200
H	8.71433500	5.05987000	1.49109400
C	1.37917100	1.44318500	2.49647800
H	1.37054600	2.50950800	2.73359600
C	6.25377900	3.14317500	5.20786800
H	5.80022100	2.76657900	6.12921500
C	0.41219100	-0.75672700	2.75575300
H	-0.31357000	-1.42332300	3.22845600
C	7.48325800	3.82960100	5.22292700
H	8.00060600	3.98441300	6.17134400
C	5.11063700	-2.44776500	4.33825000
H	5.37868500	-3.49429100	4.50593100
C	7.02788300	4.25381200	0.41690900
C	8.00424800	4.29770000	4.03686300
H	8.95346900	4.83975800	4.01860100
C	0.43664200	0.59685700	3.08464700
H	-0.27953000	1.00251400	3.80376600
C	3.17768400	-1.57830900	7.43534300
C	1.30976000	-1.25752700	1.80893100
H	1.28693700	-2.31714000	1.54139000
C	2.69310300	-2.16488000	8.72665000
H	3.09223600	-1.60334800	9.58694900
H	1.59445500	-2.10850700	8.79467900
H	2.99177900	-3.21708600	8.83233800
C	7.49242600	4.70532900	-0.93470600
H	7.61657900	3.84648000	-1.61424100
H	6.74997800	5.37277800	-1.40171000
H	8.45000900	5.24143100	-0.87728100

Ni(III)-I-Quartet

Ni	3.93200200	1.90092800	3.67707800
O	3.66822500	1.72061000	-0.39239600
O	1.83464800	2.51770600	7.25016800
N	4.20454900	2.33270700	1.77933100
N	3.07668300	1.64879200	5.49790400
N	4.46388500	-0.01504700	3.99383800
N	5.67312000	2.92811800	3.87346200
C	3.93376200	-0.53363800	5.12717500
C	2.41403900	2.62002800	6.16979600
C	3.17393700	0.35393500	5.96713500
C	3.48767100	1.68850500	0.81923800
C	2.44350800	0.79496100	1.43241400
C	7.23425800	4.26780200	2.59197900
C	6.08340200	3.44458500	2.68758100
C	3.03527400	4.15821400	4.24584700
C	5.30195100	3.12981200	1.52427900
C	1.81269900	6.29704300	5.38881000
H	1.32131600	7.15855800	5.84797900
C	2.62975300	-0.18893500	7.12746700
H	2.05188800	0.46343300	7.77898100
C	1.79533700	5.06497800	6.03975400
H	1.30012400	4.93150400	7.00566300
C	5.71763100	3.65226200	0.30610600
H	5.14346500	3.41020700	-0.58705300
C	5.17536100	-0.76702900	3.17630200
H	5.57036100	-0.28525300	2.27731600
C	3.53702200	-2.39592100	6.66382100
H	3.67794200	-3.44768800	6.92514200
C	2.45546600	6.44473500	4.15607300
H	2.46515600	7.41573200	3.65253900

C	4.11701100	-1.89904100	5.47072800
C	2.42570300	3.94920800	5.46515300
C	3.09389100	5.34738200	3.55842200
H	3.60741400	5.44666700	2.59737500
C	2.39145100	-0.54959800	1.04983200
H	3.05503000	-0.90529800	0.25791500
C	5.41249200	-2.12797900	3.43816100
H	6.00547300	-2.71656600	2.73612300
C	7.61240300	4.77997200	1.32538900
H	8.50011200	5.41346600	1.25452400
C	1.58079200	1.25722000	2.43667700
H	1.56013300	2.32114400	2.69185500
C	6.34632400	3.17983300	4.97995900
H	5.95936600	2.74073000	5.90406800
C	0.69379500	-0.97226500	2.72122800
H	0.02436300	-1.66749100	3.23397300
C	7.50310300	3.97944000	4.98006100
H	8.03180400	4.16106800	5.91732600
C	4.88211400	-2.68929700	4.57909400
H	5.04373400	-3.74587800	4.80929700
C	6.86856300	4.47875400	0.20032200
C	7.94205200	4.52255600	3.79151400
H	8.83529700	5.15226500	3.76086600
C	0.70692000	0.37527700	3.07565500
H	0.03820400	0.74659500	3.85627000
C	2.80570300	-1.55418200	7.47865000
C	1.53119000	-1.43054400	1.70078100
H	1.51764300	-2.48591200	1.41641600
C	7.24832600	5.00853500	-1.14924300
H	7.43053900	4.18395000	-1.85747800
H	6.43360500	5.61648800	-1.57520900
H	8.15364900	5.62962900	-1.10229900
C	2.18084500	-2.05029900	8.74781200
H	2.56274700	-1.49162800	9.61788800
H	1.08882900	-1.90164700	8.73224000
H	2.38107800	-3.11873800	8.90976000

Ni(IV)-I-Singlet

Ni	3.59618000	2.08779700	3.62987400
O	3.47095300	1.93862100	-0.33313900
O	1.95526900	2.60606700	7.21071400
N	4.03942300	2.45517200	1.84389900
N	3.06146900	1.71955700	5.39212500
N	4.32642300	0.20595500	3.70373000
N	5.28116000	2.86241800	4.04150800
C	4.01878800	-0.37066800	4.89515900
C	2.41136800	2.70269700	6.08218800
C	3.31431400	0.43492200	5.83658500
C	3.29622200	1.86261600	0.86980000
C	2.29638000	0.98560000	1.54693300
C	7.16483900	3.96300300	2.99163700
C	5.91070000	3.31498700	2.92355600
C	2.88751400	3.85356400	3.96679600
C	5.25577800	3.09087500	1.68630200
C	1.76560700	6.22861700	4.83412600
H	1.32560600	7.17188300	5.16605600
C	2.97001000	-0.12561100	7.05696300
H	2.43168600	0.48097300	7.78315800
C	1.77577800	5.12595200	5.68439400
H	1.35123000	5.16931800	6.69100100
C	5.86971400	3.51397600	0.51932300
H	5.37652300	3.33988500	-0.43620200
C	5.01781800	-0.46400900	2.80327200
H	5.25755100	0.04812900	1.86799200
C	4.00561900	-2.24240000	6.45633300
H	4.27680000	-3.27413400	6.69248400
C	2.31792000	6.13127700	3.55585200
H	2.30796300	6.99683700	2.88801000
C	4.38106500	-1.70532600	5.20015900
C	2.34528400	3.92871800	5.24746700
C	2.88965900	4.93473500	3.10292300
H	3.31064900	4.88000200	2.09823300
C	2.14702900	-0.34060900	1.15843700
H	2.62840500	-0.69755100	0.24585100
C	5.43292200	-1.79003400	3.02481400
H	6.00014100	-2.30620500	2.24880900

C	7.76057400	4.38674900	1.77802400
H	8.73821300	4.87269700	1.81501200
C	1.68753700	1.44893000	2.74791800
H	1.46686200	2.51884700	2.84798300
C	5.82179200	3.00551000	5.23661000
H	5.25602900	2.64191100	6.09605300
C	0.85501100	-0.78178100	3.18229600
H	0.30815700	-1.49053400	3.80735400
C	7.08045000	3.61448700	5.38349500
H	7.50471600	3.70008000	6.38479900
C	5.10788400	-2.41063200	4.21074300
H	5.40903200	-3.44374500	4.40256300
C	7.12958600	4.17075500	0.56498200
C	7.74199800	4.10171600	4.27673200
H	8.71294600	4.59260600	4.37912700
C	0.95092900	0.54677600	3.55148100
H	0.45118800	0.91495800	4.44968700
C	3.31046300	-1.47048200	7.36795900
C	1.43980600	-1.21617800	1.98135000
H	1.35363800	-2.26729700	1.69424800
C	2.90293100	-2.01822800	8.70150900
H	3.33732300	-1.42081000	9.51931200
H	1.80863400	-1.97502600	8.82440200
H	3.22423500	-3.06139900	8.82638900
C	7.74983200	4.61509900	-0.72346500
H	7.88695500	3.76105500	-1.40580700
H	7.09503400	5.33402700	-1.24179200
H	8.72617300	5.09101900	-0.56002100

Ni(IV)-I-Triplet

Ni	3.74472900	2.10356200	3.78152200
O	3.59179400	1.84287600	-0.22808200
O	1.96528500	2.60989000	7.33927700
N	4.12510000	2.43320000	1.93871500
N	3.07633800	1.67994500	5.52332100
N	4.39272700	0.20770200	3.85759700
N	5.57023800	2.93926000	4.07696300
C	3.97251600	-0.39785200	4.98328200
C	2.41240200	2.71274600	6.21920600
C	3.22490500	0.41614200	5.92116400
C	3.39564400	1.80423500	0.97999700
C	2.35017400	0.91104200	1.58979600
C	7.27918800	4.11809000	2.83131000
C	6.05569500	3.40446500	2.89962500
C	2.94917000	3.80743800	4.08605600
C	5.28622200	3.14748600	1.71749700
C	1.73786700	6.18853700	4.92324800
H	1.26891600	7.12432800	5.23571800
C	2.74460200	-0.18656600	7.11200000
H	2.18016100	0.42244700	7.81548600
C	1.75947400	5.09638800	5.78070300
H	1.31371800	5.14206500	6.77784900
C	5.77491600	3.61759200	0.50706800
H	5.20483800	3.41765400	-0.39878200
C	5.11122000	-0.47437700	2.97659500
H	5.43597700	0.06266900	2.08205400
C	3.73750000	-2.30252800	6.47037300
H	3.93746700	-3.35512200	6.68709400
C	2.31731700	6.08595900	3.65409200
H	2.29619900	6.94500500	2.97784200
C	4.23980000	-1.75129100	5.26173800
C	2.36725900	3.90755400	5.36007000
C	2.92448700	4.90085500	3.22787700
H	3.36339900	4.85155700	2.23015500
C	2.33693900	-0.44109200	1.22984300
H	3.00483300	-0.79061900	0.43911400
C	5.43850300	-1.82363600	3.17309100
H	6.03126900	-2.34375000	2.41939700
C	7.73656800	4.57798900	1.57186000
H	8.68030900	5.12640600	1.52017600
C	1.47655100	1.36524100	2.59024500
H	1.39581300	2.43400300	2.80393300
C	6.22606100	3.13619900	5.20180000
H	5.77562100	2.74490500	6.11848700
C	0.67858700	-0.88938500	2.93569700
H	0.04266600	-1.59855300	3.47124300

C	7.45248800	3.82655500	5.22829000	C	1.51523600	-1.33690800	1.90860200
H	7.96806400	3.96723500	6.17971300	H	1.53332200	-2.39698100	1.64306500
C	4.99728300	-2.47042100	4.31122600	C	7.46365300	4.81146800	-0.91373400
H	5.22703700	-3.52387400	4.48749400	H	8.41914200	5.34974500	-0.84511600
C	6.99988500	4.33327600	0.42838700	H	7.59101700	3.96570100	-1.60876500
C	7.97189500	4.31793900	4.05079900	H	6.71877900	5.48471700	-1.36819800
H	8.91798600	4.86550600	4.04296400	C	2.48545100	-2.16407500	8.63778700
C	0.64323800	0.46447400	3.25936000	H	3.32335100	-2.54762800	9.24152900
H	-0.03501300	0.82972400	4.03450000	H	1.91141000	-1.45297400	9.24578700
C	2.99404100	-1.53048500	7.38366300	H	1.84184100	-3.02564700	8.39970800

Gram-scale experiment

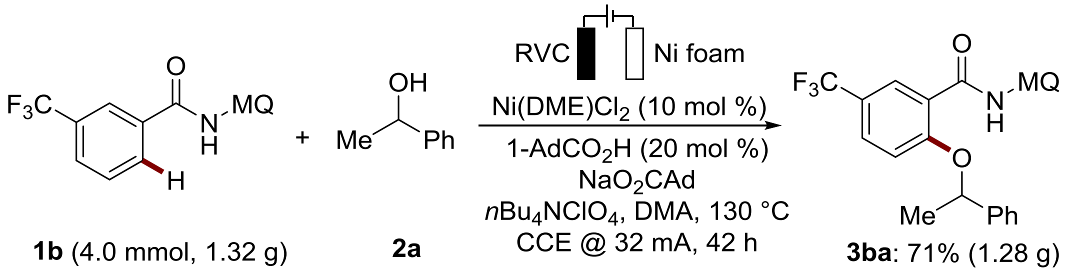

Scheme S–16: Gram-scale experiment with **1b**.

The electrolysis was carried out in 100 mL Schlenk flask, with a RVC anode (20 mm × 30 mm × 6 mm) and a nickel-foam cathode (20 mm × 30 mm × 1.4 mm). $Ni(DME)Cl_2$ (87.6 mg, 0.40 mmol, 10 mol %), 1-$AdCO_2H$ (144 mg, 0.8 mmol), NaO_2CAd (808 mg, 4.0 mmol), $n$$Bu_4NClO_4$ (7.0 mmol, 2.39 g) and benzamide **1b** (4.0 mmol, 1.32 g) were dissolved in DMA (40.0 mL), and then the 1-phenylethan-1-ol (**2a**) (40.0 mmol) was added. At 130 ℃, electrolysis was started with a constant current of 32.0 mA which was then maintained for 42 h. At ambient temperature, the mixture was transferred to a separating funnel and the electrodes were rinsed with EtOAc (50.0 mL). Then, the mixture was washed with H_2O (50.0 mL) and the organic layer was separated. The aqueous layer was extracted with EtOAc (2 × 50.0 mL). Evaporation of the collected organic layer and subsequent column chromatography on silica gel (*n*-hexane/aceton16/1→9/1) yielded the desired product **3ba** (1.28 g, 71%).

Traceless removal of the directing group

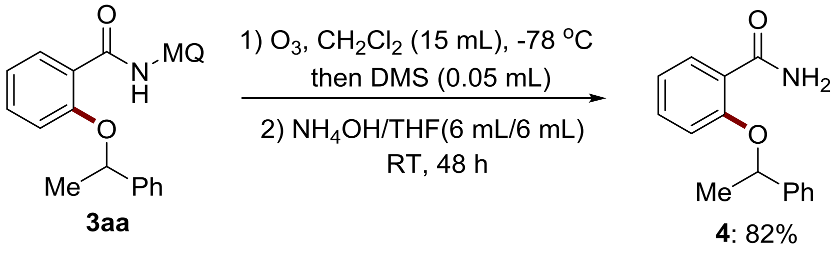

Scheme S–17: Traceless removal of the directing group to obtain primary amide **4**.

According to the literature,[10a] a stream of O_3 was passed through a solution of *N*-(6-methylquinolin-8-yl)-2-(1-phenylethoxy)benzamide (**3aa**) (76.4 mg, 0.2 mmol) in CH_2Cl_2 (15.0 mL) at -78 °C until the characteristic blue color appeared. A stream of oxygen was passed through until decolorization occurred and the mixture was quenched with dimethylsulfide (0.05 mL). After stirring at room temperature for 2.0 h, the mixture was concentrated under reduced pressure. The residue was redissolved in NH_4OH (25%)/THF (6.0 mL, 1/1) and stirred for 48 h at ambient temperature. The mixture was diluted with CH_2Cl_2 (20.0 mL) and washed with aqueous HCl (1N, 2 x 10.0 mL), and subsequently 1N NaOH (2 x 10.0 mL). The organic layer was dried with $MgSO_4$ and concentrated to dryness to give the primary amide **4** (39.7 mg, 82%). **M.p.**: 134–135 °C. **^{1}H NMR** (400 MHz, $CDCl_3$) δ = 8.23 (dd, J = 7.9, 1.9 Hz, 1H), 8.06–7.99 (m, 1H), 7.38 (d, J = 4.3 Hz, 4H), 7.35–7.28 (m, 2H), 7.02 (td, J = 7.5, 1.0 Hz, 1H), 6.85 (dd, J = 8.4, 1.0 Hz, 1H), 6.31 (s, 1H), 5.52 (q, J = 6.4 Hz, 1H), 1.77 (d, J = 6.4 Hz, 3H). **^{13}C NMR** (100 MHz, $CDCl_3$) δ = 167.3 (C_q), 156.2 (C_q), 141.6 (C_q), 133.1 (CH), 132.5 (CH), 129.0 (CH), 128.1 (CH), 125.4 (CH), 121.4 (C_q), 121.2 (CH), 114.3 (CH), 77.6 (CH), 24.4 (CH_3). **IR** (ATR): 3455, 1663, 1592, 1454, 1374, 1229, 1066, 755, 701 cm^{-1}. **MS** (ESI) *m/z* (relative intensity): 264 (100) $[M+Na]^+$, 242 (10) $[M+H]^+$, 138 (50), 117 (60). **HR-MS** (ESI) *m/z* calcd for $C_{15}H_{16}NO_2$ $[M+H]^+$: 242.1176, found: 242.1184.

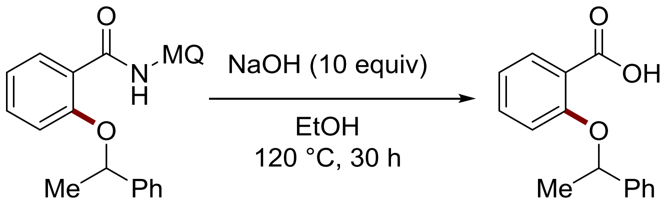

3aa **5**: 86%

Scheme S–18: Traceless removal of the directing group to obtain benzoic acid **5**.

An oven-dried 25 mL sealed tube equipped with a stir bar was charged with *N*-(6-methylquinolin-8-yl)-2-(1-phenylethoxy)benzamide (**3aa**) (95.5 mg, 0.25 mmol), NaOH (100 mg, 2.5 mmol) and EtOH (2.0 mL). The resulting mixture was stirred at 120 ℃ for 24 h. After completion, the reaction mixture was cooled down to room temperature, diluted by 20.0 mL ethyl acetate and washed by HCl (3 × 10.0 mL of 0.5 N aqueous solution). The aqueous layers were combined and extracted with EtOAc (3 × 10.0 mL). Combined organic layers were dried over $MgSO_4$. Evaporation to remove organic solvent gave the pure acid **5** (52.0 mg, 86%) as a colorless gummy. **^{1}H NMR** (400 MHz, $CDCl_3$) δ = 11.13 (s, 1H), 8.15 (dd, J = 7.8, 1.8 Hz, 1H), 7.42–7.27 (m, 6H), 7.06–7.02 (m, 1H), 6.88 (dd, J = 8.4, 0.9 Hz, 1H), 5.57 (q, J = 6.4 Hz, 1H), 1.79 (d, J = 6.4 Hz, 3H). **^{13}C NMR** (100 MHz, $CDCl_3$) δ = 165.5 (C_q), 156.5 (C_q), 140.4 (C_q), 134.7 (CH), 133.7 (CH), 129.1 (CH), 128.5 (CH), 125.4 (CH), 122.2 (CH), 118.3 (C_q), 114.4 (CH), 79.3 (CH), 24.0 (CH_3). **IR** (ATR): 3252, 2979, 1732, 1692, 1600, 1455, 1241, 1065, 753, 700 cm^{-1} **MS** (ESI) *m/z* (relative intensity): 265 (100) $[M+Na]^+$. **HR-MS** (ESI) *m/z* calcd for $C_{15}H_{14}O_3Na^+$ $[M+Na]^+$: 265.0835, found: 265.0834. The analytical data correspond with those reported in the literature.[10]

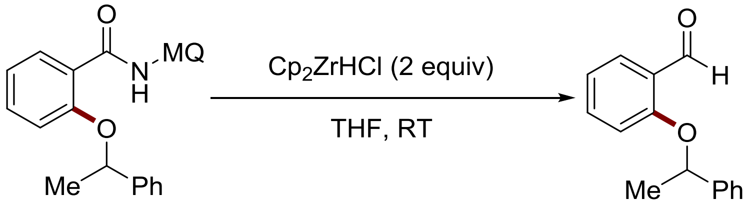

3aa

6: 72%

Scheme S–19: Traceless removal of the directing group to obtain aromatic aldehyde **6**.

According the literature,[10b] *N*-(6-methylquinolin-8-yl)-2-(1-phenylethoxy)benzamide (**3aa**) (95.5 mg, 0.25 mmol), Cp_2ZrHCl (0.50 mmol, 125 mg), and THF (2.0 mL) were charged to a 25 mL Schlenk tube under N_2. The reaction mixture was stirred at room temperature for 6 hrs before carefully being quenched by saturated ammonium chloride at 0 ℃. After being extracted with CH_2Cl_2 (3 × 25.0 mL), the combined organic extract was washed with brine, dried over Na_2SO_4, and concentrated in vacuum. The residue was purified by column chromatography on silica gel (eluent: *n*-hexane/EtOAc: 20/1) to afford aromatic aldehyde **6** as a colorless oil (40.7 mg, 72%). **^{1}H NMR** (400 MHz, $CDCl_3$) δ = 10.64 (d, *J* = 0.8 Hz, 1H), 7.80 (dd, *J* = 7.7, 1.8 Hz, 1H), 7.41–7.29 (m, 5H), 7.30–7.24 (m, 1H), 7.00–6.87 (m, 1H), 6.82 (dd, *J* = 8.5, 0.8 Hz, 1H), 5.42 (q, *J* = 6.4 Hz, 1H), 1.69 (d, *J* = 6.4 Hz, 3H). **^{13}C NMR** (100 MHz, $CDCl_3$) δ = 189.9 (CH), 160.4 (C_q), 142.1 (C_q), 135.6 (CH), 128.8 (CH), 128.2 (CH), 127.8 (CH), 125.4 (CH), 125.4 (C_q), 120.7 (CH), 114.5 (CH), 77.0 (CH), 24.2 (CH_3). **IR** (ATR): 2979, 1683, 1596, 1478, 1455, 1235, 1067, 755, 699 cm^{-1}. **MS** (ESI) *m/z* (relative intensity): 249 (100) $[M+Na]^+$, 105 (25). **HR-MS** (ESI) *m/z* calcd for $C_{15}H_{14}O_2Na$ $[M+Na]^+$: 249.0886, found: 249.08867. The analytical data correspond with those reported in the literature.[10]

Cyclic voltammetry

A glassy-carbon electrode (3 mm diameter, disc-electrode) was used as the working electrode, a Pt–wire as auxiliary electrode and an Ag–wire as pseudoreference. The measurements were then referenced using ferrocene. The measurements were carried out at a scan rate of 100 mV/s.

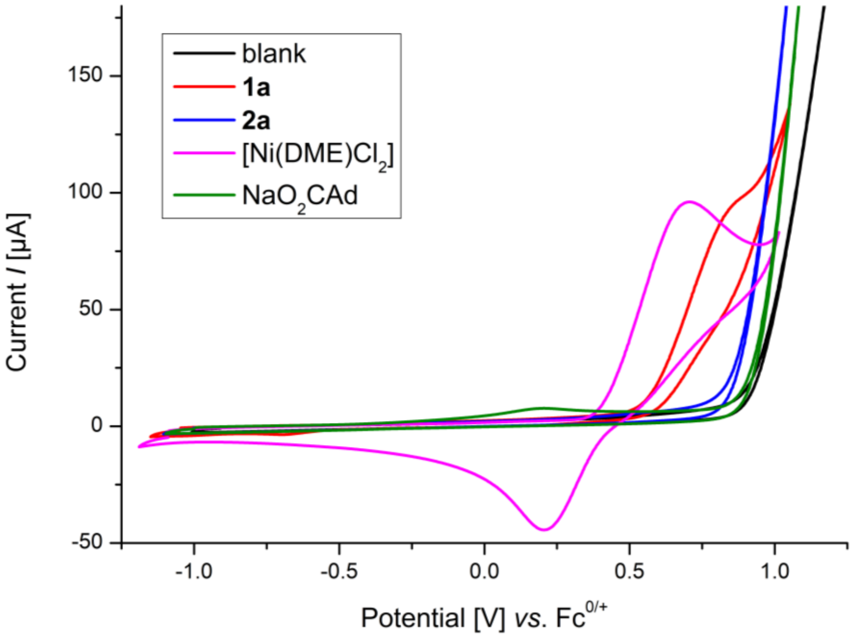

Figure S–22: Cyclic voltammograms at 100 mV/s. nBu$_4$NPF$_6$ (0.1 M in DMA); concentration of substrates 5 mM. blank (black); **1a** (red); **2a** (blue); [Ni(DME)Cl$_2$] (purple); NaO$_2$CAd (green).

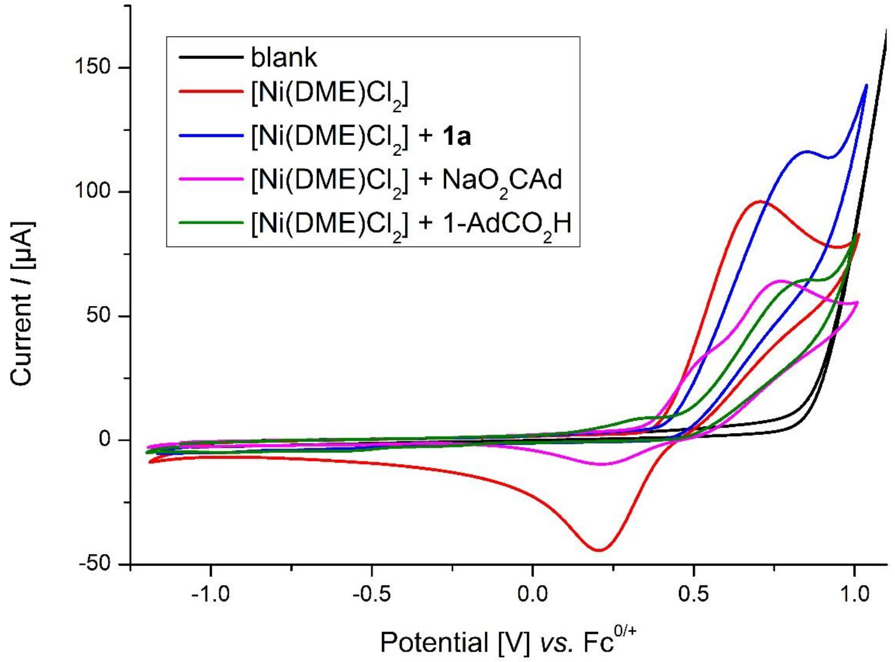

Figure S–23: Cyclic voltammograms at 100 mV/s. *n*Bu_4NPF_6 (0.1 M in DMA); concentration of substrates 5 mM. blank (black); Ni(DME)Cl_2 (red); Ni(DME)Cl_2 + **1a** (blue); Ni(DME)Cl_2 + NaO_2CAd (purple); Ni(DME)Cl_2 + 1-AdCO_2H (green).

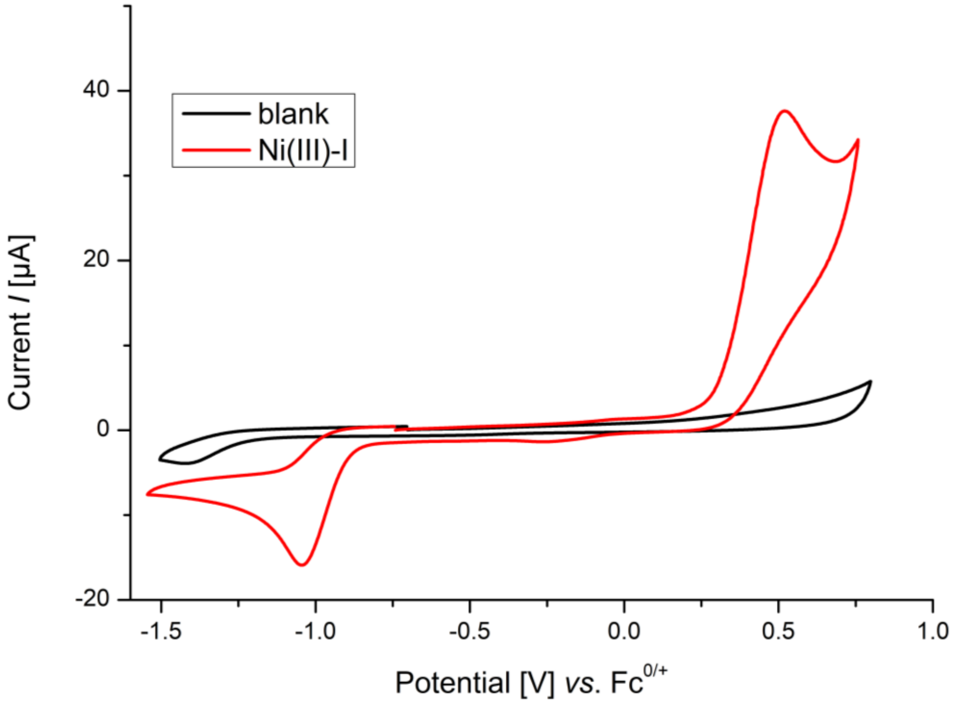

Figure S–24: Cyclic voltammograms at 100 mV/s. *n*Bu_4NPF_6 (0.1 M in DMA); concentration of substrates 5 mM. blank (black); **Ni(III)-I** complex (red).

References

[1] a) M. Elsherbini, T. Wirth, *Acc. Chem. Res.* **2019**, DOI: 10.1021/acs.accounts.9b00497; b) W.-J. Kong, L. H. Finger, A. M. Messinis, R. Kuniyil, J. C. A. Oliveira, L. Ackermann, *J. Am. Chem. Soc.* **2019**, *141*, 17198-17206.

[2] a) D. Kalsi, N. Barsu, B. Sundararaju, *Chem. Eur. J.* **2018**, *24*, 2360–2364; b) Q. Yan, Z. Chen, W. Yu, H. Yin, Z. Liu, Y. Zhang, *Org. Lett.* **2015**, *17*, 2482–2485; c) L.-B. Zhang, X.-Q. Hao, S.-K. Zhang, Z.-J. Liu, X.-X. Zheng, J.-F. Gong, J.-L. Niu, M.-P. Song, *Angew. Chem. Int. Ed.* **2015**, *54*, 272–275; d) Y. Aihara, M. Tobisu, Y. Fukumoto, N. Chatani, *J. Am. Chem. Soc.* **2014**, *136*, 15509–15512; e) L. D. Tran, J. Roane, O. Daugulis, *Angew. Chem. Int. Ed.* **2013**, *52*, 6043–6046.

[3] a) N. Sauermann, T. H. Meyer, C. Tian, L. Ackermann, *J. Am. Chem. Soc.* **2017**, *139*, 18452–18455; b) Z.-L. Li, K.-K. Sun, C. Cai, *Org. Chem. Front.* **2019**, *6*, 637–642; c) N. Rajesh, B. Sundararaju, *Asian. J. Org. Chem.* **2018**, *7*, 1368–1371; d) S. Bhadra, C. Matheis, D. Katayev, L. J. Gooßen, *Angew. Chem. In. Ed.* **2013**, *52*, 9279–9283; e) J. Roane, O. Daugulis, *Org. Lett.* **2013**, *15*, 5842–5845; f) Z. Yin, X. Jiang, P. Sun, *J. Org. Chem.* **2013**, *78*, 10002–10007; g) C. Zhang, P. Sun, *J. Org. Chem.* **2014**, *79*, 8457−8461; h) F. Péron, C. Fossey, J. Sopkova-de Oliveira Santos, T. Cailly, F. Fabis, *Chem. Eur. J.* **2014**, *20*, 7507–7513.

[4] O. V. Dolomanov, L. J. Bourhis, R. J. Gildea, J. A. K. Howard, H. Puschmann, *J. Appl. Crystallogr.* **2009**, *42*, 339–341.

[5] G. Sheldrick, *Acta Crystallogr. A* **2015**, *71*, 3–8.

[6] G. Sheldrick, *Acta Crystallogr. A* **2008**, *64*, 112–122.

[7] Z. He, Y. Huang, *ACS Catal.* **2016**, *6*, 7814–7823.

[8] a) C. Adamo, V. Barone, *J. Chem. Phys.* **1999**, *110*, 6158–6170; b) F. Weigend, R. Ahlrichs, *Phys. Chem. Chem. Phys.* **2005**, *7*, 3297–3305; c) S. Grimme, J. Antony, S. Ehrlich, H. Krieg, *J. Chem. Phys.* **2010**, *132*, 154104; d) S. Grimme, S. Ehrlich, L. Goerigk, *J. Compt. Chem.* **2011**, *32*, 1456–1465; e) A. V. Marenich, C. J. Cramer, D. G. Truhlar, *J. Phys. Chem. B* **2009**, *113*, 6378–6396; f) R. S. Mulliken,

J. Chem. Phys. **1955**, *23*, 1833–1840; g) M. J. Frisch, G. W. Trucks, H. B. Schlegel, G. E. Scuseria, M. A. Robb, J. R. Cheeseman, G. Scalmani, V. Barone, G. A. Petersson, H. Nakatsuji, X. Li, M. Caricato, A. V. Marenich, J. Bloino, B. G. Janesko, R. Gomperts, B. Mennucci, H. P. Hratchian, J. V. Ortiz, A. F. Izmaylov, J. L. Sonnenberg, D. Williams-Young, F. Ding, F. Lipparini, F. Egidi, J. Goings, B. Peng, A. Petrone, T. Henderson, D. Ranasinghe, V. G. Zakrzewski, J. Gao, N. Rega, G. Zheng, W. Liang, M. Hada, M. Ehara, K. Toyota, R. Fukuda, J. Hasegawa, M. Ishida, T. Nakajima, Y. Honda, O. Kitao, H. Nakai, T. Vreven, K. Throssell, J. A. Montgomery, Jr., J. E. Peralta, F. Ogliaro, M. J. Bearpark, J. J. Heyd, E. N. Brothers, K. N. Kudin, V. N. Staroverov, T. A. Keith, R. Kobayashi, J. Normand, K. Raghavachari, A. P. Rendell, J. C. Burant, S. S. Iyengar, J. Tomasi, M. Cossi, J. M. Millam, M. Klene, C. Adamo, R. Cammi, J. W. Ochterski, R. L. Martin, K. Morokuma, O. Farkas, J. B. Foresman, and D. J. Fox, *Gaussian 16, Revision A.03*, Gaussian, Inc., Wallingford CT, **2016.**

[9] a) M. Berger, R. Chauhan, C. A. B. Rodrigues, N. Maulide, *Chem. Eur.J.* **2016**, *22*, 16805–16808; b) V. G. Landge, C. H. Shewale, G. Jaiswal, M. K. Sahoo, S. P. Midya, E. Balaraman, *Catal. Sci. Technol.* **2016**, *6*, 1946–1951.

[10] a) S. E. Barber, K. E.S. Dean, A. J. Kirby, *Can. J. Chem.* **1999**, *77*, 792–801; b) W. Kirmse, W. Konrad, I. S. Özkir, *Tetrahedron* **1997**, *53*, 9935–9964; c) W. Kirmse, I. S. Özkir, *J. Am. Chem. Soc.* **1992**, *114*, 7590–7591.

NMR spectra

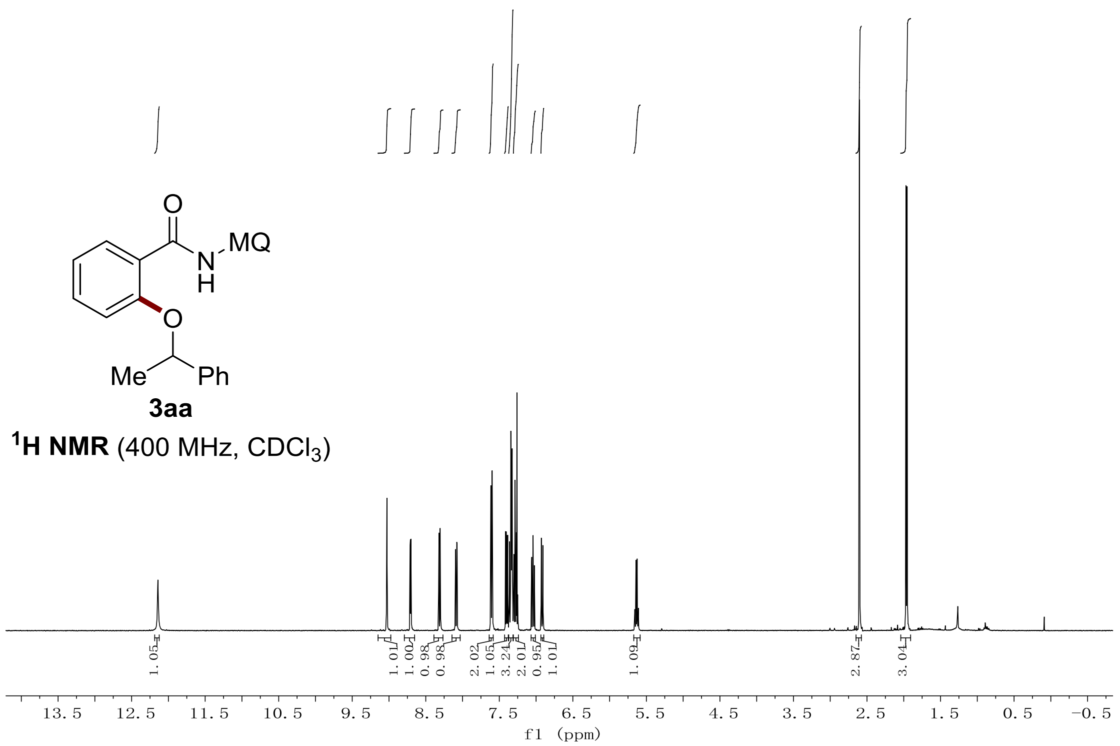

MQ
N
H
O
Me
Ph
3aa
1H NMR (400 MHz, CDCl3)
1.05
1.01
1.00
0.98
0.98
2.02
1.05
3.24
2.01
0.95
1.01
1.09
2.87
3.04
f1 (ppm)

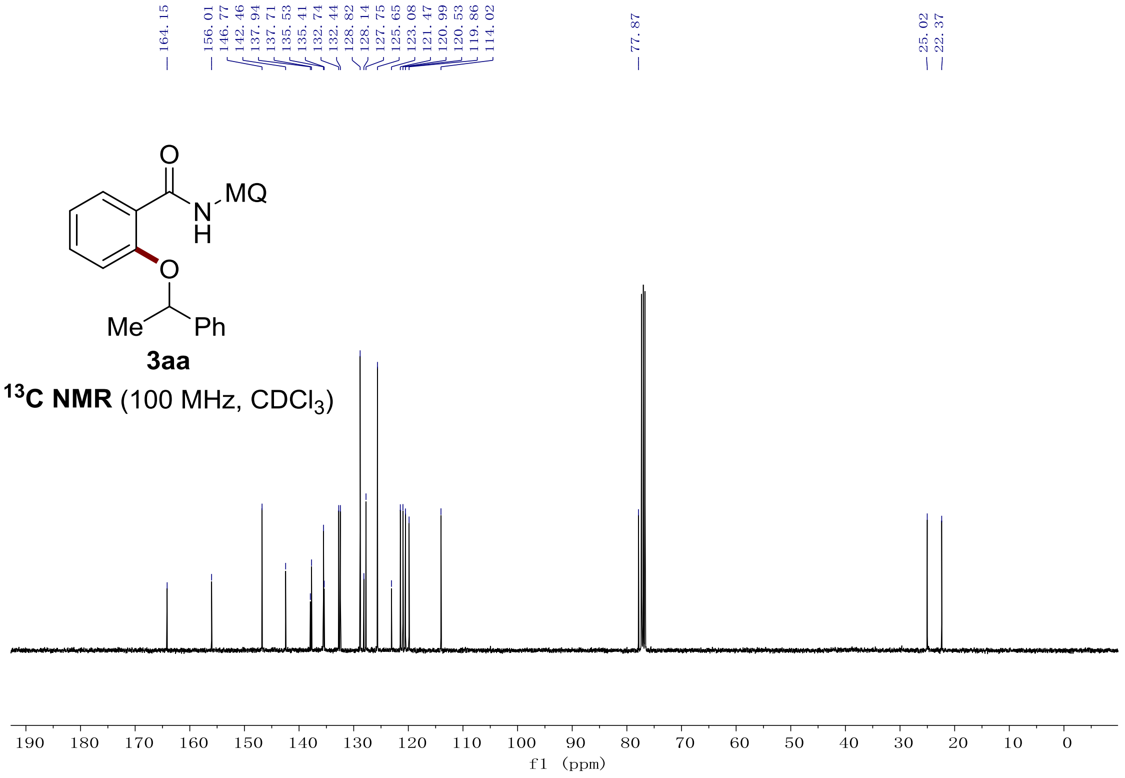

164.15
156.01
146.77
142.46
137.94
137.71
135.53
135.41
132.74
132.44
128.82
128.14
127.75
125.65
123.08
121.47
120.99
120.53
119.86
114.02
77.87
25.02
22.37
MQ
N
H
O
Me
Ph
3aa
13C NMR (100 MHz, CDCl3)
f1 (ppm)

3ab

^{1}H NMR (300 MHz, $CDCl_3$)

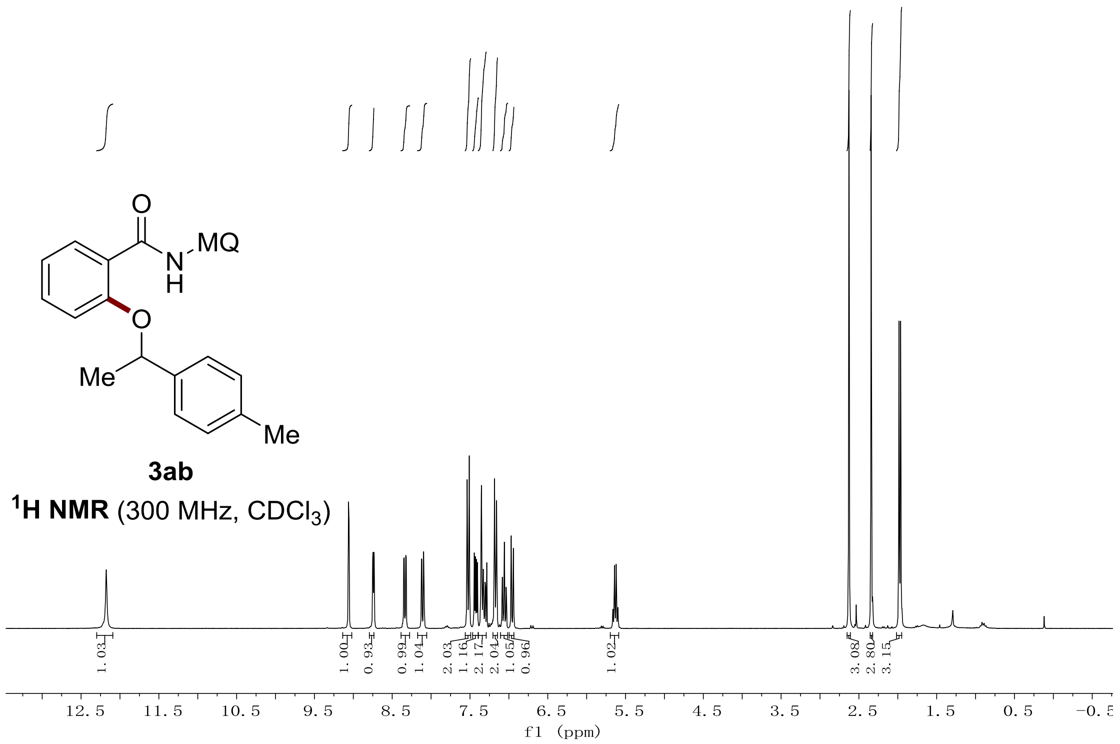

3ab

^{13}C NMR (75 MHz, $CDCl_3$)

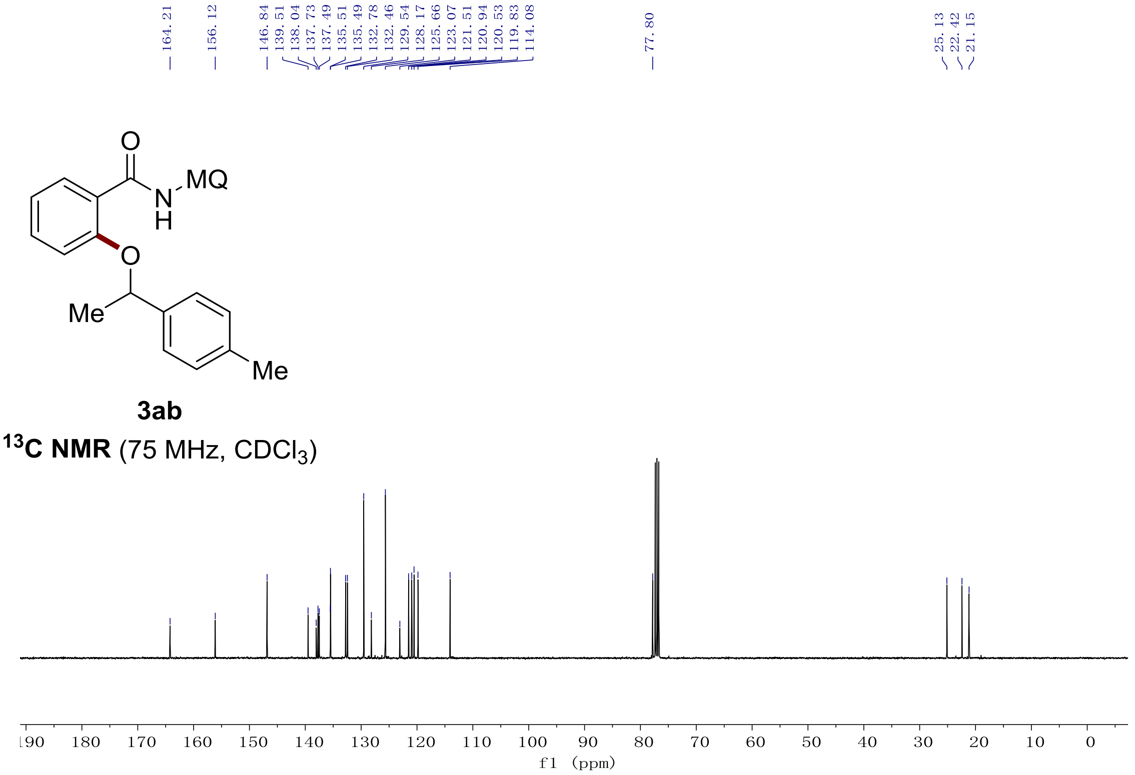

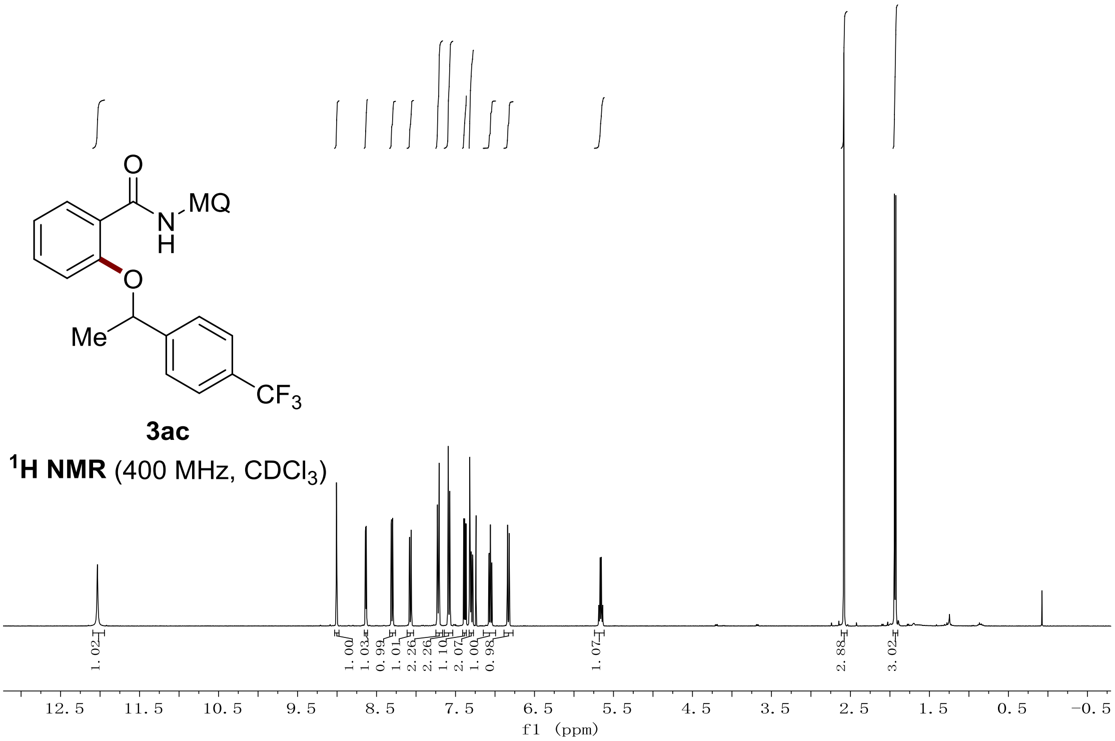
MQ
Me
CF3
3ac
1H NMR (400 MHz, CDCl3)
f1 (ppm)

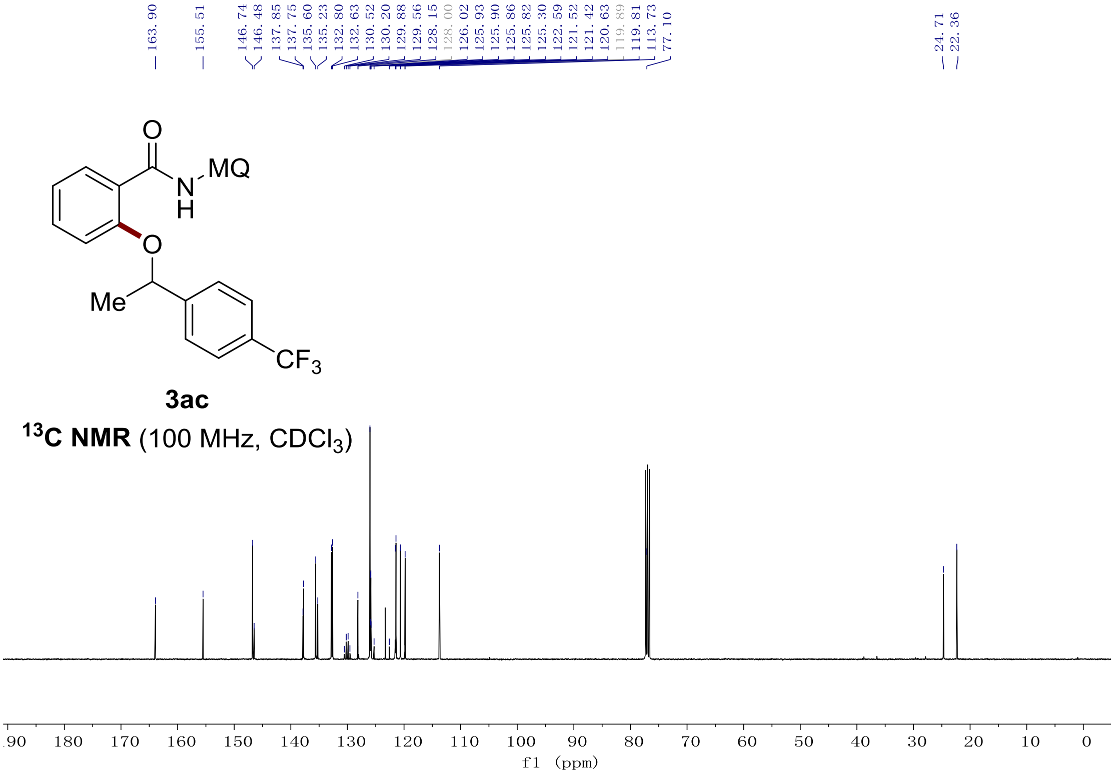
163.90
155.51
146.74
146.48
137.85
137.75
135.60
135.23
132.80
132.63
130.52
130.20
129.88
129.56
128.15
128.00
126.02
125.93
125.90
125.86
125.82
125.30
122.59
121.52
121.42
120.63
119.89
119.81
113.73
77.10
24.71
22.36
MQ
Me
CF3
3ac
13C NMR (100 MHz, CDCl3)
f1 (ppm)

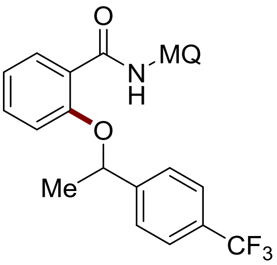

3ac

^{19}F NMR (282 MHz, $CDCl_3$)

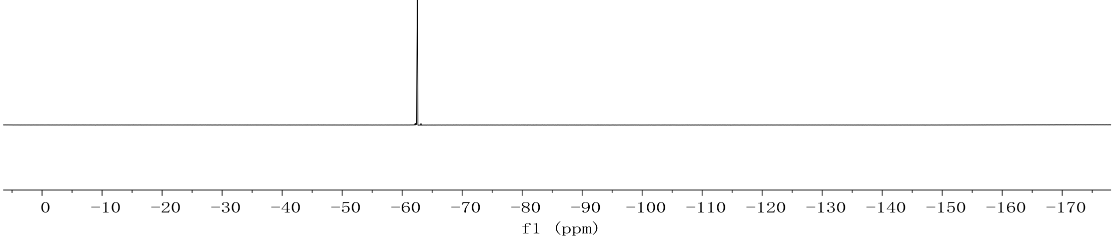

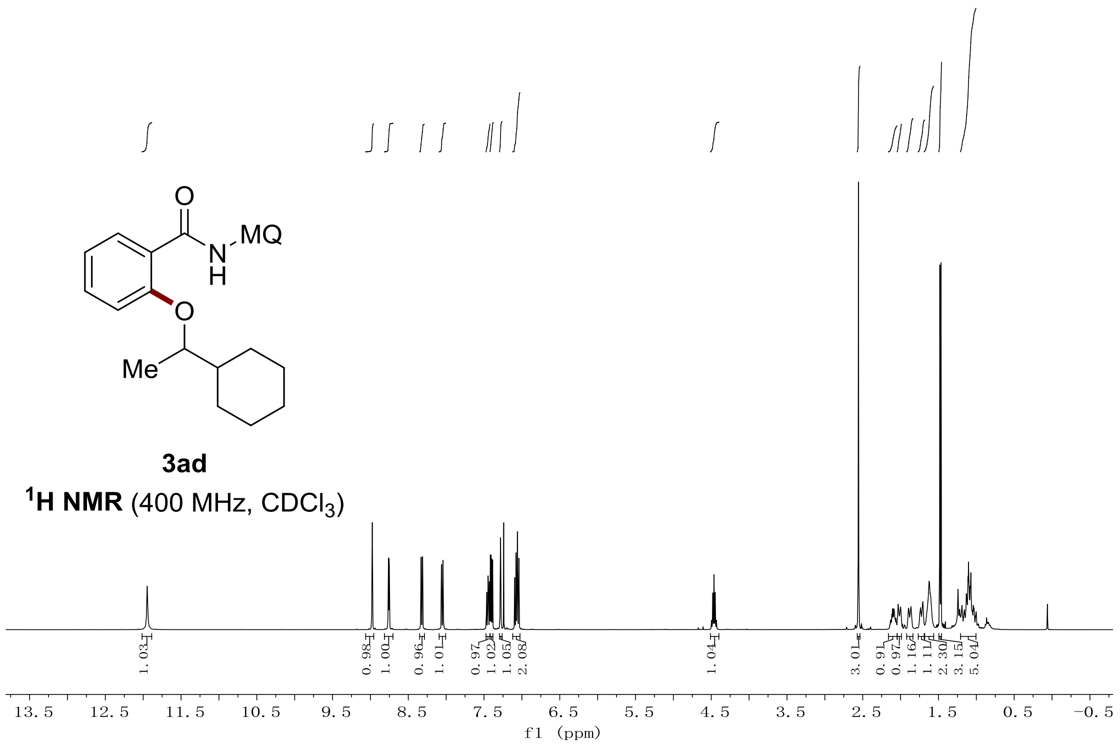

3ad

^{1}H NMR (400 MHz, $CDCl_3$)

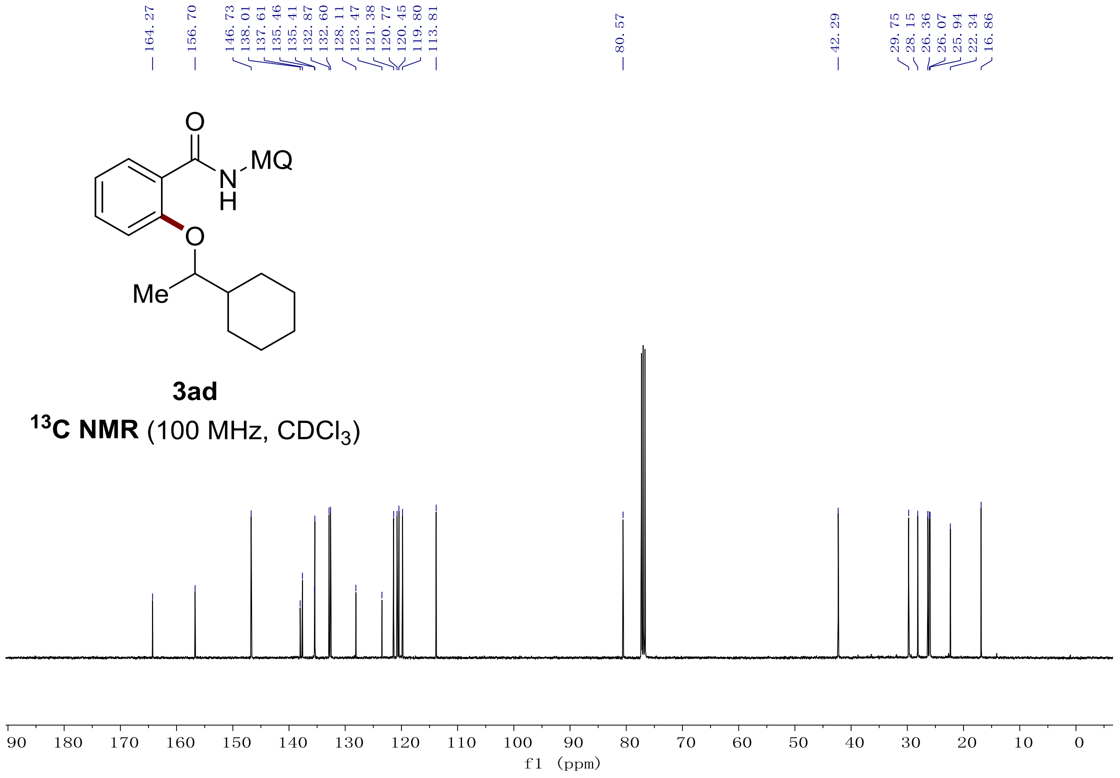

3ad

^{13}C NMR (100 MHz, $CDCl_3$)

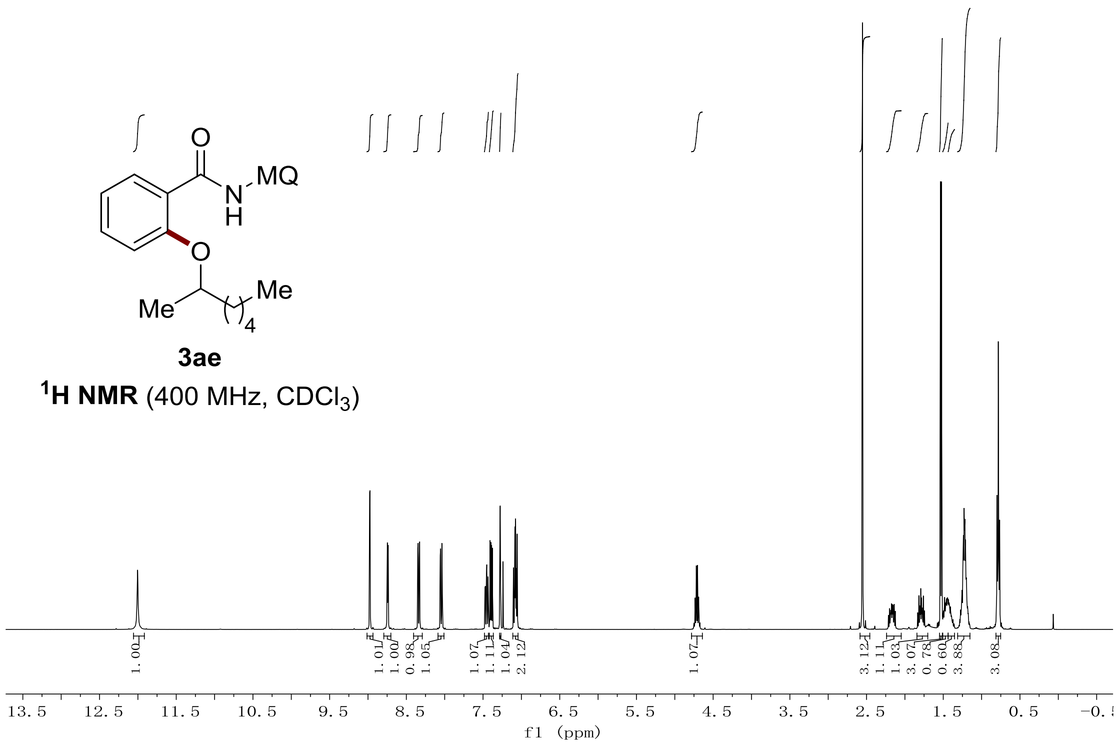
MQ
3ae
1H NMR (400 MHz, CDCl3)
1.00 1.01 1.00 0.98 1.05 1.07 1.11 1.04 2.12 1.07 3.12 1.11 1.03 3.07 0.78 0.60 3.88 3.08
f1 (ppm)

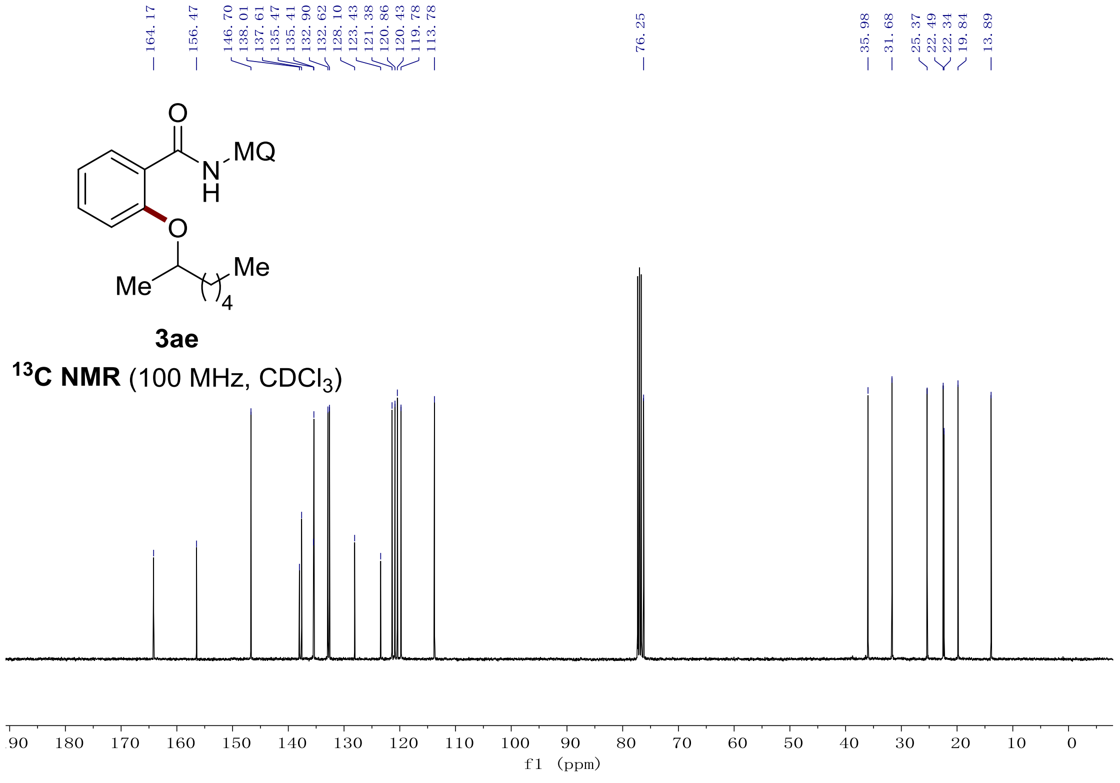
164.17
156.47
146.70
138.01
137.61
135.47
135.41
132.90
132.62
128.10
123.43
121.38
120.86
120.43
119.78
113.78
76.25
35.98
31.68
25.37
22.49
22.34
19.84
13.89
MQ
3ae
13C NMR (100 MHz, CDCl3)
f1 (ppm)

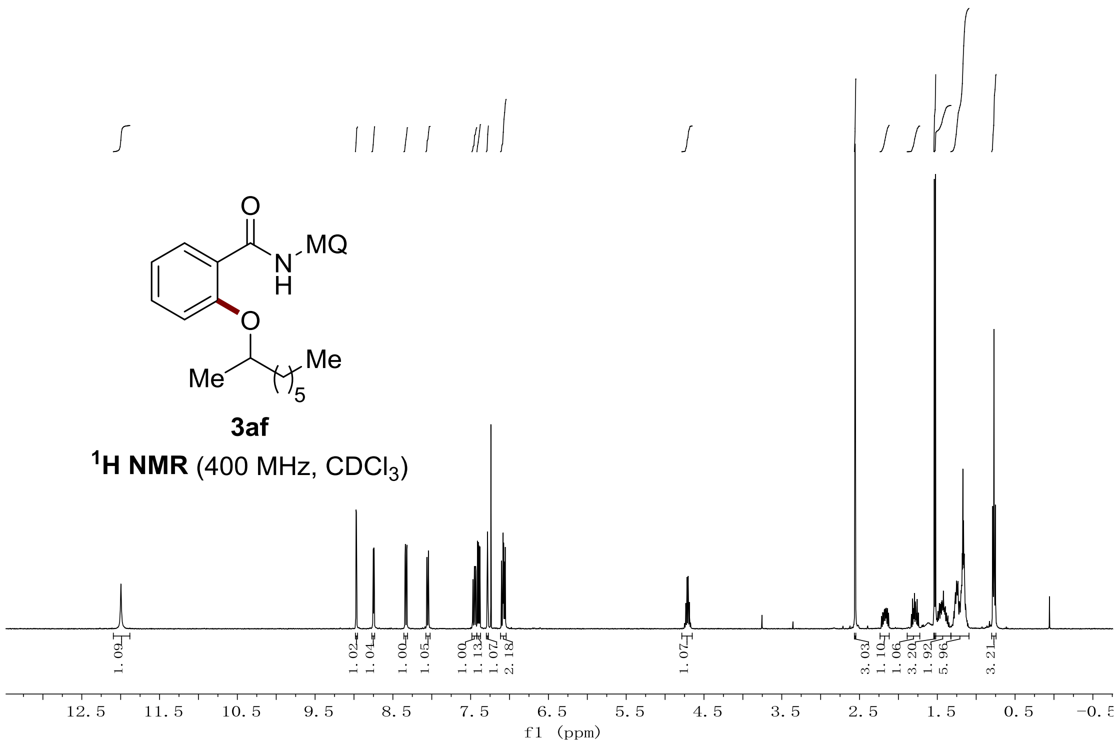
MQ
Me
Me
3af
1H NMR (400 MHz, CDCl3)
f1 (ppm)

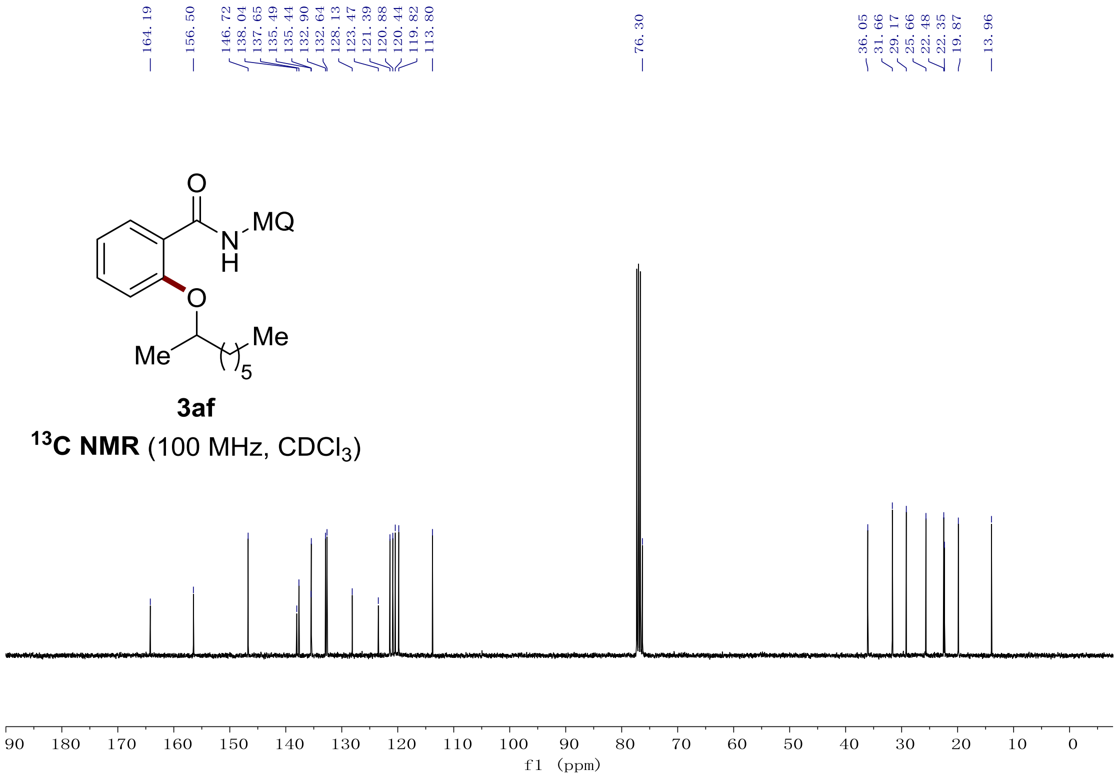
164.19
156.50
146.72
138.04
137.65
135.49
135.44
132.90
132.64
128.13
123.47
121.39
120.88
120.44
119.82
113.80
76.30
36.05
31.66
29.17
25.66
22.48
22.35
19.87
13.96
MQ
Me
Me
3af
13C NMR (100 MHz, CDCl3)
f1 (ppm)

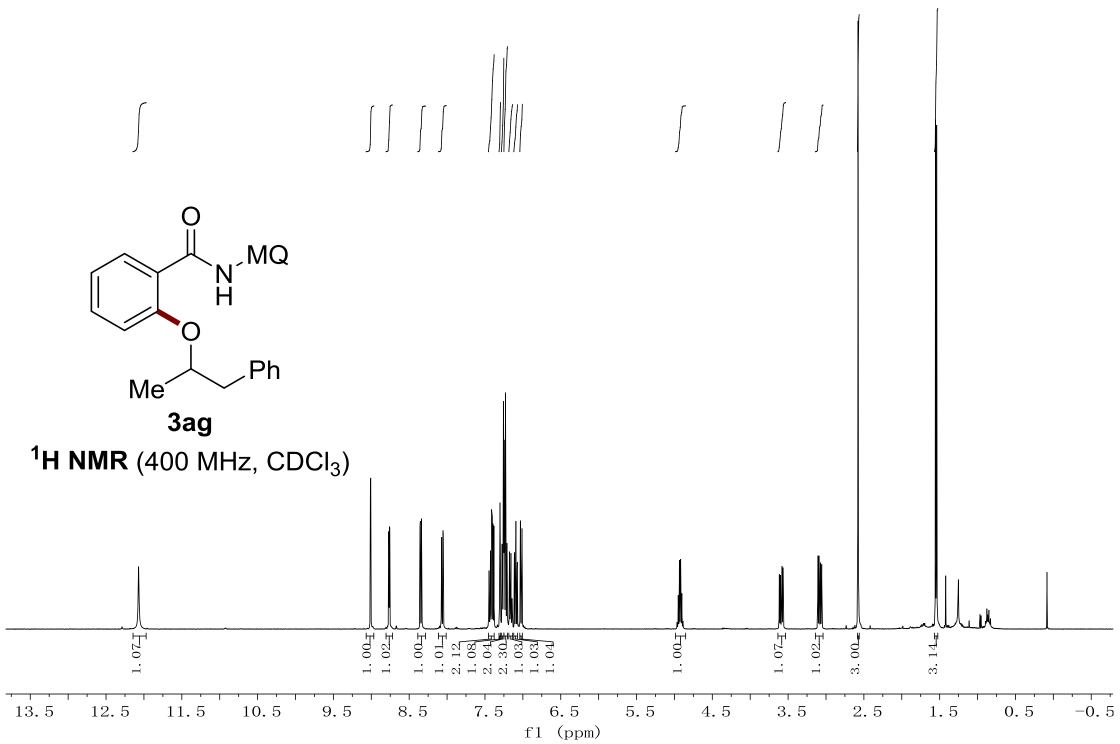

O
N
H
MQ
O
Me
Ph
3ag
1H NMR (400 MHz, CDCl3)
f1 (ppm)

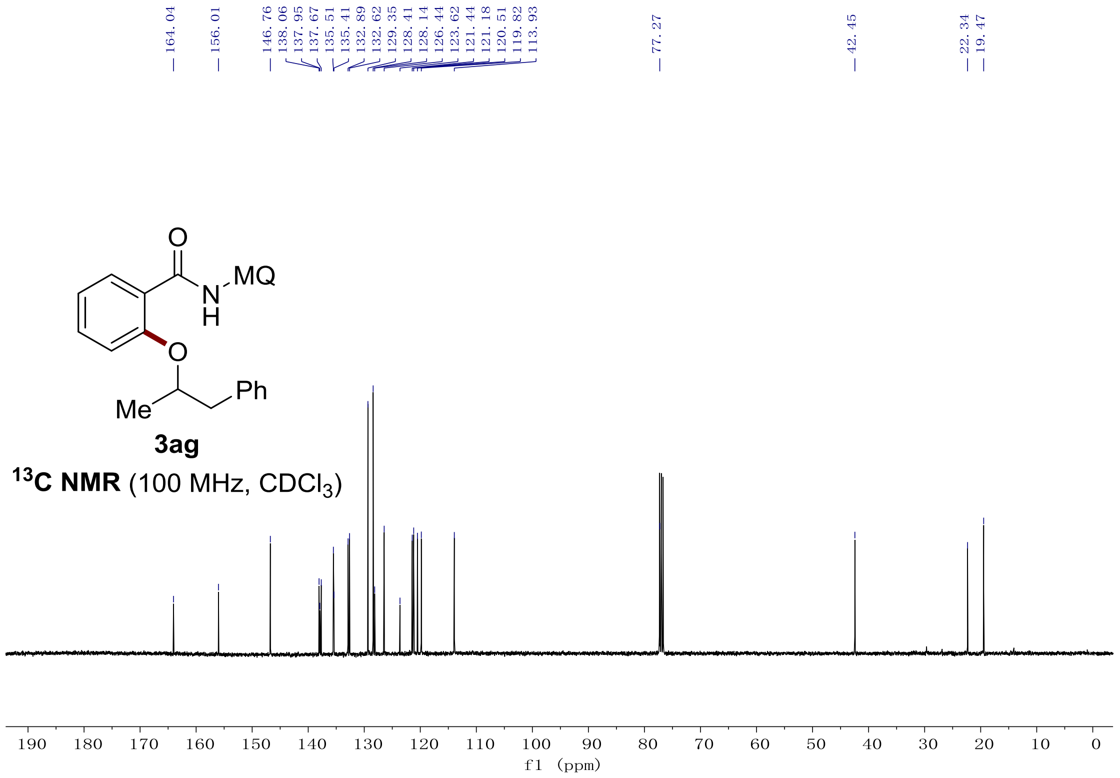

164.04
156.01
146.76
138.06
137.95
137.67
135.51
135.41
132.89
132.62
129.35
128.41
128.14
126.44
123.62
121.44
121.18
120.51
119.82
113.93
77.27
42.45
22.34
19.47
O
N
H
MQ
O
Me
Ph
3ag
13C NMR (100 MHz, CDCl3)
f1 (ppm)

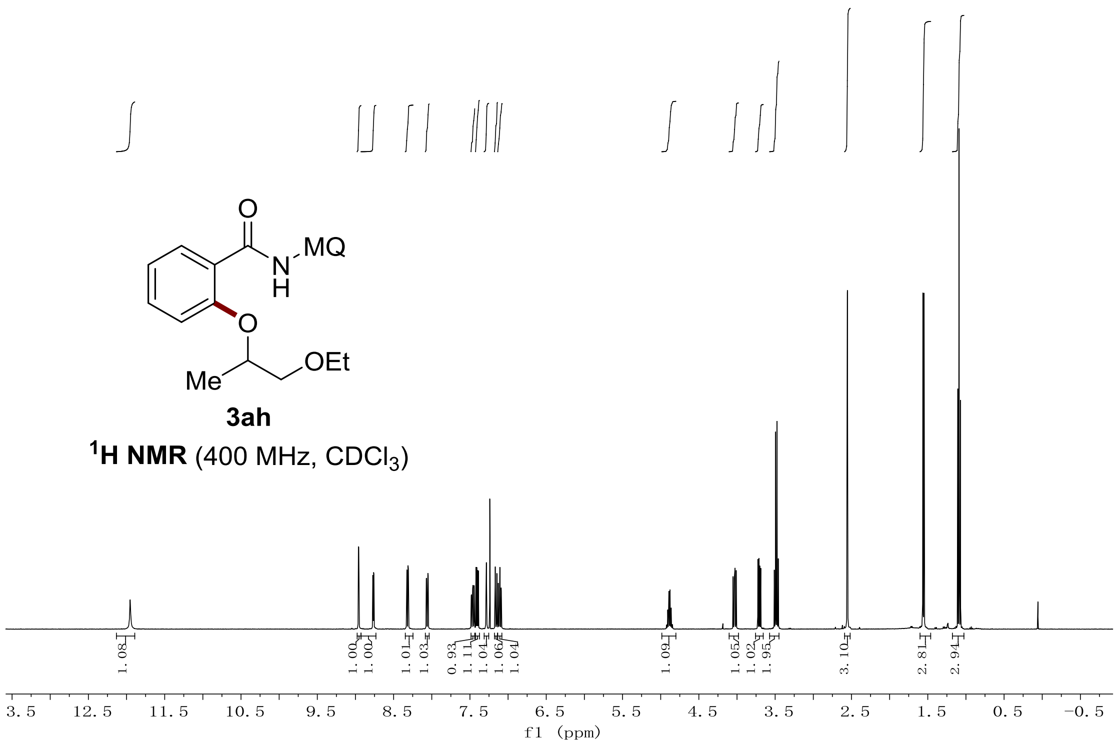
MQ
N
H
O
OEt
Me
3ah
^{1}H NMR (400 MHz, $CDCl_3$)
1.08
1.00
1.00
1.01
1.03
0.93
1.11
1.04
1.06
1.04
1.09
1.05
1.02
1.95
3.10
2.81
2.94
f1 (ppm)

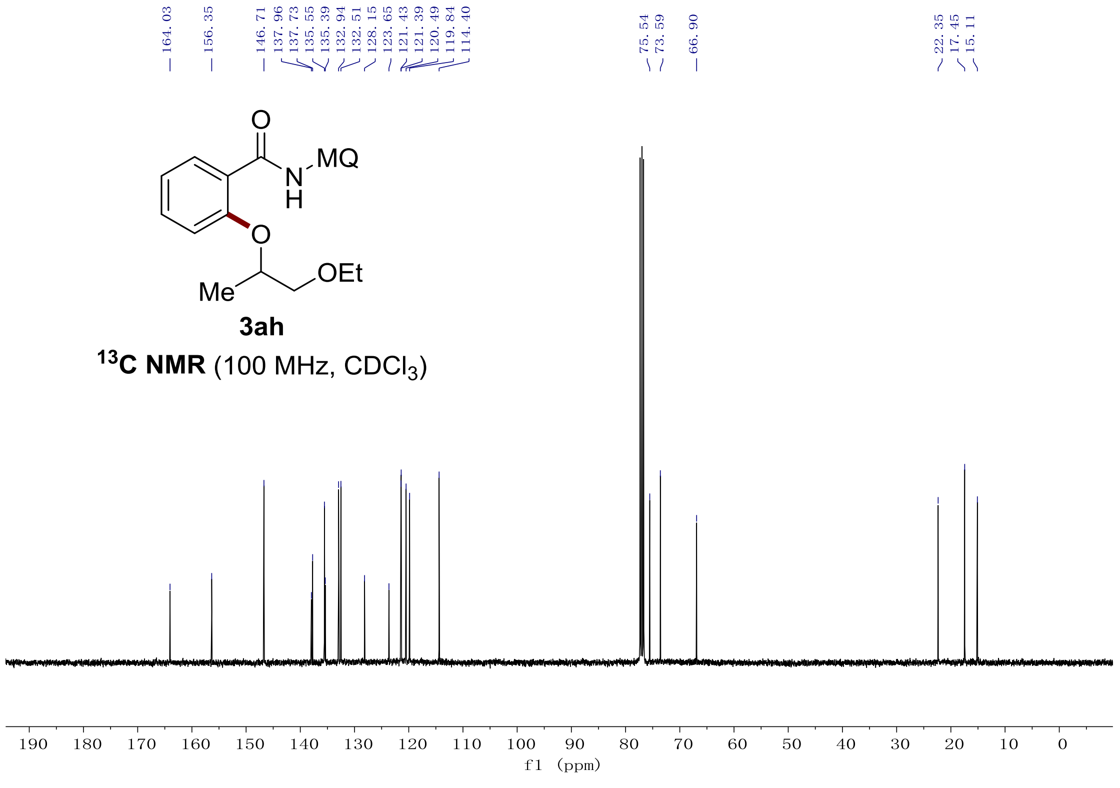
164.03
156.35
146.71
137.96
137.73
135.55
135.39
132.94
132.51
128.15
123.65
121.43
121.39
120.49
119.84
114.40
75.54
73.59
66.90
22.35
17.45
15.11
MQ
N
H
O
OEt
Me
3ah
^{13}C NMR (100 MHz, $CDCl_3$)
f1 (ppm)

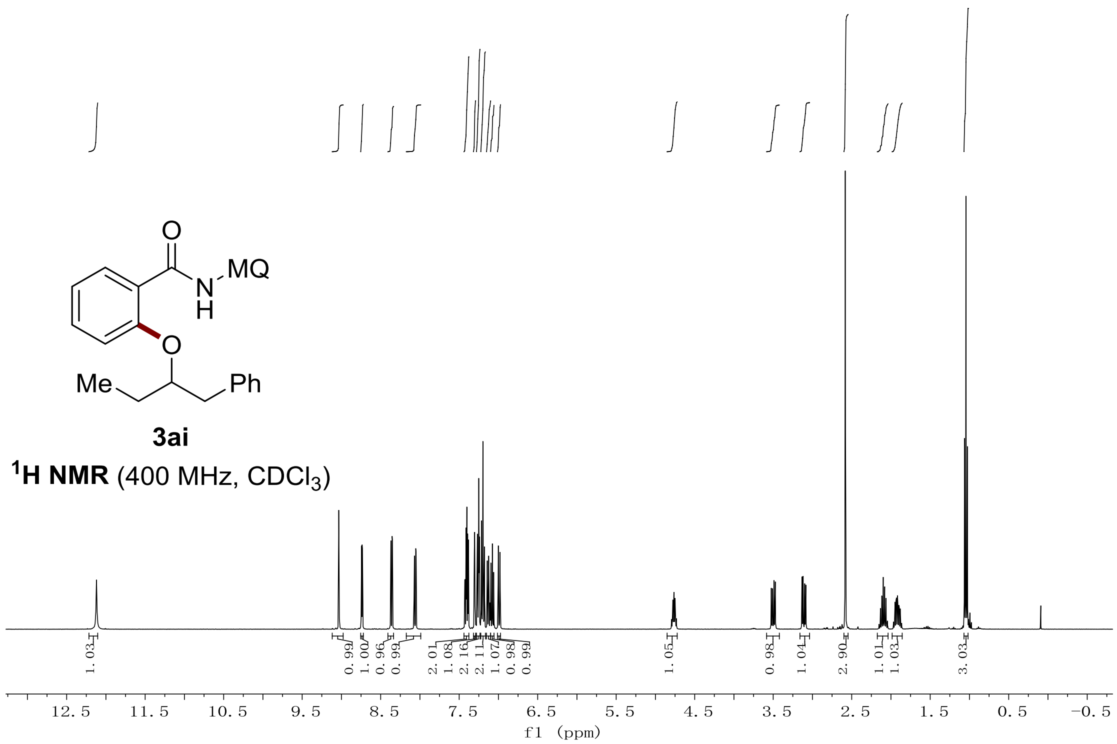
O
N
MQ
H
O
Me
Ph
3ai
^{1}H NMR (400 MHz, CDCl$_3$)
1.03
0.99
1.00
0.96
0.99
2.01
1.08
2.16
2.11
1.07
0.98
0.99
1.05
0.98
1.04
2.90
1.01
1.03
3.03
f1 (ppm)

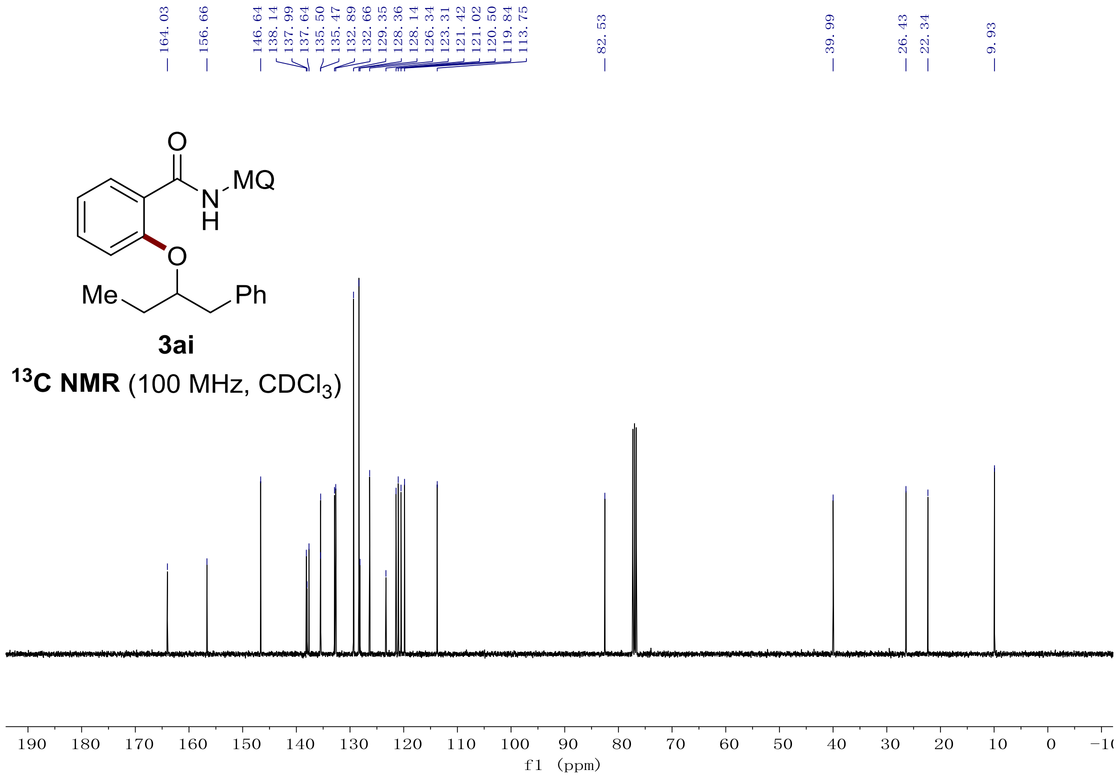
164.03
156.66
146.64
138.14
137.99
137.64
135.50
135.47
132.89
132.66
129.35
128.36
128.14
126.34
123.31
121.42
121.02
120.50
119.84
113.75
82.53
39.99
26.43
22.34
9.93
O
N
MQ
H
O
Me
Ph
3ai
^{13}C NMR (100 MHz, CDCl$_3$)
f1 (ppm)

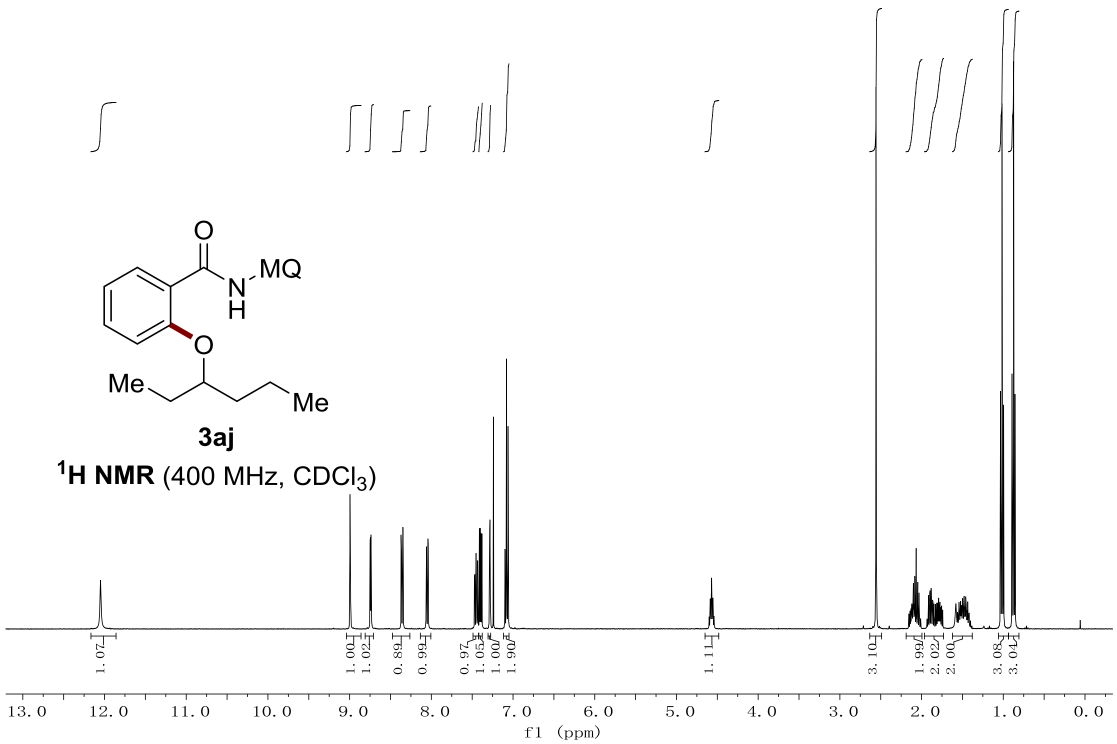

3aj
^{1}H NMR (400 MHz, $CDCl_3$)

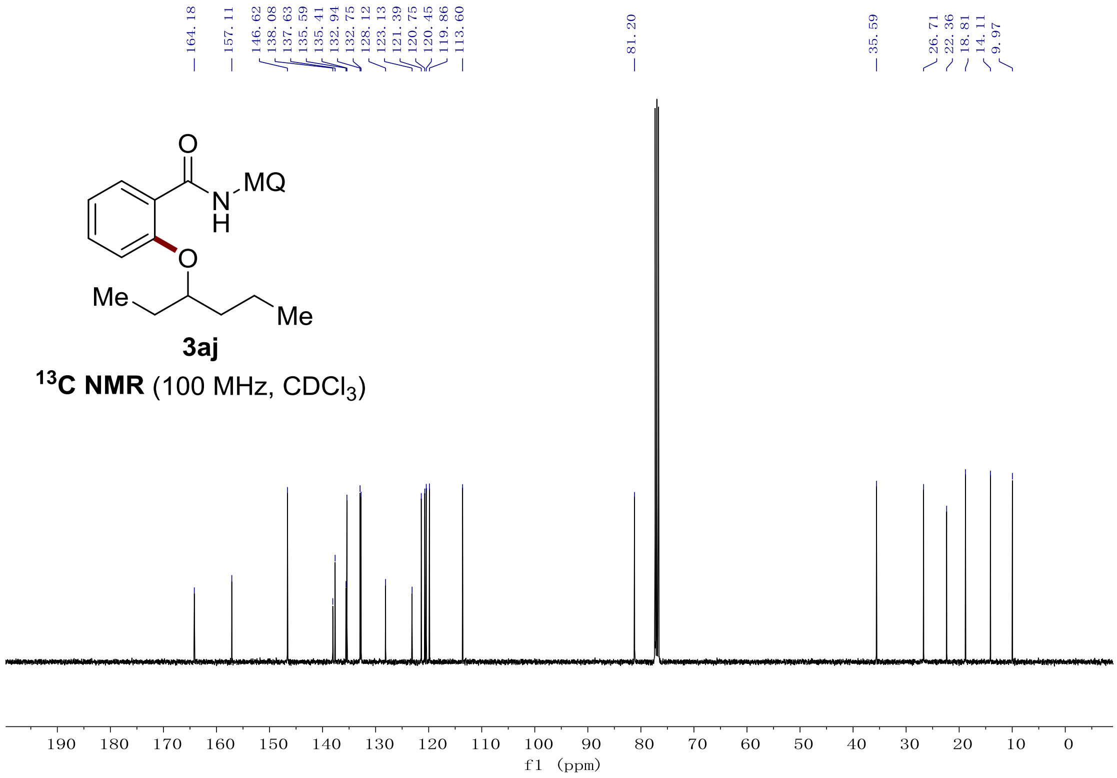

3aj
^{13}C NMR (100 MHz, $CDCl_3$)

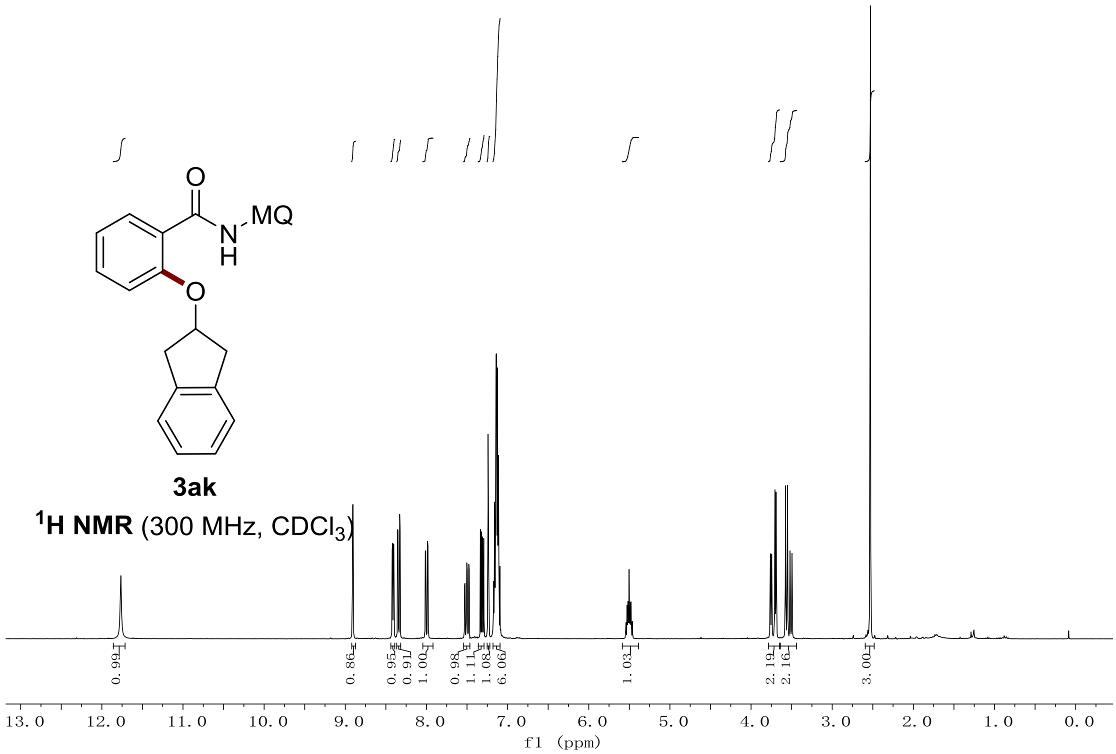

3ak

^{1}H NMR (300 MHz, $CDCl_3$)

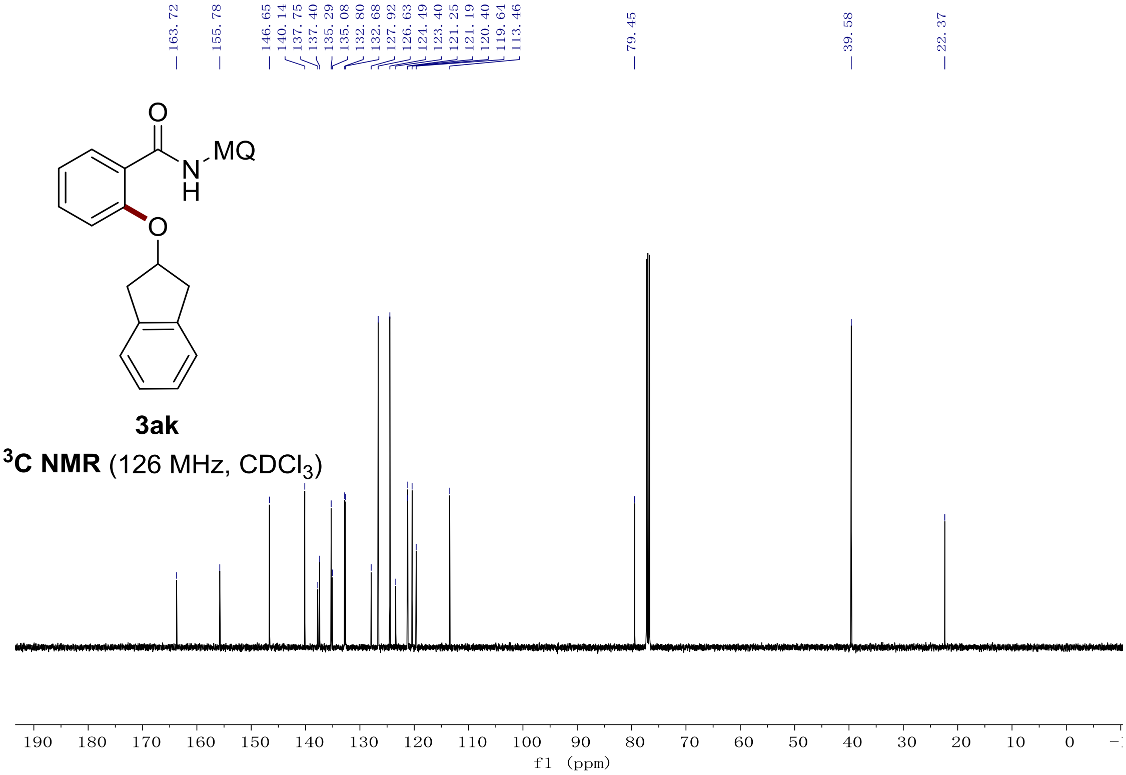

3ak

^{13}C NMR (126 MHz, $CDCl_3$)

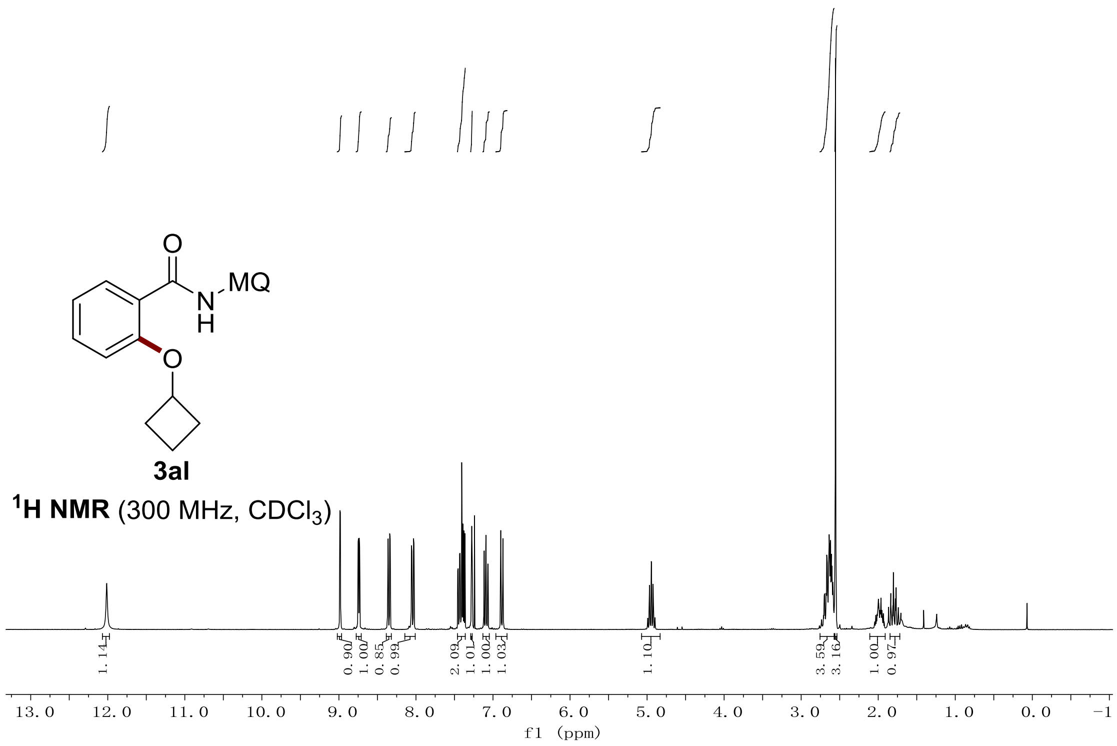

3al

^{1}H NMR (300 MHz, $CDCl_3$)

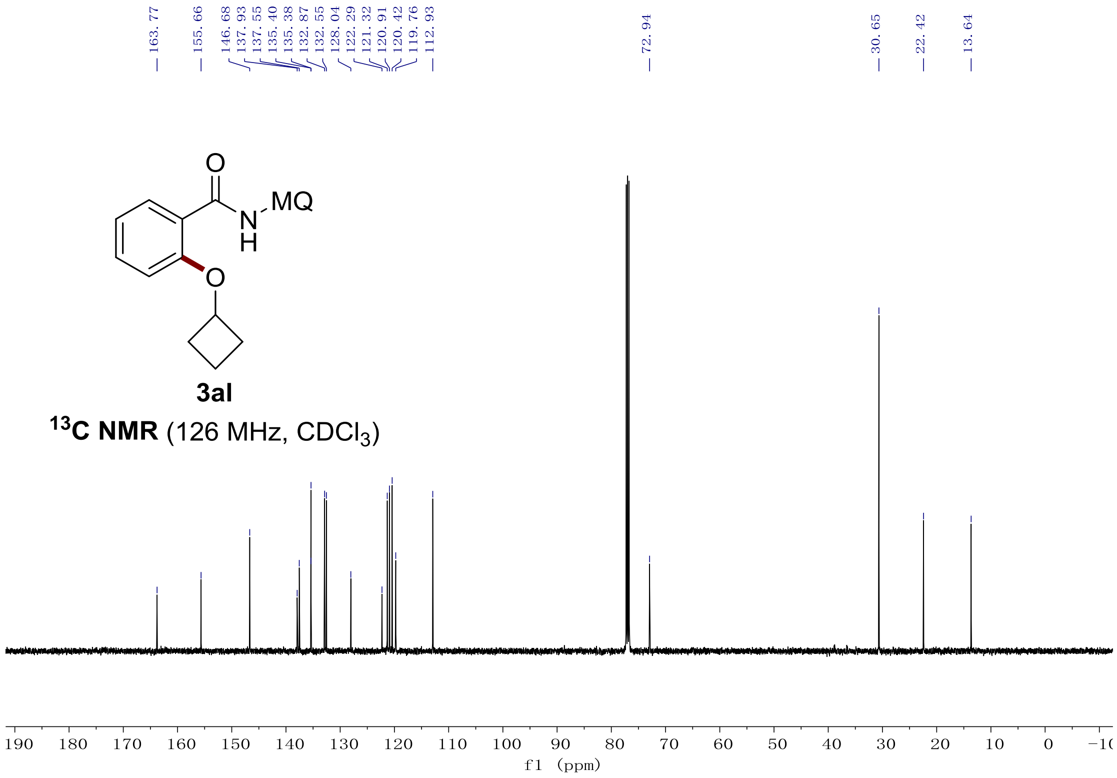

3al

^{13}C NMR (126 MHz, $CDCl_3$)

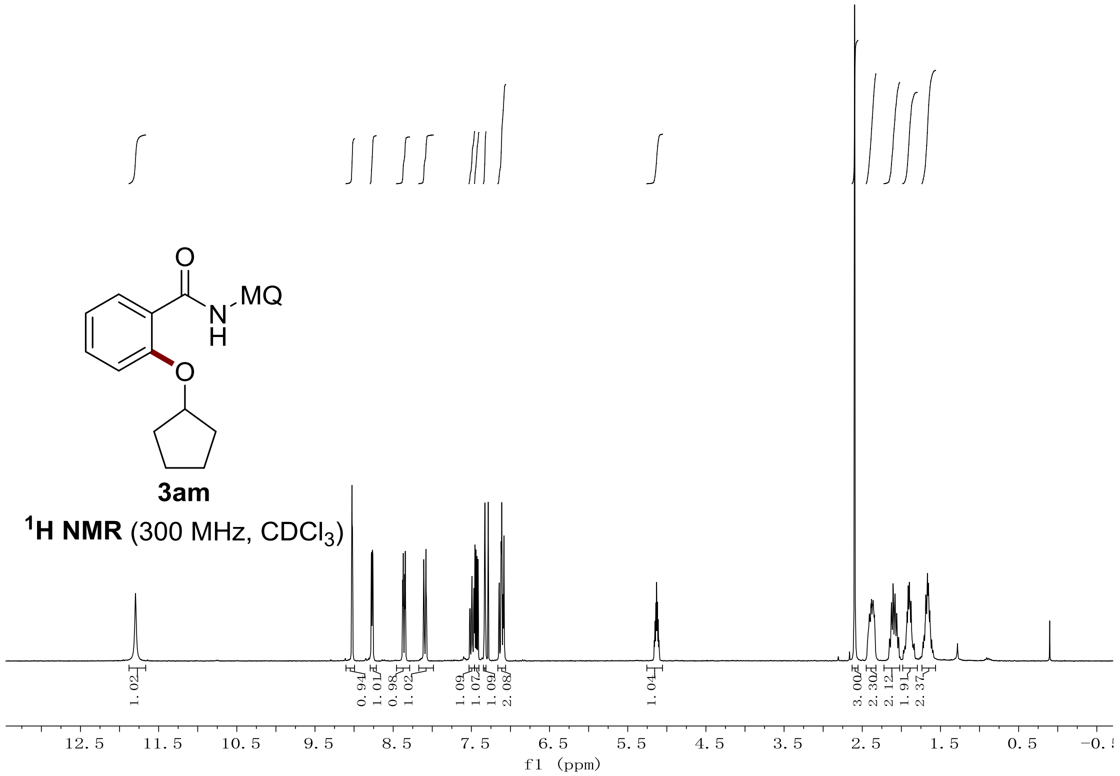
MQ
N
H
O
3am
^{1}H NMR (300 MHz, CDCl$_3$)
1.02
0.94
1.01
0.98
1.02
1.09
1.07
1.09
2.08
1.04
3.00
2.30
2.12
1.91
2.37
12.5 11.5 10.5 9.5 8.5 7.5 6.5 5.5 4.5 3.5 2.5 1.5 0.5
f1 (ppm)

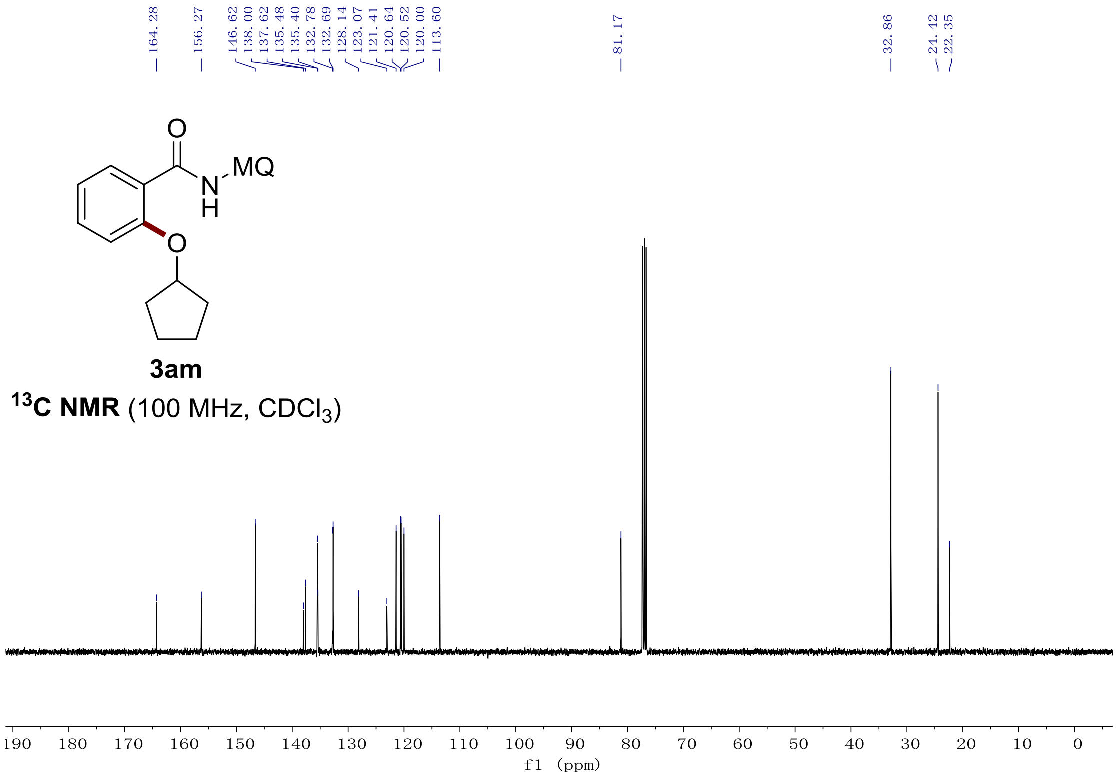
164.28
156.27
146.62
138.00
137.62
135.48
135.40
132.78
132.69
128.14
123.07
121.41
120.64
120.52
120.00
113.60
81.17
32.86
24.42
22.35
MQ
N
H
O
3am
^{13}C NMR (100 MHz, CDCl$_3$)
190 180 170 160 150 140 130 120 110 100 90 80 70 60 50 40 30 20 10 0
f1 (ppm)

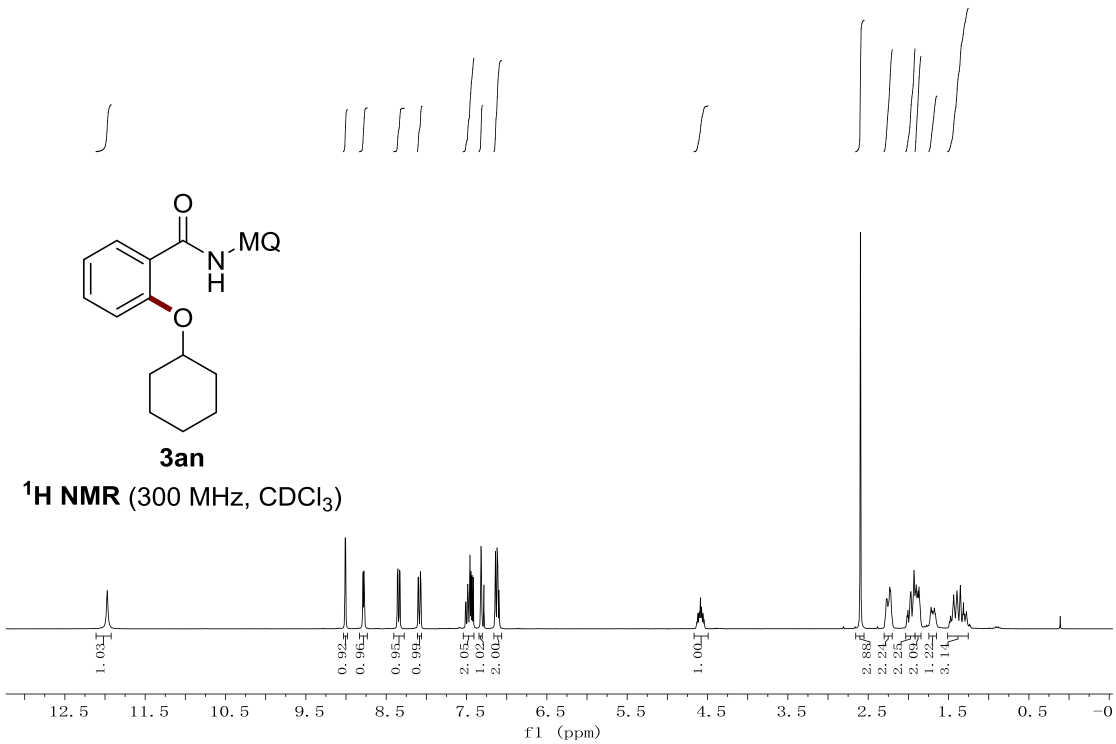

3an

^{1}H NMR (300 MHz, $CDCl_3$)

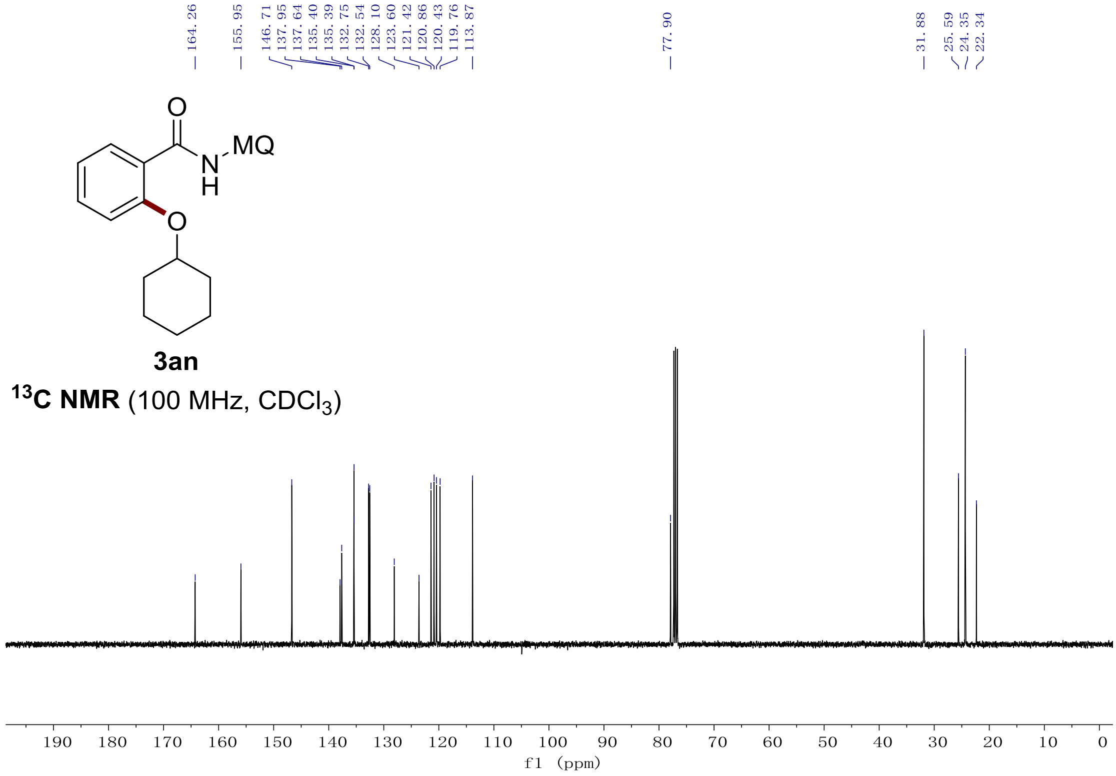

3an

^{13}C NMR (100 MHz, $CDCl_3$)

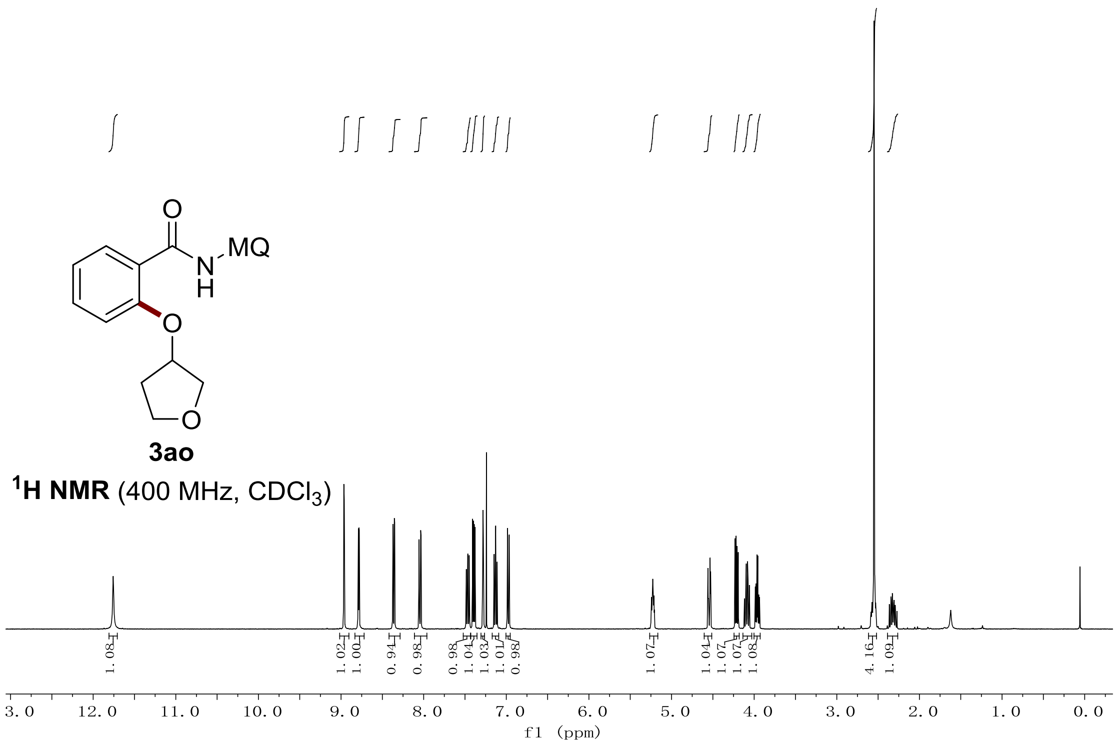
O
N
H
MQ
O
O
3ao
1H NMR (400 MHz, CDCl3)
1.08
1.02
1.00
0.94
0.98
0.98
1.04
1.03
1.01
0.98
1.07
1.04
1.07
1.07
1.08
4.16
1.09
f1 (ppm)

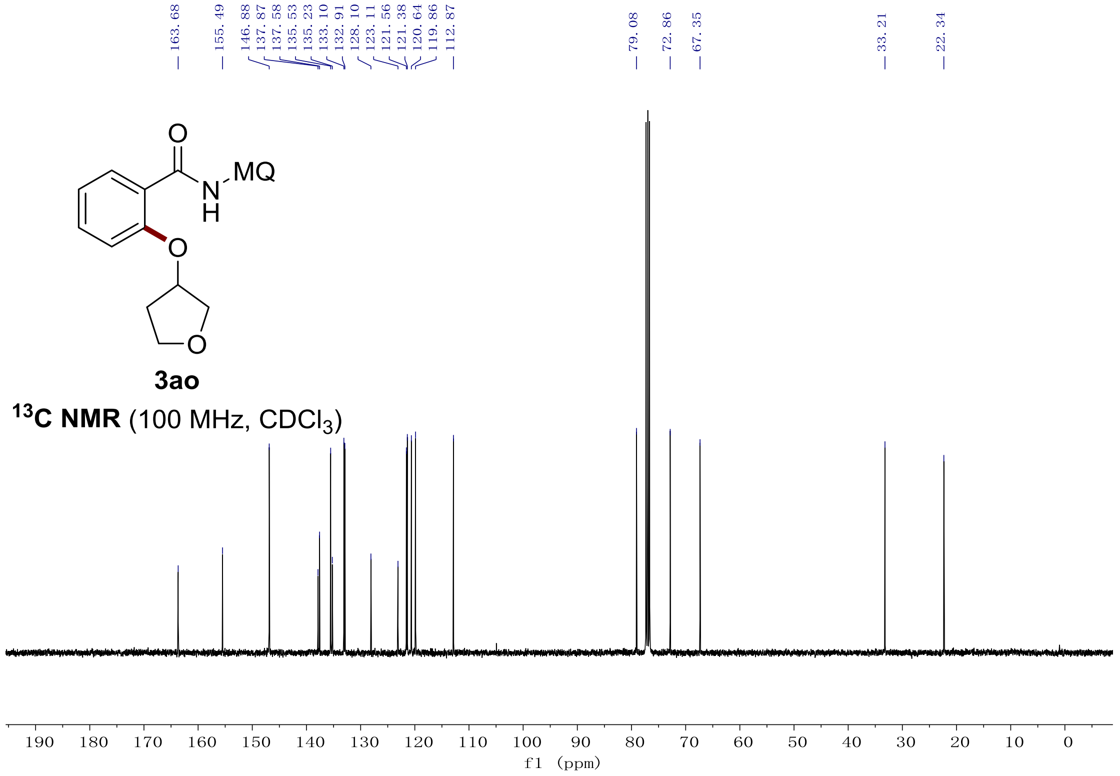
163.68
155.49
146.88
137.87
137.58
135.53
135.23
133.10
132.91
128.10
123.11
121.56
121.38
120.64
119.86
112.87
79.08
72.86
67.35
33.21
22.34
O
N
H
MQ
O
O
3ao
13C NMR (100 MHz, CDCl3)
f1 (ppm)

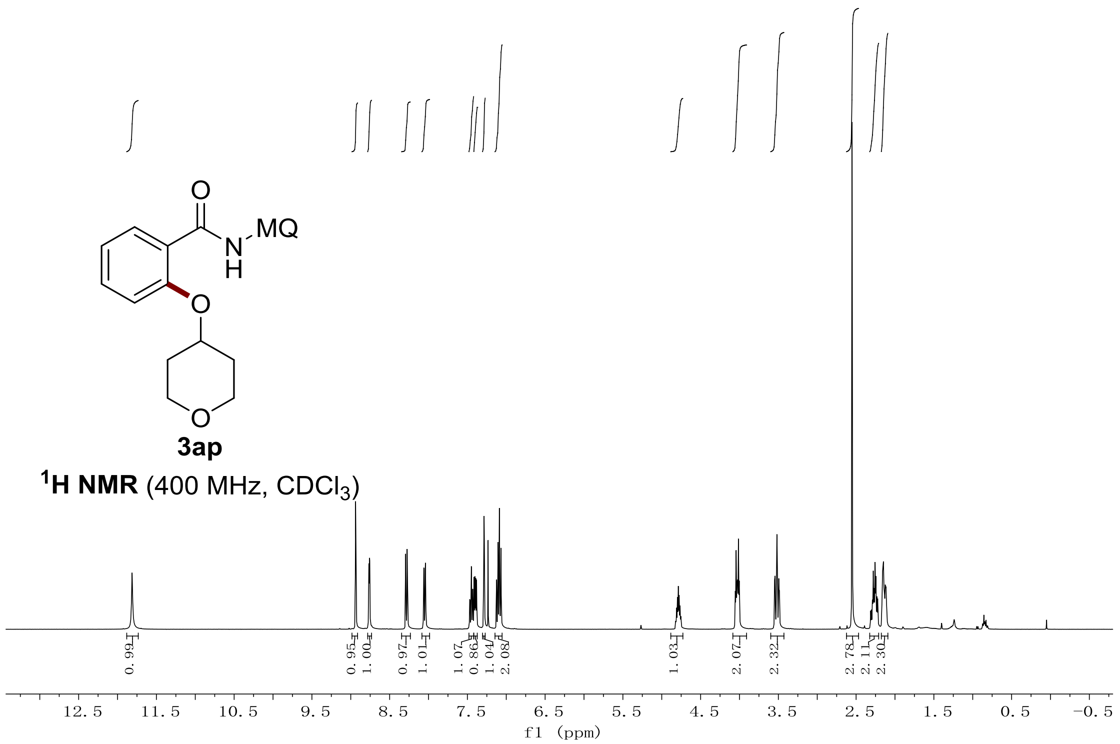

3ap
1H NMR (400 MHz, CDCl3)
0.99
0.95
1.00
0.97
1.01
1.07
0.86
1.04
2.08
1.03
2.07
2.32
2.78
2.11
2.30
f1 (ppm)

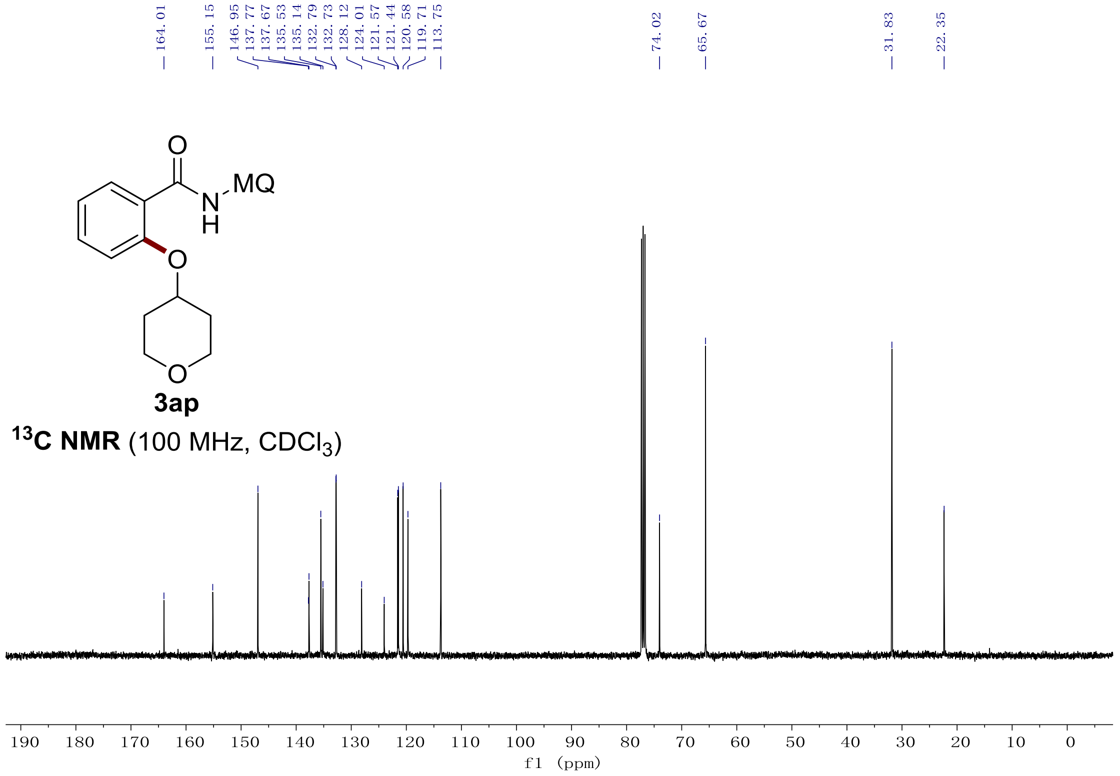

164.01
155.15
146.95
137.77
137.67
135.53
135.14
132.79
132.73
128.12
124.01
121.57
121.44
120.58
119.71
113.75
74.02
65.67
31.83
22.35
3ap
13C NMR (100 MHz, CDCl3)
f1 (ppm)

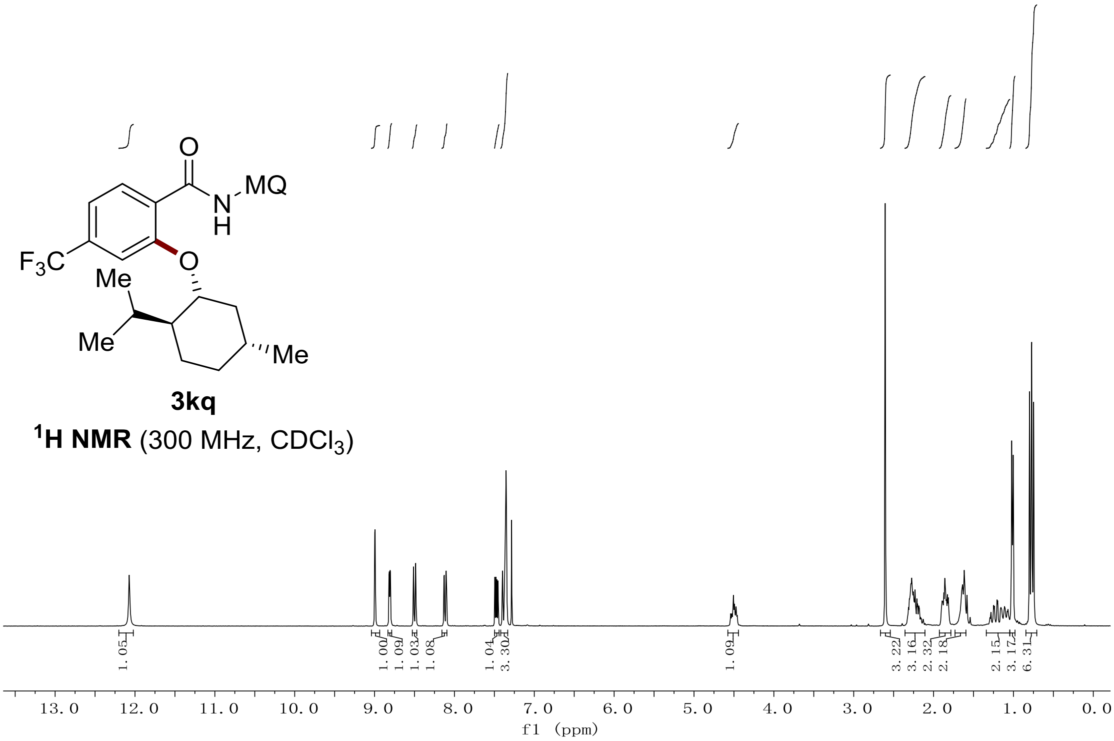
O
N
MQ
H
F3C
O
Me
Me
Me
3kq
1H NMR (300 MHz, CDCl3)
1.05
1.00
1.09
1.03
1.08
1.04
3.30
1.09
3.22
3.16
2.32
2.18
2.15
3.17
6.31
13.0 12.0 11.0 10.0 9.0 8.0 7.0 6.0 5.0 4.0 3.0 2.0 1.0 0.0
f1 (ppm)

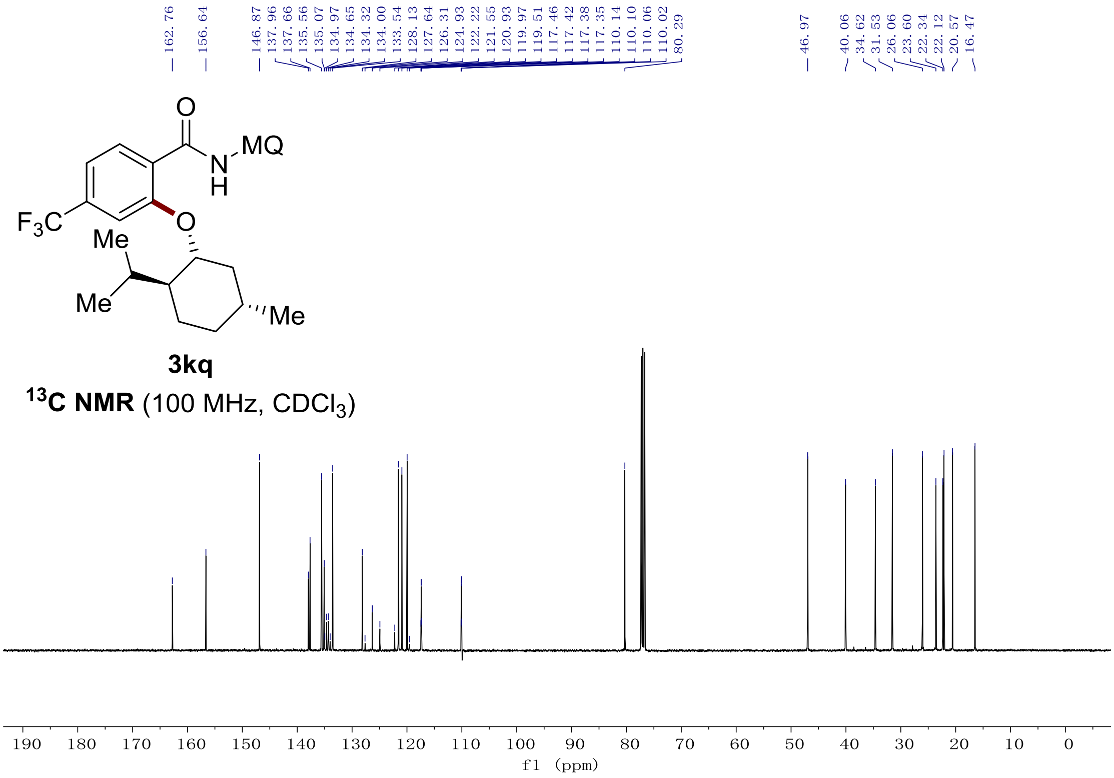
162.76
156.64
146.87
137.96
137.66
135.56
135.07
134.97
134.65
134.32
134.00
133.54
128.13
127.64
126.31
124.93
122.22
121.55
120.93
119.97
119.51
117.46
117.42
117.38
117.35
110.14
110.10
110.06
110.02
80.29
46.97
40.06
34.62
31.53
26.06
23.60
22.34
22.12
20.57
16.47
O
N
MQ
H
F3C
O
Me
Me
Me
3kq
13C NMR (100 MHz, CDCl3)
190 180 170 160 150 140 130 120 110 100 90 80 70 60 50 40 30 20 10 0
f1 (ppm)

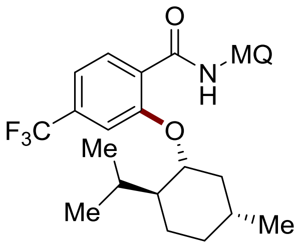

3kq

^{19}F NMR (376 MHz, $CDCl_3$)

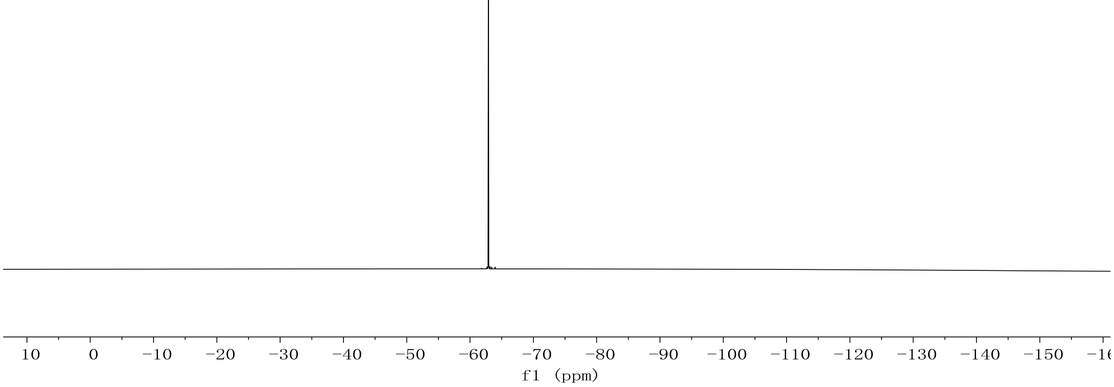

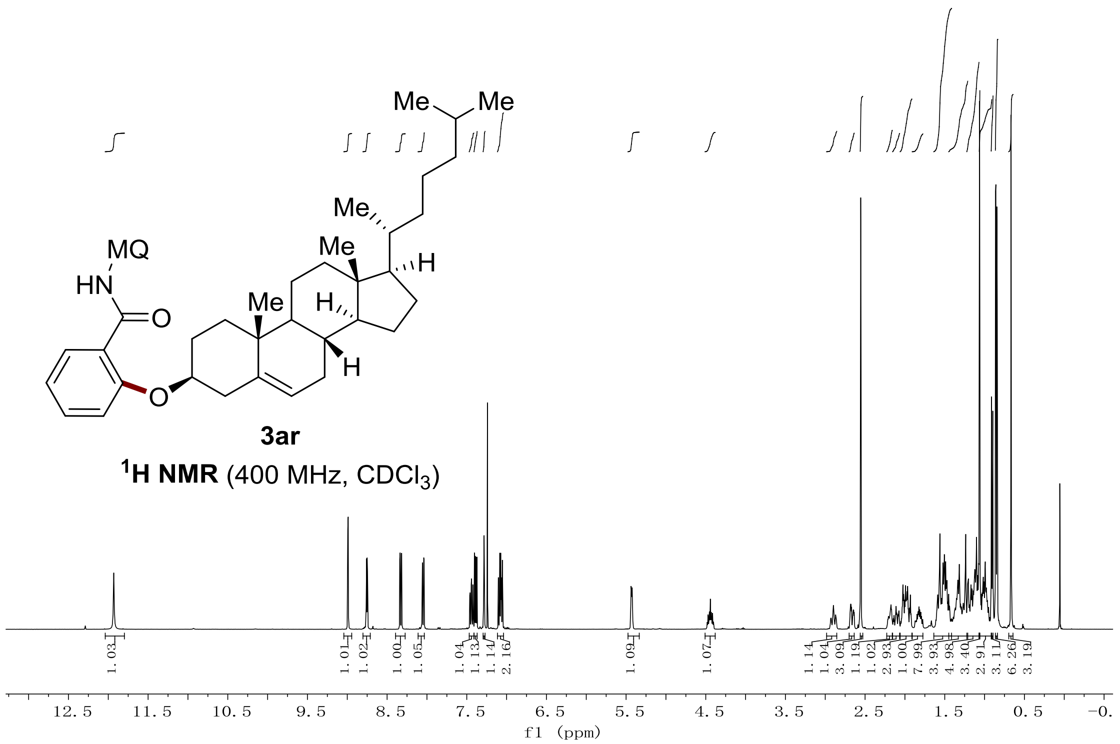

3ar

^{1}H NMR (400 MHz, $CDCl_3$)

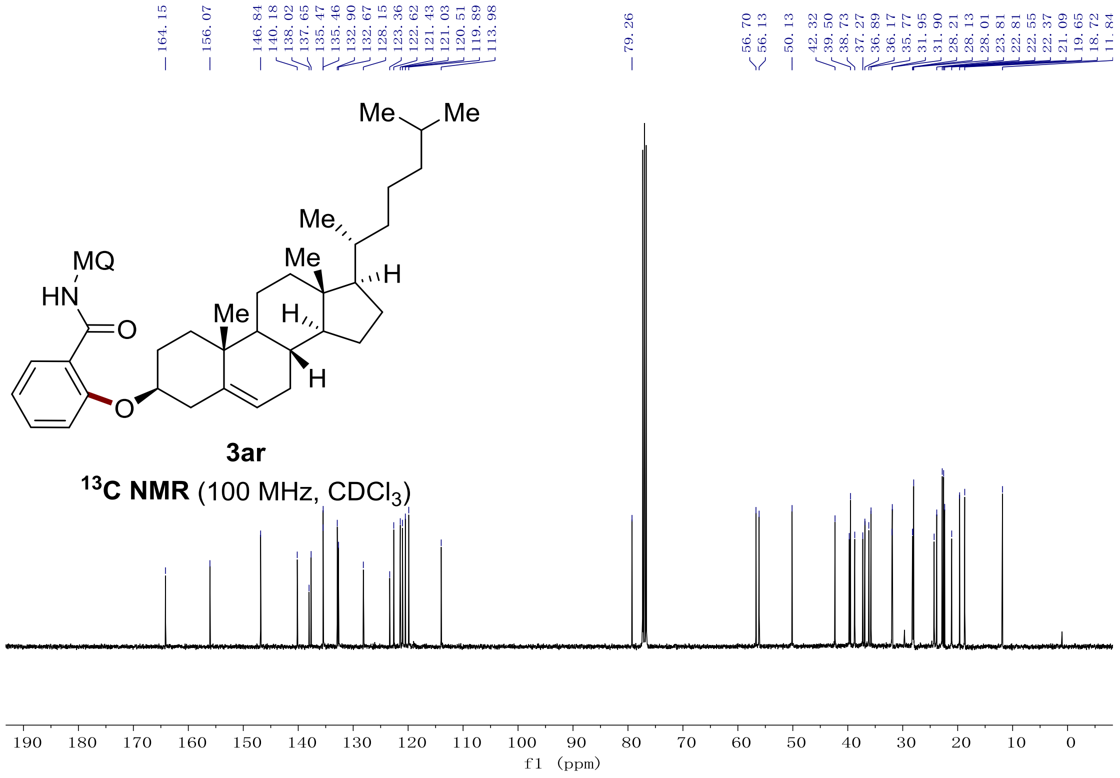

3ar

^{13}C NMR (100 MHz, $CDCl_3$)

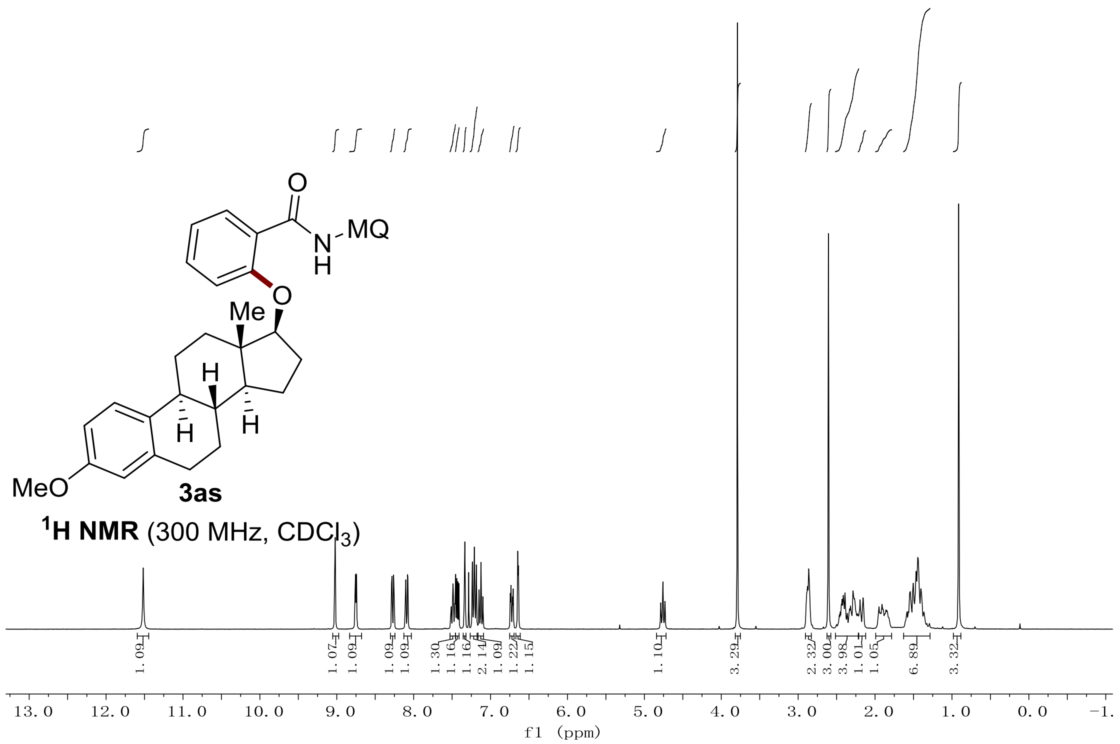

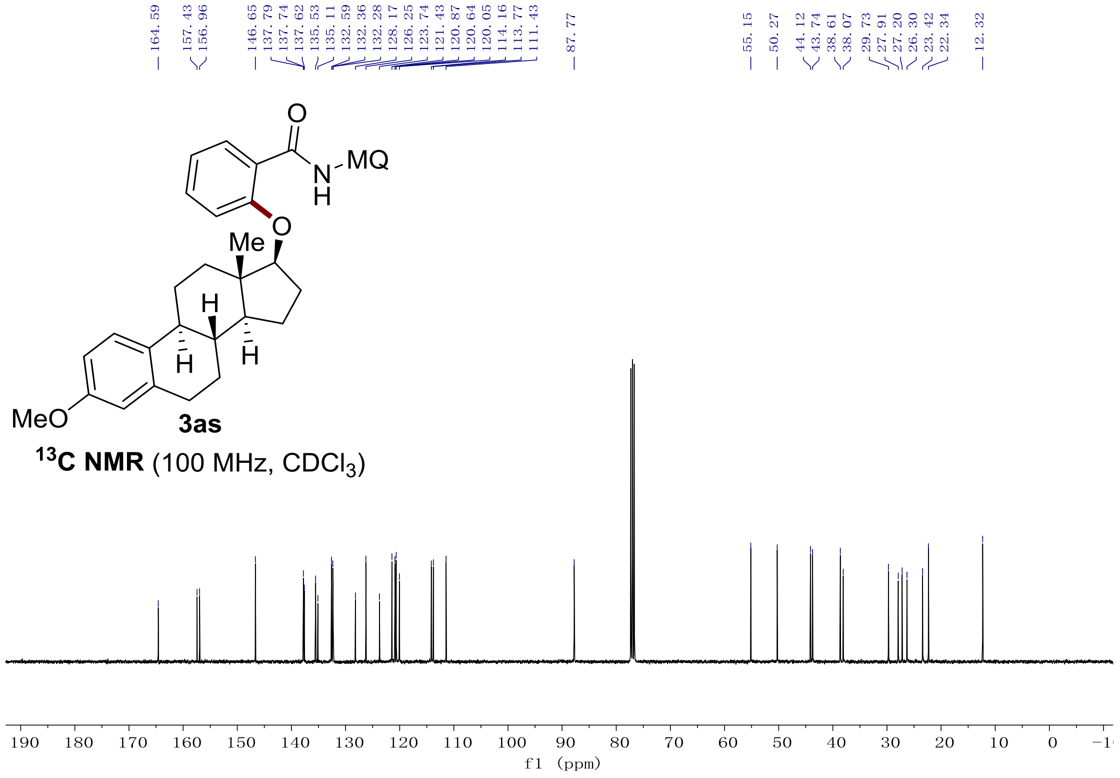

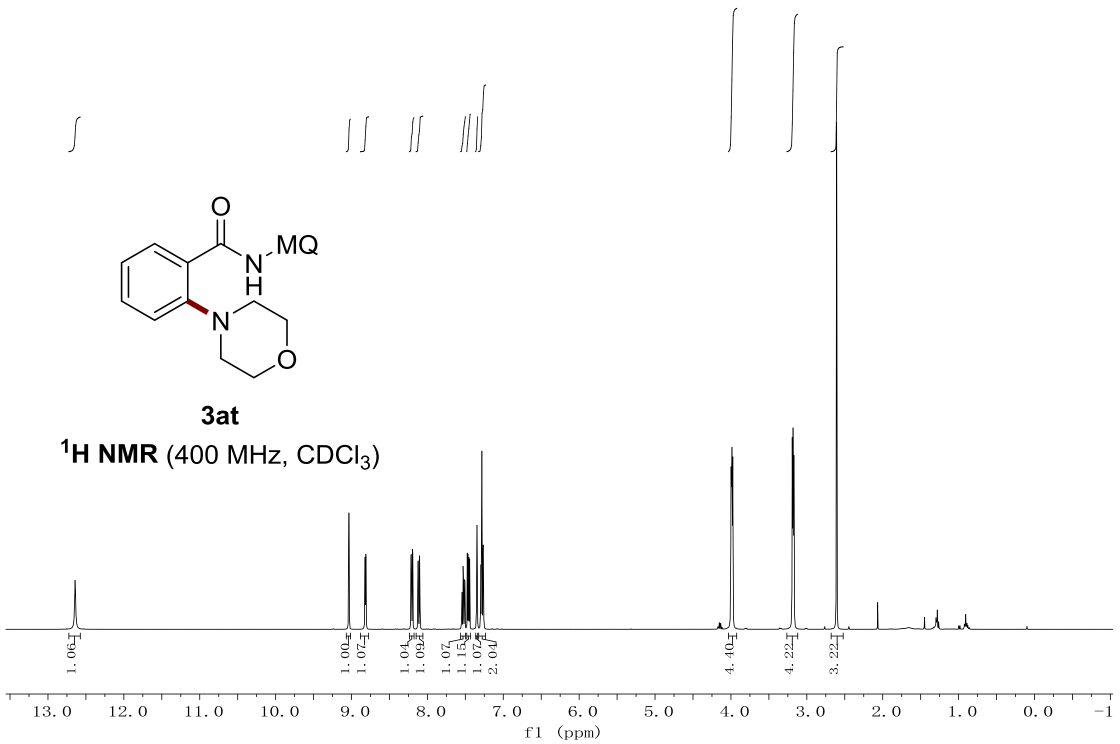
3at
1H NMR (400 MHz, CDCl3)
f1 (ppm)

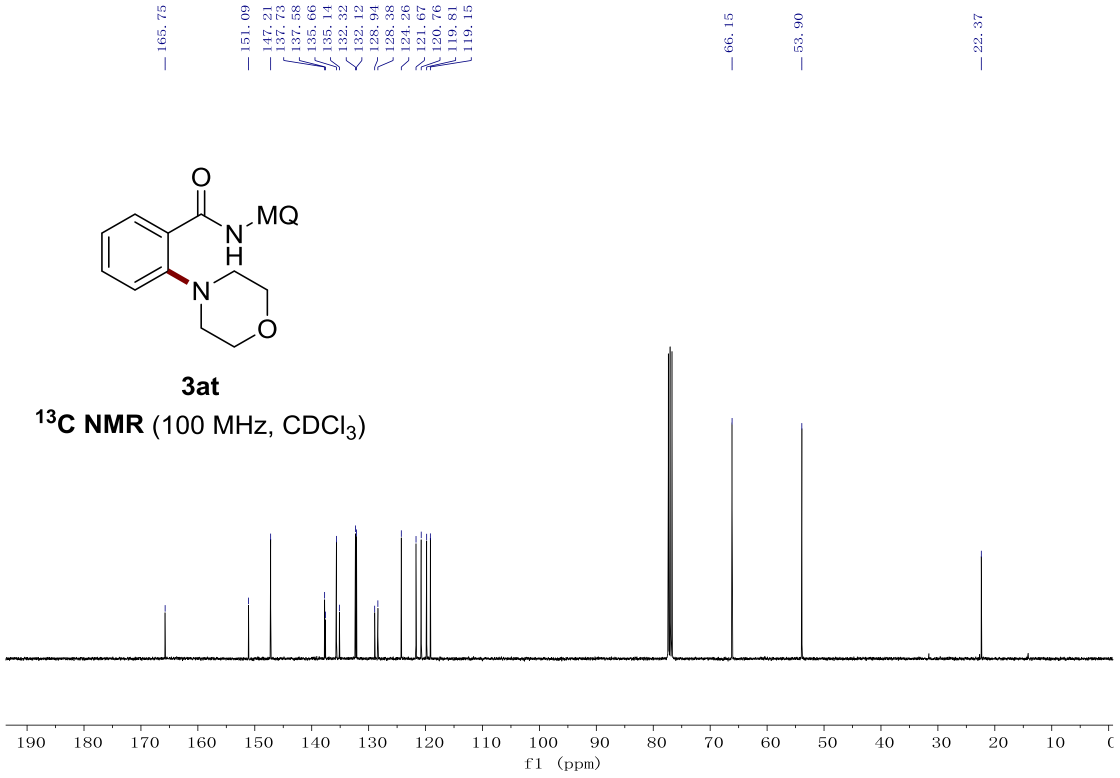
165.75
151.09
147.21
137.73
137.58
135.66
135.14
132.32
132.12
128.94
128.38
124.26
121.67
120.76
119.81
119.15
66.15
53.90
22.37
3at
13C NMR (100 MHz, CDCl3)
f1 (ppm)

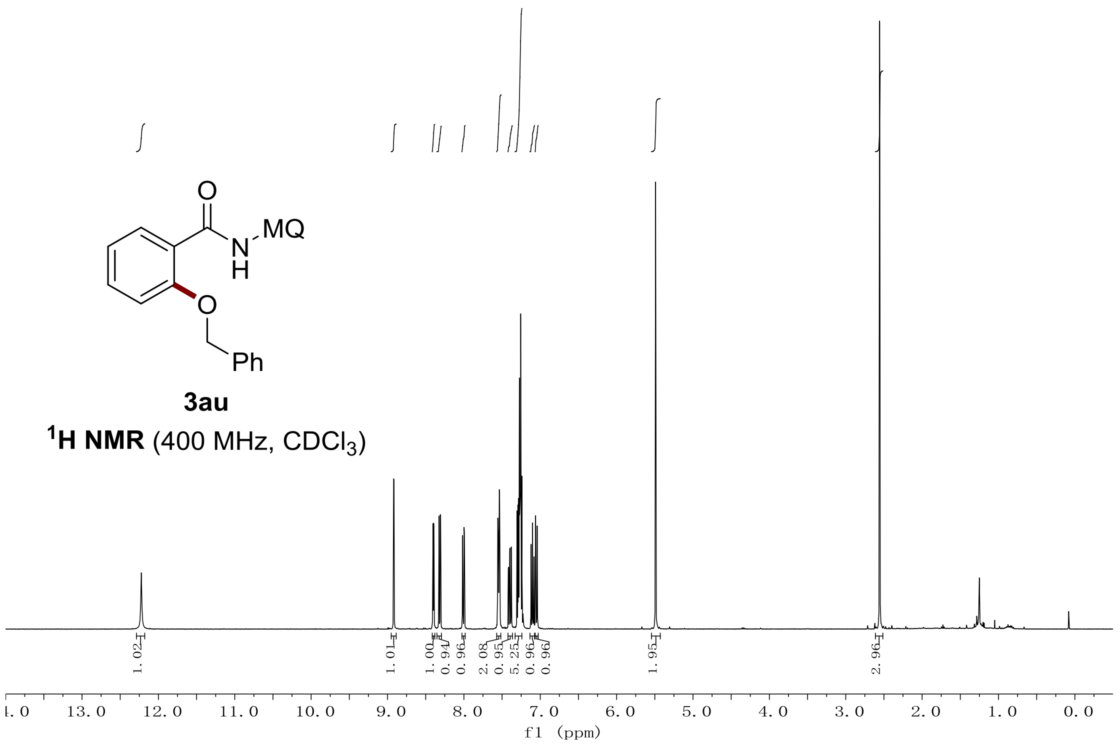

O
N
MQ
H
O
Ph
3au
¹H NMR (400 MHz, CDCl3)
1.02
1.01
1.00
0.94
0.96
2.08
0.95
5.25
0.96
0.96
1.95
2.96
f1 (ppm)

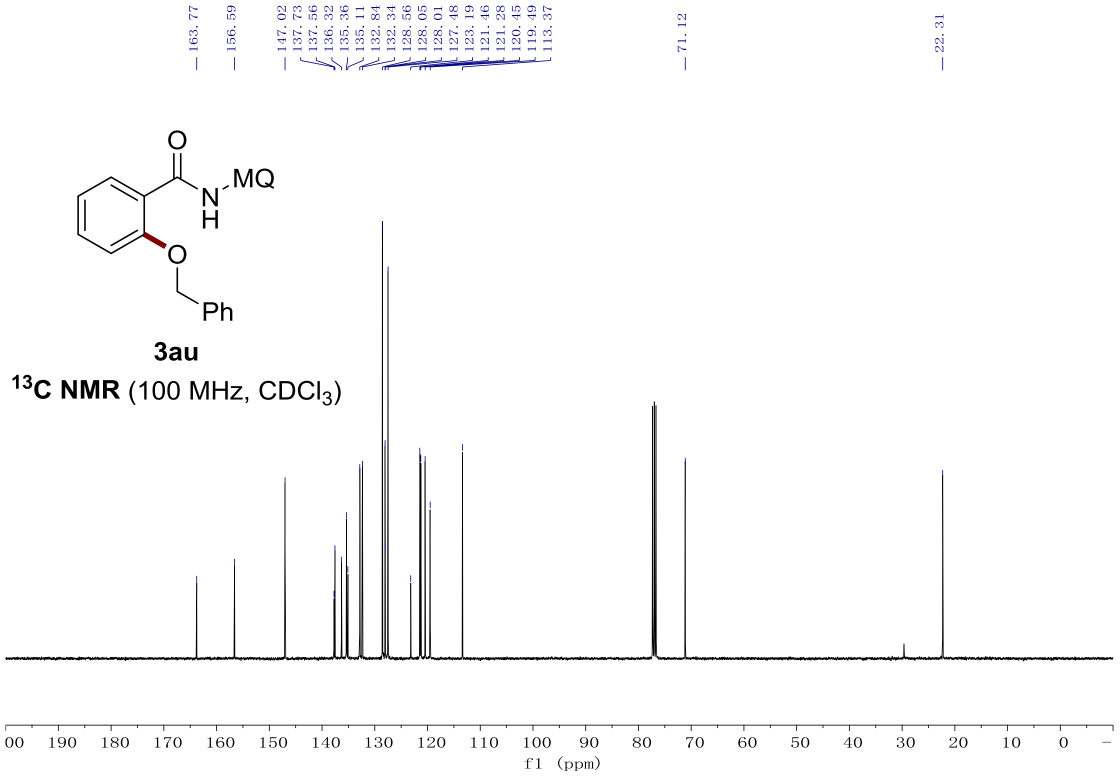

163.77
156.59
147.02
137.73
137.56
136.32
135.36
135.11
132.84
132.34
128.56
128.05
128.01
127.48
123.19
121.46
121.28
120.45
119.49
113.37
71.12
22.31
O
N
MQ
H
O
Ph
3au
¹³C NMR (100 MHz, CDCl3)
f1 (ppm)

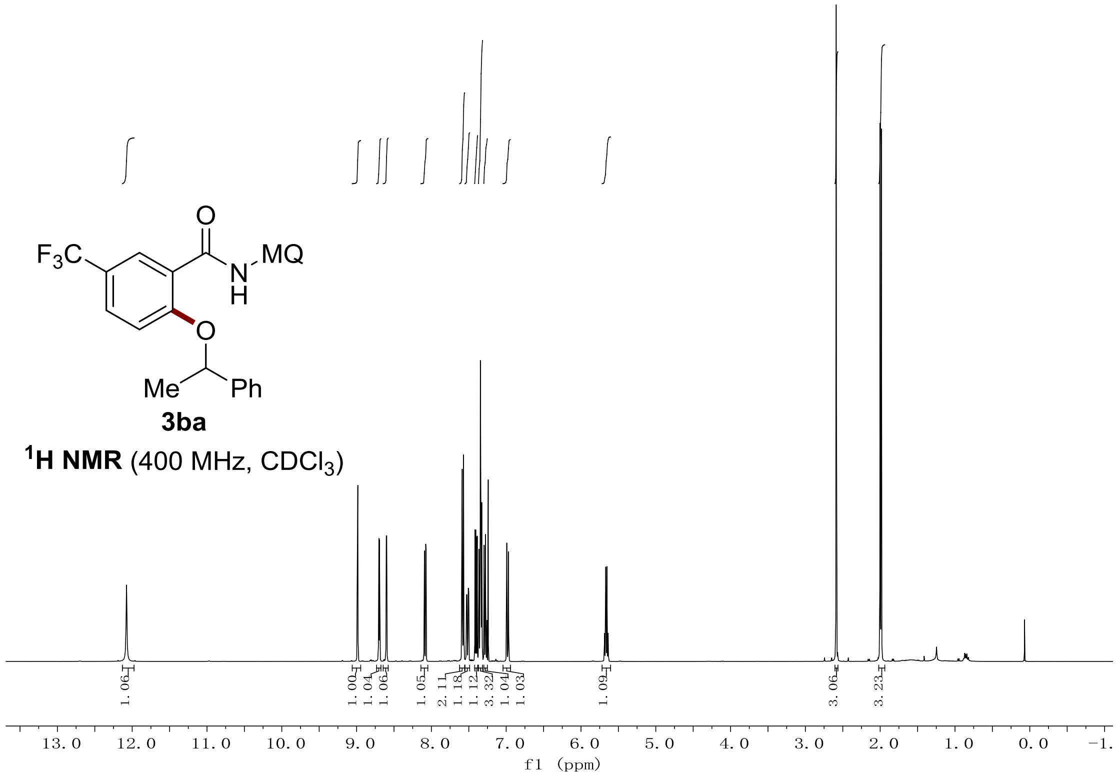
F3C
O
N
H
MQ
O
Me
Ph
3ba
1H NMR (400 MHz, CDCl3)
1.06
1.00
1.04
1.06
1.05
2.11
1.18
1.12
3.32
1.04
1.03
1.09
3.06
3.23
f1 (ppm)

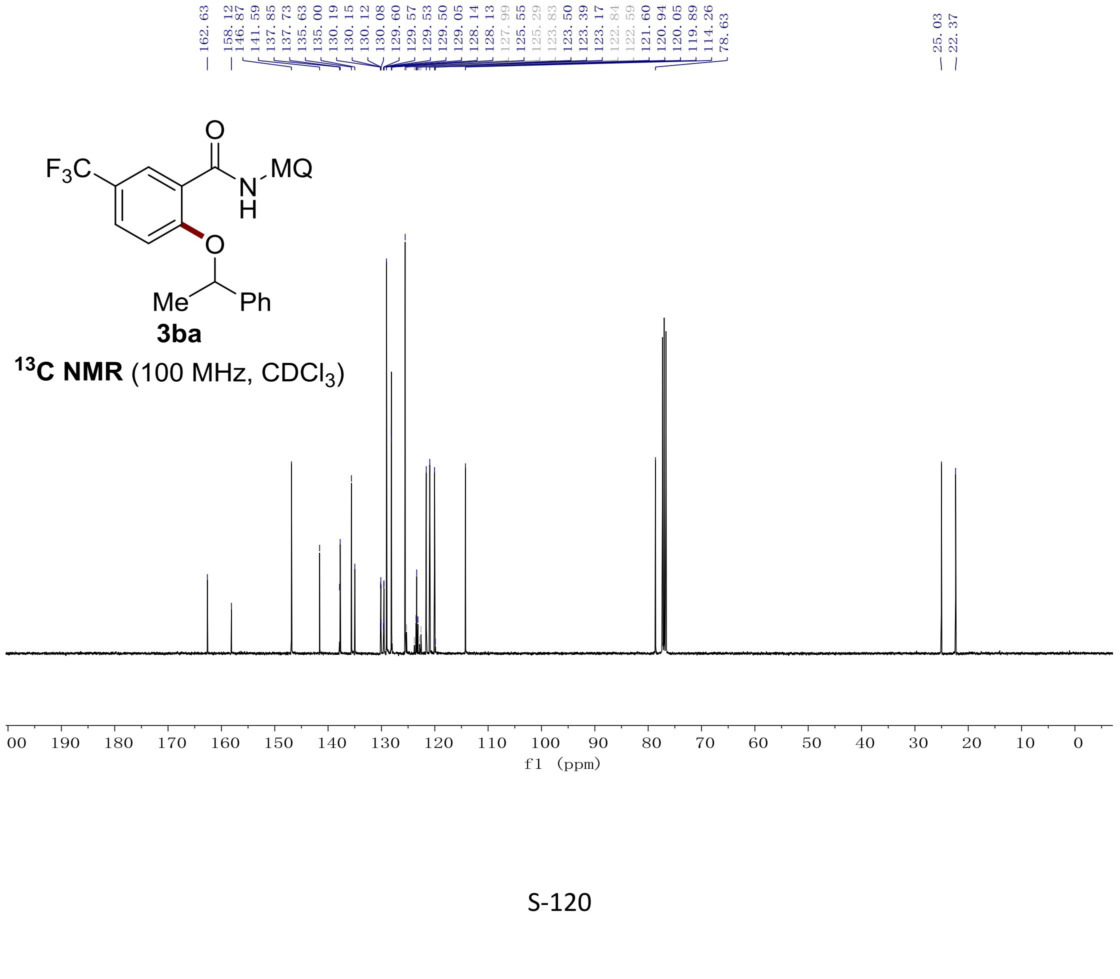
162.63
158.12
146.87
141.59
137.85
137.73
135.63
135.00
130.19
130.15
130.12
130.08
129.60
129.57
129.53
129.50
129.05
128.14
128.13
127.99
125.55
125.29
123.83
123.50
123.39
123.17
122.84
122.59
121.60
120.94
120.05
119.89
114.26
78.63
25.03
22.37
F3C
O
N
H
MQ
O
Me
Ph
3ba
13C NMR (100 MHz, CDCl3)
f1 (ppm)

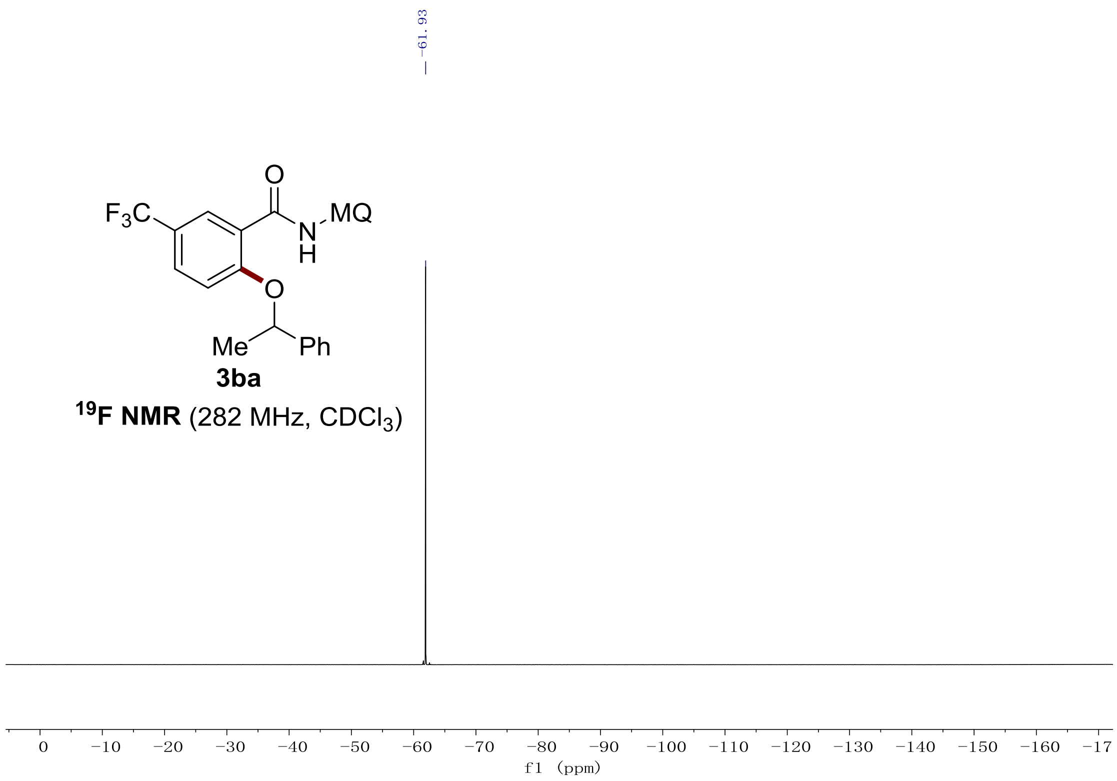

−61.93
F3C
O
N
H
MQ
O
Me
Ph
3ba
19F NMR (282 MHz, CDCl3)
0 −10 −20 −30 −40 −50 −60 −70 −80 −90 −100 −110 −120 −130 −140 −150 −160 −17
f1 (ppm)

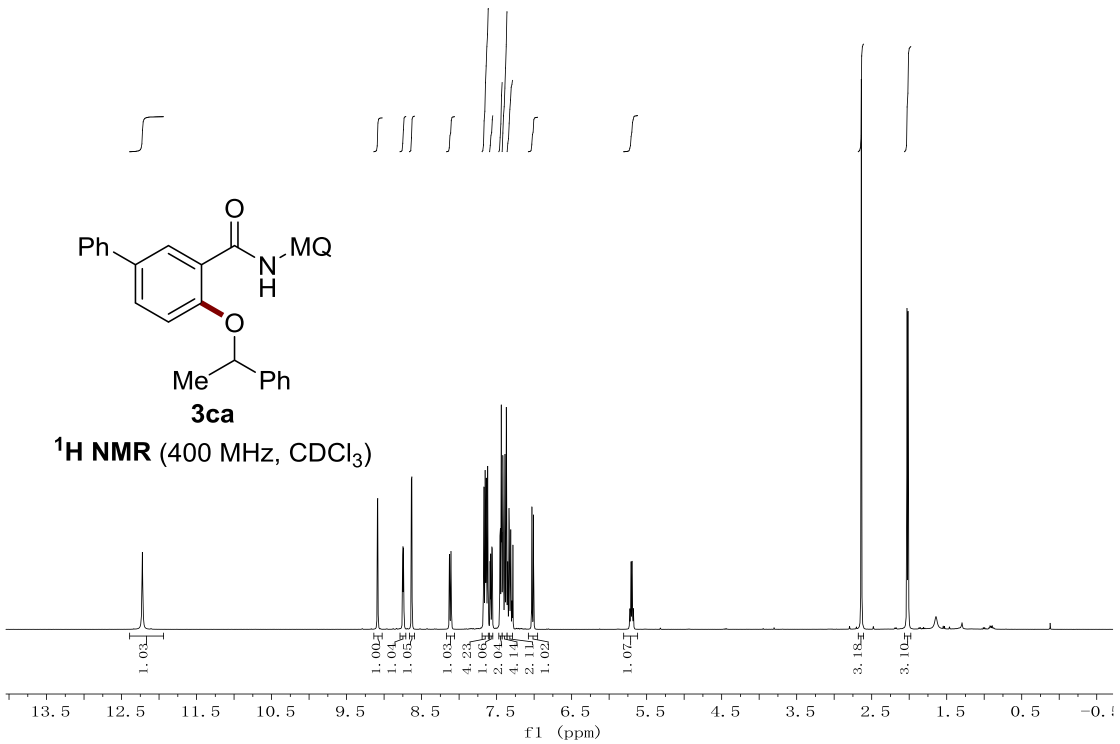

3ca

^{1}H NMR (400 MHz, $CDCl_3$)

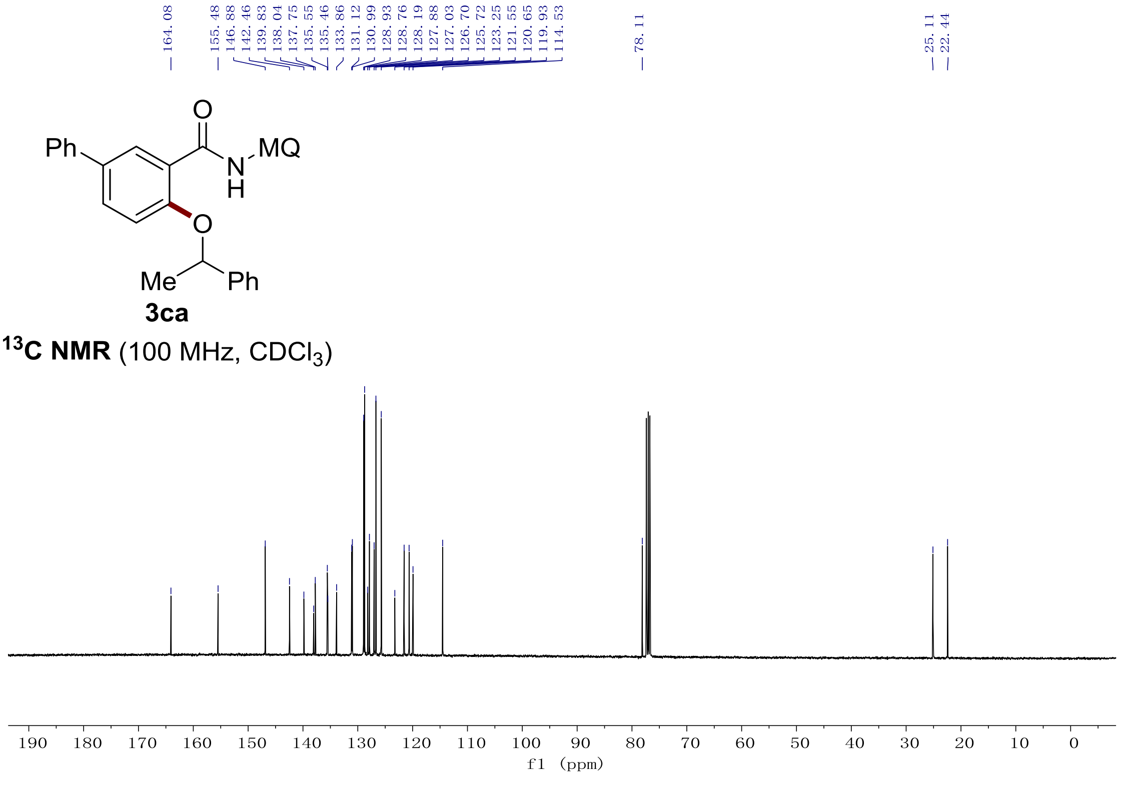

3ca

^{13}C NMR (100 MHz, $CDCl_3$)

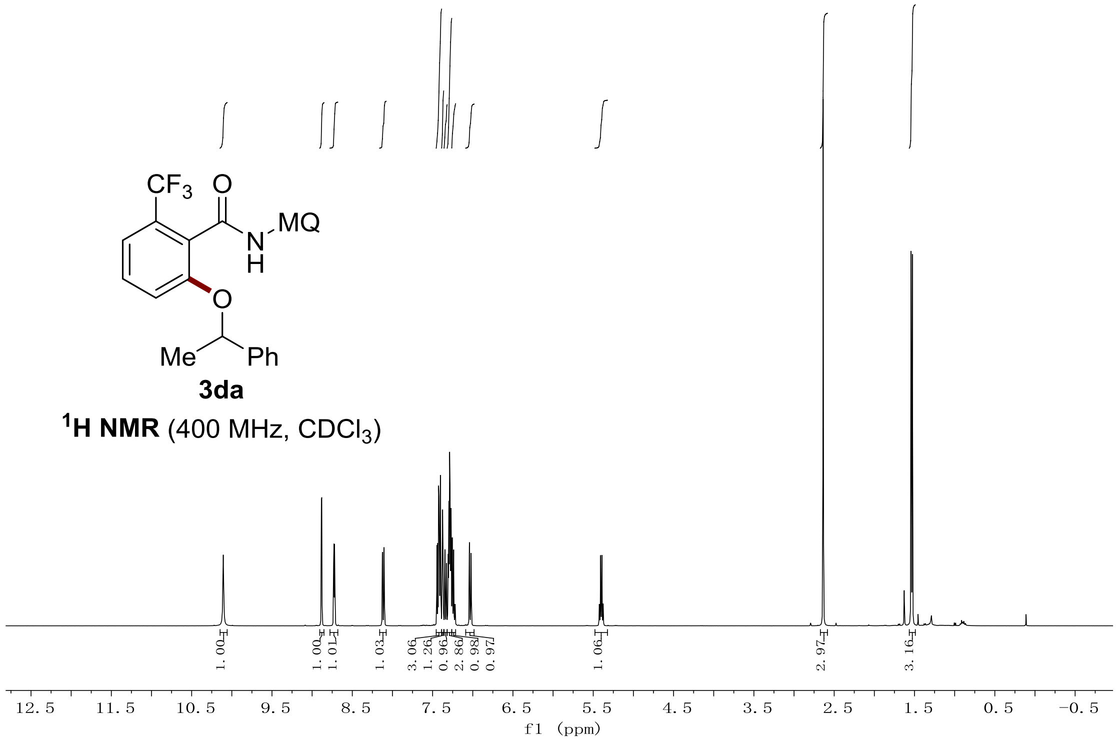

3da

^{1}H NMR (400 MHz, $CDCl_3$)

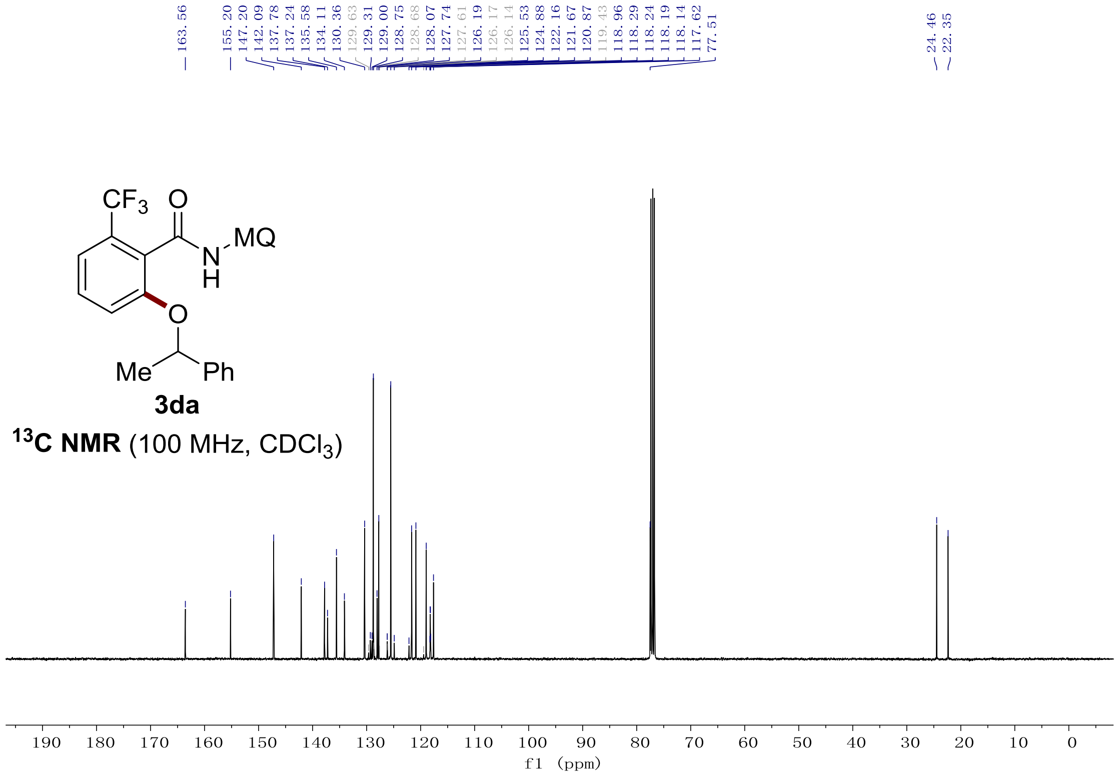

3da

^{13}C NMR (100 MHz, $CDCl_3$)

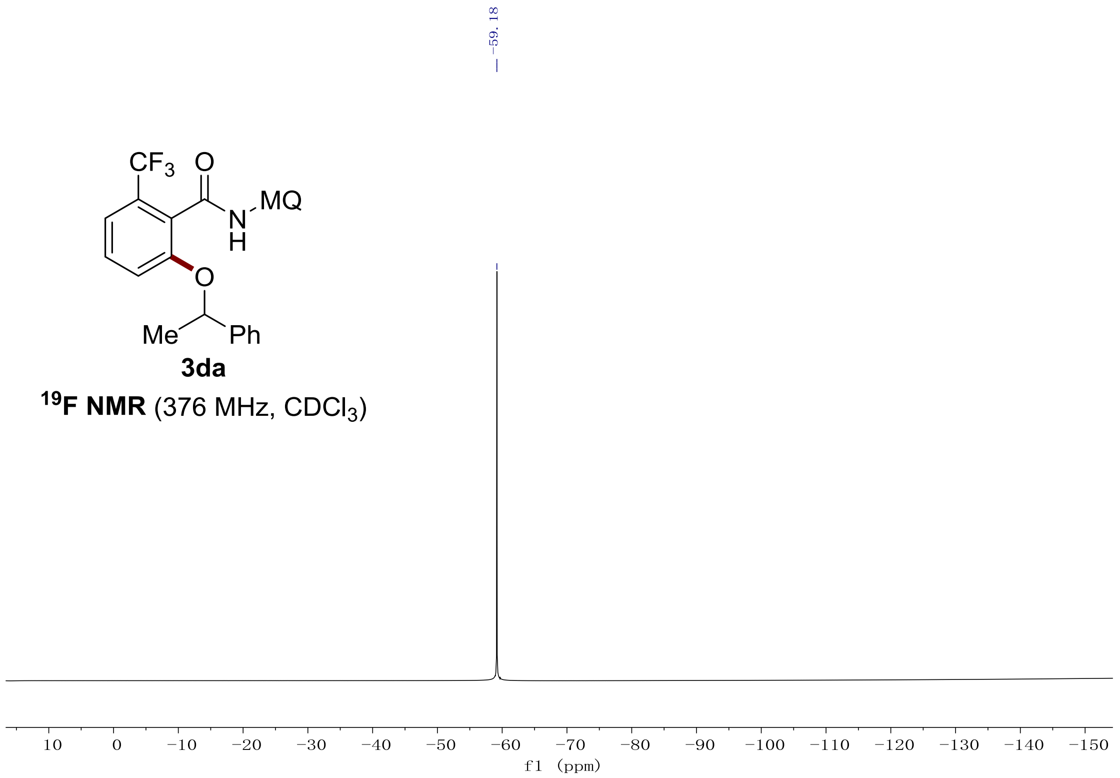

−59.18
CF3
O
N
H
MQ
O
Me
Ph
3da
19F NMR (376 MHz, CDCl3)
10 0 −10 −20 −30 −40 −50 −60 −70 −80 −90 −100 −110 −120 −130 −140 −150
f1 (ppm)

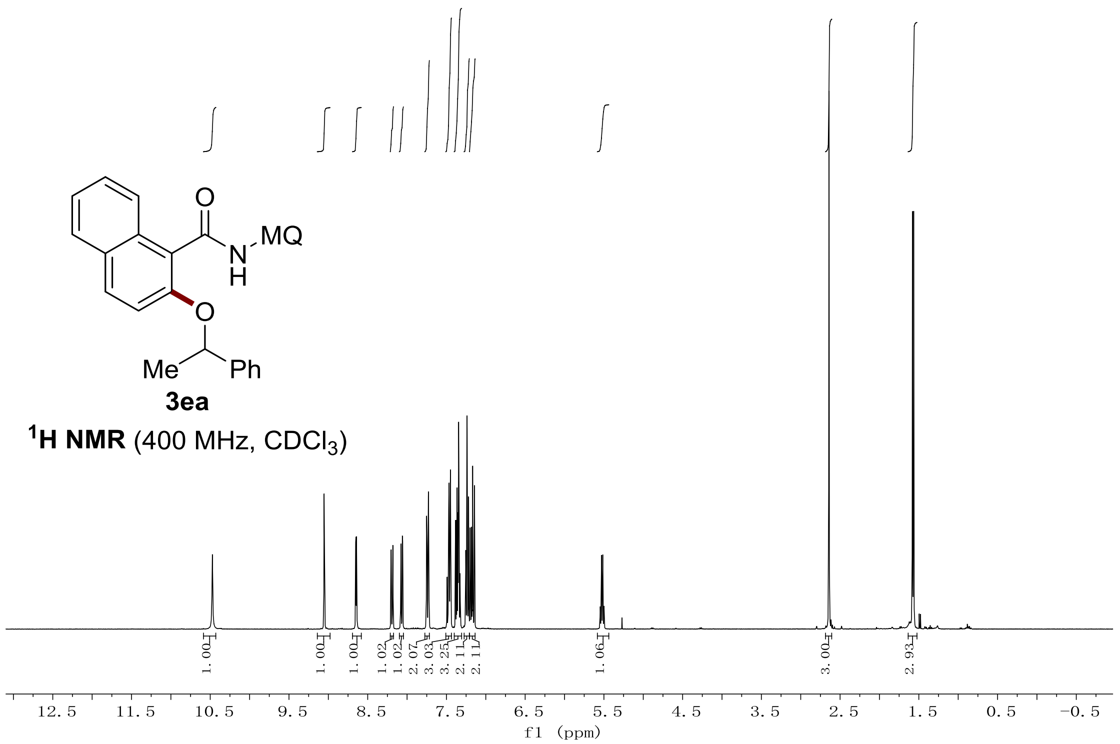
O
N
H
MQ
O
Me
Ph
3ea
^{1}H NMR (400 MHz, CDCl$_3$)
1.00
1.00
1.00
1.02
1.02
2.07
3.03
3.25
2.11
2.11
1.06
3.00
2.93
12.5 11.5 10.5 9.5 8.5 7.5 6.5 5.5 4.5 3.5 2.5 1.5 0.5 −0.5
f1 (ppm)

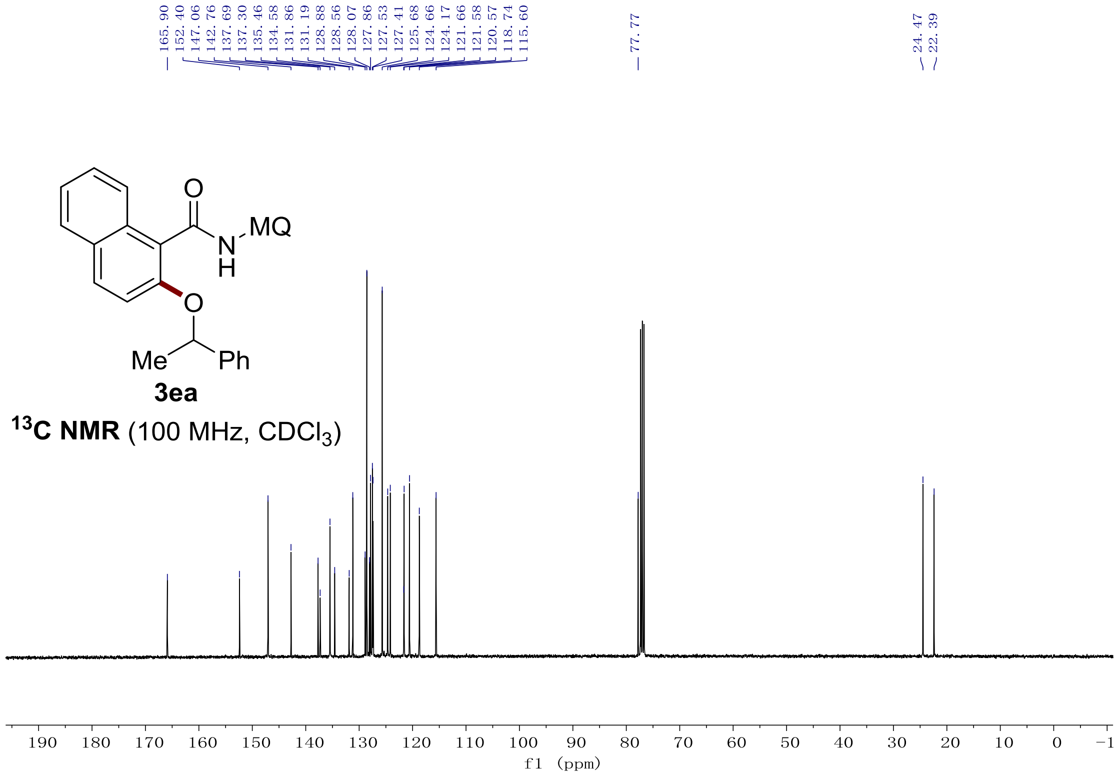
165.90
152.40
147.06
142.76
137.69
137.30
135.46
134.58
131.86
131.19
128.88
128.56
128.07
127.86
127.53
127.41
125.68
124.66
124.17
121.66
121.58
120.57
118.74
115.60
77.77
24.47
22.39
O
N
H
MQ
O
Me
Ph
3ea
^{13}C NMR (100 MHz, CDCl$_3$)
190 180 170 160 150 140 130 120 110 100 90 80 70 60 50 40 30 20 10 0 −1
f1 (ppm)

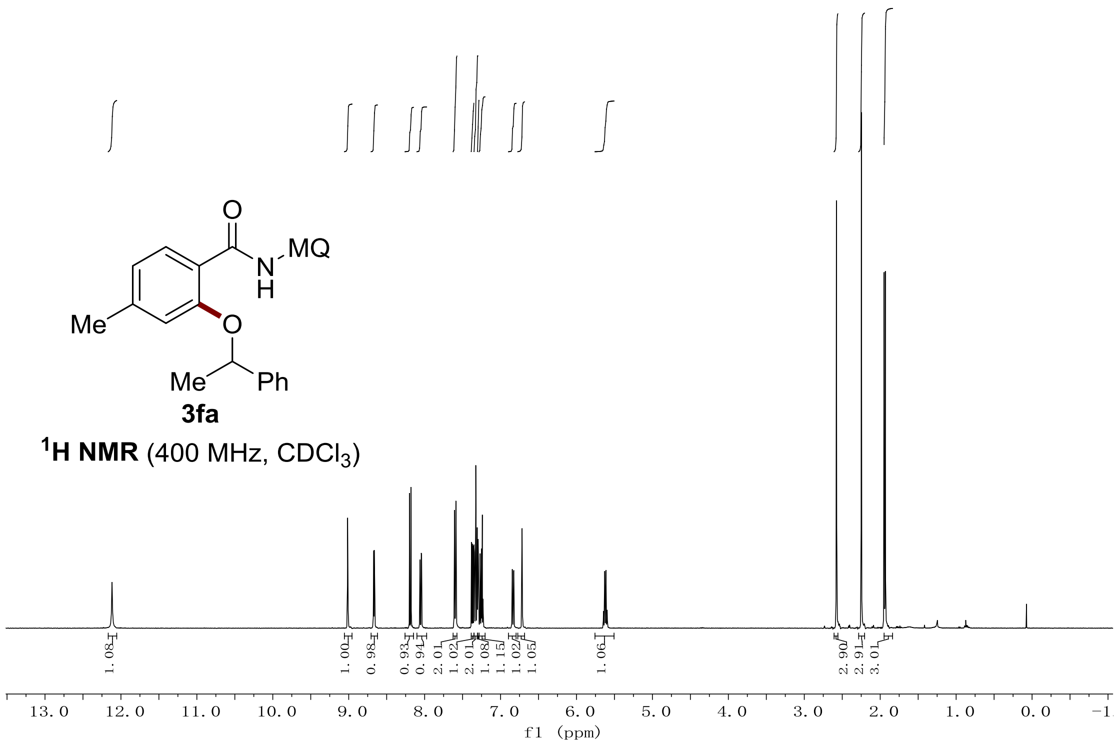

3fa

^{1}H NMR (400 MHz, $CDCl_3$)

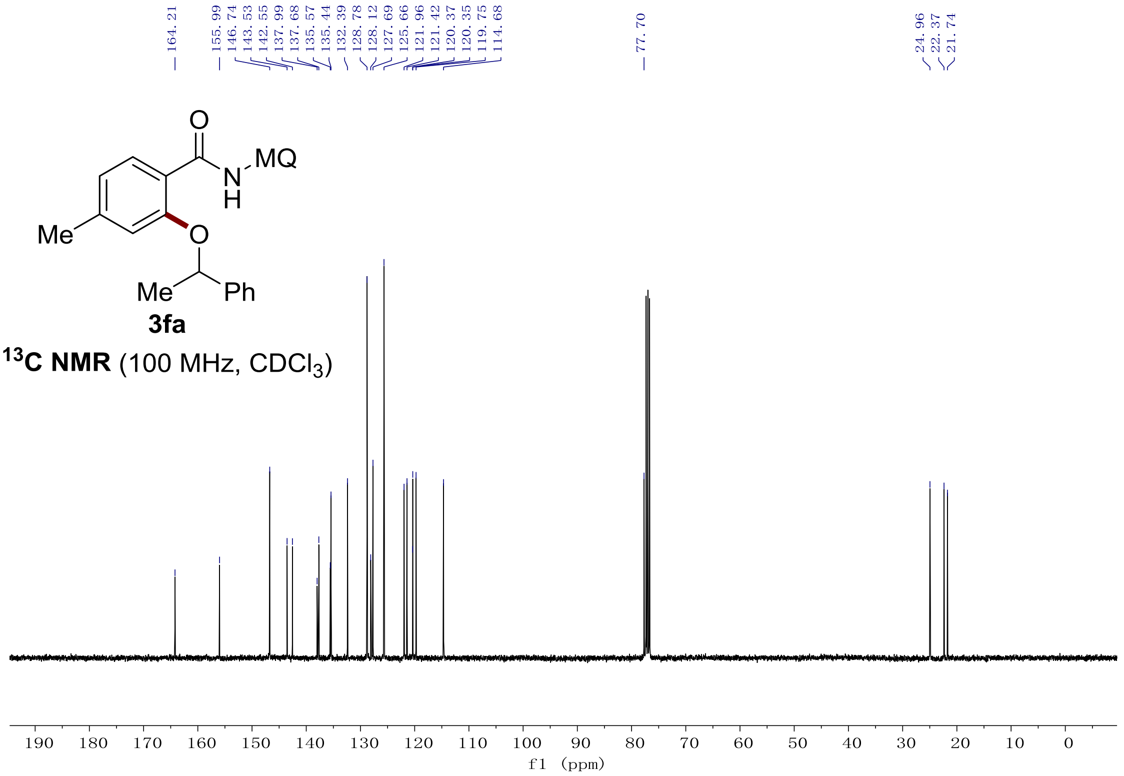

3fa

^{13}C NMR (100 MHz, $CDCl_3$)

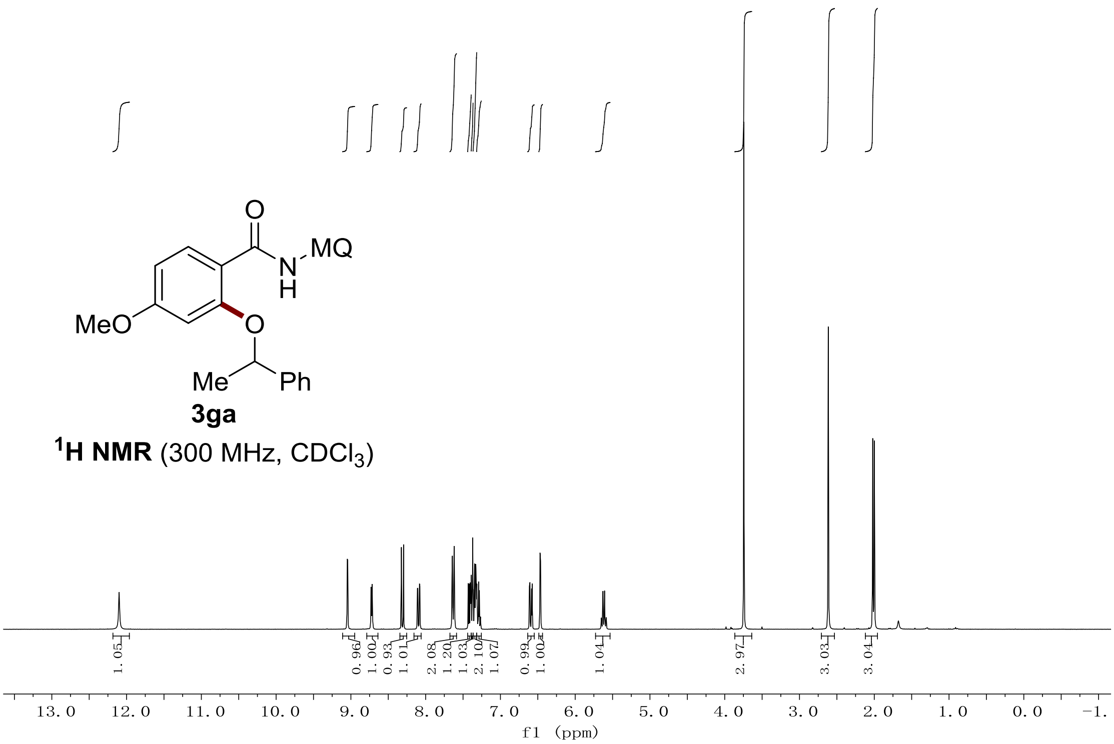

3ga

^{1}H NMR (300 MHz, $CDCl_3$)

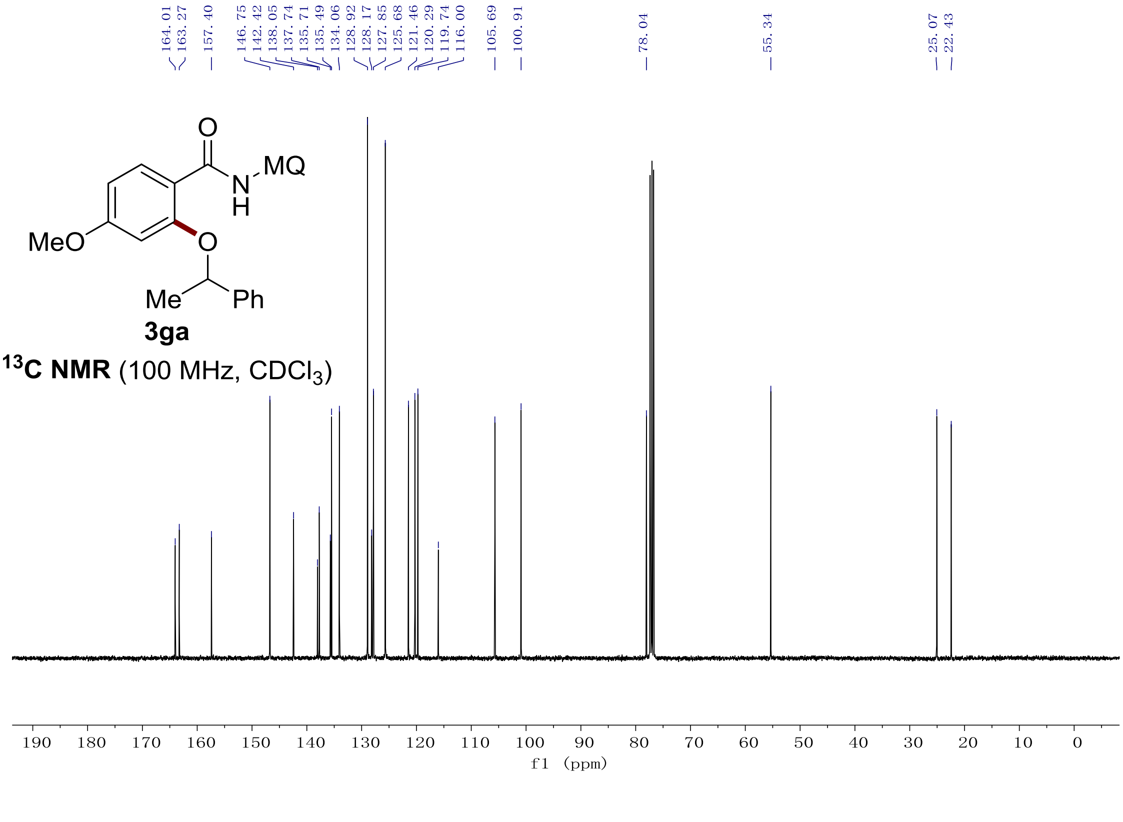

3ga

^{13}C NMR (100 MHz, $CDCl_3$)

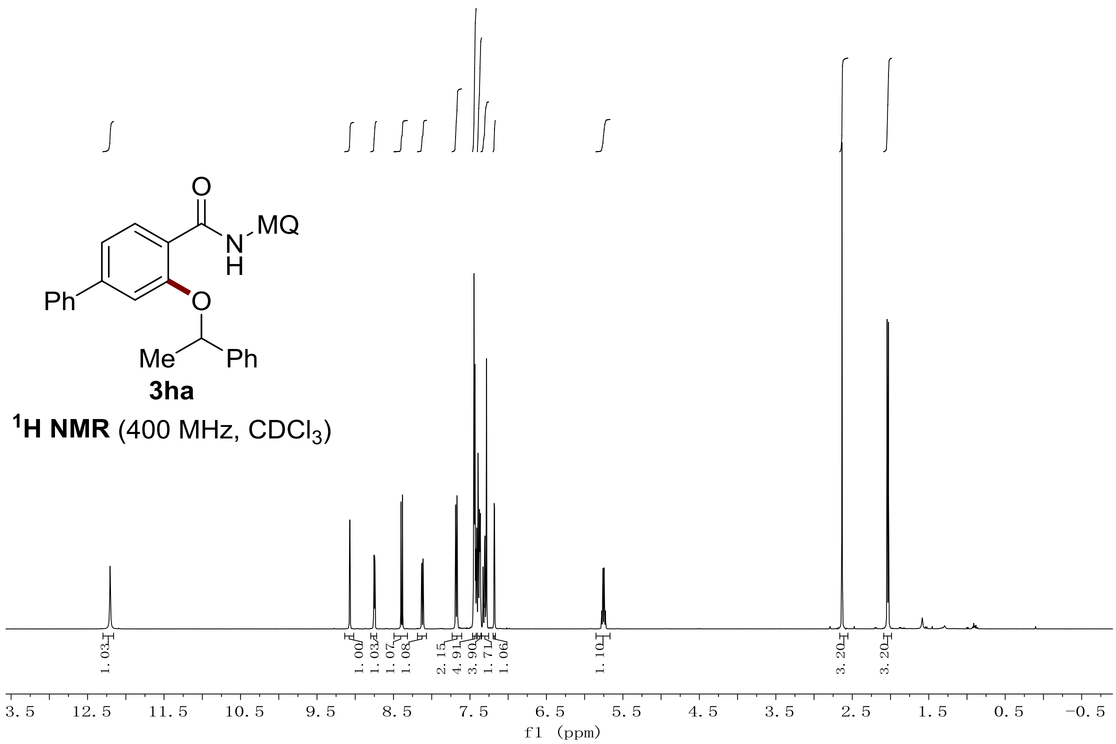

3ha

^{1}H NMR (400 MHz, $CDCl_3$)

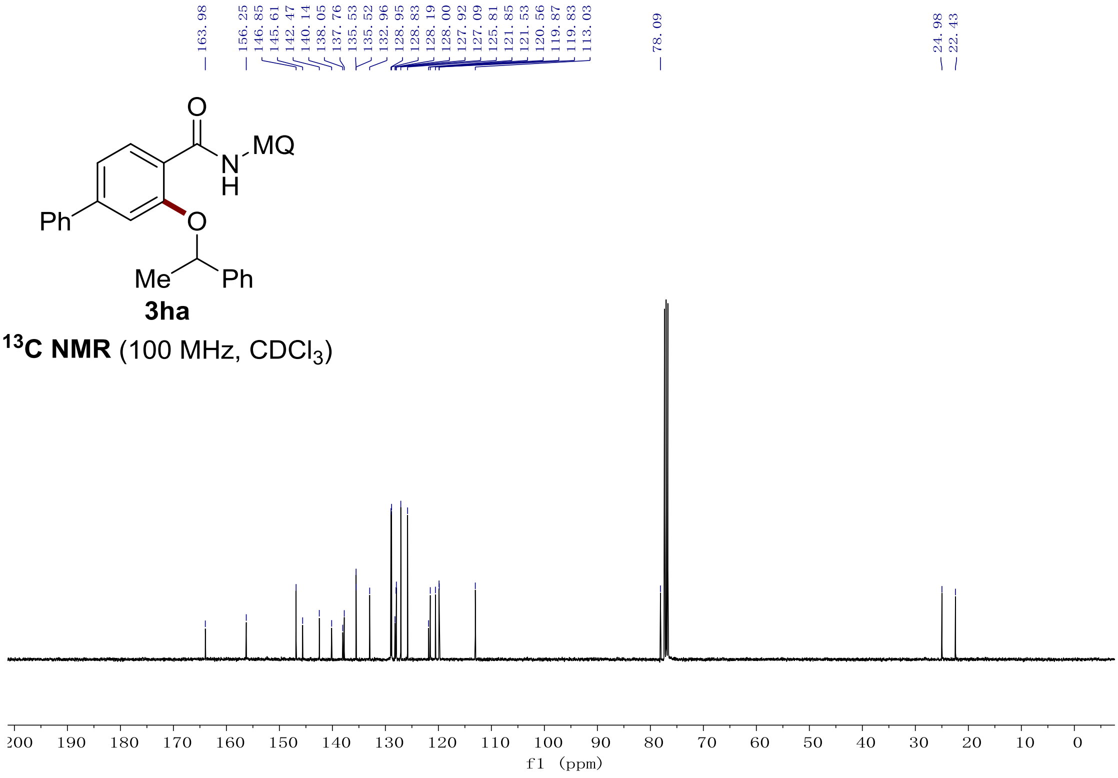

3ha

^{13}C NMR (100 MHz, $CDCl_3$)

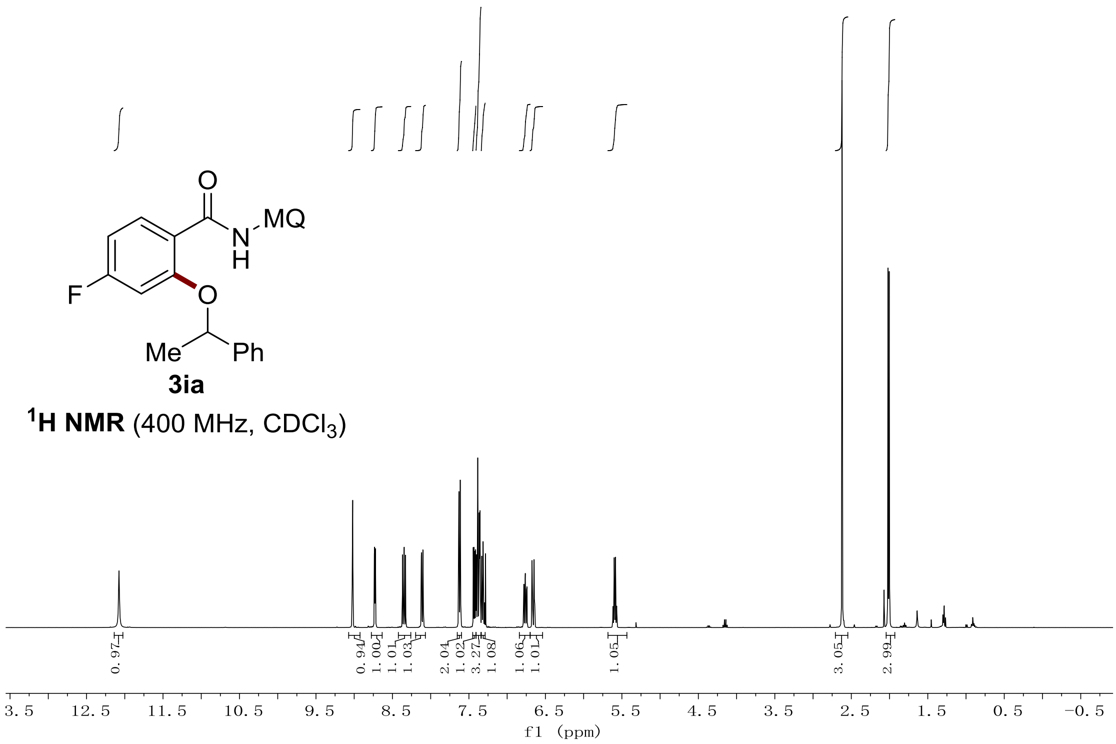
O
N
MQ
H
F
O
Me
Ph
3ia
1H NMR (400 MHz, CDCl3)
0.97
0.94
1.00
1.01
1.03
2.04
1.02
3.27
1.08
1.06
1.01
1.05
3.05
2.99
f1 (ppm)

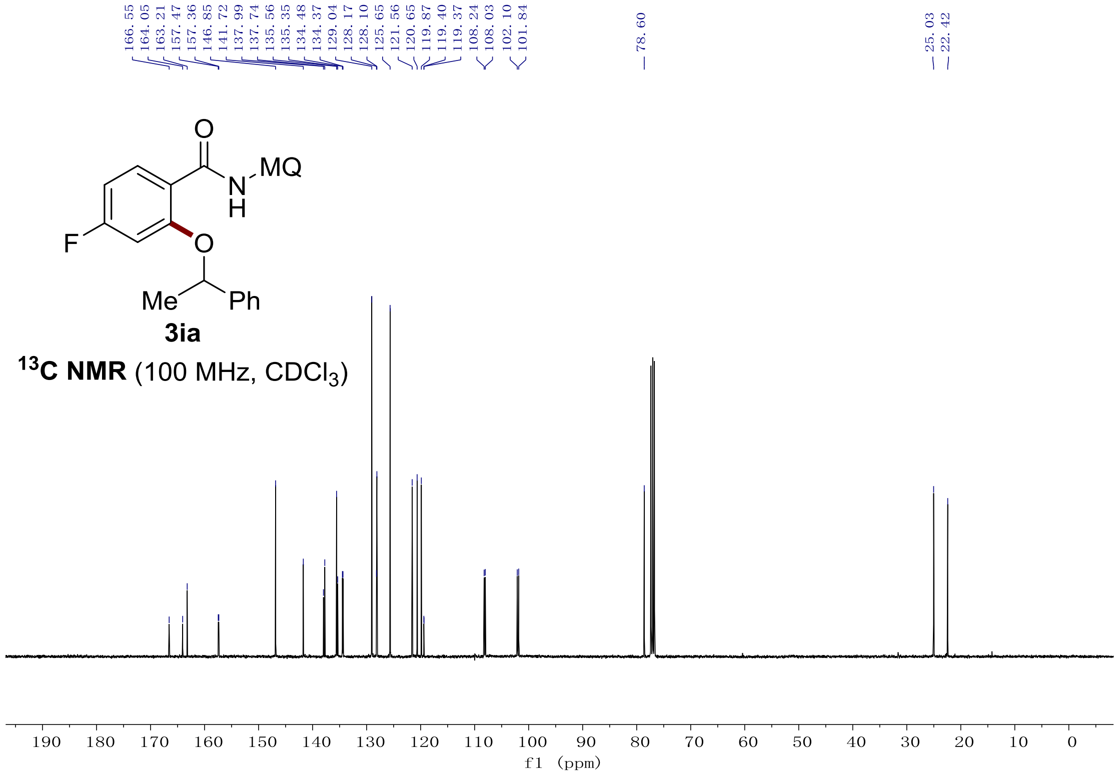
166.55
164.05
163.21
157.47
157.36
146.85
141.72
137.99
137.74
135.56
135.35
134.48
134.37
129.04
128.17
128.10
125.65
121.56
120.65
119.87
119.40
119.37
108.24
108.03
102.10
101.84
78.60
25.03
22.42
O
N
MQ
H
F
O
Me
Ph
3ia
13C NMR (100 MHz, CDCl3)
f1 (ppm)

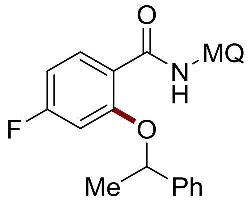

3ia

^{19}F NMR (376 MHz, $CDCl_3$)

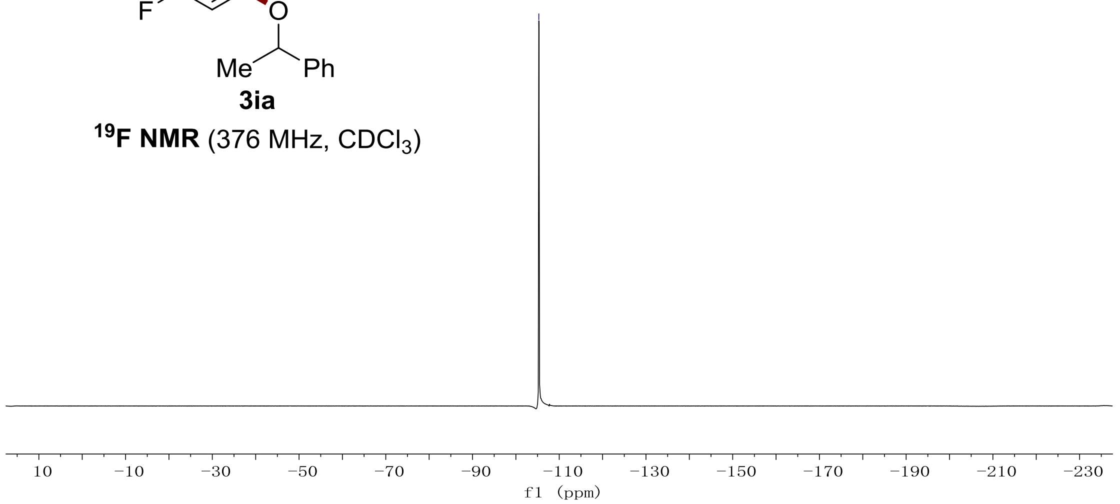

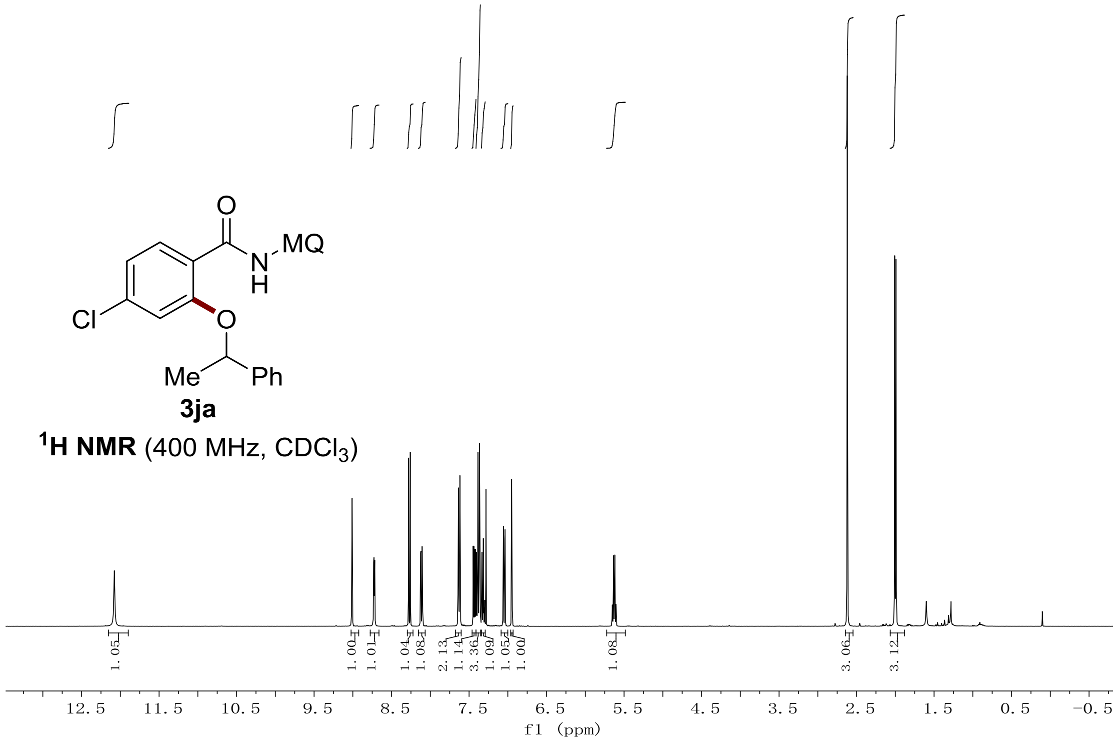

3ja

^{1}H NMR (400 MHz, $CDCl_3$)

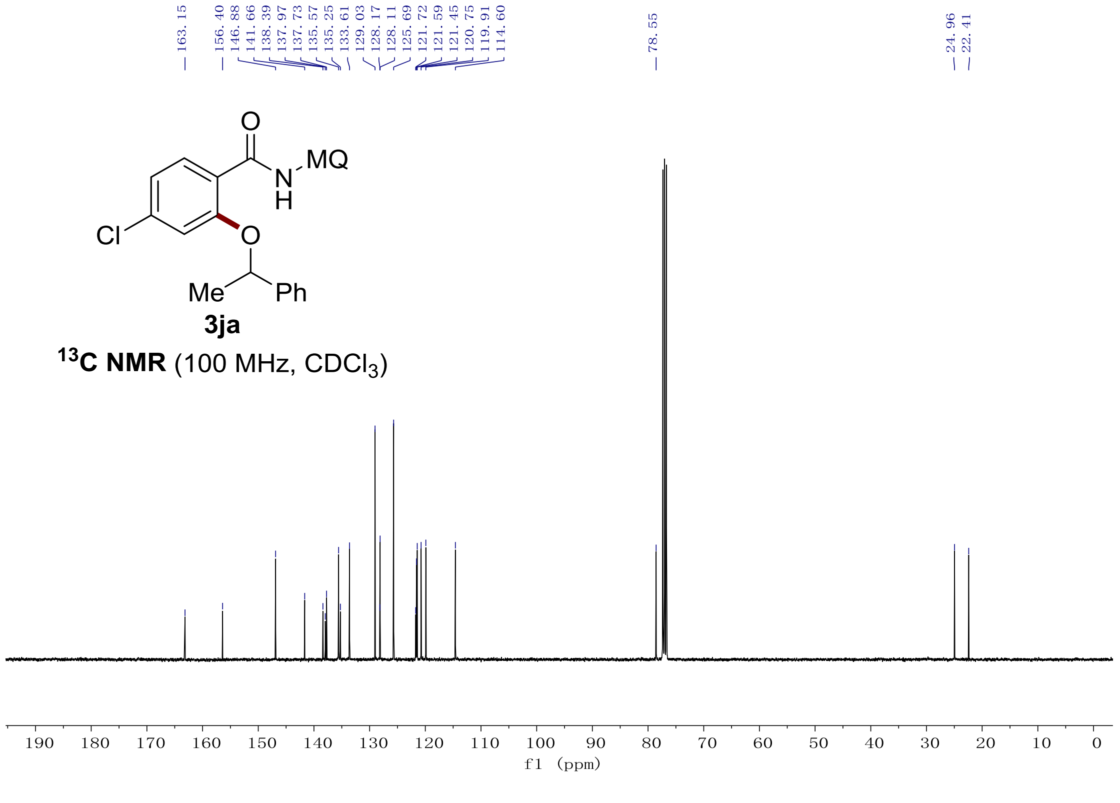

3ja

^{13}C NMR (100 MHz, $CDCl_3$)

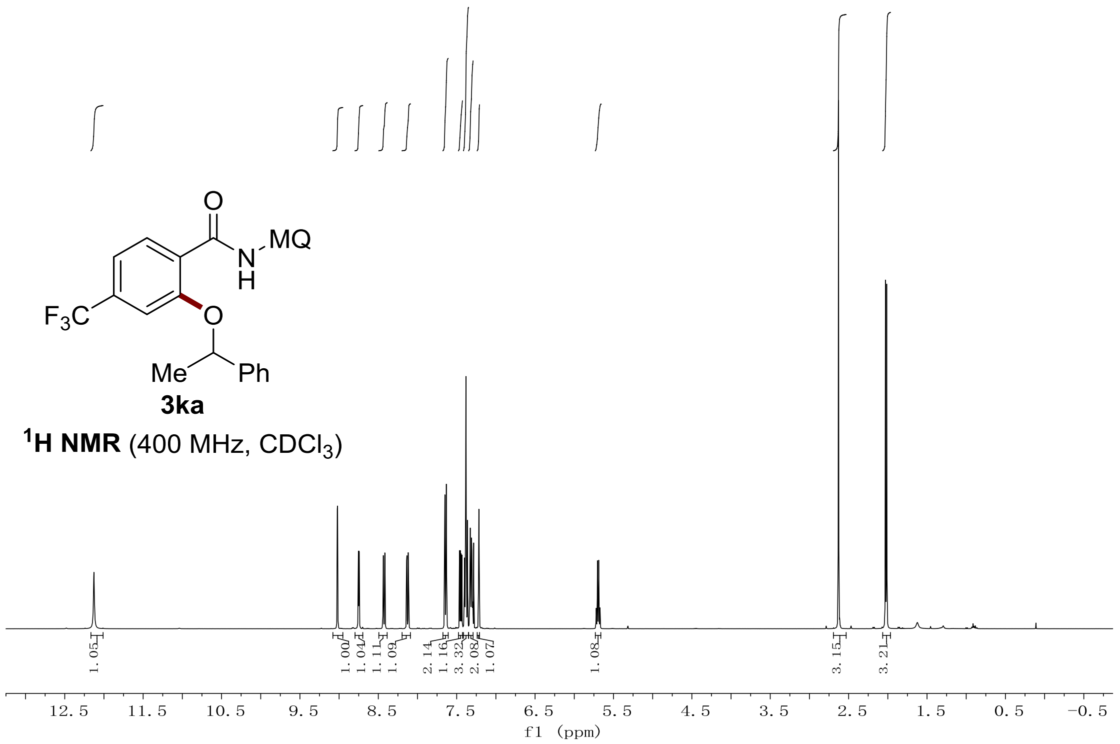
O
N
MQ
H
F3C
O
Me
Ph
3ka
1H NMR (400 MHz, CDCl3)
1.05
1.00
1.04
1.11
1.09
2.14
1.16
3.32
2.08
1.07
1.08
3.15
3.21
12.5
11.5
10.5
9.5
8.5
7.5
6.5
5.5
4.5
3.5
2.5
1.5
0.5
-0.5
f1 (ppm)

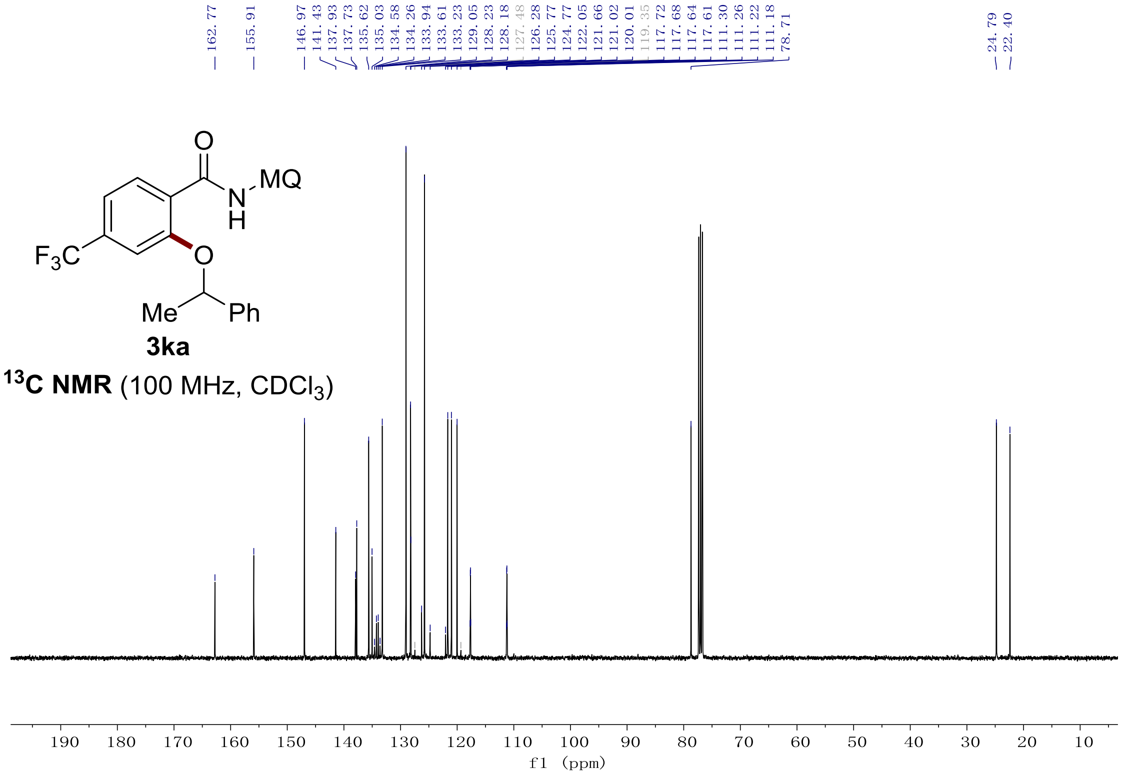
162.77
155.91
146.97
141.43
137.93
137.73
135.62
135.03
134.58
134.26
133.94
133.61
133.23
129.05
128.23
128.18
127.48
126.28
125.77
124.77
122.05
121.66
121.02
120.01
119.35
117.72
117.68
117.64
117.61
111.30
111.26
111.22
111.18
78.71
24.79
22.40
O
N
MQ
H
F3C
O
Me
Ph
3ka
13C NMR (100 MHz, CDCl3)
190
180
170
160
150
140
130
120
110
100
90
80
70
60
50
40
30
20
10
f1 (ppm)

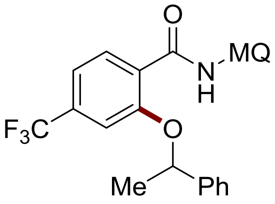

3ka

^{19}F NMR (376 MHz, $CDCl_3$)

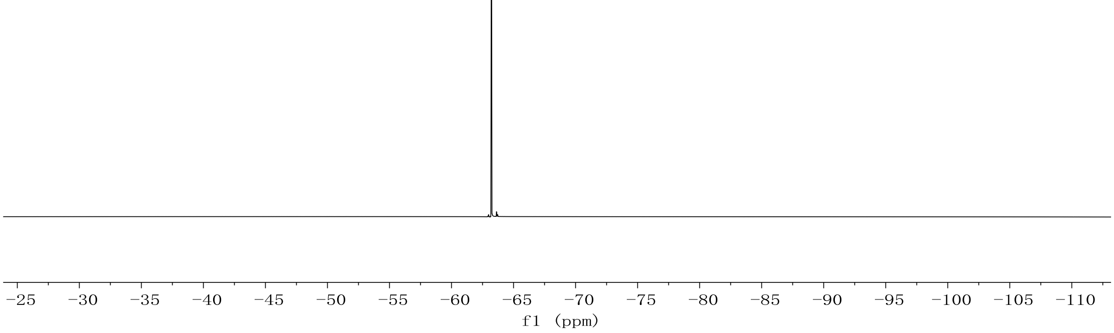

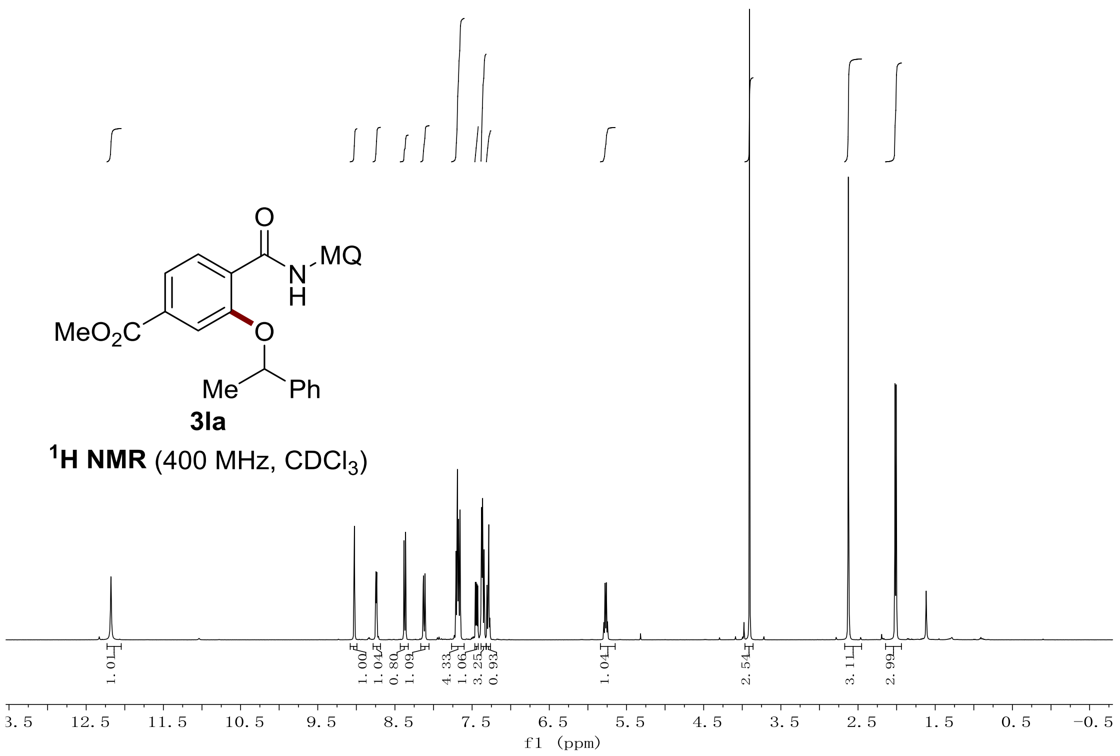
O
N
MQ
H
MeO2C
O
Me
Ph
3la
1H NMR (400 MHz, CDCl3)
1.01
1.00
1.04
0.80
1.09
4.33
1.06
3.25
0.93
1.04
2.54
3.11
2.99
f1 (ppm)

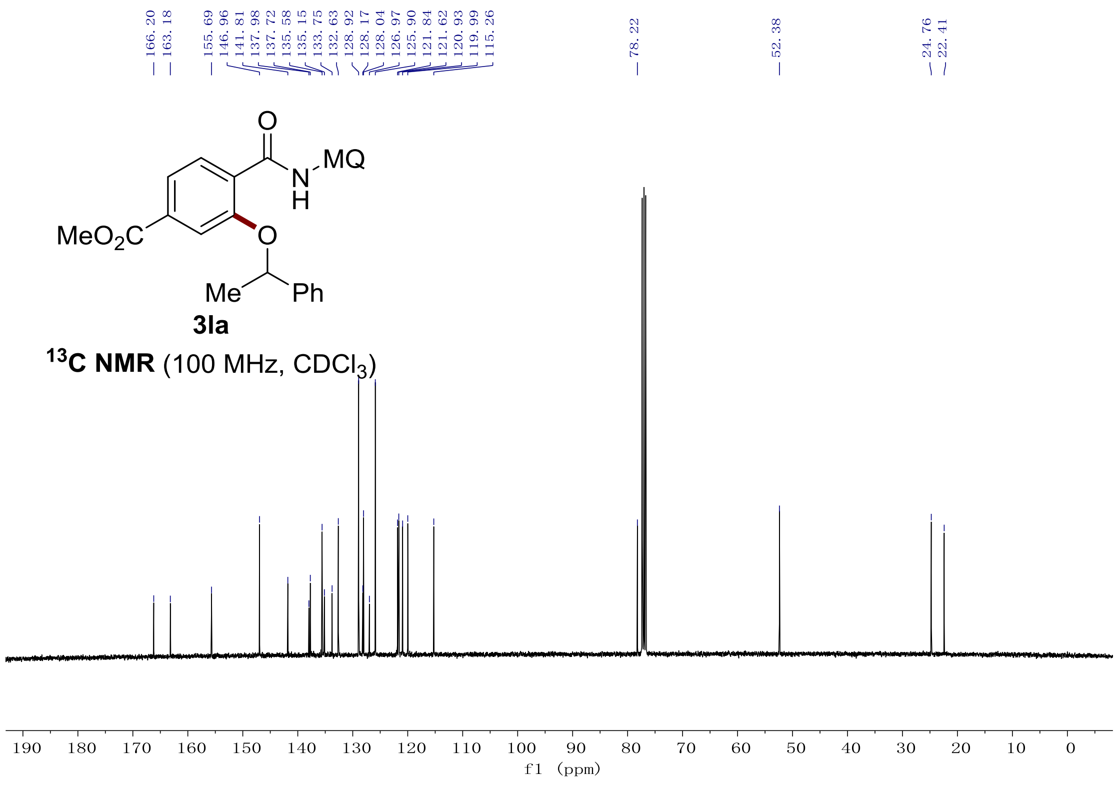
166.20
163.18
155.69
146.96
141.81
137.98
137.72
135.58
135.15
133.75
132.63
128.92
128.17
128.04
126.97
125.90
121.84
121.62
120.93
119.99
115.26
78.22
52.38
24.76
22.41
O
N
MQ
H
MeO2C
O
Me
Ph
3la
13C NMR (100 MHz, CDCl3)
f1 (ppm)

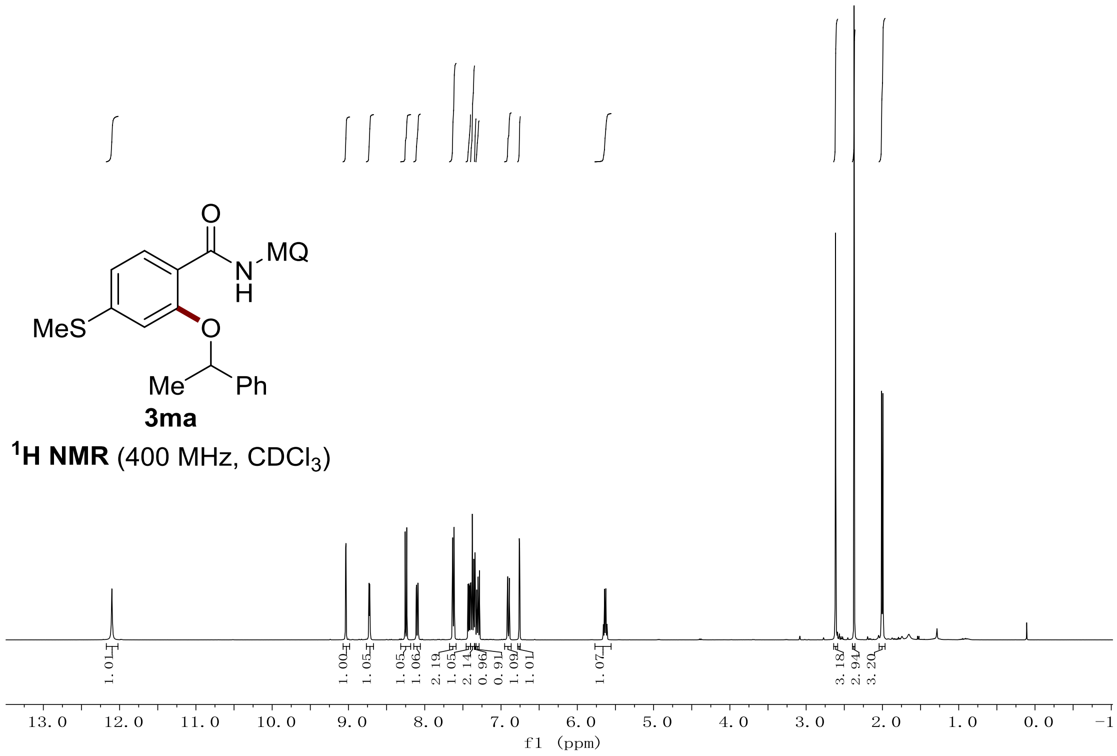
MQ
MeS
Me
Ph
3ma
^{1}H NMR (400 MHz, CDCl$_3$)
1.01
1.00
1.05
1.05
1.06
2.19
1.05
2.14
0.96
0.91
1.09
1.01
1.07
3.18
2.94
3.20
f1 (ppm)

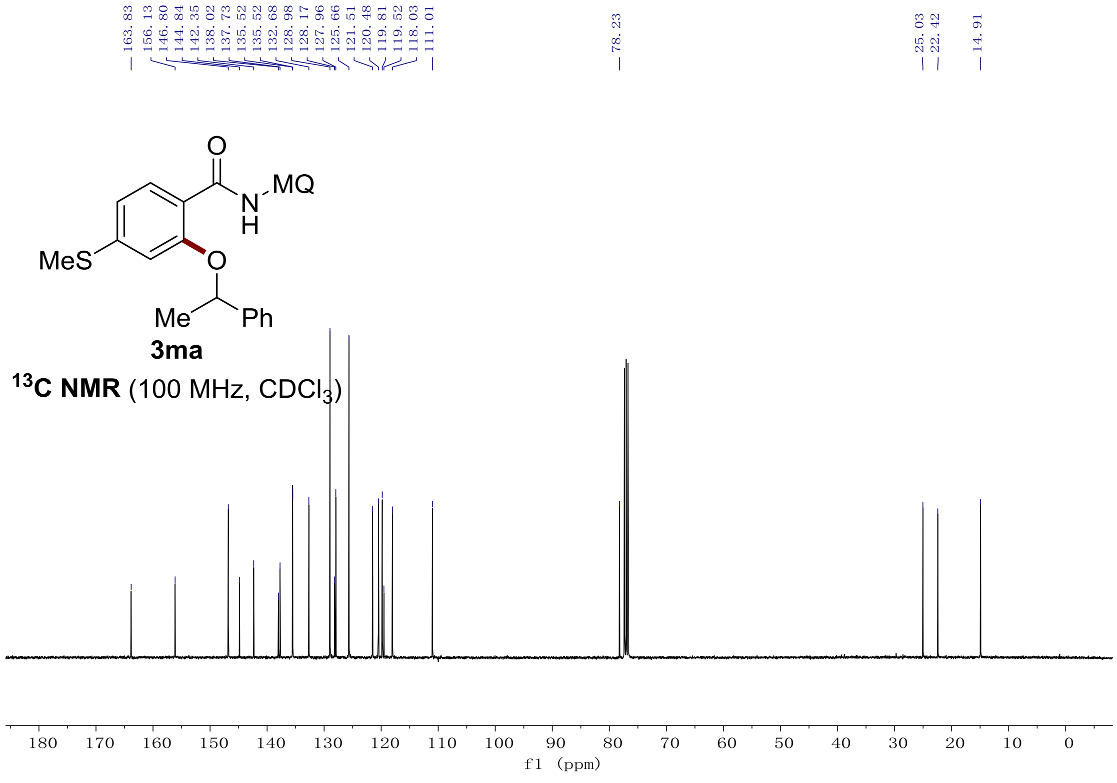
163.83
156.13
146.80
144.84
142.35
138.02
137.73
135.52
135.52
132.68
128.98
128.17
127.96
125.66
121.51
120.48
119.81
119.52
118.03
111.01
78.23
25.03
22.42
14.91
MQ
MeS
Me
Ph
3ma
^{13}C NMR (100 MHz, CDCl$_3$)
f1 (ppm)

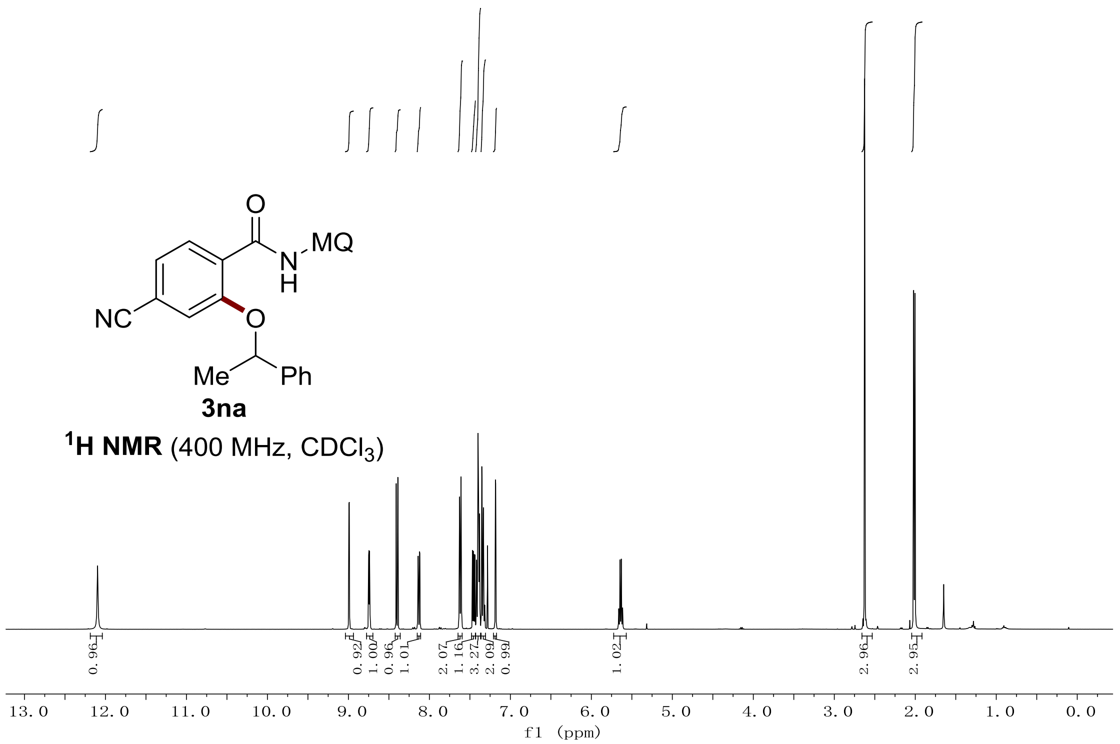

O
N
H
MQ
NC
O
Me
Ph
3na
^{1}H NMR (400 MHz, CDCl$_3$)
0.96
0.92
1.00
0.96
1.01
2.07
1.16
3.27
2.09
0.99
1.02
2.96
2.95
13.0 12.0 11.0 10.0 9.0 8.0 7.0 6.0 5.0 4.0 3.0 2.0 1.0 0.0
f1 (ppm)

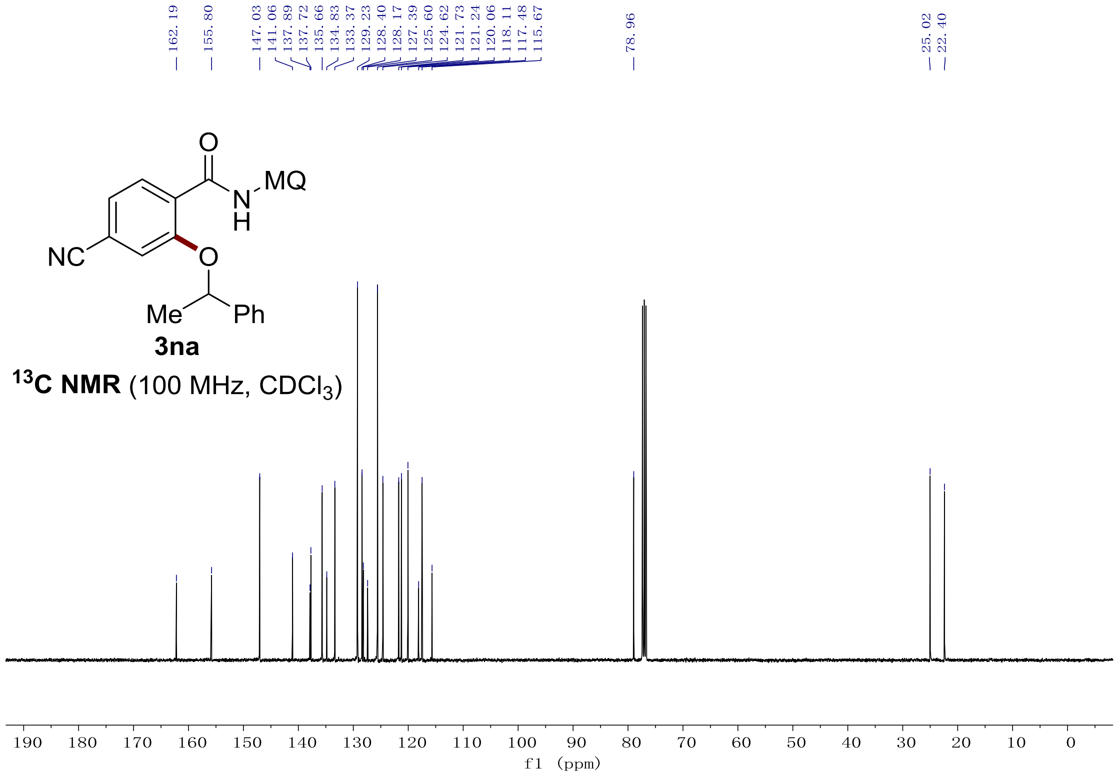

162.19
155.80
147.03
141.06
137.89
137.72
135.66
134.83
133.37
129.23
128.40
128.17
127.39
125.60
124.62
121.73
121.24
120.06
118.11
117.48
115.67
78.96
25.02
22.40
O
N
H
MQ
NC
O
Me
Ph
3na
^{13}C NMR (100 MHz, CDCl$_3$)
190 180 170 160 150 140 130 120 110 100 90 80 70 60 50 40 30 20 10 0
f1 (ppm)

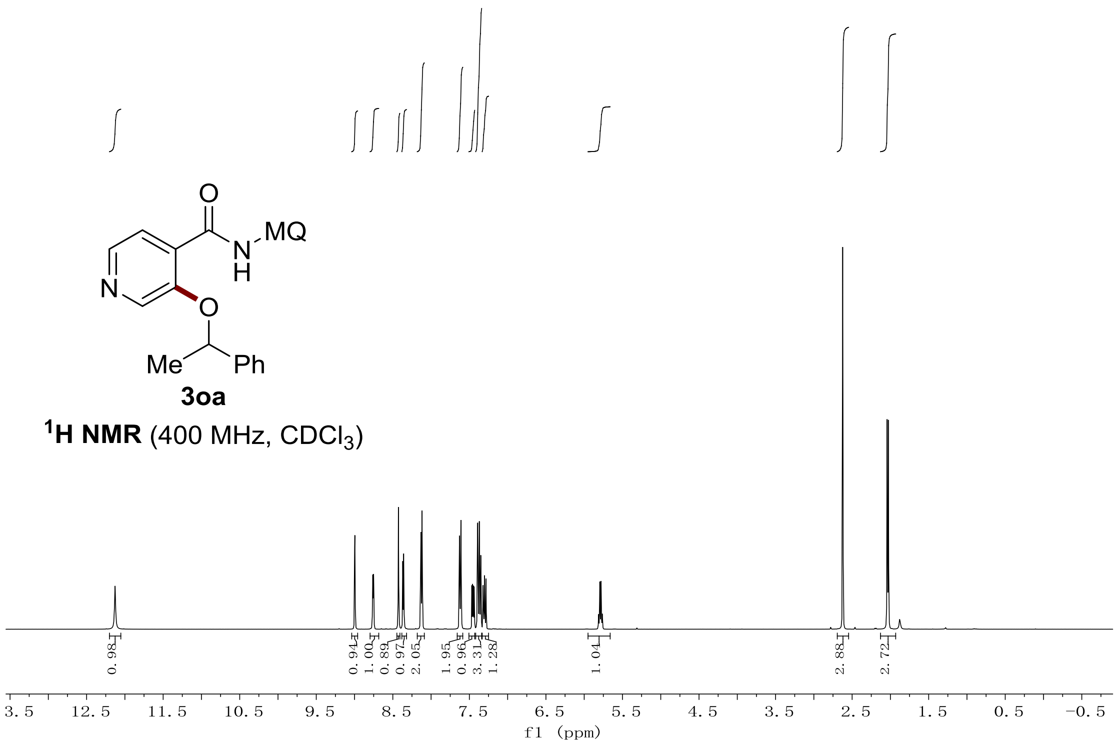
3oa
¹H NMR (400 MHz, CDCl3)
f1 (ppm)

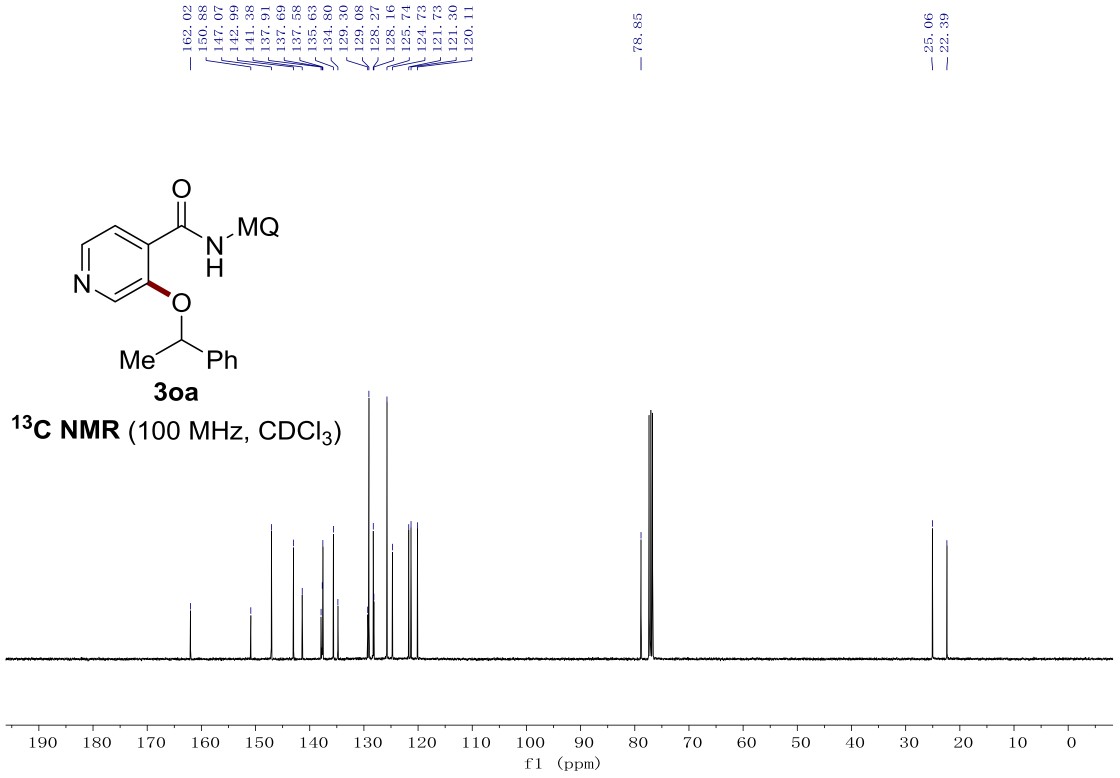
162.02
150.88
147.07
142.99
141.38
137.91
137.69
137.58
135.63
134.80
129.30
129.08
128.27
128.16
125.74
124.73
121.73
121.30
120.11
78.85
25.06
22.39
3oa
¹³C NMR (100 MHz, CDCl3)
f1 (ppm)

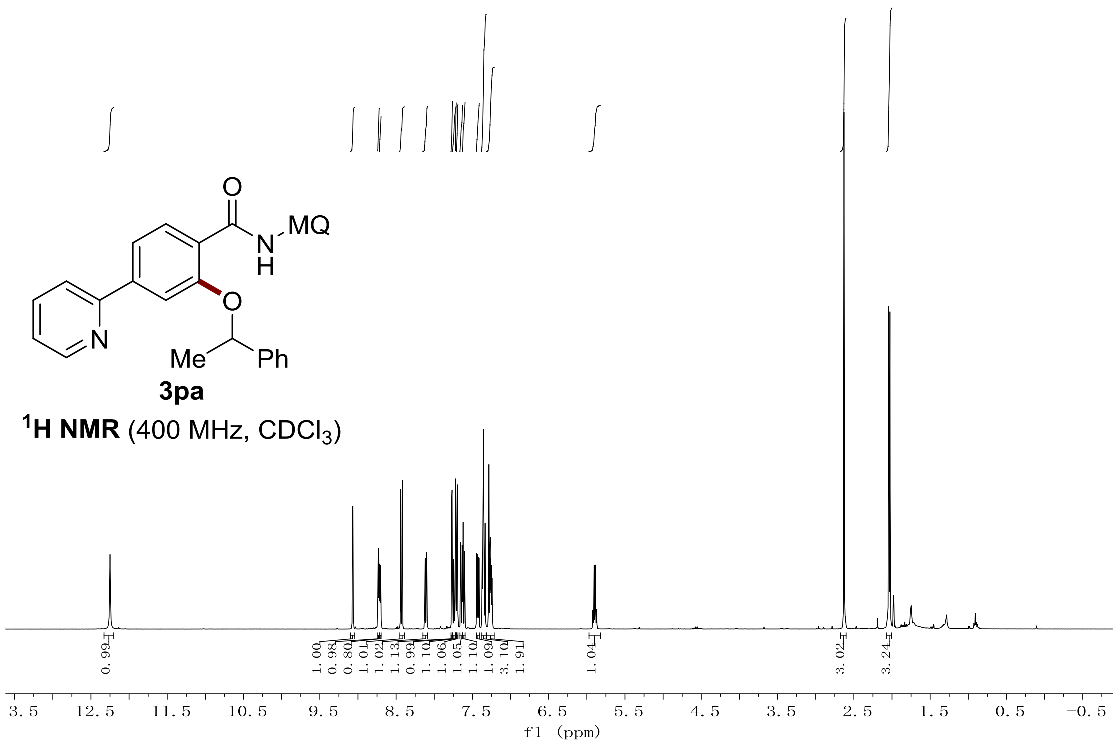
O
N
H
MQ
O
N
Me
Ph
3pa
^{1}H NMR (400 MHz, CDCl$_3$)
0.99
1.00 0.98 0.80 1.01 1.02 1.13 0.99 1.10 1.06 1.05 1.10 1.09 3.10 1.91
1.04
3.02
3.24
13.5 12.5 11.5 10.5 9.5 8.5 7.5 6.5 5.5 4.5 3.5 2.5 1.5 0.5 −0.5
f1 (ppm)

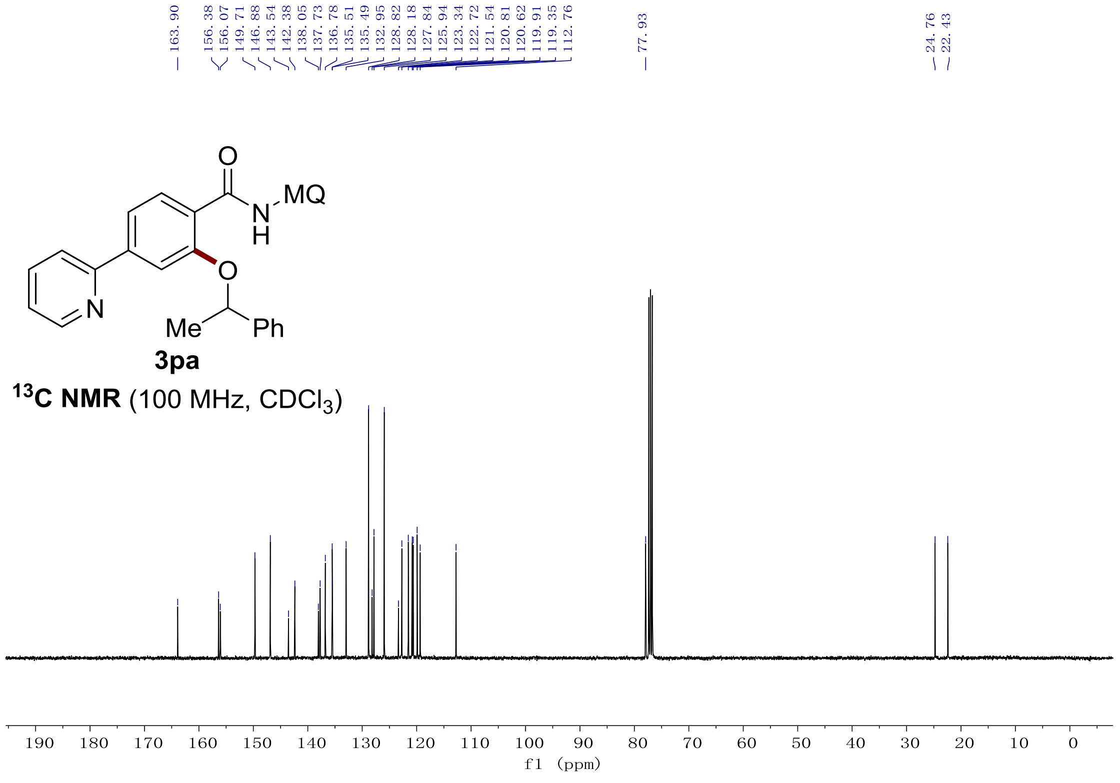
163.90
156.38 156.07 149.71 146.88 143.54 142.38 138.05 137.73 136.78 135.51 135.49 132.95 128.82 128.18 127.84 125.94 123.34 122.72 121.54 120.81 120.62 119.91 119.35 112.76
77.93
24.76 22.43
O
N
H
MQ
O
N
Me
Ph
3pa
^{13}C NMR (100 MHz, CDCl$_3$)
190 180 170 160 150 140 130 120 110 100 90 80 70 60 50 40 30 20 10 0
f1 (ppm)

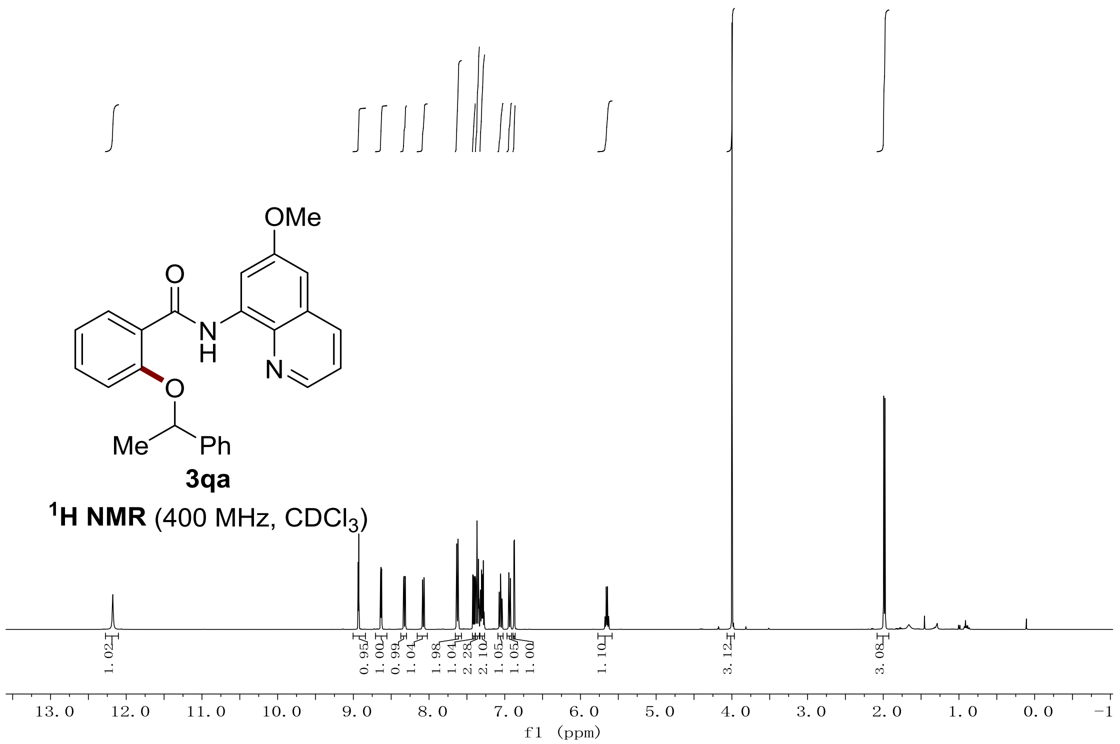
OMe
O
N
H
N
O
Me
Ph
3qa
1H NMR (400 MHz, CDCl3)
1.02
0.95
1.00
0.99
1.04
1.98
1.04
2.28
2.10
1.05
1.05
1.00
1.10
3.12
3.08
13.0
12.0
11.0
10.0
9.0
8.0
7.0
6.0
5.0
4.0
3.0
2.0
1.0
0.0
-1
f1 (ppm)

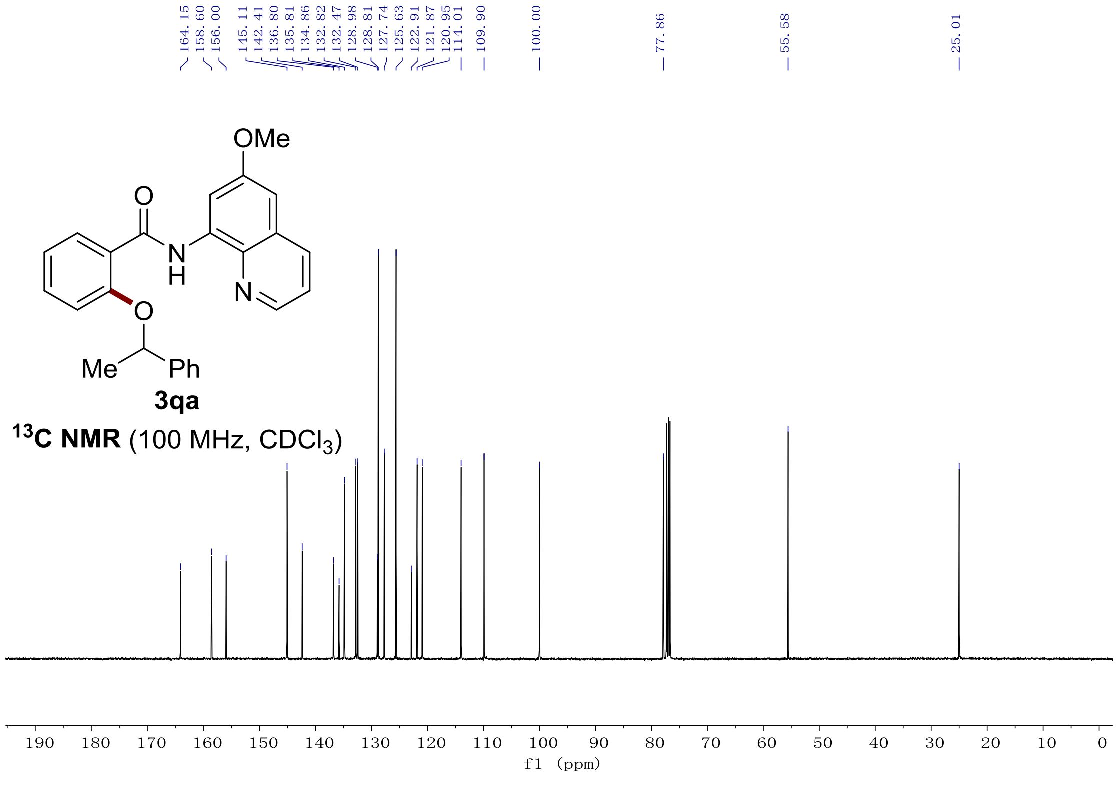
164.15
158.60
156.00
145.11
142.41
136.80
135.81
134.86
132.82
132.47
128.98
128.81
127.74
125.63
122.91
121.87
120.95
114.01
109.90
100.00
77.86
55.58
25.01
OMe
O
N
H
N
O
Me
Ph
3qa
13C NMR (100 MHz, CDCl3)
190
180
170
160
150
140
130
120
110
100
90
80
70
60
50
40
30
20
10
0
f1 (ppm)

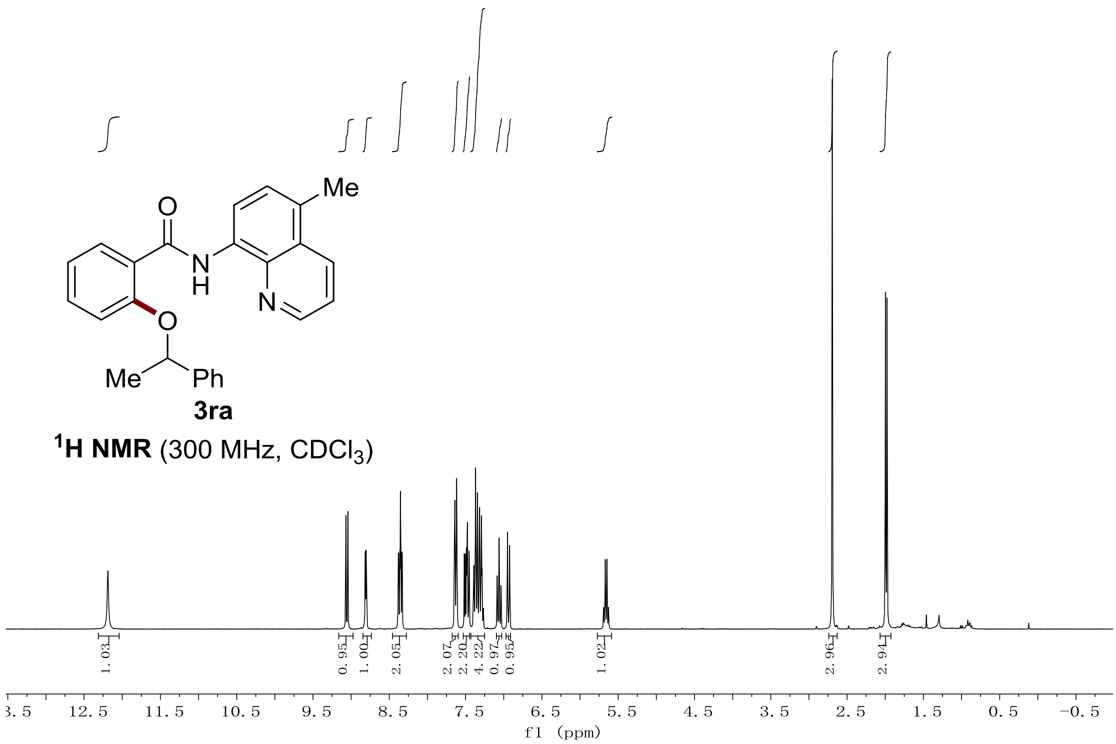

Me
O
N
H
N
O
Me
Ph
3ra
1H NMR (300 MHz, CDCl3)
1.03
0.95
1.00
2.05
2.07
2.20
4.22
0.97
0.95
1.02
2.96
2.94
f1 (ppm)

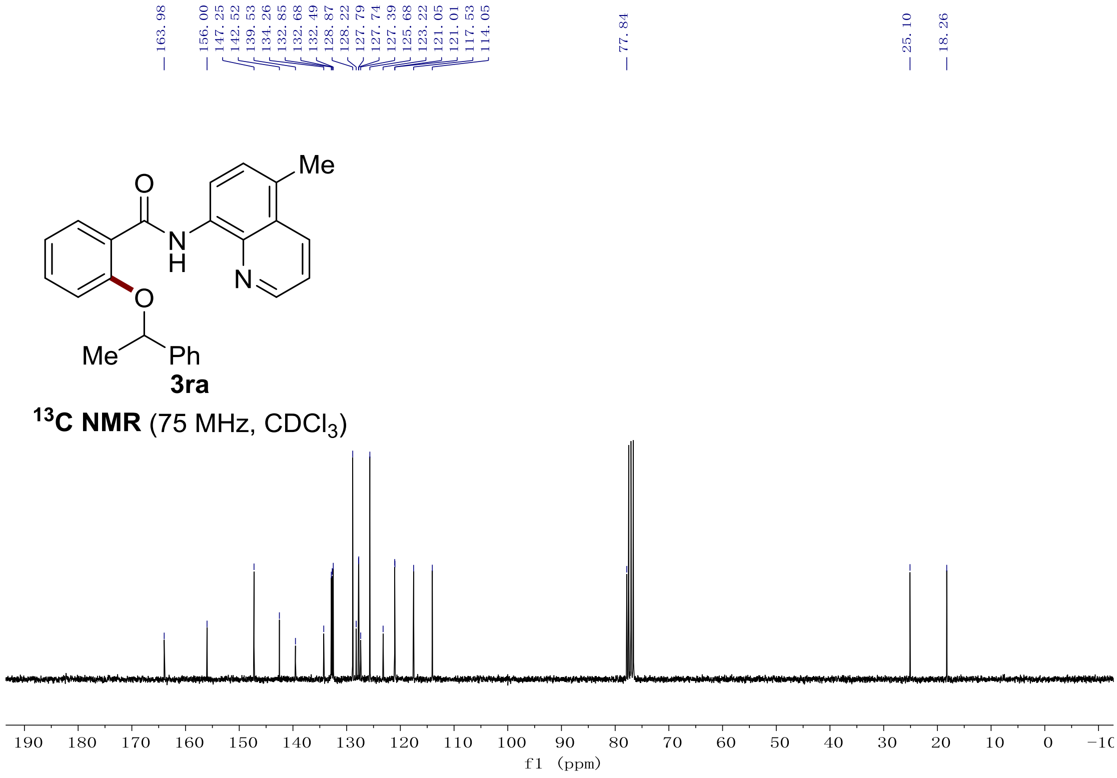

163.98
156.00
147.25
142.52
139.53
134.26
132.85
132.68
132.49
128.87
128.22
127.79
127.74
127.39
125.68
123.22
121.05
121.01
117.53
114.05
77.84
25.10
18.26
Me
O
N
H
N
O
Me
Ph
3ra
13C NMR (75 MHz, CDCl3)
f1 (ppm)

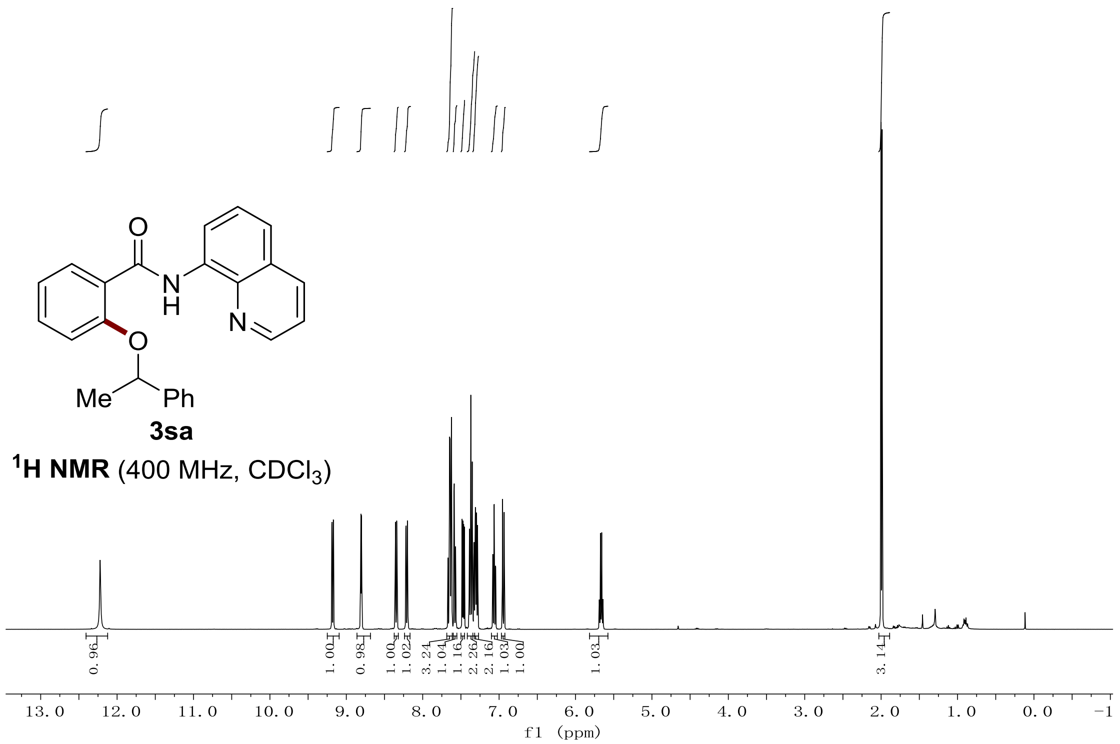

3sa

^{1}H NMR (400 MHz, $CDCl_3$)

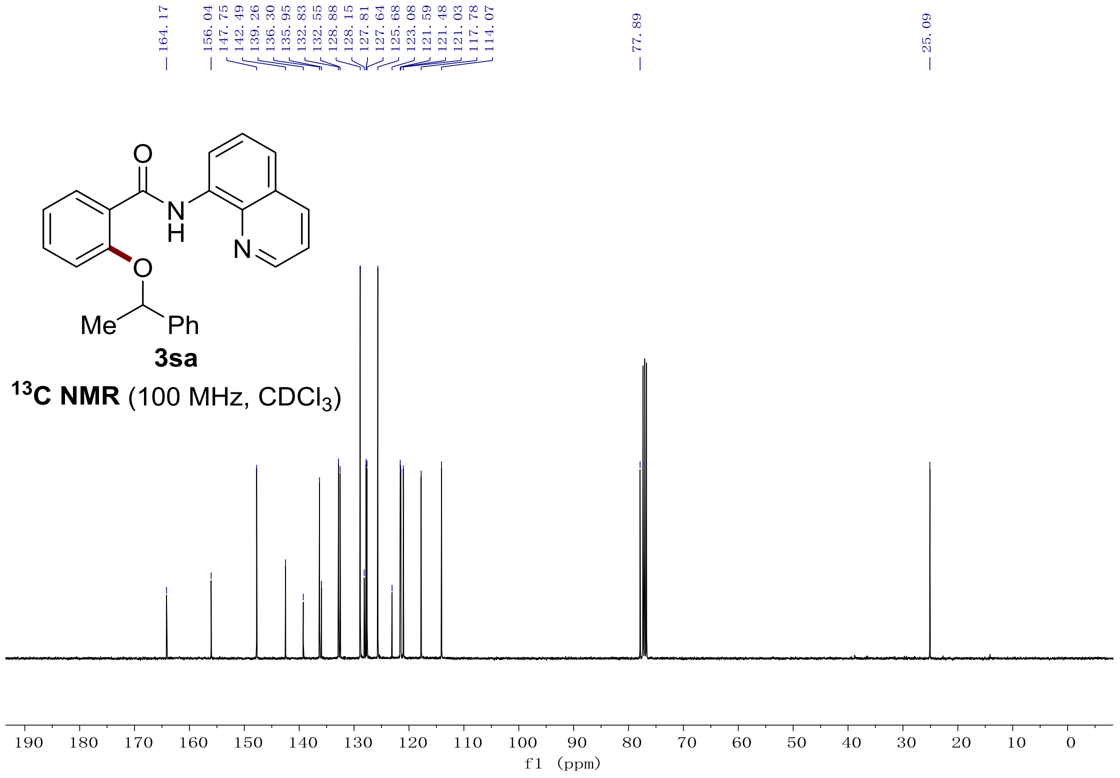

3sa

^{13}C NMR (100 MHz, $CDCl_3$)

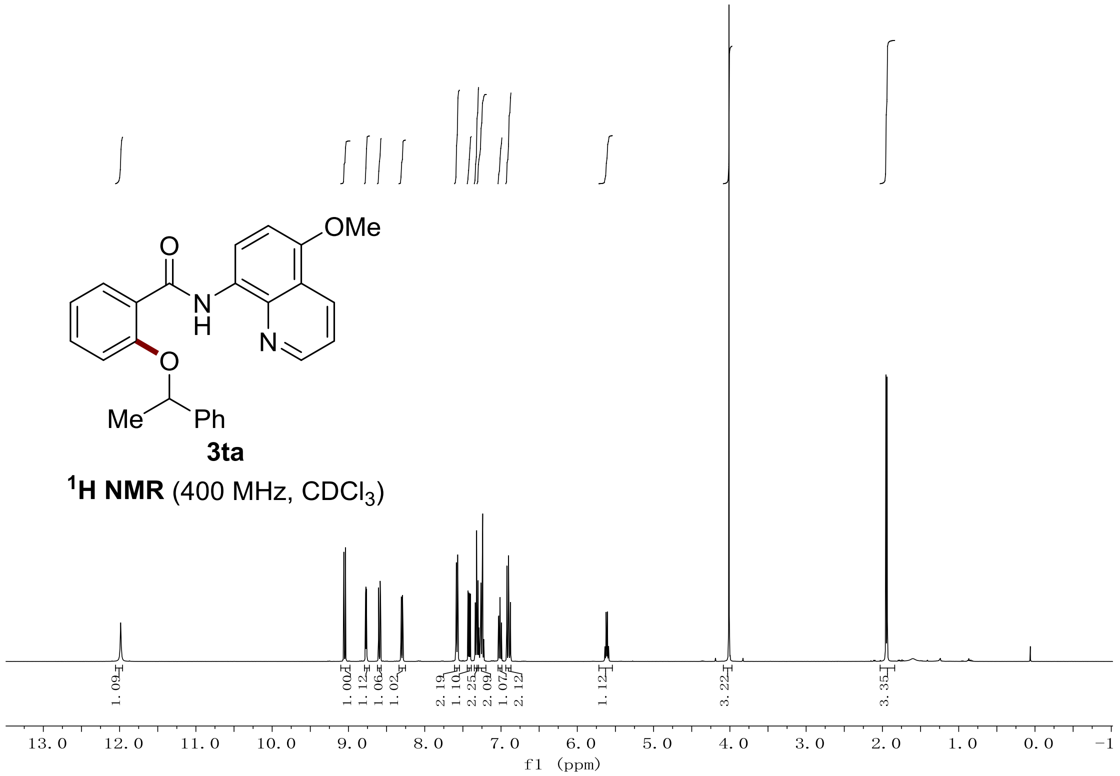

3ta

^{1}H NMR (400 MHz, $CDCl_3$)

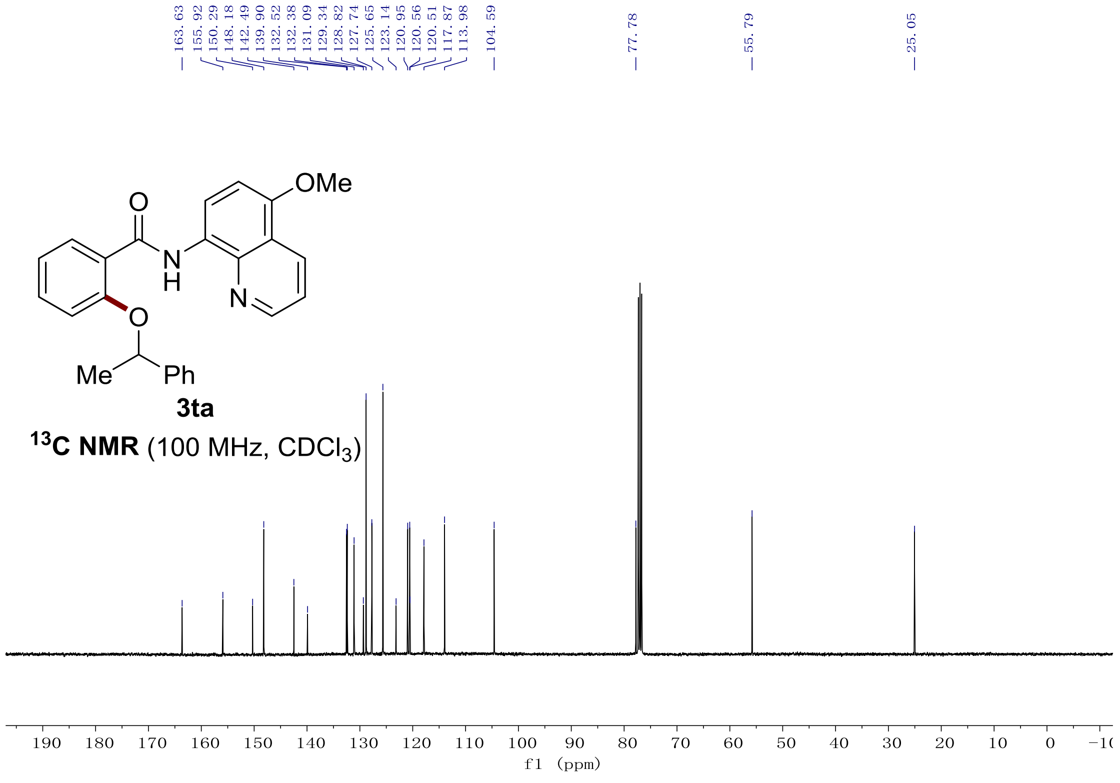

3ta

^{13}C NMR (100 MHz, $CDCl_3$)

3xa

¹H NMR (400 MHz, $CDCl_3$)

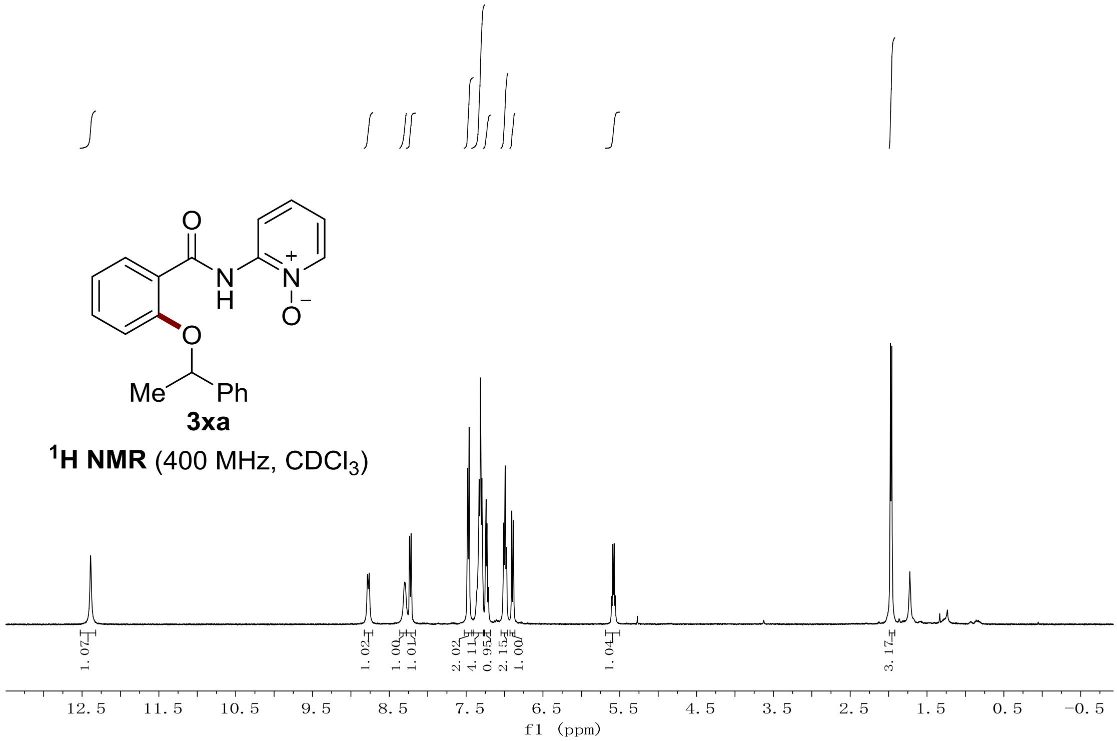

3xa

¹³C NMR (100 MHz, $CDCl_3$)

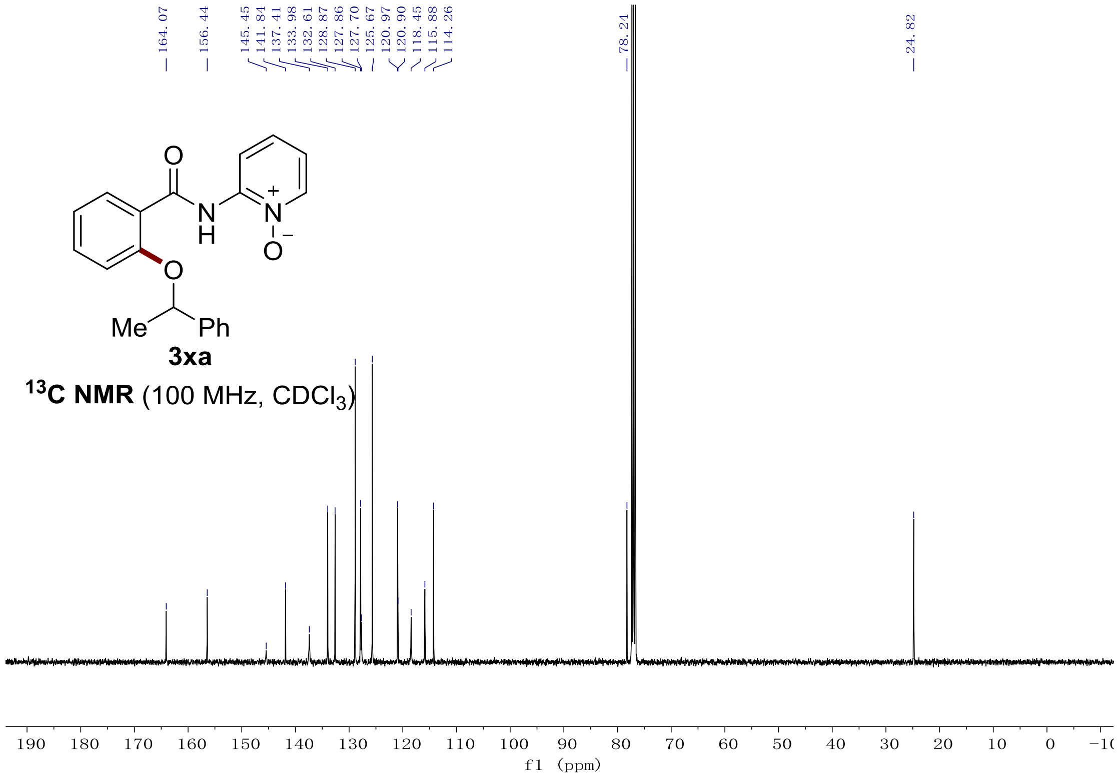

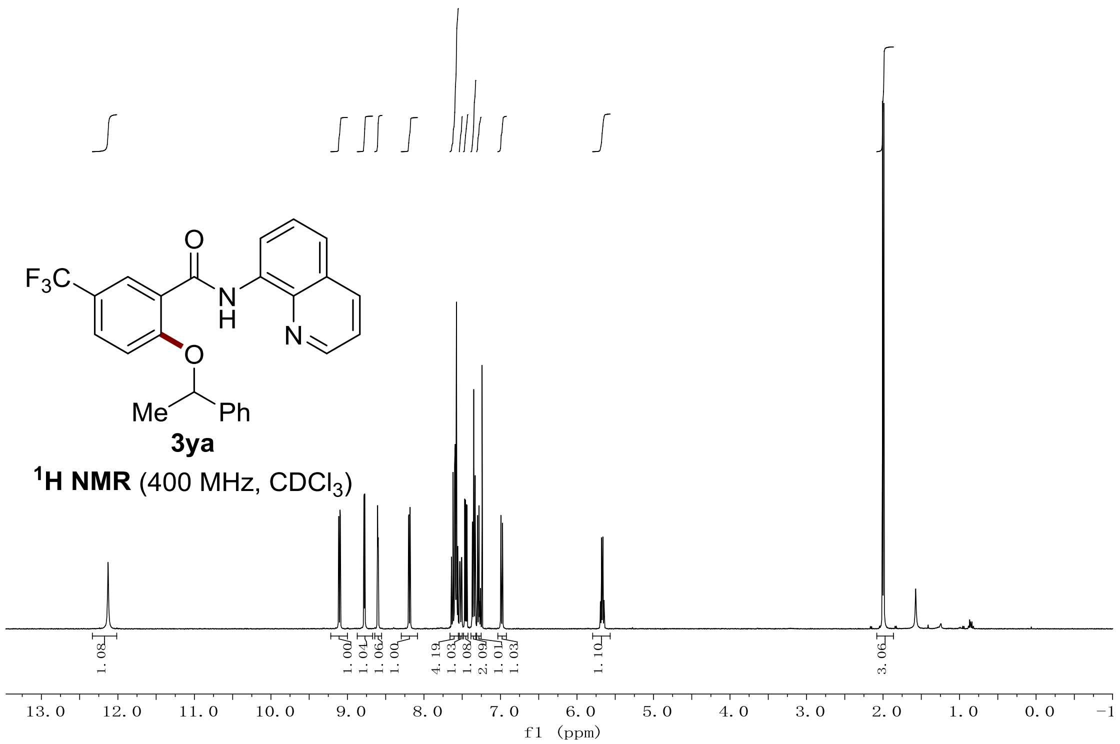

F3C
O
N
H
N
O
Me
Ph
3ya
1H NMR (400 MHz, CDCl3)
1.08
1.00
1.04
1.06
1.00
4.19
1.03
1.08
2.09
1.01
1.03
1.10
3.06
f1 (ppm)

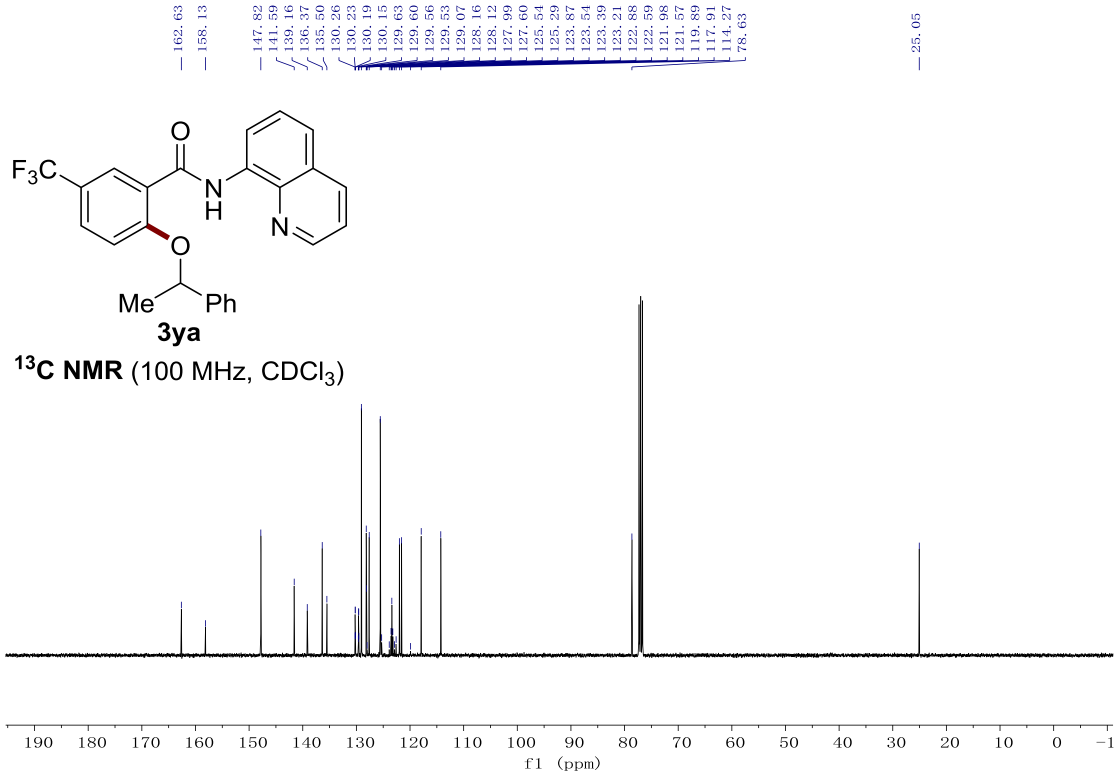

162.63
158.13
147.82
141.59
139.16
136.37
135.50
130.26
130.23
130.19
130.15
129.63
129.60
129.56
129.53
129.07
128.16
128.12
127.99
127.60
125.54
125.29
123.87
123.54
123.39
123.21
122.88
122.59
121.98
121.57
119.89
117.91
114.27
78.63
25.05
F3C
O
N
H
N
O
Me
Ph
3ya
13C NMR (100 MHz, CDCl3)
f1 (ppm)

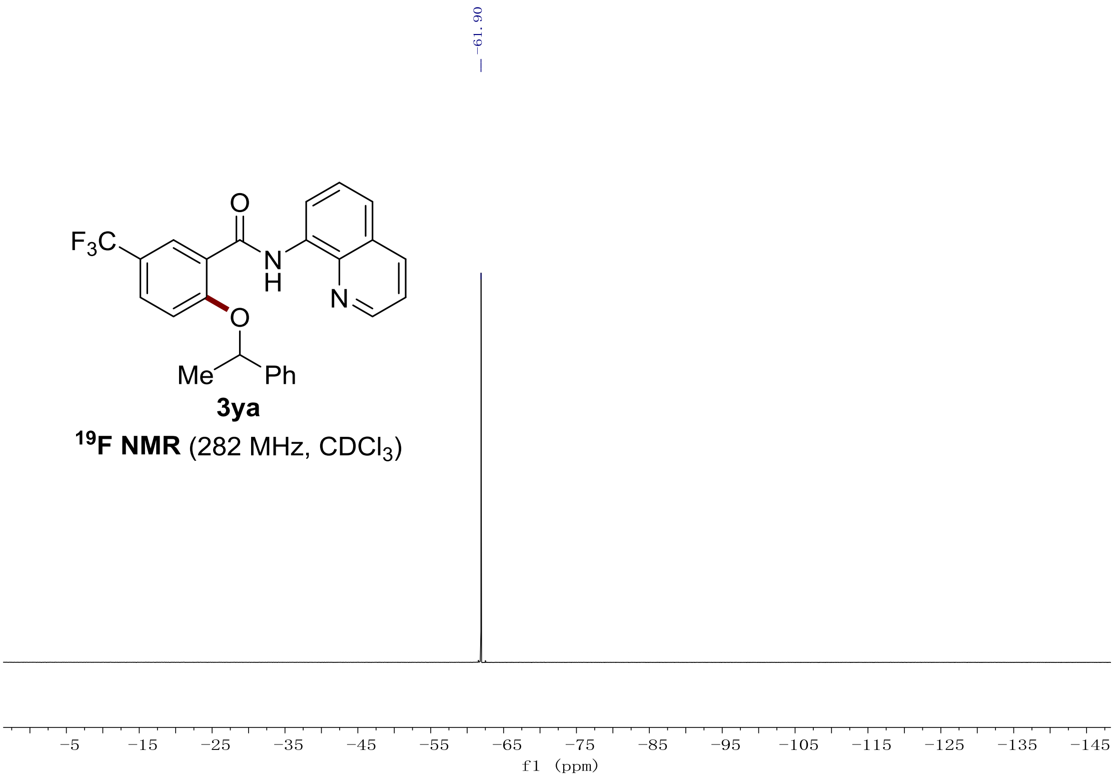

3ya

^{19}F NMR (282 MHz, $CDCl_3$)

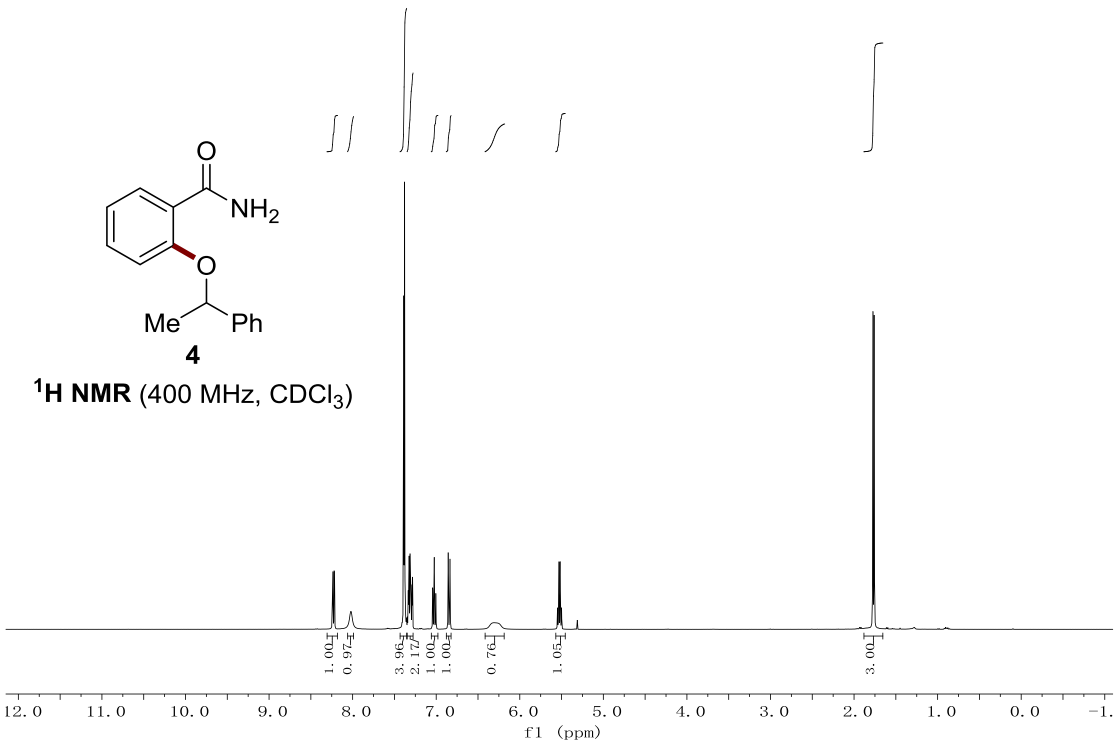

^{1}H NMR (400 MHz, $CDCl_3$)

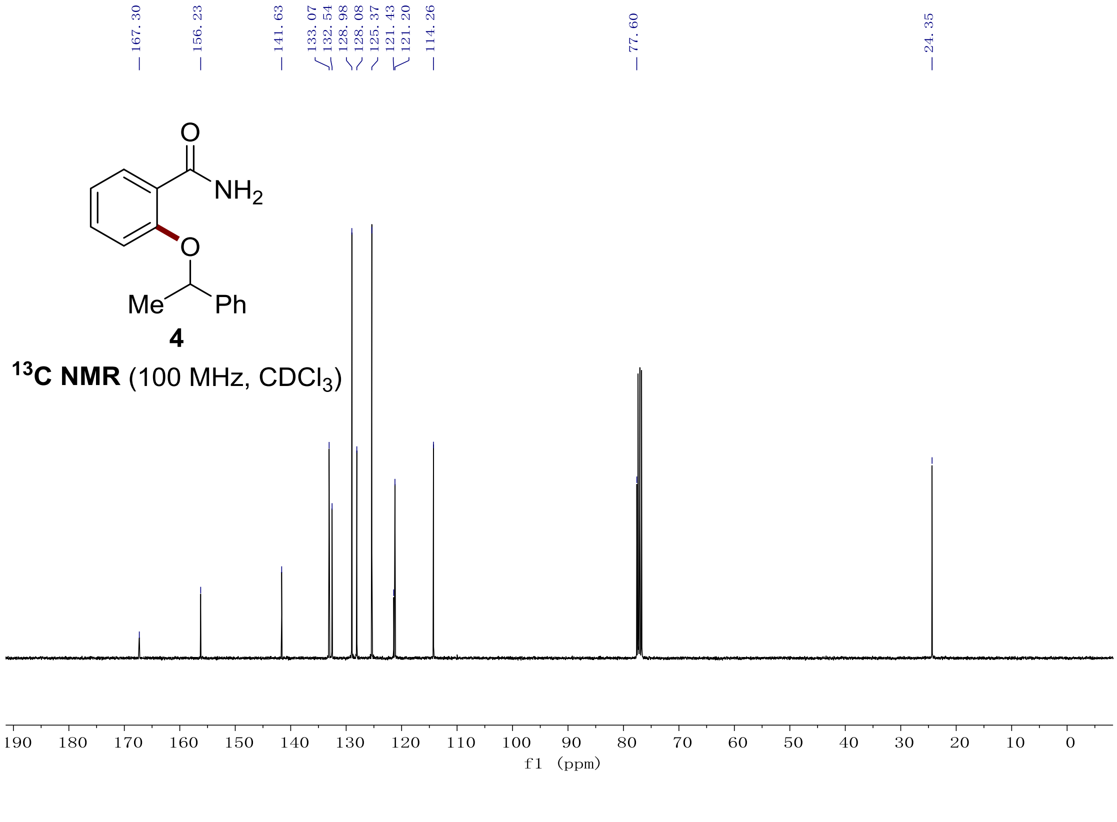

^{13}C NMR (100 MHz, $CDCl_3$)

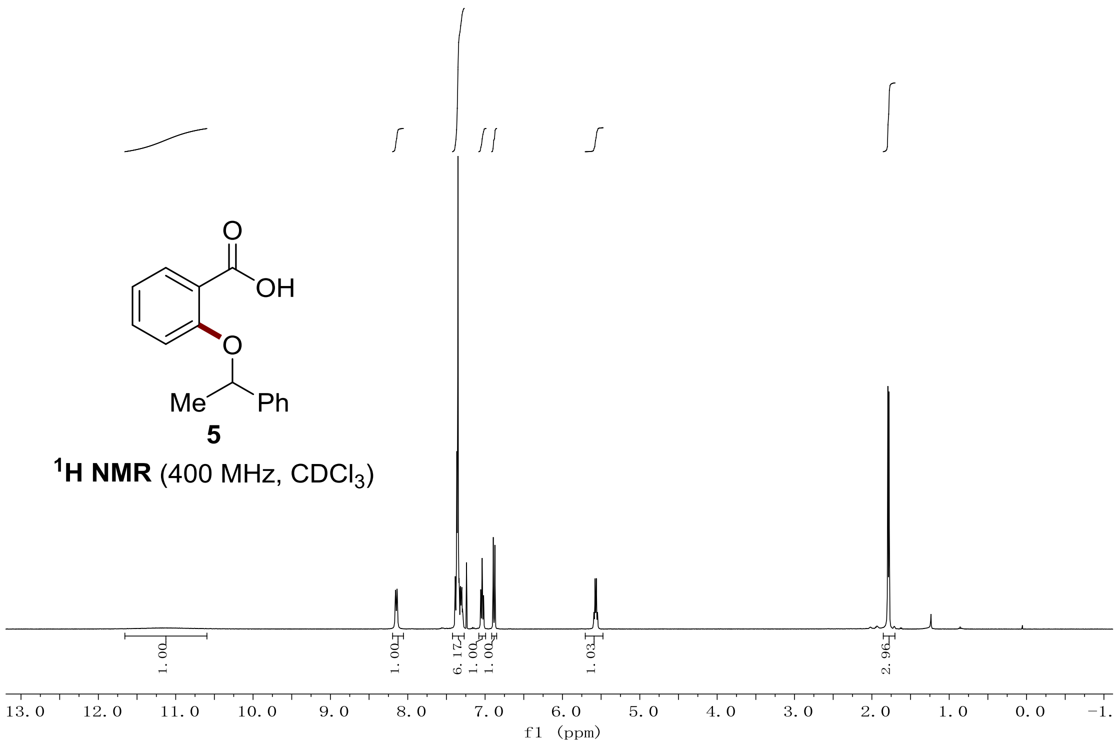
O
OH
O
Me
Ph
5
1H NMR (400 MHz, CDCl3)
1.00
1.00
6.17
1.00
1.00
1.03
2.96
13.0 12.0 11.0 10.0 9.0 8.0 7.0 6.0 5.0 4.0 3.0 2.0 1.0 0.0 -1.
f1 (ppm)

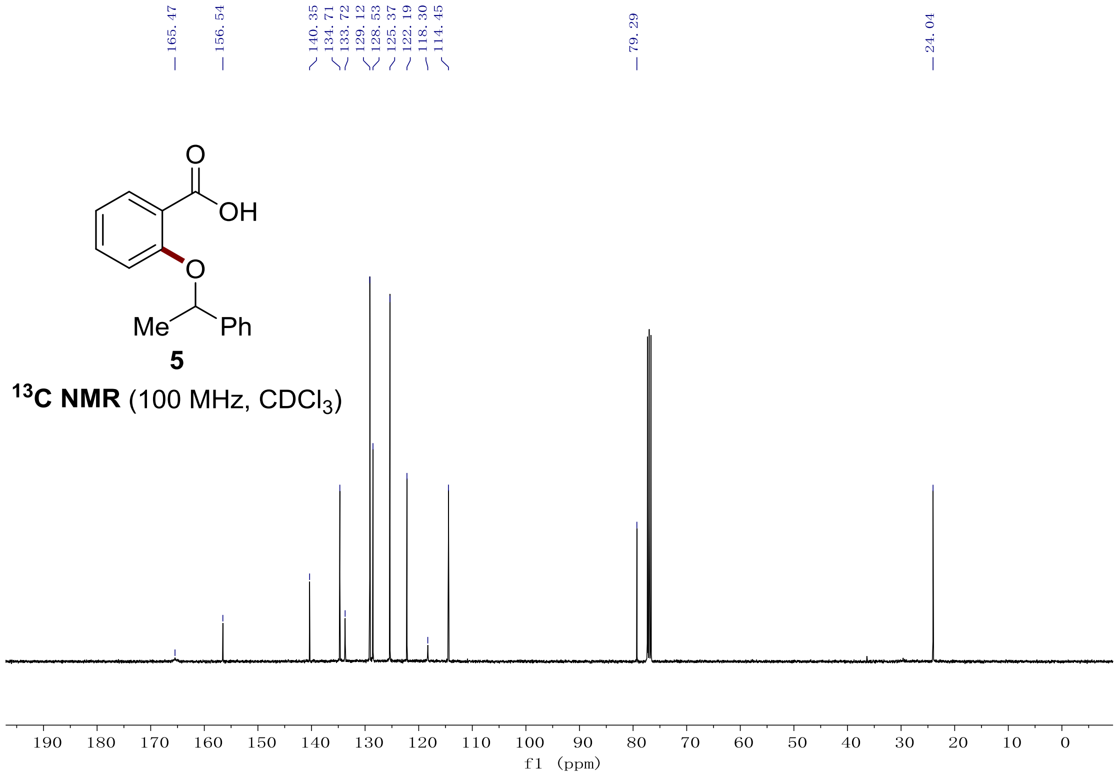
165.47
156.54
140.35
134.71
133.72
129.12
128.53
125.37
122.19
118.30
114.45
79.29
24.04
O
OH
O
Me
Ph
5
13C NMR (100 MHz, CDCl3)
190 180 170 160 150 140 130 120 110 100 90 80 70 60 50 40 30 20 10 0
f1 (ppm)

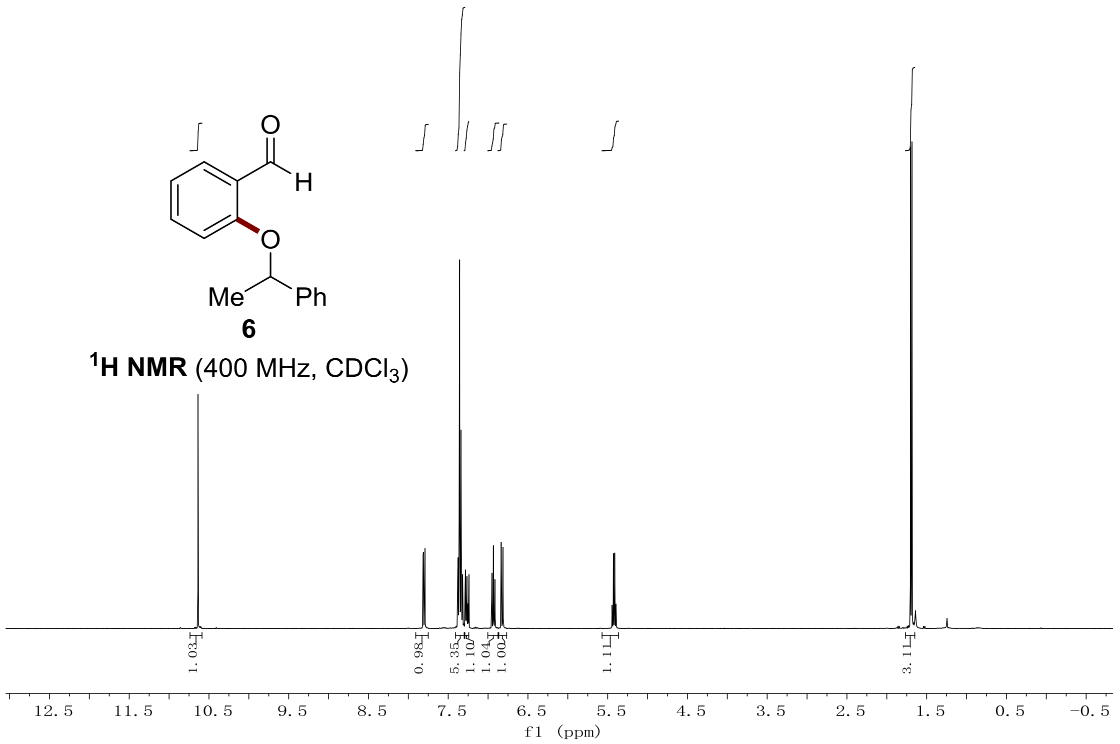
O
H
O
Me
Ph
6
1H NMR (400 MHz, CDCl3)
1.03
0.98
5.35
1.10
1.04
1.00
1.11
3.11
12.5
11.5
10.5
9.5
8.5
7.5
6.5
5.5
4.5
3.5
2.5
1.5
0.5
−0.5
f1 (ppm)

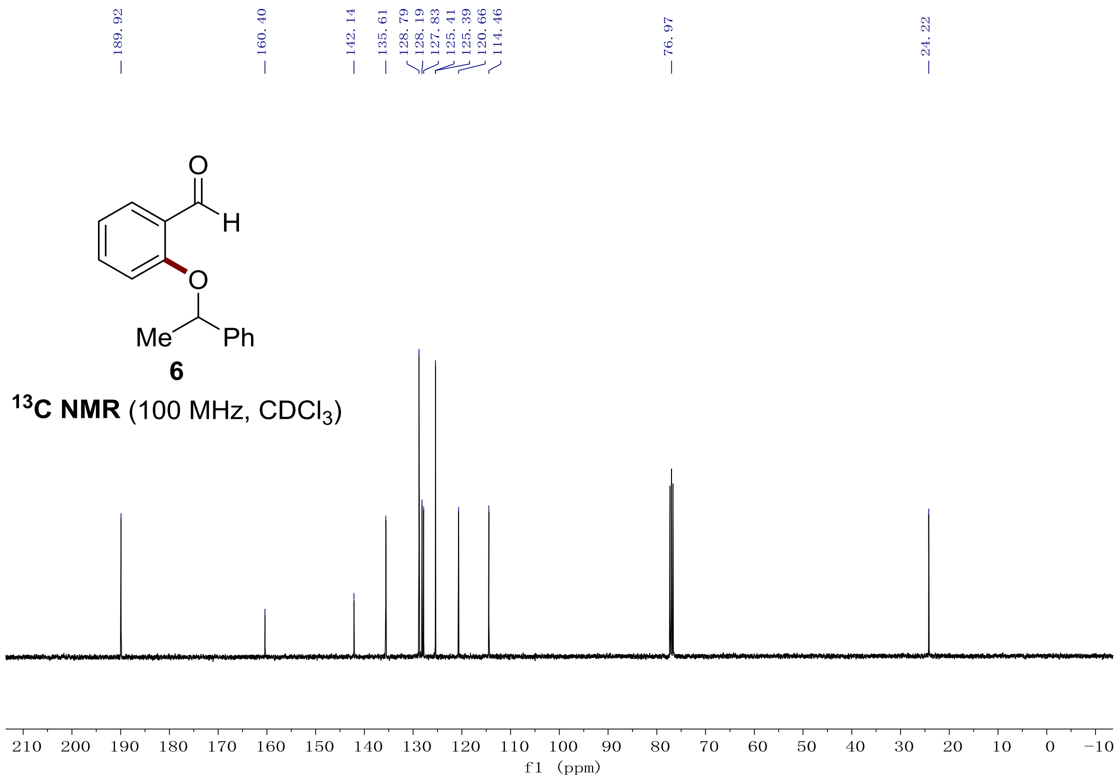
189.92
160.40
142.14
135.61
128.79
128.19
127.83
125.41
125.39
120.66
114.46
76.97
24.22
O
H
O
Me
Ph
6
13C NMR (100 MHz, CDCl3)
210
200
190
180
170
160
150
140
130
120
110
100
90
80
70
60
50
40
30
20
10
0
−10
f1 (ppm)
